# Distinctive Immune Signatures Driven by Structural Alterations in Desmuramylpeptide NOD2 Agonists

**DOI:** 10.1021/acs.jmedchem.4c01577

**Published:** 2024-09-30

**Authors:** Špela Janež, Samo Guzelj, Petra Kocbek, Eveline A. de Vlieger, Bram Slütter, Žiga Jakopin

**Affiliations:** † Faculty of Pharmacy, University of Ljubljana, SI-1000 Ljubljana, Slovenia; ‡ Div. BioTherapeutics, Leiden Academic Centre for Drug Research, Leiden University, 2333 CC Leiden, The Netherlands

## Abstract

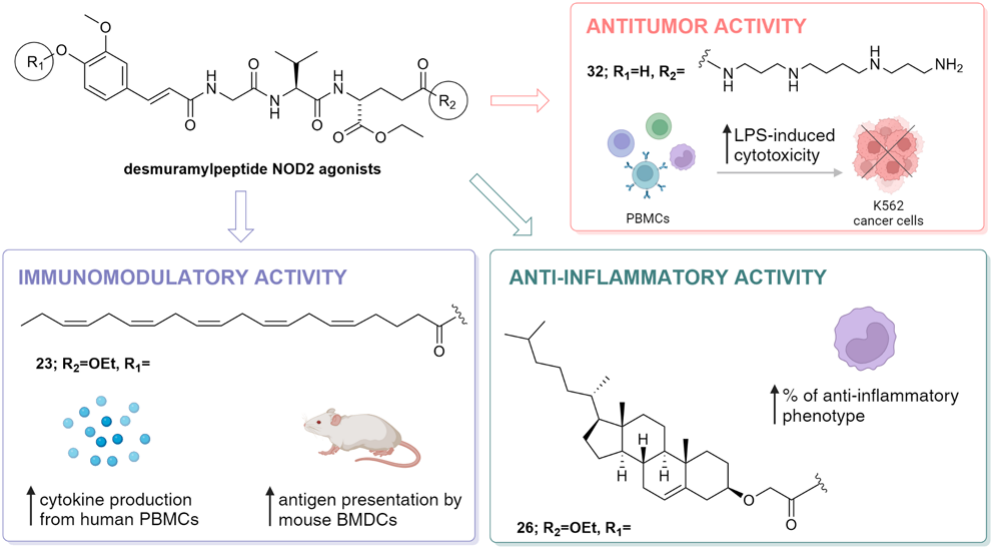

Herein we report on the design, synthesis and biological evaluation of a series of nucleotide-binding oligomerization-domain-containing protein 2 (NOD2) desmuramylpeptide agonists. The structural prerequisites that shape both physicochemical and immunomodulatory profiles of desmuramylpeptide NOD2 agonists have been delineated. Within this context, we identified **3**, a butyrylated desmuramylpeptide, as a potent *in vitro* NOD2 agonist (EC_50_ = 4.6 nM), exhibiting an almost 17-fold enhancement in potency compared to its unsubstituted counterpart **1** (EC_50_ = 77.0 nM). The novel set of desmuramylpeptides demonstrate unique *in vitro* immunomodulatory activities. They elicited cytokine production in peripheral blood mononuclear cells (PBMCs), both alone and in conjunction with lipopolysaccharide (LPS). The spermine-decorated **32** also stimulated the LPS-induced cytotoxic activity (2.95-fold) of PBMCs against K562 cancer cells. Notably, the cholesterol-conjugate **26** displayed anti-inflammatory actions, highlighted by its capacity to convert the inflammatory monocyte subset into an anti-inflammatory phenotype. Finally, the eicosapentaenoylated derivative **23** augmented antigen presentation by mouse bone marrow-derived dendritic cells (BMDCs), thus highlighting its potential as a vaccine adjuvant.

## 1 Introduction

Nucleotide-binding oligomerization-domain-containing protein 2 (NOD2) is an innate immune receptor located in the cytosol of leukocytes and intestinal epithelial cells, specifically evolved to detect and respond to fragments of bacterial peptidoglycan.^1^ When activated by its respective ligands, NOD2 triggers downstream signaling pathways, including the nuclear factor κB (NF-κB) and mitogen-activated protein kinase, leading to a proinflammatory response marked by the production of cytokines, type I interferons (IFNs), nitric oxide, and reactive oxygen species.^2^ The generated immune response not only provides immediate protection against invading microbes but also enhances antigen-specific adaptive immunity.^3,4^

The clinical significance of NOD2 agonists was initially demonstrated by the identification of muramyl dipeptide (MDP), the smallest peptidoglycan fragment capable of activating NOD2, as the adjuvant in Freund’s complete adjuvant.^5−7^ Besides promoting cytokine production, NOD2 agonists also induce the maturation and activation of dendritic cells (DCs) and initiate autophagy, all of which are desirable qualities in vaccine adjuvant development.^8−10^ Beyond conventional prophylactic vaccines, NOD2 agonists also play a crucial role in the mucosal and systemic responses of mucosal vaccines.^11,12^ Furthermore, their potential in cancer immunotherapy is highlighted by their ability to enhance the antitumor activity of immune cells.^13^ Importantly, NOD2 agonists have also been demonstrated to promote immune checkpoint inhibitor therapy.^14−16^ Engagement of NOD2 not only safeguards against microbial infections,^17^ but also, intriguingly, induces a shift in monocytes from the inflammatory to the patrolling subset.^18^ MDP has thus shown promise in mouse models of chronic inflammatory diseases, such as multiple sclerosis, Alzheimer’s disease, and colitis, where sustained NOD2 activation proved to be beneficial.^19−23^

Challenges posed by pyrogenicity, rapid elimination, and metabolic instability, hinder clinical utility of MDP.^24−27^ However, the potential for both enhanced efficacy and a more favorable safety profile exists through chemical modifications of the parent structure.^28^ In efforts to overcome these challenges and enhance its clinical applicability, extensive research has been conducted on structural modifications of MDP. Numerous reviews provide comprehensive insights into the structure–activity relationships (SAR) associated with these modifications.^28−31^ Particularly noteworthy are two lipophilic derivatives of MDP, namely romurtide^32^ and mifamurtide,^33^ which are presently employed in treating leukopenia and osteosarcoma, respectively. Additionally, the hydrophilic MDP derivatives, murabutide and nor-MDP, have undergone investigation in several clinical trials as potential vaccine adjuvants.^34,35^ Desmuramylpeptides are a class of compounds sans the MurNAc moiety and usually comprise the preserved or slightly varied MDP peptide portion attached to diverse lipophilic groups.^36^

Here, we describe the design, synthesis, and biological evaluation of a series of novel desmuramylpeptides that are based on the structure of **1**, a potent NOD2 agonist previously reported by our group that contains a *trans*-feruloyl-glycine MurNAc mimetic.^37^ After an initial screen of their NOD2 agonistic activities, we validated selected compounds in different *in vitro*/*ex vivo* models. The novel set of desmuramylpeptides demonstrate unique *in vitro*/*ex vivo* immunomodulatory activities thus providing a springboard toward novel adjuvants/immunomodulators and new leads for a variety of NOD2-responsive diseases.

## 2 Results and Discussion

### 2.1 Design

**Figure 1:**
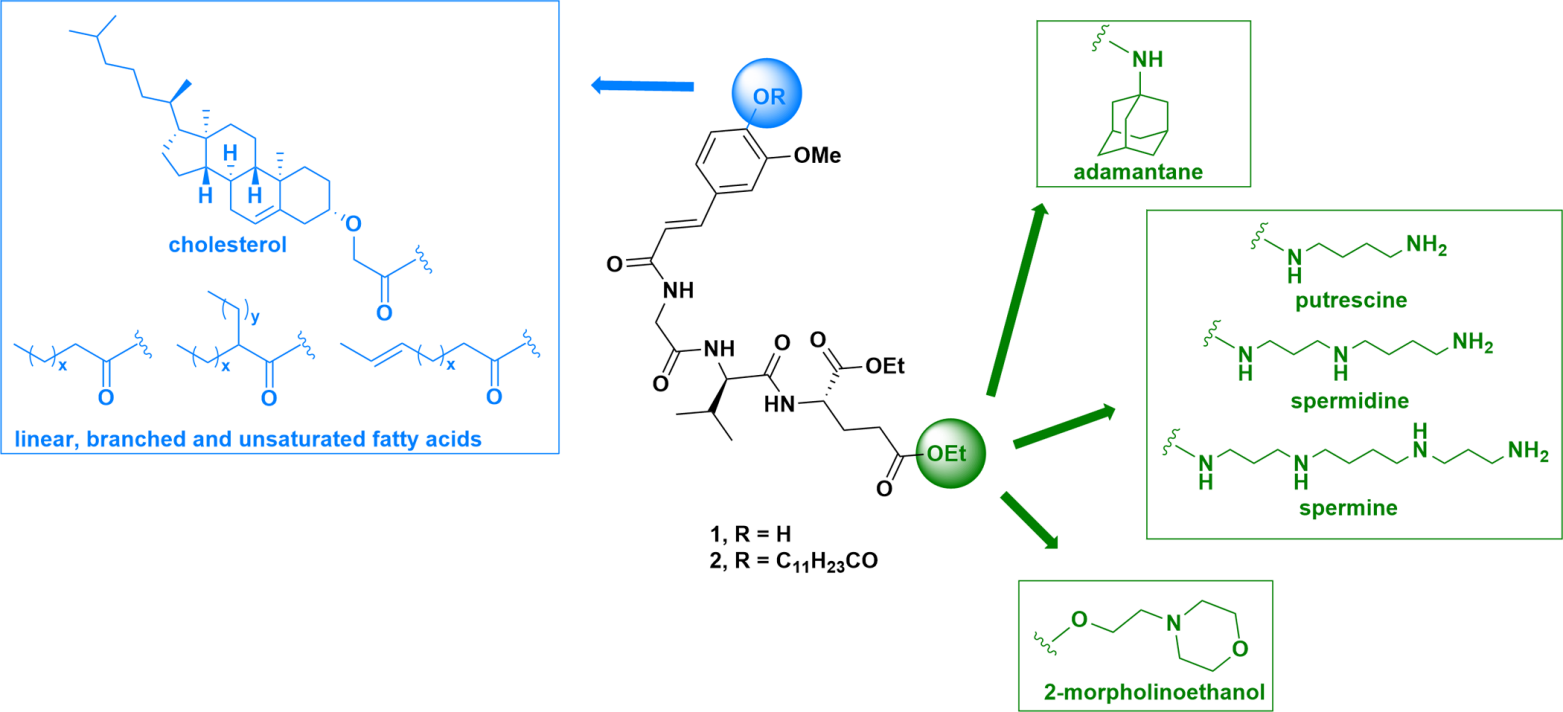
Design of novel NOD2 agonists based on **1** and **2**.

In our earlier work, we uncovered desmuramylpeptides **1**(37) and **2**(38) both based on the glycine-l-valine-d-glutamate tripeptide core and featuring a *trans*-feruloyl-glycine moiety in lieu of the *N*-acetylmuramic acid portion of MDP. These analogs displayed NOD2 stimulatory activity in the low nanomolar range yet differed in their physicochemical properties. **1** is water-soluble and does not incorporate well into liposomes, while **2** enables liposome encapsulation but suffers from poor aqueous solubility. Building upon this discovery, our efforts since focused on systematically exploring structural alterations that can further enhance their NOD2 agonistic activity (see Figure 1), optimize their physicochemical properties and tailor their immune signatures.

In this study, we undertake a systematic search for chemical modifications that could modulate the physicochemical traits of desmuramylpeptides, including lipophilicity and kinetic solubility, while retaining or even improving NOD2 agonism. Lipidation is a well-known approach to improve intracellular delivery and facilitate incorporation into delivery systems (e.g., liposomes and nanoparticles) by exploiting the concept of membrane targeting via lipophilic anchors.^39^ Notably, such approach can vastly affect the biological activity as well. For example, lipidated MDP analogs exhibited enhanced NOD2 activation on account of their membrane targeting capacity,^40^ while decoration of MDP with linear and branched fatty acids led to prominent adjuvant properties.^28,41,42^ Conjugation of peptides, including muramyl peptides, with unsaturated fatty acids,^43^ cholesterol,^44,45^ and adamantane^46^ improved their cellular uptake. On top of that, cholesterol-conjugation confers lymph node targeting abilities^47^ while adamantane as an auxiliary group improved adjuvant activity and *in vivo* stability of peptides.^48−51^ Endogenous polyamines, including putrescine, spermidine and spermine, are internalized via specific receptor-mediated endocytosis—this transport apparatus can be exploited to enhance the cellular uptake^52^ since the polyamine binding sites of these proteins can accommodate substantial modifications of the parent polyamine structure.^53^

In light of these considerations, we aimed to modulate the physicochemical properties of the parent molecule through three distinct approaches. First, based on previous findings that introducing lipophilic acyl groups to the carbohydrate and d-isoglutamine portions of MDP improved adjuvant and immunoprotective properties while decreasing pyrogenicity, we applied analogous transformations to **1**. This involved a direct acylation of the phenolic hydroxyl group on the *trans*-feruloyl moiety with linear/branched saturated fatty acids and (poly)unsaturated fatty acids while the cholesterol portion was installed via a short linker thus yielding a library of prodrug derivatives (**3**–**24** and **26**; refer to Table 1). Second, we expanded our investigation of SAR to include NOD2 agonists linked with adamantane or polyamines via the γ-carboxylic acid (**28, 30, 32, 34**; refer to Table 2). Third, our attention was aimed toward increasing the aqueous solubility by introducing specific structural features; previous studies namely suggested that morpholinoethyl esters impart aqueous solubility in certain settings while sinapinic acid is expected to outperform *trans*-ferulic acid in terms of solubility.^54^ To that end, the morpholinoethyl ester functionality was installed in lieu of the ethyl ester thus yielding **35**, while we also replaced the *trans*-ferulic acid with its closely related sinapinic acid to afford **37** (refer to Table 3).

**Table 1:**
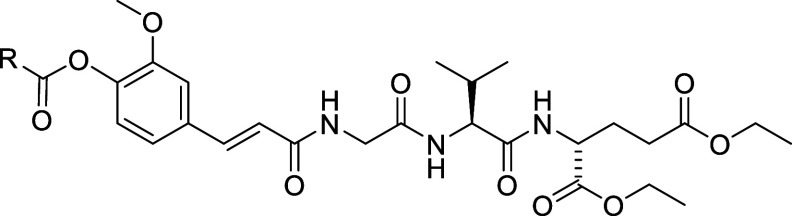

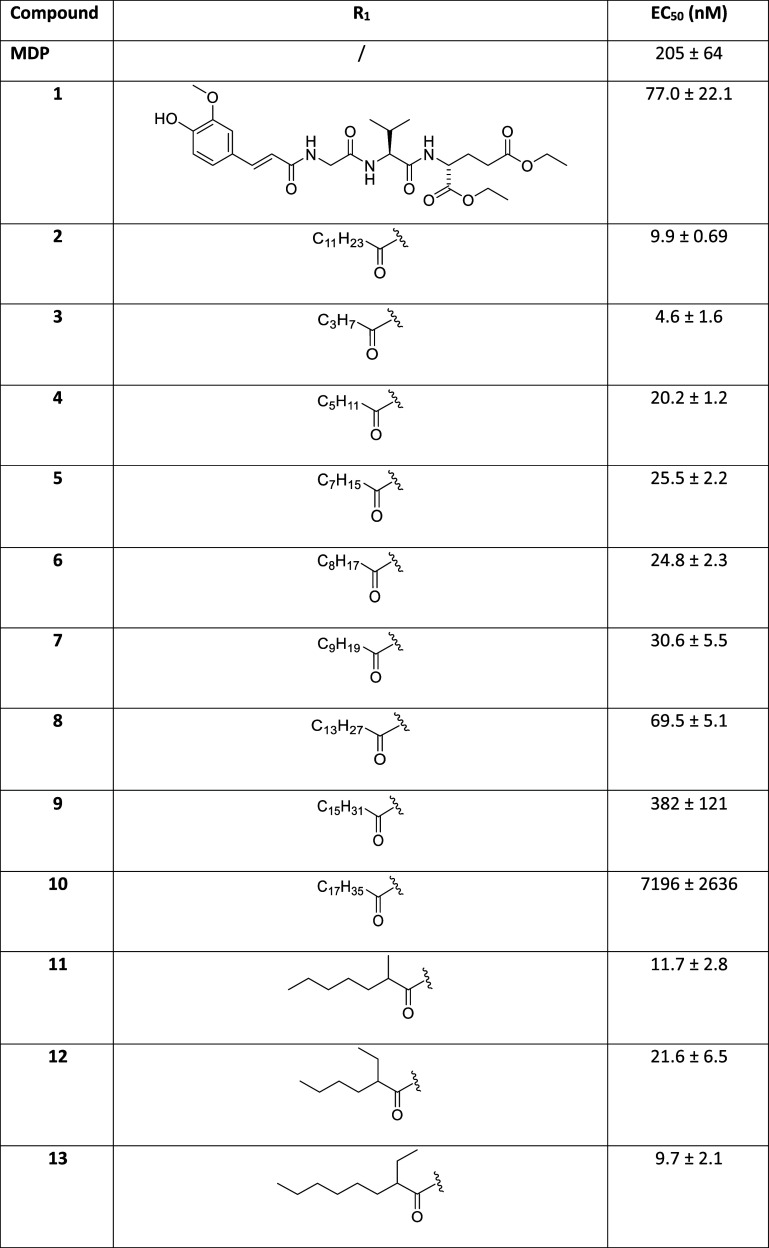

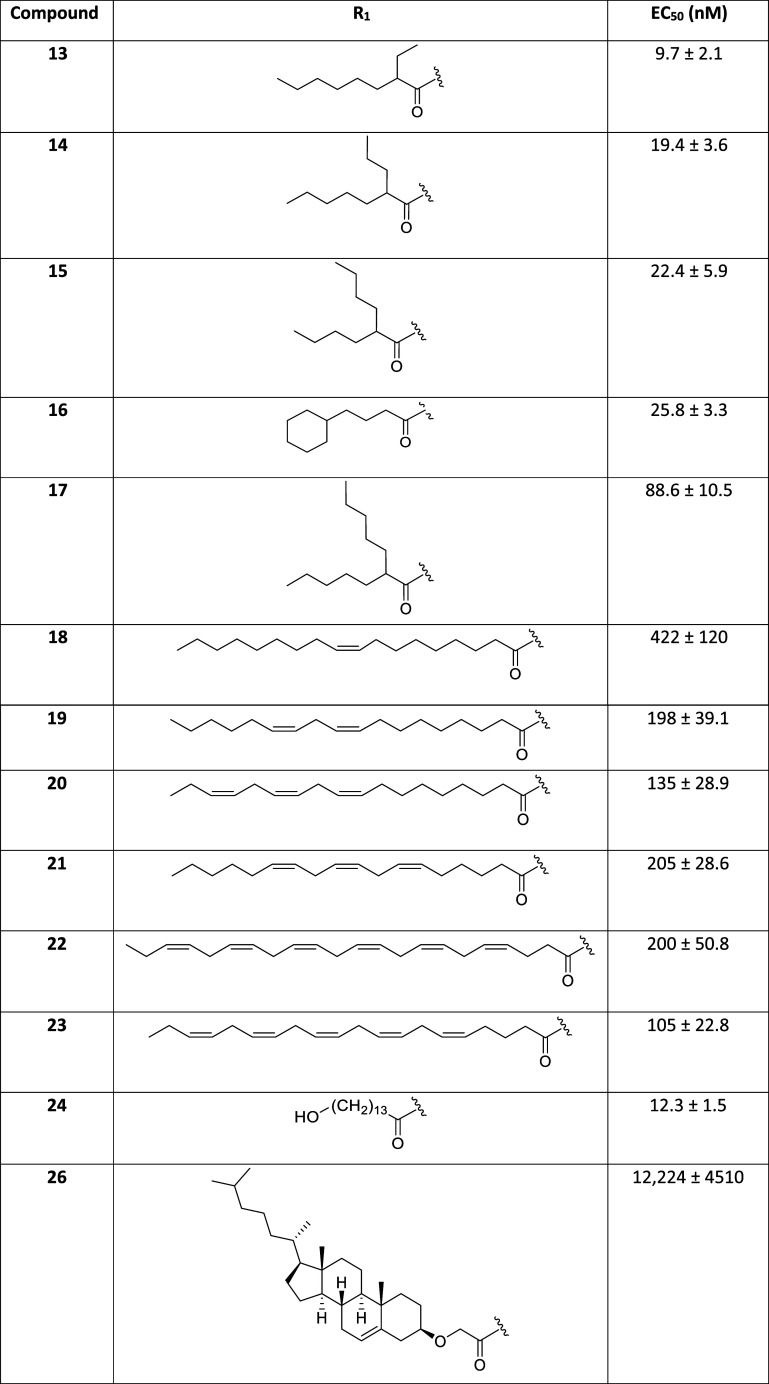
NOD2 Agonistic Activities of the Desmuramylpeptides with Modified *trans*-Feruloyl Moiety

**Table 2:**
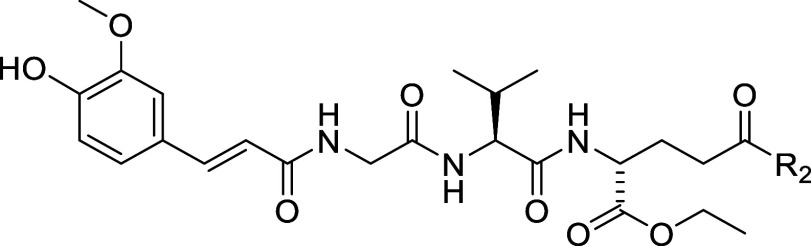

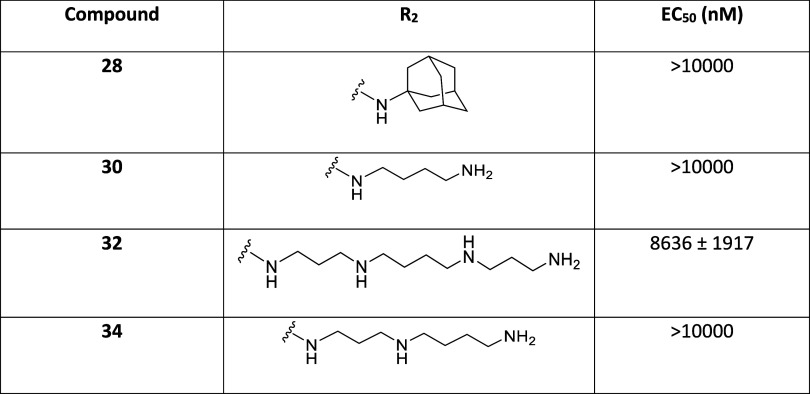
NOD2 Agonistic Activities of Desmuramylpeptides with Modified γ-Carboxylic Acid of the d-Glutamic Moiety

**Table 3:**
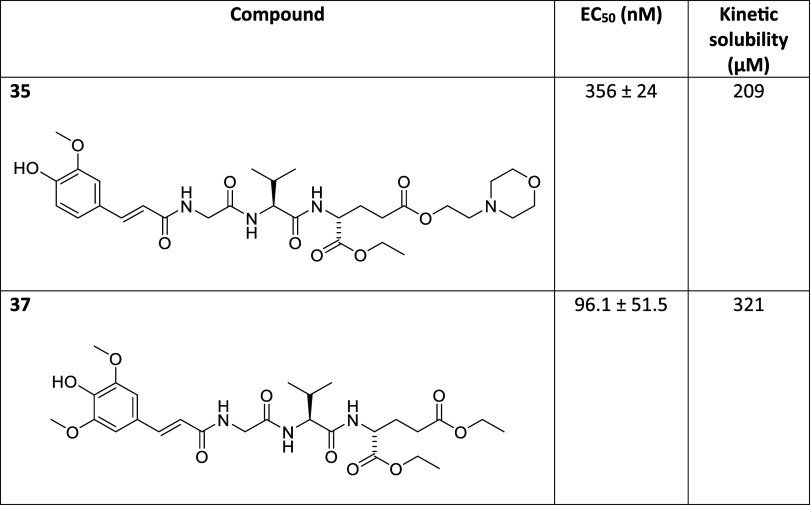
NOD2 Agonistic Activities and Kinetic Solubilities of Desmuramylpeptides with Specific Structural Features

### 2.2 Chemistry

**Scheme 1:**
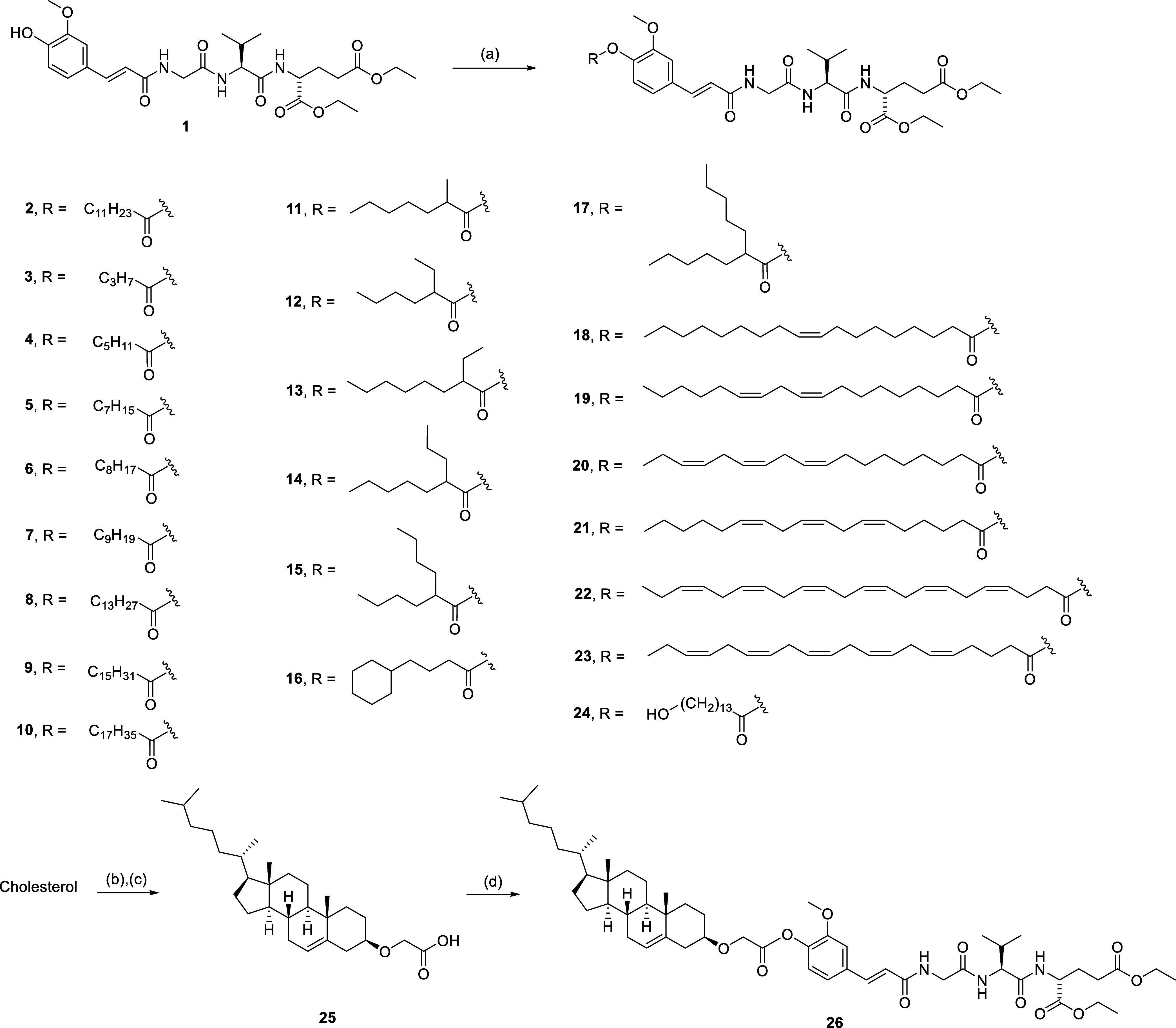
Synthesis of Compounds **3**–**24** and **26** Reagents and conditions: (a) RCOCl, Et_3_N, tetrahydrofuran (THF), rt. or RCOOH, COMU, *N*,*N*-diisopropylethylamine (DIPEA), *N*,*N*-dimethylformamide (DMF), rt; (b) BF_3_OEt_2_, ethyl diazoacetate, dichloromethane (DCM), rt; (c) NaOH, EtOH, rt; (d) **1**, COMU, DIPEA, DMF, rt.

The synthesis of compounds with linear/branched saturated fatty acids and (poly)unsaturated fatty acids attached to the hydroxyl group of the *trans*-feruloyl moiety is shown in Scheme 1. The NOD2 agonist **1** was synthesized as previously described by our group.^37^**1** was either acylated with fatty acyl chlorides in the presence of triethylamine or coupled with fatty acids employing (1-cyano-2-ethoxy-2-oxoethylidenaminooxy)-dimethylamino-morpholino-carbenium hexafluorophosphate (COMU) as the coupling reagent to produce its fatty acyl ester derivatives **3**–**24** (Scheme 1). **2** and **10** were synthesized as previously described.^38^**26** that features a cholesterol moiety was prepared by reacting ethyl diazoacetate with cholesterol in the presence of BF_3_OEt_2_ to give the ethyl ester, which was converted to free acid **25** on alkaline hydrolysis with aqueous 2 M NaOH in EtOH (Scheme 1). Subsequently, **25** was coupled to **1** under the COMU coupling conditions to afford the desired cholesterol-conjugated desmuramylpeptide **26**.

**Scheme 2:**
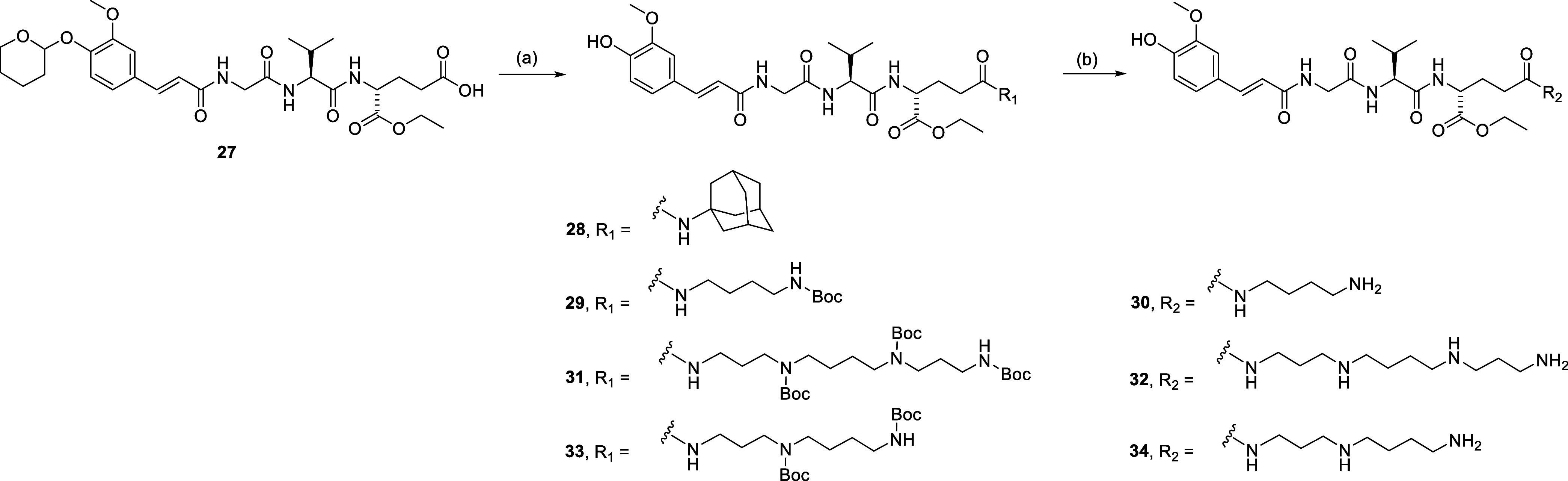
Synthesis of Compounds **28**, **30**, **32**, and **34** Reagents and conditions: (a) R_1_-NH_2_, DIPEA, COMU, DMF, rt; (b) TFA/DCM (1:5), rt.

Assembly of the desmuramypeptides carrying modifications of the d-glutamic acid moiety is shown in Scheme 2. First, the key intermediate **27**, a fully protected derivative of **1** featuring a free γ-carboxylic acid, was prepared as previously described.^55^ It was coupled to 1-adamantylamine using COMU to give the amide **28**. Similarly, COMU-mediated coupling of **27** to Boc-putrescine, tri-Boc spermine and bis-Boc spermidine produced compounds **29**, **31**, and **33**, respectively, which were deprotected with trifluoroacetic acid (TFA) to afford the polyamine-desmuramylpeptide conjugates **30**, **32**, and **34** (the tetrahydropyranyl group underwent removal during workup under acidic conditions).

**Scheme 3:**
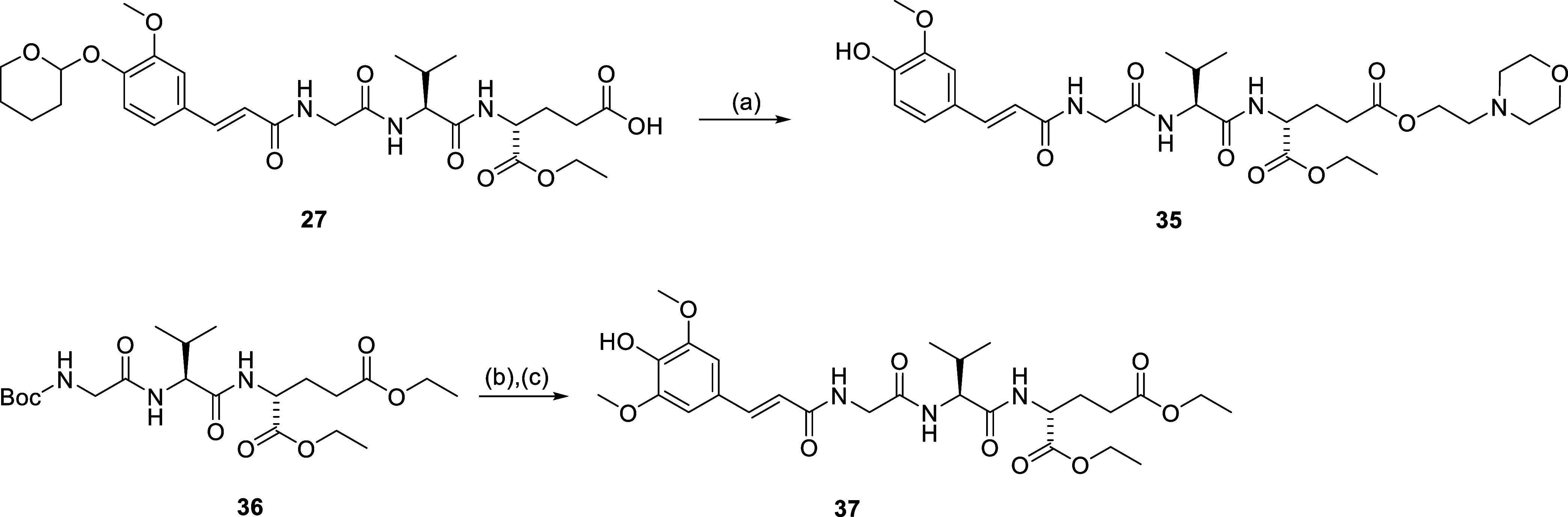
Synthesis of Compound **35** and **37** Reagents and conditions: (a) 4-(2-hydroxyethyl)morpholine, COMU, DIPEA, DMF, rt; (b) TFA/DCM (1:5), rt; (c) sinapinic acid, DIPEA, EDC, HOBt, DMF, rt.

Finally, Scheme 3 depicts the synthesis of **35** featuring a morpholinoethyl ester functionality in lieu of the ethyl ester. Briefly, 4-(2-hydroxyethyl)morpholine was coupled to **27** using COMU to afford the ester **35**. In addition, compound **37** was assembled by TFA-mediated deprotection of the Boc-protected tripeptide **36**(38) followed by coupling with sinapinic acid using 1-ethyl-3-(3-(dimethylamino)propyl)carbodiimide (EDC) and HOBt to produce the sinapinoyl derivative **37**.

### 2.3 Biological Studies

#### 2.3.1 NOD2 Agonistic Activity of Synthesized Desmuramylpeptides

To assess the NOD2 agonistic potential of the synthesized desmuramylpeptides at a cellular level, we employed the HEK-Blue NOD2 cell line reporter assay, a validated and commercially available method. All synthesized compounds were evaluated for their dose-dependent activities to establish their EC_50_ values. HEK-Blue NOD2 cells were initially exposed to MDP, **1**, and the synthesized desmuramylpeptides at various concentrations spanning from 0.5 nM to 4 μM (or 10 μM) over an 18 h period. The NF-κB transcriptional activity observed was normalized to that of the control HEK-Blue NOD2 cells treated with the vehicle (0.1% DMSO). Notably, none of the tested compounds displayed cytotoxic effects on the HEK-Blue NOD2 cells at 10 μM, as confirmed by the (3-(4,5-dimethylthiazol-2-yl)-5-(3-carboxymethoxyphenyl)-2-(4-sulfophenyl)-2*H*-tetrazolium) (MTS) cell viability assay (see Supporting Information (SI), Figure S1).

The phenolic hydroxy group of **1** served as a useful attachment point for the introduction of various acyl groups through esterification. Exploration around the chemical space of the *trans*-feruloyl moiety of **1** (EC_50_ = 77.0 nM) produced compounds **3**–**24** and **26** (Table 1). **3**, with an EC_50_ of 4.6 nM exhibits an almost 17-fold improvement over **1** and is the most potent agonist in this series. In general, the introduction of butyryl (**3**; EC_50_ = 4.6 nM), hexanoyl (**4**; EC_50_ = 20.2 nM), octanoyl (**5**; EC_50_ = 25.5 nM), nonanoyl (**6**; EC_50_ = 24.8 nM), decanoyl (**7**; EC_50_ = 30.6 nM), dodecanoyl (**2**; EC_50_ = 9.9 nM), and myristoyl (**8**; EC_50_ = 69.5 nM) groups resulted in improved activity over **1**, with activity slightly declining once the aliphatic chain length exceeded C12. Further increase in lipophilicity with longer alkyl chains including palmitoyl (**9**; EC_50_ = 382 nM) and stearoyl (**10**; EC_50_ = 7.2 μM), however, resulted in a more significant drop in NOD2 activity in spite of stearoyl group being a structural motif that has been used repeatedly in the preparation of potent MDP derivatives.^56−58^

The observed differences in *in vitro* NOD2 activation are likely attributed to more facile cleavage of the short-chain and midchain fatty acyl esters, compared to the longer esters (e.g., stearoyl), by the intracellular enzymes in the HEK293 cell line used. Conversely, these effects could prove advantageous *in vivo*, potentially boosting the efficacy of such derivatives, since acyl chain length is a critical factor determining micellar partitioning, a major determinant of fatty acid uptake.^59^ In addition, while enhanced lipophilicity has been associated with augmented membrane permeability, it also reinforces the compound-membrane interactions. Consequently, the retention of lipophilic derivatives within the membrane could, at least partially, clarify their diminished efficacy observed in cell assays, potentially due to their limited availability for binding to intracellular NOD2 receptors. The incorporation of NOD2 agonists of both muramylpeptide and desmuramylpeptide structural classes into nanoparticles has been shown to augment their NOD2 agonist activities, likely owing to enhanced cell internalization.^60−63^ Our preliminary experiments with **2** incorporated into polylactic acid-based nanoparticles demonstrated an increase in NOD2 agonist activity *in vitro*. Lipid-based nanoparticles, including solid lipid nanoparticles (SLN) and nanostructured lipid carriers (NLC), are widely investigated nanodelivery systems that have already been utilized in the setting of preventative and therapeutic vaccines.^64−66^ In this study, we wanted to ascertain whether incorporation of **2** into such lipid-based nanoparticles also translates to increased NOD2 agonist activity. To that end, SLN and NLC formulations of **2** (see Table S4) were prepared by melt-emulsification method. The NOD2 agonist activities of free **2**, **2**-loaded SLNs and **2**-loaded NLCs were then investigated employing the HEK-Blue NOD2 cell line. Expectedly, the incorporation of **2** boosted its NOD2 agonist activity by a factor of 5, with **2**-loaded SLNs and **2**-loaded NLCs having EC_50_ values of 2.1 and 3.4 nM, respectively (EC_50_ of free **2** was 10.5 nM). On the other hand, the **2**-free SLN and NLC were devoid of any activity. Given that HEK cells do not display significant phagocytic activity, we speculated that an increase in NOD2 agonistic activity was indeed a result of improved cell internalization.

Compounds from the C8-acylated series, namely the linear **5** (octanoyl; EC_50_ = 25.5 nM), as well as branched **11** (2-methylheptanoyl; EC_50_ = 11.7 nM) and **12** (2-ethylhexanoyl; EC_50_ = 21.6), display similar NOD2 agonistic capacities, with EC_50_ values in the low nanomolar range. Similarly, the C10-acyl moiety featuring series showed no discernible branching-dependent pattern; the potency of derivatives carrying the same number of carbon atoms in the acyl chain differed only slightly with increasing branching of fatty acids as demonstrated by **7** (decanoyl; EC_50_ = 30.6 nM), **13** (2-ethyloctanoyl; EC_50_ = 9.7 nM), **14** (2-propylheptanoyl; EC_50_ = 19.4 nM), **15** (2-butylhexanoyl; EC_50_ = 22.4 nM) and **16** (4-cyclohexylbutanoyl; EC_50_ = 25.8 nM). In fact, a pronounced effect of branching on the NOD2 activating capacity of desmuramylpeptides was only seen with **17** (EC_50_ = 88.6 nM), a symmetrically branched derivative of **2** (lauroyl; EC_50_ = 9.9 nM), which exhibited a drop of activity for a factor of 9.

Interestingly, the introduction of a double bond into the C18 chain brought about a 17-fold increase in potency as seen with the oleoyl derivative (**18**; EC_50_ = 422 nM) compared to its saturated counterpart compound **10** (EC_50_ = 7.2 μM). An additional ethylene functionality featured in **19**, which carries a linoleoyl portion, further enhanced the NOD2 agonistic activity (EC_50_ = 198 nM). The α-linolenoyl derivative **20** that incorporates three double bonds was slightly more potent, with an EC_50_ of 135 nM, while such improvement in potency was not seen with its positional isomer, the γ-linolenoyl derivative **21** (EC_50_ = 205 nM). Docosahexaenoic acid and eicosapentaenoic acid (EPA), prominent representatives of the poly unsaturated fatty acids (PUFA), were installed into **22** (EC_50_ = 200 nM) and **23** (EC_50_ = 105 nM), respectively. Clearly, the eicosapentaenoylated derivative **23** displayed the greatest potency of the (poly)unsaturated fatty acyl derivatives of **1**. Park et al. have shown that the degree of saturation of fatty acids exerts a profound impact on permeability of fatty acylated peptides—a higher degree of unsaturation results in higher permeability.^43^ PUFA can also induce significant biophysical perturbations upon incorporation into membrane phospholipids.^67,68^ Installment of another polar functionality, a hydroxyl group, into the terminus of the myristoyl side chain (**24**; EC_50_ = 12.3 nM) boosted the NOD2 agonistic activity 6-fold compared to the unsubstituted myristoylated derivative **8** (EC_50_ = 69.5 nM). Given that the hydrophobicity of fatty acyl residues in general reduces as the number of unsaturated sites increases,^69,70^ we decided to ascertain how such structural modifications translate to the kinetic solubility of the molecule as a whole. To that end, the kinetic solubilities of selected acylated analogs of **1** (kinetic solubility −417 μM), namely **2**, **3**, **9**, and **23**, were measured using the method described by Hoelke et al.^71^ Of the saturated fatty acyl analogs, the long-chain (C16) analog **9** and the midchain (C12) analog **2** proved to be insoluble, while the short-chain (C4) analog **3** displayed a solubility of 178 μM. Decoration of **1** by the long-chain polyunsaturated fatty acid EPA diminished the solubility of the parent compound by a factor of 4, however, compound **23** still displayed a solubility of 105 μM. The acylation with PUFA thus provides an elegant, albeit counterintuitive, approach, by which permeability of the molecule as a whole as well as its capacity for liposomal encapsulation can be enhanced without completely abolishing its aqueous solubility.

To expand the concept of lipophilic anchors to other entities than fatty acids, **1** was conjugated to cholesterol via a methylenecarbonyl bridge to afford **26** (EC_50_ = 12.2 μM). While it was not as active in the HEK Blue reporter assay, it is expected to exert pronounced NOD2-related biological activities in functional assays as was the case with MDP-cholesterol conjugates reported by Merhi et al.^44^

Exploration of the chemical space around the γ-carboxylic acid of the d-Glu moiety produced desmuramylpeptides **28**, **30**, **32**, and **34** (Table 2). Adamantane- and polyamine-functionalized desmuramylpeptides were evaluated as potential NOD2 agonists. Interestingly, the adamantane-conjugated desmuramylpeptide **28** (EC_50_ > 10 μM) was devoid of NOD2 agonistic activity. Nonetheless, dipeptides carrying an adamantylamine portion attached to the d-glutamic acid were previously shown to display *in vivo* adjuvant activities;^49,50^ such a substitution has yet to be evaluated in the context of desmuramylpeptides. Further, the NOD2 agonistic activities of polyamine-decorated analogs **30** (putrescine, EC_50_ > 10 μM), **32** (spermine, EC_50_ = 8.6 μM) and **34** (spermidine, EC_50_ > 10 μM) were also abrogated. It is well-known that HEK293 cells have low hydrolytic activity due to low expression of carboxylesterases, the enzymes executing the hydrolysis of esters and amides.^72,73^ These data are in agreement with the previously reported impaired activities of amidated muramylpeptide derivatives.^74,75^ Similarly, a direct replacement of both ethyl ester groups in a desmuramylpeptide NOD2 agonist with their ethyl amide counterparts resulted in a substantially diminished activity,^38^ suggesting that while the polyamine moieties attached to the desmuramylpeptides via an amide linkage could assist in compound internalization, they do not contribute to the NOD2 binding.

In our last group of modifications (see Table 3), we probed how introduction of specific structural features suggested to impart aqueous solubility, affected the activity and kinetic solubility of **1**. As the cellular assays are defined by both the crossing of the membrane by the compounds and their activation of NOD2, the potential hydrophilicity of these compounds could negatively impact their cellular NOD2 activities. The 3,5-dimethoxy-4-hydroxy substitution pattern of the aromatic ring in **37** (EC_50_ = 96.1 nM) was evidently comparable to the 4-hydroxy-3-methoxy pattern of the *trans*-feruloyl moiety featured in lead compound (**1**; EC_50_ = 77.0 nM) in terms of activity. On the other hand, a replacement of the ethyl ester in **1** with the morpholinoethyl portion (**35**; EC_50_ = 356 nM) brought about a significant drop in activity by a factor of 4. Diminished susceptibility of such modified ester to both enzymatic and spontaneous hydrolytic processes might explain less pronounced effects of **35** in the HEK-Blue NOD2 cell assays. Surprisingly, both prepared compounds proved to be inferior to **1** with regard to kinetic solubility (**1**—417 μM, **35**—209 μM and **37**—321 μM) in spite of the morpholinoethyl group being a structural motif previously reported to improve aqueous solubility.^54^

We note here that the readouts from the HEK-Blue cell assay system do not accurately recapitulate the behaviors of these synthesized desmuramylpeptides in primary cells. For this purpose, further biological evaluations were carried out for the most prominent NOD2 agonists representing different structural classes, **3**, **23**, **26**, **28**, **32**, as well as **2** and MDP as the internal and external positive controls, respectively.

#### 2.3.2 Immunomodulatory Effect of Desmuramylpeptides on Peripheral Blood Mononuclear Cells (PBMCs)

The immunomodulatory effects of the selected desmuramylpeptides were examined in human primary peripheral blood mononuclear cells (PBMCs). This diverse mixture of immune cells provided a platform to investigate the effects of these NOD2 agonists within a physiologically relevant environment, encompassing other NOD2-interacting and downstream signaling proteins. Initially, the effects of desmuramylpeptides on the secreted cytokine profile were determined using the LEGENDplex Human Essential Immune Response Panel (IL-4, IL-2, IP-10, IL-1β, TNF-α, MCP-1, IL-17A, IL-6, IL-10, IFN-γ, IL-12p70, IL-8, TGF-β1). Given that NOD2 activation often modulates the Toll-like receptor (TLR)-induced cytokine responses, we investigated the impact of combining desmuramylpeptides with lipopolysaccharide (LPS), a well-known TLR4 agonist, on cytokine secretion.^76−78^

**Figure 2:**
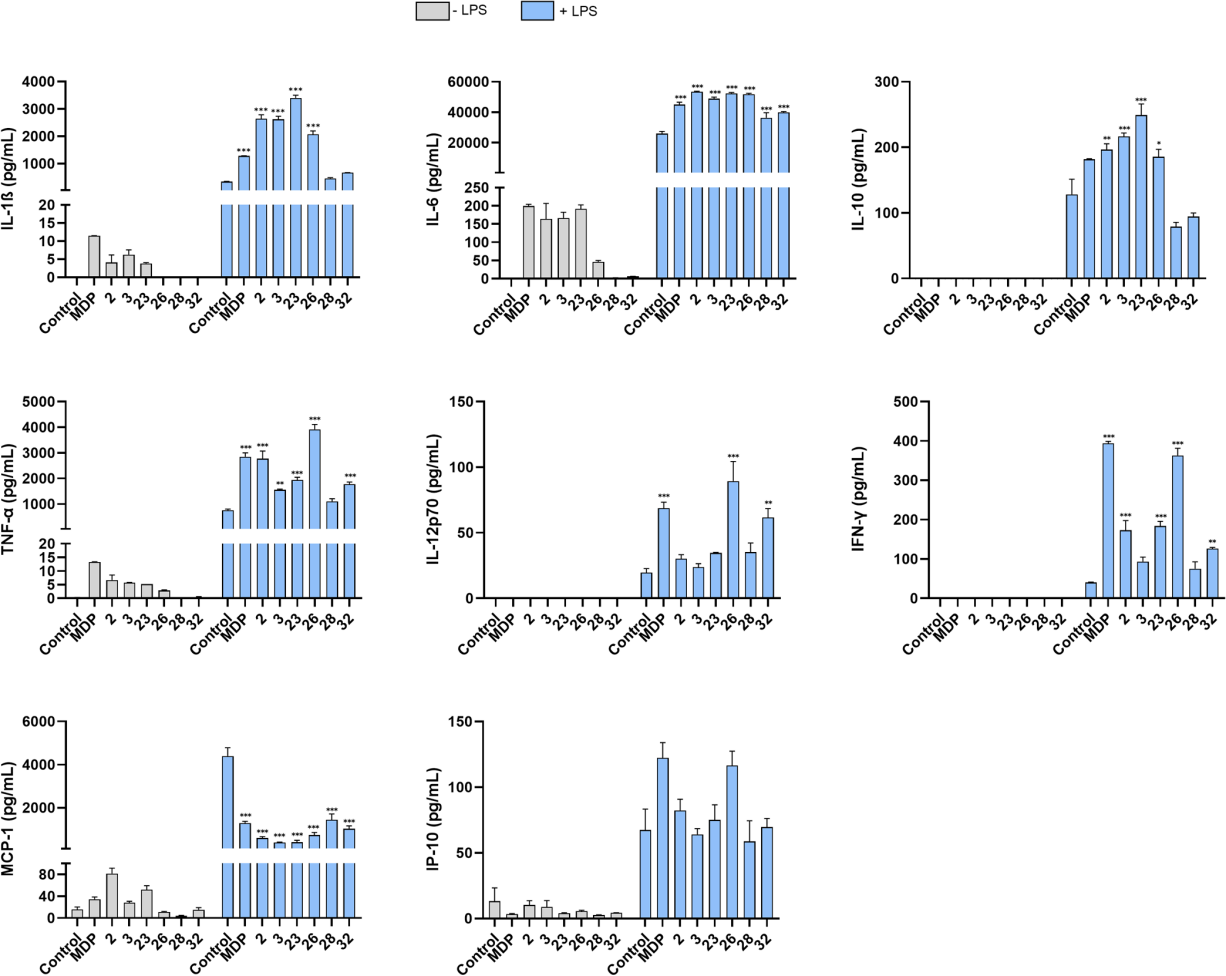
Effects of MDP and desmuramylpeptide treatments on the release of cytokines from human PBMCs. Cytokine concentrations were measured after 18 h stimulation with MDP (2 μM) or the desmuramylpeptides (2 μM) in the absence or presence of LPS (10 ng/mL). Data are expressed as mean ± standard error of the mean (SEM) of 2 (− LPS) or 3 (+ LPS) independent experiments. *p ≤ 0.05, **p ≤ 0.01, ***p ≤ 0.001 versus LPS-treated PBMCs.

Figure 2 shows the effects of the desmuramylpeptides (2 μM) on induction of various cytokines, both alone and in combination with LPS (10 ng/mL). In line with previous studies,^79^ overnight stimulation of PBMCs with MDP and desmuramylpeptides alone led to minor increases in production of proinflammatory cytokines and chemokines, including IL-6, IL-8, and MCP-1. Compounds **28** and **32** in particular, but also **26**, induced smaller increases in the levels of these cytokines, compared to those of MDP, **3**, **2**, and **23**. The elicited cytokine profiles thus broadly reflect the structure–activity relationships obtained in the reporter gene assays.

Stimulation with LPS, however, elicited substantial increases in overall cytokine production, which were further enhanced by MDP and the desmuramylpeptides. The IL-8 produced appeared to have plateaued following the concurrent stimulation with LPS and the NOD2 agonists, suggesting saturation of IL-8 production (data shown in SI). However, compared to LPS stimulation alone, all of the tested compounds markedly boosted the levels of IL-1β, IL-6, TNF-α, and IFN-γ in a synergistic manner, which is in line with the previously described synergistic signal amplification between NOD2 and TLR4.^80,81^ In sharp contrast, simultaneous treatment with LPS and each tested NOD2 agonist brought about a decrease in the levels of MCP-1 compared to the effect of LPS alone. The levels of LPS-induced IL-10 were amplified by MDP, **2**, **3**, **23**, and **26**, while the opposite occurred following cotreatment of PBMCs with **28** and **32**. The immune signatures obtained following simultaneous NOD2/TLR4 engagement can be classified into three distinct groups. The first group entails fatty acyl analogs **2**, **3**, and **23**, characterized by increased secretion of IL-1β, IL-10, and TGFβ and significantly diminished production of MCP-1 and IFN-γ, all compared to the levels induced by the MDP/LPS combination. Of note, the observed effects were most pronounced for **23**. The second group includes the γ-carboxylate derivatized amide analogs **28** and **32**, whose impact on LPS-induced cytokine and chemokine secretion are not as prominent. While the levels of secreted MCP-1 were on par with that induced by the MDP/LPS combination, they elicited lesser amounts of IL-1β, IL-6, IL-10, TNF-α, and IFN-γ and thus proved as inferior to MDP. The last group is represented by **26**, which is comparable to MDP in terms of the capacity to induce IP-10 and IFN-γ secretion but outperforms it by eliciting higher production of IL-12p70 and TNF-α.

#### 2.3.3 The Effect of Desmuramylpeptides on PBMC-Induced Cytotoxicity

In this study, we investigated the potential of desmuramylpeptides, both alone and in combination with LPS, to enhance the cytotoxic activity of PBMCs against cancer cells employing a flow-cytometry-based cytotoxicity assay based on coincubation of preactivated PBMCs with fluorescently labeled K562 cancer cells.^82^ Among the diverse subpopulations within PBMCs, natural killer (NK) cells play a pivotal role in detecting and eliminating cancer cells. NOD2-mediated NK cell activity against cancer cells arises from both direct NOD2 agonist elicited cytolytic activity of NK cells^83^ and indirect activation through cytokine release by other NOD2-responsive cells, such as monocytes, which comprise approximately 10% of PBMCs; such interactions potentially reinforce the immune response.^84^ Monocytes *per se* also demonstrate nonspecific cytolytic activity against cancer cells, which is further potentiated following MDP stimulation.^85,86^ To that end, we utilized the entire PBMC population as effector cells rather than isolated NK cells, to mimic conditions more closely resembling the *in vivo* environment while K562 chronic myelogenous leukemia cells were used as the target cells in a 40:1 effector to target cell ratio.^87^

**Figure 3:**
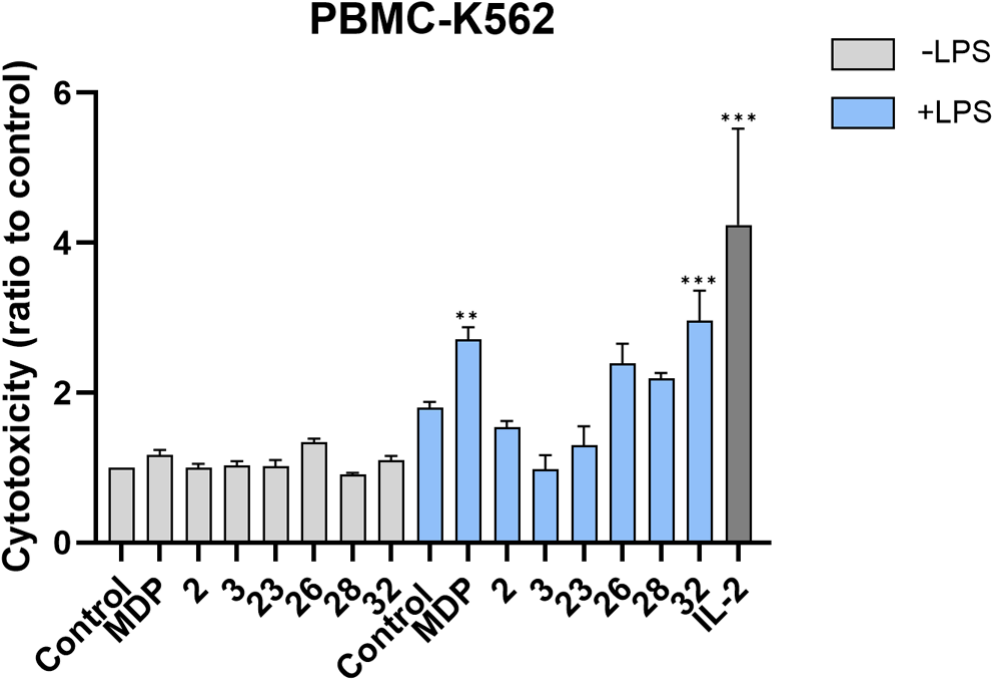
Effects of MDP and desmuramylpeptides on the cytotoxic activities of PBMCs against K562 cells. PBMCs were treated for 18 h with MDP (10 μM), desmuramylpeptides (10 μM), in the presence or absence of LPS (1 μg/mL), IL-2 (200 U/mL), or the corresponding vehicle (0.1% DMSO), before the addition of the K562 cells. Cytotoxicity was determined after 4 h coincubation. Each experiment was conducted in duplicate and repeated in three independent biological replicates. Data are shown as relative activities to the negative control (NT, 0.1% DMSO) and are means ± SEM of three independent experiments. **, *p* < 0.01, ***, *p* < 0.001 versus relevant negative controls.

As illustrated by Figure 3, among the compounds tested, only the cholesterol-conjugated **26** (1.34-fold), alongside LPS (1.80-fold), and IL-2 (4.24-fold) as the positive controls,^88^ exhibited a notable enhancement in the cytotoxicity of PBMCs against K562 cancer cells at a concentration of 10 μM, whereas other desmuramylpeptides and MDP showed no activity by themselves. The **26**-induced cytotoxicity, albeit not statistically significant, lends weight to the previously identified *in vitro* and *in vivo* antitumor activity of the MDP-l-Ala-cholesterol derivative.^44,89,90^ In general, however, the results present a notable disparity from the observations in the HEK-Blue NOD2 cell line, where **26** exhibited only a fraction of the NOD2 agonistic activity compared to MDP and other desmuramylpeptides. Yet they are consistent with our recently reported observations where low nanomolar NOD2 agonists failed to induce the cytotoxic activity against K562 cells.^38^ Although previous studies suggested lipophilicity-dependent effects on immune cell stimulation that may contribute to PBMC cytotoxicity,^91^ compounds **2**, **3**, and **23** that also feature extended lipophilic tails, displayed no activity. Similarly, the amide derivatives **28** and **32** were also devoid of the capacity to induce cytotoxicity. Additionally, PBMCs and cancer cells alone were treated with desmuramylpeptides to assess any direct cytotoxic effects on these cells. No notable increase in cell death proportions was observed, confirming that the observed moderate activity of **26** can be attributed to the stimulation of PBMC cytotoxicity (Table S2).

Co-stimulation of PBMCs with combinations of LPS and desmuramylpeptides did not fully reflect the results obtained in the absence of LPS. On the one hand, combinations with **2** (1.54-fold), **3** (0.98-fold), and **23** (1.30-fold) even suppressed the PBMC cytotoxicity elicited by LPS alone (1.80-fold), while cotreatments with MDP (2.71-fold), **26** (2.39-fold), **28** (2.19-fold), and **32** (2.95-fold) boosted the LPS-induced cytotoxic activity of PBMCs, with all combinations displaying slightly lower activity to that of the positive control IL-2. Remarkably, and in contrast with the results obtained in the absence of costimuli, the spermine-featuring **32** exhibited considerably more pronounced activity compared to those of other desmuramylpeptides. Interestingly, spermine-linked anticancer agents have previously been shown to exert increased cytotoxicity against cancer cells (antiproliferative activity on a panel of tumor cell lines) compared to their unconjugated counterparts.^92,93^ Collectively, the distinctive cytokine profiles induced by the tested desmuramylpeptides in PBMCs (Figure 2), along with the substantial differences in their cytotoxicity activation (Figure 3) suggest a nonlinear relationship between lipophilicity and immunostimulatory effects across various PBMC cell subpopulations that seems to rely more on the presence of specific structural features.

#### 2.3.4 The Effect of Desmuramylpeptides on Monocyte Phenotype Modulation

**Figure 4:**
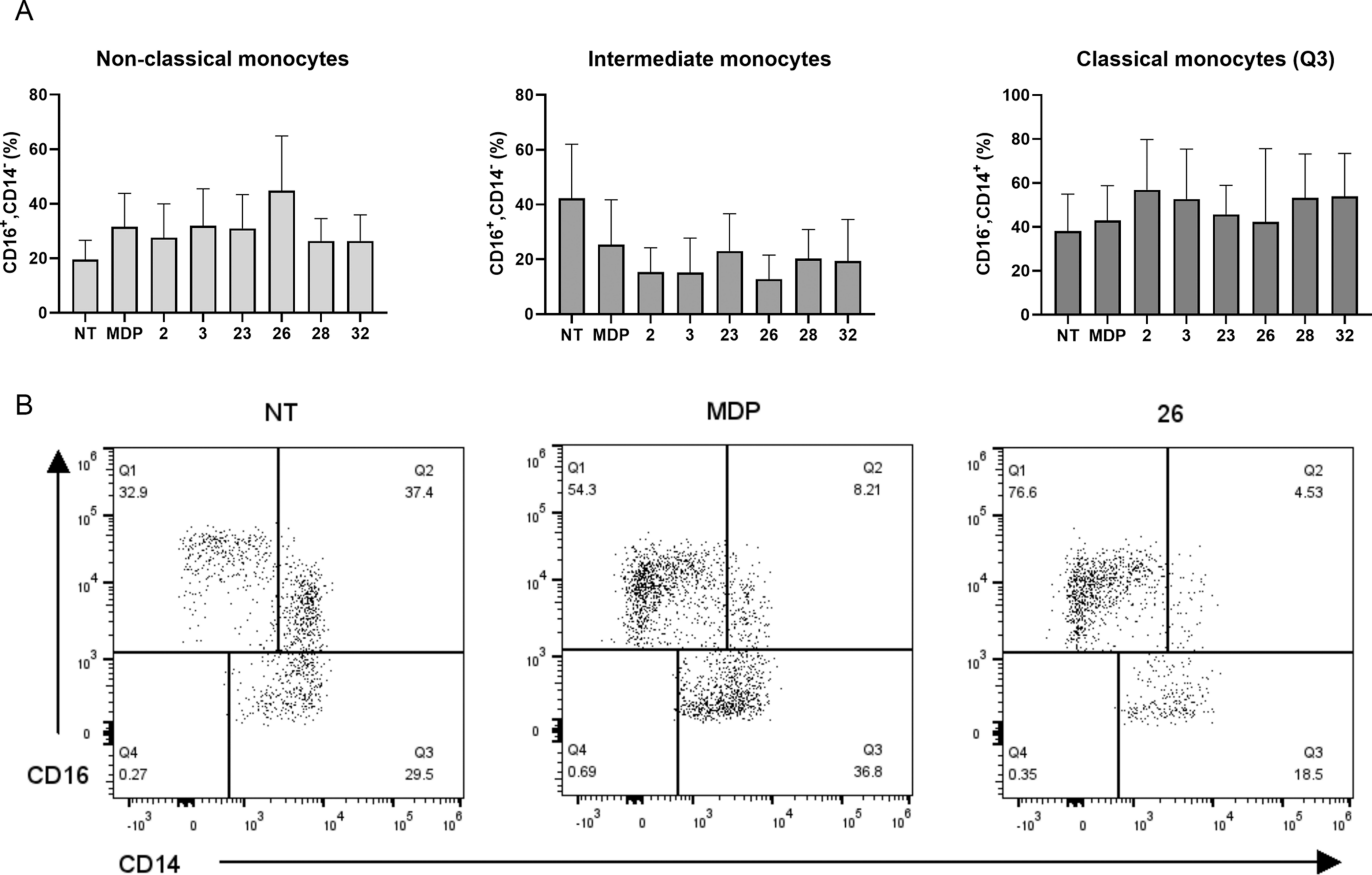
Effects of MDP and desmuramylpeptides on the conversion of monocytes. PBMCs were treated for 18 h with MDP (10 μM) or desmuramylpeptides (10 μM). (A) Frequencies (%) of blood monocyte subsets. Data are shown as relative activities to the negative control (NT, 0.1% DMSO) and are means ± SEM of three independent experiments. (B) Representative flow cytometry dot plots of three monocyte subsets (Q1; nonclassical monocytes, Q2; intermediate monocytes, Q3 classical monocytes) for NT, MDP and **26**.

Engagement of NOD2 has been reported to increase the levels of an anti-inflammatory monocyte subset, denoted as patrolling monocytes (CD14^–^, CD16^+2^), thus exerting a counterintuitive role in inflammatory processes, while concomitantly increasing the levels of total blood monocytes (released from bone marrow).^18^ The patrolling monocytes have been suggested to differentiate into M2-like macrophages following their recruitment in the inflamed tissue.^18^ This process involves a transition from the inflammatory monocytes, typified by CD14^+^CD16^–^, through an intermediate subset of CD14^+^CD16^+^ monocytes. Freshly isolated monocytes predominantly consist of over 80% classical monocytes, with intermediate monocytes comprising 1–3%, and nonclassical monocytes making up 5–7%, while the process of *in vitro* culturing significantly increases the percentage of the intermediate phenotype (reaching up to 80%).^18^ To recapitulate this phenomenon, we modified the reported procedure and employed PBMCs in lieu of isolated monocytes. Identification of human blood monocyte subsets was achieved through flow cytometry, utilizing the expression of CD14 and CD16 markers as distinguishing criteria. As illustrated in Figure 4, treatment of PBMCs with MDP brought about a boost in the levels of nonclassical monocytes from 19.5% (NT) to 31.5%. **3** and **23** achieved a comparable conversion rate to the nonclassical monocyte subset, namely 31.9 to 31.0%, respectively. On the other hand, **2**, **28**, and **32** exhibited slightly lower conversion rates ranging from 26.3 to 27.6%. Similar to what has been reported (e.g., inactivity of murabutide),^18^ also in our case not all NOD2 agonists were equally endowed with the ability to generate patrolling monocytes. Strikingly, the cholesterol-conjugated analog **26** increased the levels of nonclassical monocytes to 44.8%. Our findings suggest that the most prominent contribution to the increased fraction of patrolling monocytes originated from the desmuramylpeptide-elicited conversion of the intermediate phenotype. Albeit not statistically significant due to high interindividual variability, the obtained results demonstrate that NOD2 activation elicits modulation of monocyte phenotype and function, thus highlighting its contribution in monocyte plasticity, and shedding some light on the structural features required for conversion of monocyte phenotype.

#### 2.3.5 *Ex Vivo* Adjuvant Properties of Desmuramylpeptides in Murine BMDCs

DCs play a pivotal role in orchestrating the connection between the innate and adaptive branches of the immune system. They achieve this by processing and presenting antigens to naive T cells, thereby initiating antigen-specific T-cell and B-cell immune responses.^94^ There is clear evidence that NOD2 activation leads to DC maturation and enhanced antigen presentation *in vitro*, which translates into adjuvant activities *in vivo*.^8,95^ Depending on the stimuli encountered during DC activation, these cells produce different cytokines, which in turn drive the polarization of T helper cells (CD4^+^ T cells) toward distinct effector functions. Notably, among these functions, the Th2 and Th1 subtypes are known to respectively promote antigen-specific humoral and cellular immunity. Cellular immune responses are further characterized by the induction of cytotoxic CD8^+^ T cells, which play a crucial role in protective immunity against intracellular pathogens and tumors. NOD2 activation allows for DC-mediated cross-priming of cytotoxic CD8^+^ T cells by upregulating MHC class I-dependent antigen cross-presentation pathways.^9,96^

For this study, we narrowed down the selection of suitable desmuramylpeptides even further. In order to assess the adjuvant potential of selected desmuramylpeptides with regard to their effect on DC-mediated activation of CD4^+^ and CD8^+^ T cells, we conducted an *ex vivo* antigen-presentation assay utilizing C57BL/6 mouse bone marrow-derived DCs (BMDCs). Upon stimulation with desmuramylpeptides at 10 μM, in conjunction with ovalbumin soluble protein (50 μg/mL), BMDCs were cocultured with carboxyfluorescein succinimidyl ester (CFSE)-labeled naïve ovalbumin-specific CD4^+^ or CD8^+^ T cells, which were isolated from splenocytes of OT II and OT I transgenic mice, respectively. The expression of CD25 (the α subunit of the IL-2 receptor)^97^ as an indicator of T-cell activation, and CFSE dilution as a marker of T-cell proliferation were evaluated after 72 h. Additionally, we investigated how the priming of T cells elicited by the tested compounds impacted the cytokine profiles secreted in BMDC/T-cell cocultures.

**Figure 5:**
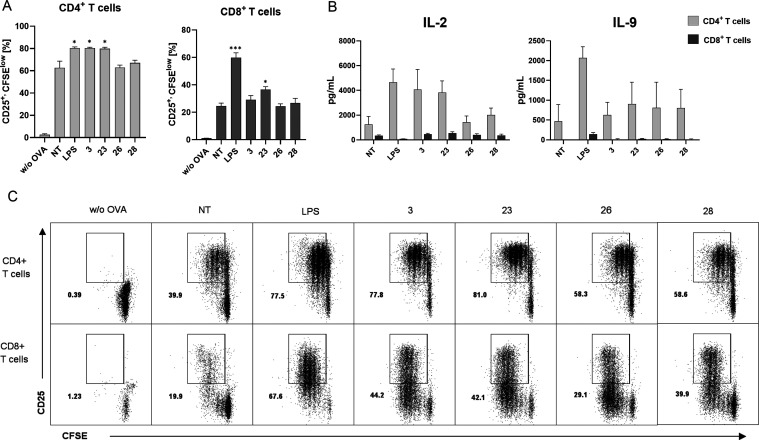
CD4^+^ and CD8^+^ T cell activation, proliferation, and cytokine secretion in response to ovalbumin presentation by bone-marrow-derived dendritic cells (BMDCs) pretreated with desmuramylpeptides. BMDCs from C57BL/6 mice were treated with desmuramylpeptides (10 μM) and ovalbumin (50 μg/mL). After 18 h, they were washed and cocultured for 72 h with CFSE-labeled ovalbumin-specific CD4^+^ or CD8^+^ T cells, isolated from OT II or OT I mouse splenocytes, respectively. (A) Quantification of the proportions of CD25^+^, CFSE^low^ T cells. The analysis regions are shown in panel C. (B) Cytokine concentrations in coculture supernatants following coincubation with BMDCs as described in panel A. (C) Representative dot plots of live Thy1.2^+^/CD4^+^ or Thy1.2^+^/CD8^+^ T cells, showing CD25 expression and CFSE dilution. Data are means ± SEM of triplicates (A) or duplicates (B) of two independent experiments. *, *p* < 0.05, ***, *p* < 0.001. Statistical significance was determined using paired *t* tests.

As depicted in Figure 5A, most desmuramylpeptides augmented CD4^+^ T-cell activation and proliferation mediated by BMDCs in spite of an unusually high fraction of proliferating cells in the OVA-treated cells serving as the control. Specifically, the effect of compounds featuring fatty acyl residues were most pronounced—they increased the fraction of activated and proliferating CD4^+^ T-cells from 63.8% (OVA-treated cells) to 79.7% (**23**) and 77.9% (**3**); positive control LPS achieved a comparable effect of 80.4%. The adamantane-decorated analog **28** (67.0%) and cholesterol-conjugated **26** (62.7%), on the other hand, had little to no impact in this regard. The enhanced T-cell activation was further characterized by increased levels of IL-2 and IL-9 (see Figure 5B). The positive control, LPS, was employed at a high concentration, and has therefore surpassed all tested desmuramylpeptides in the extent of secretion of all measured cytokines. Nonetheless, the impact of **23** and **3** was most notable in stimulating IL-2 and IL-9 production. IL-2 is known for promoting CD4^+^ T-cell growth and enhancing the activity of CD8^+^ and NK cells. While activated CD4^+^ T cells are major producers of IL-2, Th1 cells are typically recognized as the primary source.^98^ Other compounds displayed less pronounced activity on IL-2 secretion. Remarkably, the data revealed that **23**, and to a lesser extent **28** and **26**, augmented the production of the Th9-associated cytokine IL-9, suggesting a potential capacity to elicit a Th9 polarized response. To the best of our knowledge, this finding is the first one to establish a link between NOD2 engagement and IL-9 secretion. IL-9 is not only known for its protective role in immunity to parasites^99^ but also for its antitumor immunity achieved via activation of immune responses and by promoting tumor cell apoptosis.^100^ Our discovery is therefore noteworthy and provides a novel avenue of NOD2 agonists that needs to be further explored and potentially exploited. Further, **3**, **23**, and **28** also brought about slightly increased levels of IL-6 and TNF-α secreted by CD4^+^ T cells. Intriguingly, previous studies have associated NOD2 activation by MDP with driving Th2 polarization, characterized by reduced levels of IFN-γ and increased IL-4 production.^101^ However, it is worth noting that these findings were derived from studies utilizing human-monocyte-derived DCs, whereas our study employed BMDCs from C57BL/6 mice, a strain known for its Th1 dominance.^102^ To provide a comprehensive understanding of the Th1/Th2 polarization promoted by desmuramylpeptides, further investigations employing other mouse strains and human DCs are warranted.

The positive control LPS increased the fraction of activated and proliferating CD8^+^ T-cells to 55.6% (OVA-treated cells; 23.3%). A similar trend in the CD8^+^ T-cell activating capacity of selected desmuramylpeptides has been observed compared to that exerted on CD4^+^ T-cells, albeit less pronounced. Fatty acylated analogs exhibited a significant yet moderate increase in the processing and cross-presentation of ovalbumin to CD8^+^ T cells by BMDCs, bringing about an increase in their activation and proliferation (the fraction of activated and proliferating CD8^+^ T-cells was most pronounced for **23** (34.1%) and less so for **3** (29.3%)), as well as cytokine secretion. Other tested desmuramylpeptides were devoid of any activity on CD8^+^ T-cells. Previous studies, including the *in vitro* cross-priming assay conducted by Asano et al.,^9^ have demonstrated that activation of NOD1 and NOD2 led to enhanced proliferation of IFN-γ-secreting CD8^+^ T cells *in vivo*, consequently enhancing antigen-specific antitumor and antibacterial cytotoxic activities. Additionally, complementary data from intranasally immunized mice were reported for adamantylamide dipeptide, another member of the desmuramylpeptide class of adjuvants, and a structural congener of **28**.^103^ Given that most currently licensed adjuvants predominantly elicit antibody responses, there is great need for adjuvants capable of inducing cellular immunity, particularly in the field of cancer immunotherapy. The findings outlined above suggest that the eicosapentaenoylated derivative **23** holds a significant promise in addressing this need.

## 3 Conclusions

In the present study, we performed a focused structure–activity relationship optimization of **1**, which resulted in a library of structurally diverse, novel desmuramylpeptide NOD2 agonists. Our investigation has delineated the structural prerequisites that shape both physicochemical and immunomodulatory profiles of desmuramylpeptide NOD2 agonists. In particular, we identified compound **3** as a potent *in vitro* NOD2 agonist, exhibiting almost 17-fold enhancement in potency compared to **1**. The novel set of desmuramylpeptides demonstrated unique *in vitro* immunomodulatory activities. First, they elicited cytokine production in PBMCs, both alone and in the presence of LPS. Second, compound **32** stimulated the LPS-induced cytotoxic activity PBMCs against K562 cancer cells. Third, compound **26** displayed the capacity to convert the inflammatory monocyte subset into an anti-inflammatory phenotype. Fourthly, compound **23** augmented antigen presentation by BMDCs. On the basis of conducted *in vitro*/*ex vivo* experiments, **23** is a suitable candidate for follow-up research in *in vivo* assays to evaluate its potential as a vaccine adjuvant. Taken together, these data complement the existing yet limited reports on the structural features required to achieve the versatile biological activities of NOD2 agonists thus allowing for the design of more tunable immunomodulators.

## 4 Experimental Section

### 4.1 Materials

Materials. Chemicals were obtained from Sigma-Aldrich (St. Louis, MO, USA), Tokyo Chemical Industry (Tokyo, Japan), Acros Organics (Geel, Belgium), Enamine (Monmouth Junction, NJ, USA), and Apollo (Stockport, U.K.) and were used without further purification. MDP, and LPS (from *Escherichia coli* O55:B5) were obtained from InvivoGen, Inc. (San Diego, CA, USA). Analytical thin-layer chromatography (TLC) was performed on Merck 60 F254 silica gel plates (0.25 mm), with visualization using ultraviolet light, ninhydrin, and potassium permanganate. Column chromatography was carried out on silica gel 60 (particle size 240–400 mesh). ^1^H and ^13^C NMR spectra were recorded at 400 and 100 MHz, respectively, on an Avance III spectrometer (Bruker Corporation, Billerica, MA, USA) in CDCl_3_ or DMSO-*d*_6_ (MeOD) with tetramethylsilane as the internal standard. Mass spectra were obtained using an Exactive Plus orbitrap mass spectrometer (Thermo Fisher Scientific, Waltham, MA, USA) or on Expression CMS mass spectrometer (Advion Inc., Ithaca, NY, USA). Analytical ultra high-performance liquid chromatography (UHPLC) analyses were performed on a Dionex UltiMate 3000 Rapid Separation Binary System (Thermo Fisher Scientific, Waltham, MA, USA) equipped with an autosampler, a binary pump system, a photodiode array detector, a thermostated column compartment, and the Chromeleon Chromatography data system. The columns used were Waters Acquity UPLC BEH C18 (1.7 μm, 2.1 mm × 50 mm) or Waters Acquity UPLC CSH C18 (1.7 μm, 2.1 mm × 50 mm) with a flow rate of 0.3 mL/min. The eluent was a mixture of 0.1% TFA in water (A) and acetonitrile (B) with a gradient (%B) as follows: 0–10 min, 5–95%; 10–12 min, 95%; 12–12.5 min, 95–5%. The columns were thermostated at 40 °C. All of the compounds tested were established to be ≥95% pure. Compounds **1**, **2**, **10**, **27**, and **36** were prepared as previously described by our group^37,55^ The analytical data here were identical to those reported previously. The assembly of the final compounds was as described below.

### 4.2 General Synthetic Procedures

#### 4.2.1 General Procedure A: Acylation with Acyl Chlorides

To an ice-chilled stirring solution of **1** (1 equiv) in THF, Et_3_N (1.2 equiv) and the acyl chloride (1.2 equiv) were added dropwise and the resulting mixture was allowed to warm to room temperature. The stirring was continued for 1.5 h, after which the mixture was diluted with ethyl acetate (25 mL) and washed with 1 M HCl (2 × 10 mL), saturated NaHCO_3_ (2 × 10 mL), brine (10 mL), dried over anhydrous Na_2_SO_4_, concentrated *in vacuo*.

#### 4.2.2 General Procedure B: COMU-Mediated Coupling I

To an ice-chilled stirring solution of **1** (1 equiv) in DMF, carboxylic acid (1.1 equiv), DIPEA (3 equiv) and COMU (1.1 equiv) were added. The mixture was allowed to warm to room temperature and the stirring continued overnight after ethyl acetate (30 mL) was added and washed with 1 M HCl (2 × 20 mL), saturated NaHCO_3_ (2 × 20 mL), brine (30 mL), dried over anhydrous Na_2_SO_4_, concentrated *in vacuo*.

#### 4.2.3 General Procedure C: COMU-Mediated Coupling II

To an ice-chilled stirring solution of **27** (1 equiv) in DMF, amine (1.5 equiv), DIPEA (3 equiv) and COMU (1.5 equiv) were added. The mixture was allowed to warm to room temperature and the stirring continued overnight after which 1 M HCl (10 mL) was added. After 15 min ethyl acetate (2 × 30 mL) was added and washed with 1 M HCl (2 × 20 mL), saturated NaHCO_3_ (2 × 20 mL), brine (30 mL), dried over anhydrous Na_2_SO_4_, concentrated *in vacuo*.

### 4.2.4 General Procedure D: TFA-Mediated Acidolysis

The Boc-protected compound was added to an ice-chilled stirred mixture of TFA and DCM (1:5), and the mixture was allowed to warm to room temperature. After 3 h, the solvent was evaporated off *in vacuo*.

### 4.3 Synthesis and Characterization of Compounds

#### 4.3.1 Diethyl ((*E*)-3-(4-(Butyryloxy)-3-methoxyphenyl)acryloyl)glycyl-l-valyl-d-glutamate (**3**)

Synthesized from butyryl chloride (25 μL, 0.240 mmol, 1.6 equiv) using General procedure A. The crude product was purified by Isolera One flash chromatography (acetonitrile/0.1% TFA gradient from 20 to 100%) to obtain a compound **3** as a white solid (35 mg, 44%). ^1^H NMR (400 MHz, Chloroform-*d*) δ 7.64–7.54 (m, 2H), 7.48 (d, *J* = 8.9 Hz, 1H), 7.31 (t, *J* = 5.2 Hz, 1H), 7.14–7.08 (m, 2H), 6.99 (d, *J* = 8.0 Hz, 1H), 6.59–6.50 (m, 1H), 4.63–4.41 (m, 2H), 4.24–3.97 (m, 6H), 3.83 (s, 3H), 2.56 (t, *J* = 7.3 Hz, 2H), 2.46–2.31 (m, 2H), 2.26–2.11 (m, 2H), 2.11–1.96 (m, 1H), 1.84–1.70 (m, 2H), 1.26–1.17 (m, 6H), 1.05 (t, *J* = 7.4 Hz, 3H), 1.00–0.92 (m, 6H). ^13^C NMR (100 MHz, CDCl_3_) δ 172.79, 171.69, 171.52, 171.38, 169.51, 166.50, 151.34, 141.09, 140.95, 133.66, 123.16, 120.48, 120.30, 111.81, 77.37, 77.06, 76.74, 61.62, 60.75, 58.58, 55.88, 51.90, 43.59, 35.86, 31.05, 30.41, 26.84, 19.32, 18.52, 17.92, 14.13, 14.09, 13.57. HRMS *m*/*z* calculated for C_30_H_44_O_10_N_3_: (M + H)^+^ 606.3021, found 606.3007.

#### 4.3.2 Diethyl ((*E*)-3-(4-(Hexanoyloxy)-3-methoxyphenyl)acryloyl)glycyl-l-valyl-d-glutamate (**4**)

Synthesized from hexanoyl chloride (31 μL, 0.220 mmol) using General procedure A. The crude product was purified by column chromatography (MeOH/DCM 1:30) to give compound **4** as a white solid (54 mg, 54%). ^1^H NMR (400 MHz, Chloroform-*d*) δ 7.60 (d, *J* = 15.6 Hz, 1H), 7.35 (d, *J* = 7.6 Hz, 1H), 7.18–7.06 (m, 3H), 7.04–6.97 (m, 2H), 6.49 (d, *J* = 15.6 Hz, 1H), 4.63–4.39 (m, 2H), 4.23–4.02 (m, 6H), 3.84 (s, 3H), 2.57 (t, *J* = 7.5 Hz, 2H), 2.49–2.33 (m, 2H), 2.33–2.14 (m, 2H), 2.11–1.97 (m, 1H), 1.82–1.69 (m, 2H), 1.46–1.30 (m, 4H), 1.30–1.14 (m, 6H), 1.03–0.86 (m, 9H). ^13^C NMR (100 MHz, CDCl_3_) δ 172.92, 171.70, 171.66, 171.17, 169.37, 166.42, 151.37, 141.17, 133.56, 123.20, 120.62, 120.08, 111.64, 77.35, 77.04, 76.72, 61.66, 60.80, 58.53, 55.89, 51.96, 43.68, 33.99, 31.21, 30.85, 30.42, 26.80, 24.69, 22.34, 19.35, 17.75, 14.15, 14.11, 13.97. HRMS *m*/*z* calculated for C_32_H_48_O_10_N_3_: (M + H)^+^ 634.3334, found 634.3319.

#### 4.3.3 Diethyl ((*E*)-3-(3-Methoxy-4-(octanoyloxy)phenyl)acryloyl)glycyl-l-valyl-d-glutamate (**5**)

Synthesized from octanoyl chloride (38 μL, 0.220 mmol) using General procedure A. The crude product was purified by column chromatography (hexane/ethyl acetate 1:4) to give compound **5** as a white solid (41 mg, 41%). ^1^H NMR (400 MHz, Chloroform-*d*) δ 7.60 (d, *J* = 15.6 Hz, 1H), 7.18 (d, *J* = 7.3 Hz, 1H), 7.15–7.04 (m, 2H), 7.05–6.97 (m, 1H), 6.92 (d, *J* = 8.7 Hz, 1H), 6.81 (t, *J* = 5.3 Hz, 1H), 6.46 (d, *J* = 15.6 Hz, 1H), 4.60–4.50 (m, 1H), 4.47–4.37 (m, 1H), 4.25–4.04 (m, 6H), 3.85 (s, 3H), 2.58 (t, *J* = 7.5 Hz, 2H), 2.45–2.35 (m, 2H), 2.29–2.13 (m, 2H), 2.11–1.99 (m, 1H), 1.82–1.73 (m, 2H), 1.36–1.18 (m, 14H), 0.99–0.85 (m, 9H). ^13^C NMR (100 MHz, CDCl_3_) δ 173.03, 171.70, 171.64, 171.03, 169.28, 166.39, 151.39, 141.33, 141.24, 133.48, 123.21, 120.71, 119.91, 111.55, 77.34, 77.02, 76.71, 61.69, 60.85, 58.49, 55.89, 52.01, 43.73, 34.04, 31.70, 30.70, 30.42, 29.01, 28.94, 26.76, 25.00, 22.62, 19.35, 17.63, 14.14, 14.09. HRMS *m*/*z* calculated for C_34_H_52_O_10_N_3_: (M + H)^+^ 662.3647, found 662.3632.

#### 4.3.4 Diethyl ((*S*)-3-(2-((*E*)-3-(3-Methoxy-4-(nonanoyloxy)phenyl)acrylamido)acetamido)-4-methylpent-1-en-2-yl)-d-glutamate (**6**)

Synthesized from nonanoyl chloride (33 μL, 0.180 mmol) using General procedure A. The crude product was purified by Isolera One flash chromatography (acetonitrile/0.1% TFA gradient from 20 to 100%) to obtain a compound **6** as a white solid (27 mg, 34%). ^1^H NMR (400 MHz, Chloroform-*d*) δ 7.71 (d, *J* = 7.8 Hz, 1H), 7.68–7.53 (m, 2H), 7.45–7.36 (m, 1H), 7.15–7.06 (m, 2H), 6.99 (d, *J* = 8.0 Hz, 1H), 6.57 (d, *J* = 15.6 Hz, 1H), 4.67–4.47 (m, 2H), 4.34–3.97 (m, 6H), 3.83 (s, 3H), 2.57 (t, *J* = 7.5 Hz, 2H), 2.45–2.34 (m, 2H), 2.27–2.12 (m, 2H), 2.12–1.94 (m, 1H), 1.84–1.68 (m, 2H), 1.49–1.10 (m, 16H), 1.11–0.80 (m, 9H). ^13^C NMR (100 MHz, CDCl_3_) δ 172.75, 171.67, 171.41, 169.50, 166.48, 151.34, 141.06, 140.88, 133.70, 123.15, 120.39, 111.88, 77.38, 77.06, 76.74, 61.58, 60.72, 58.56, 55.88, 51.87, 43.57, 34.02, 31.82, 31.17, 30.41, 29.23, 29.16, 29.05, 26.86, 24.99, 22.65, 19.32, 17.98, 14.13, 14.10, 14.08. HRMS *m*/*z* calculated for C_35_H_54_O_10_N_3_: (M + H)^+^ 676.3804, found 676.3790.

#### 4.3.5 Diethyl ((*E*)-3-(4-(Decanoyloxy)-3-methoxyphenyl)acryloyl)glycyl-l-valyl-d-glutamate (**7**)

Synthesized from decanoyl chloride (46 μL, 0.220 mmol) using General procedure A. The crude product was purified by column chromatography (hexane/ethyl acetate 1:4) to give compound **7** as a white solid (21 mg, 20%). ^1^H NMR (400 MHz, Chloroform-*d*) δ 7.59 (d, *J* = 15.6 Hz, 1H), 7.35 (d, *J* = 7.7 Hz, 1H), 7.19–7.06 (m, 3H), 7.05–6.97 (m, 2H), 6.48 (d, *J* = 15.5 Hz, 1H), 4.64–4.49 (m, 1H), 4.53–4.39 (m, 1H), 4.25–4.03 (m, 6H), 3.84 (s, 3H), 2.57 (t, *J* = 7.5 Hz, 2H), 2.40 (q, *J* = 7.5, 7.1 Hz, 2H), 2.24–2.15 (m, 2H), 2.09–1.95 (m, 1H), 1.82–1.70 (m, 2H), 1.49–1.16 (m, 18H), 1.03–0.83 (m, 9H). ^13^C NMR (100 MHz, CDCl_3_) δ 172.94, 171.71, 171.21, 169.44, 166.48, 151.38, 141.25, 141.21, 133.51, 123.20, 120.65, 119.97, 111.61, 77.35, 77.03, 76.72, 61.69, 60.82, 58.58, 55.88, 51.97, 43.66, 34.04, 31.88, 30.78, 30.41, 29.47, 29.29, 29.06, 26.77, 25.01, 22.69, 19.34, 17.73, 14.13, 14.10. HRMS *m*/*z* calculated for C_36_H_56_O_10_N_3_: (M + H)^+^ 690.3960, found 690.3945.

#### 4.3.6 Diethyl ((*E*)-3-(3-Methoxy-4-(tetradecanoyloxy)phenyl)acryloyl)glycyl-l-valyl-d-glutamate (**8**)

Synthesized from myristoyl chloride (60 μL, 0.220 mmol) using General procedure A. The crude product was purified by column chromatography (ethyl acetate) to give compound **8** as a white solid (30 mg, 30%). ^1^H NMR (400 MHz, Chloroform-*d*) δ 7.59 (d, *J* = 15.6 Hz, 1H), 7.31 (d, *J* = 7.6 Hz, 1H), 7.14–7.01 (m, 3H), 7.01 (d, *J* = 7.5 Hz, 1H), 6.95 (t, *J* = 5.1 Hz, 1H), 6.47 (d, *J* = 15.6 Hz, 1H), 4.62–4.37 (m, 2H), 4.27–3.99 (m, 6H), 3.84 (s, 3H), 2.57 (t, *J* = 7.5 Hz, 2H), 2.40 (q, *J* = 7.2, 6.6 Hz, 2H), 2.26–2.12 (m, 2H), 2.10–1.98 (m, 1H), 1.76 (q, *J* = 7.5 Hz, 2H), 1.44–1.19 (m, 26H), 0.96 (dd, *J* = 11.8, 6.8 Hz, 6H), 0.91–0.86 (m, 3H). ^13^C NMR (100 MHz, CDCl_3_) δ 172.96, 171.70, 171.15, 169.39, 166.42, 151.38, 141.21, 133.52, 123.20, 120.67, 120.00, 111.59, 77.35, 77.03, 76.71, 61.68, 60.82, 58.55, 55.89, 51.97, 43.69, 34.04, 31.93, 30.76, 30.42, 29.70, 29.66, 29.63, 29.52, 29.36, 29.29, 29.07, 26.77, 25.01, 22.70, 19.35, 17.71, 14.14, 14.11. HRMS *m*/*z* calculated for C_40_H_64_O_10_N_3_: (M + H)^+^ 746.4586, found 746.4572.

#### 4.3.7 Diethyl ((*E*)-3-(3-Methoxy-4-(palmitoyloxy)phenyl)acryloyl)glycyl-l-valyl-d-glutamate (**9**)

Synthesized from palmitoyl chloride (55 μL, 0.180 mmol) using General procedure A. The crude product was purified by column chromatography (MeOH/DCM 1:50) to give compound **9** as a white solid (60 mg, 80%). ^1^H NMR (400 MHz, Chloroform-*d*) δ 7.70 (d, *J* = 7.8 Hz, 1H), 7.66–7.50 (m, 2H), 7.44–7.36 (m, 1H), 7.11 (d, *J* = 8.5 Hz, 2H), 6.99 (d, *J* = 8.0 Hz, 1H), 6.57 (d, *J* = 15.6 Hz, 1H), 4.65–4.45 (m, 2H), 4.33–4.01 (m, 6H), 3.83 (s, 3H), 2.57 (t, *J* = 7.5 Hz, 2H), 2.45–2.34 (m, 2H), 2.17 (d, *J* = 4.7 Hz, 2H), 2.11–1.97 (m, 1H), 1.82–1.68 (m, 2H), 1.43–1.18 (m, 30H), 0.97 (dd, *J* = 11.0, 6.7 Hz, 6H), 0.88 (t, *J* = 6.7 Hz, 3H). ^13^C NMR (100 MHz, CDCl_3_) δ 172.75, 171.67, 171.66, 171.40, 169.50, 166.48, 151.34, 141.07, 140.88, 133.70, 123.15, 120.40, 111.88, 77.38, 77.06, 76.74, 61.58, 60.72, 58.56, 55.88, 51.87, 43.58, 34.03, 31.92, 31.16, 30.41, 29.70, 29.66, 29.63, 29.52, 29.36, 29.30, 29.07, 26.86, 25.00, 22.69, 19.32, 17.98, 14.13, 14.08. HRMS *m*/*z* calculated for C_42_H_68_O_10_N_3_: (M + H)^+^ 774.4899, found 774.4889.

#### 4.3.8 Diethyl ((*E*)-3-(3-Methoxy-4-((2-methylheptanoyl)oxy)phenyl)acryloyl)glycyl-l-valyl-d-glutamate (**11**)

Synthesized from 2-methylheptanoic acid (50 μL, 0.320 mmol) using General procedure B. The crude product was purified by column chromatography (MeOH/DCM 1:30) to give compound **11** as a white solid (44 mg, 41%). ^1^H NMR (400 MHz, Chloroform-*d*) δ 7.61 (d, *J* = 15.5 Hz, 1H), 7.15–7.07 (m, 2H), 6.99 (dd, *J* = 14.1, 7.8 Hz, 2H), 6.65 (d, *J* = 8.7 Hz, 1H), 6.58–6.50 (m, 1H), 6.42 (d, *J* = 15.6 Hz, 1H), 4.62–4.44 (m, 1H), 4.44–4.31 (m, 1H), 4.24–4.06 (m, 5H), 3.84 (s, 3H), 2.78–2.64 (m, 1H), 2.50–1.95 (m, 5H), 1.87–1.72 (m, 1H), 1.49–1.39 (m, 2H), 1.38–1.17 (m, 15H), 1.03–0.86 (m, 9H). HRMS *m*/*z* calculated for C_34_H_52_O_10_N_3_: (M + H)^+^ 662.3647, found 662.3632.

#### 4.3.9 Diethyl ((*E*)-3-(4-((2-ethylhexanoyl)oxy)-3-methoxyphenyl)acryloyl)glycyl-l-valyl-d-glutamate (**12**)

Synthesized from 2-ethylhexanoic acid (30 mg, 0.205 mmol) using General procedure B. The crude product was purified by column chromatography (MeOH/DCM 1:30) to give compound **12** as a white solid (30 mg, 24%). ^1^H NMR (400 MHz, Chloroform-*d*) δ 7.64–7.54 (m, 2H), 7.46 (d, *J* = 8.9 Hz, 1H), 7.33–7.24 (m, 1H), 7.11 (d, *J* = 8.2 Hz, 2H), 6.97 (d, *J* = 7.9 Hz, 1H), 6.54 (d, *J* = 15.6 Hz, 1H), 4.64–4.44 (m, 2H), 4.28–3.97 (m, 6H), 3.82 (s, 3H), 2.60–2.47 (m, 1H), 2.44–2.33 (m, 2H), 2.23–2.14 (m, 2H), 2.10–1.96 (m, 1H), 1.85–1.72 (m, 2H), 1.69–1.54 (m, 2H), 1.41 (tt, *J* = 16.7, 5.8 Hz, 4H), 1.28–1.14 (m, 6H), 1.08–0.87 (m, 12H). ^13^C NMR (100 MHz, CDCl_3_) δ 174.16, 172.81, 171.71, 171.39, 169.58, 166.54, 151.46, 141.19, 141.01, 133.62, 123.22, 120.53, 120.24, 111.74, 61.62, 60.76, 58.61, 55.74, 51.92, 47.27, 43.60, 31.86, 30.97, 30.42, 29.52, 26.84, 25.60, 22.67, 19.33, 17.89, 14.14, 14.09, 14.03, 11.75. HRMS *m*/*z* calculated for C_34_H_52_O_10_N_3_: (M + H)^+^ 662.3647, found 662.3638.

#### 4.3.10 Diethyl ((*E*)-3-(4-((2-Ethyloctanoyl)oxy)-3-methoxyphenyl)acryloyl)glycyl-l-valyl-d-glutamate (**13**)

Synthesized from 2-ethyloctanoic acid (45 μL, 0.240 mmol) using General procedure B. The crude product was purified by Isolera One flash chromatography (acetonitrile/0.1% TFA gradient from 20 to 100%) to obtain a compound **13** as a white solid (14 mg, 18%). ^1^H NMR (400 MHz, Chloroform-*d*) δ 7.60 (d, *J* = 15.6 Hz, 1H), 7.40 (d, *J* = 7.6 Hz, 1H), 7.21 (d, *J* = 8.8 Hz, 1H), 7.08 (d, *J* = 13.3 Hz, 3H), 6.98 (d, *J* = 8.0 Hz, 1H), 6.49 (d, *J* = 15.6 Hz, 1H), 4.65–4.50 (m, 1H), 4.50–4.40 (m, 1H), 4.21–4.03 (m, 6H), 3.83 (s, 3H), 2.58–2.49 (m, 1H), 2.45–2.35 (m, 2H), 2.21 (tq, *J* = 13.3, 6.9 Hz, 2H), 2.11–1.98 (m, 1H), 1.84–1.72 (m, 2H), 1.69–1.51 (m, 2H), 1.43–1.17 (m, 14H), 1.05–0.85 (m, 12H). ^13^C NMR (100 MHz, CDCl_3_) δ 174.15, 172.91, 171.72, 171.23, 169.43, 166.47, 151.47, 141.23, 141.17, 133.51, 123.24, 120.61, 120.03, 111.62, 77.35, 77.03, 76.72, 61.66, 60.80, 58.56, 55.74, 51.95, 47.31, 43.67, 32.18, 31.78, 30.83, 30.42, 29.29, 27.31, 26.79, 25.60, 22.63, 19.34, 17.76, 14.14, 14.10, 11.76. HRMS *m*/*z* calculated for C_36_H_56_O_10_N_3_: (M + H)^+^ 690.3960, found 690.3942.

#### 4.3.11 Diethyl ((*E*)-3-(3-Methoxy-4-((2-propylheptanoyl)oxy)phenyl)acryloyl)glycyl-l-valyl-d-glutamate (**14**)

Synthesized from 2-propylheptanoic acid (50 μL, 0.260 mmol) using General procedure B. The crude product was purified by column chromatography (MeOH/DCM 1:30) to give compound **14** as a white solid (10 mg, 12%). ^1^H NMR (400 MHz, Chloroform-*d*) δ 7.61 (d, *J* = 15.6 Hz, 1H), 7.16–7.05 (m, 3H), 6.99 (d, *J* = 8.0 Hz, 1H), 6.87 (d, *J* = 8.7 Hz, 1H), 6.74 (t, *J* = 5.2 Hz, 1H), 6.45 (d, *J* = 15.6 Hz, 1H), 4.61–4.49 (m, 1H), 4.44–4.34 (m, 1H), 4.23–4.04 (m, 6H), 3.83 (s, 3H), 2.69–2.55 (m, 1H), 2.48–2.35 (m, 2H), 2.30–2.15 (m, 2H), 2.04 (td, *J* = 14.5, 7.9 Hz, 1H), 1.76 (dtd, *J* = 13.9, 9.3, 5.3 Hz, 2H), 1.65–1.26 (m, 10H), 1.24 (dt, *J* = 10.2, 7.1 Hz, 6H), 1.01–0.89 (m, 12H). ^13^C NMR (100 MHz, CDCl_3_) δ 174.28, 173.04, 171.62, 171.02, 169.29, 166.41, 151.50, 141.41, 141.32, 133.41, 123.28, 120.71, 119.81, 111.51,, 76.70, 61.69, 60.85, 58.49, 55.76, 52.02, 45.49, 43.74, 34.72, 32.53, 31.81, 30.63, 30.41, 27.04, 26.74, 22.60, 20.55, 19.36, 17.59, 14.14, 14.12, 14.07. HRMS *m*/*z* calculated for C_36_H_56_O_10_N_3_: (M + H)^+^ 690.3960, found 690.3945.

#### 4.3.12 Diethyl ((*E*)-3-(4-((2-Butylhexanoyl)oxy)-3-methoxyphenyl)acryloyl)glycyl-l-valyl-d-glutamate (**15**)

Synthesized from 2-butylhexanoic acid (50 μL, 0.350 mmol) using General procedure B. The crude product was purified by column chromatography (MeOH/DCM 1:20) to give compound **15** as a white solid (44 mg, 31%). ^1^H NMR (400 MHz, Chloroform-*d*) δ 7.60 (d, *J* = 15.6 Hz, 1H), 7.17 (d, *J* = 7.5 Hz, 1H), 7.13–7.07 (m, 2H), 6.98 (dd, *J* = 8.4, 4.5 Hz, 2H), 6.82 (t, *J* = 5.3 Hz, 1H), 6.46 (d, *J* = 15.6 Hz, 1H), 4.60–4.49 (m, 1H), 4.46–4.38 (m, 1H), 4.24–3.99 (m, 6H), 3.84 (s, 3H), 2.65–2.53 (m, 1H), 2.46–2.31 (m, 2H), 2.29–2.13 (m, 2H), 2.08–1.98 (m, 1H), 1.84–1.55 (m, 6H), 1.44–1.35 (m, 6H), 1.24 (dt, *J* = 10.4, 7.2 Hz, 6H), 0.99–0.89 (m, 12H). ^13^C NMR (100 MHz, CDCl_3_) δ 180.07, 174.31, 173.01, 171.60, 171.09, 169.38, 166.43, 151.49, 141.36, 141.29, 133.44, 123.28, 120.70, 119.86, 111.50,, 61.70, 60.85, 58.53, 55.71, 52.02, 45.68, 45.24, 43.69, 32.29, 31.98, 30.67, 30.40, 29.58, 29.55, 26.75, 22.68, 22.63, 19.35, 17.64, 14.14, 14.11, 14.04, 13.94. HRMS *m*/*z* calculated for C_36_H_56_O_10_N_3_: (M + H)^+^ 690.3960, found 690.3944.

#### 4.3.13 Diethyl ((*E*)-3-(4-((4-Cyclohexylbutanoyl)oxy)-3-methoxyphenyl)acryloyl)glycyl-l-valyl-d-glutamate (**16**)

Synthesized from 4-cyclohexylbutyric acid (50 μL, 0.280 mmol) using General procedure B. The crude product was purified by column chromatography (MeOH/DCM 1:40) to give compound **16** as a white solid (68 mg, 73%). ^1^H NMR (400 MHz, Chloroform-*d*) δ 7.63–7.52 (m, 2H), 7.46 (d, *J* = 8.9 Hz, 1H), 7.28–7.24 (m, 1H), 7.13–7.07 (m, 2H), 6.99 (d, *J* = 8.1 Hz, 1H), 6.53 (d, *J* = 15.6 Hz, 1H), 4.62–4.45 (m, 2H), 4.24–4.02 (m, 6H), 3.83 (s, 3H), 2.55 (t, *J* = 7.5 Hz, 2H), 2.47–2.36 (m, 2H), 2.36–2.27 (m, 1H), 2.25–2.14 (m, 2H), 2.09–1.99 (m, 1H), 1.81–1.66 (m, 7H), 1.34–1.17 (m, 13H), 1.00–0.93 (m, 6H). ^13^C NMR (100 MHz, CDCl_3_) δ 172.80, 171.68, 171.64, 171.36, 169.54, 166.47, 151.35, 141.11, 140.98, 133.64, 123.17, 120.51, 120.27, 111.76, 61.62, 60.75, 58.60, 55.88, 51.91, 43.57, 37.39, 36.79, 34.31, 33.29, 33.24, 31.01, 30.41, 26.84, 26.66, 26.35, 22.42, 22.28, 19.33, 17.90, 14.14, 14.10. HRMS *m*/*z* calculated for C_36_H_54_O_10_N_3_: (M + H)^+^ 688.3804, found 688.3790.

#### 4.3.14 Diethyl ((*E*)-3-(3-Methoxy-4-((2-pentylheptanoyl)oxy)phenyl)acryloyl)glycyl-l-valyl-d-glutamate (**17**)

Synthesized from 2-pentylheptanoic acid (40 μL, 0.180 mmol) using General procedure B. The crude product was purified by column chromatography (MeOH/DCM 1:50) to give compound **17** as a white solid (50 mg, 63%). ^1^H NMR (400 MHz, Chloroform-*d*) δ 7.62 (d, *J* = 15.5 Hz, 1H), 7.15–7.06 (m, 2H), 7.02–6.97 (m, 1H), 6.93 (d, *J* = 7.4 Hz, 1H), 6.57 (d, *J* = 8.6 Hz, 1H), 6.50–6.38 (m, 2H), 4.60–4.47 (m, 1H), 4.43–4.32 (m, 1H), 4.25–4.03 (m, 6H), 3.84 (s, 3H), 2.68–2.55 (m, 1H), 2.50–2.13 (m, 4H), 2.12–1.99 (m, 1H), 1.86–1.67 (m, 2H), 1.50–1.30 (m, 13H), 1.29–1.20 (m, 7H), 1.00–0.88 (m, 12H). HRMS *m*/*z* calculated for C_38_H_60_O_10_N_3_: (M + H)^+^ 718.4273, found 718.4256.

#### 4.3.15 Diethyl ((*E*)-3-(3-Methoxy-4-(oleoyloxy)phenyl)acryloyl)glycyl-l-valyl-d-glutamate (**18**)

Synthesized from (9*Z*)-9-octadecanoic acid (58 mg, 0.187 mmol) using General procedure B. The crude product was purified by column chromatography (MeOH/DCM 1:40) to give compound **18** as a white solid (90 mg, 60%). ^1^H NMR (400 MHz, Chloroform-*d*) δ 7.61 (d, *J* = 15.6 Hz, 1H), 7.14–7.08 (m, 2H), 7.05–6.98 (m, 2H), 6.71 (d, *J* = 8.7 Hz, 1H), 6.59 (t, *J* = 5.3 Hz, 1H), 6.43 (d, *J* = 15.6 Hz, 1H), 5.37–5.33 (m, 2H), 4.58–4.50 (m, 1H), 4.43–4.36 (m, 1H), 4.24–4.03 (m, 6H), 3.85 (s, 3H), 2.58 (t, *J* = 7.5 Hz, 2H), 2.44–2.33 (m, 2H), 2.30–2.15 (m, 2H), 2.06–1.98 (m, 5H), 1.80–1.72 (m, 2H), 1.36–1.21 (m, 26H), 0.96 (dd, *J* = 14.3, 6.8 Hz, 6H), 0.87 (d, *J* = 6.9 Hz, 3H). ^13^C NMR (100 MHz, CDCl_3_) δ 172.65, 171.60, 171.54, 169.58, 166.49, 151.33, 141.01, 140.75, 133.79, 130.03, 129.71, 123.13, 120.57, 120.27, 112.02, 61.53, 60.67, 58.58, 55.87, 51.84, 43.49, 34.00, 31.90, 31.34, 30.39, 29.77, 29.71, 29.52, 29.32, 29.22, 29.19, 29.15, 29.04, 27.22, 27.18, 26.90, 24.98, 22.68, 19.30, 18.10, 14.12, 14.07. HRMS *m*/*z* calculated for C_44_H_70_O_10_N_3_: (M + H)^+^ 800.5056, found 800.5048.

#### 4.3.16 Diethyl ((*E*)-3-(3-Methoxy-4-(((9*Z*,12*Z*)-octadeca-9,12-dienoyl)oxy)phenyl)acryloyl)glycyl-l-valyl-d-glutamate (**19**)

Synthesized from (9*Z*, 12*Z*)-octadeca-9,12-dienoic acid (58 mg, 0.187 mmol) using General procedure B. The crude product was purified by column chromatography (MeOH/DCM 1:50) to give compound **19** as a white solid (45 mg, 31%). ^1^H NMR (400 MHz, Chloroform-*d*) δ 7.61 (d, *J* = 15.6 Hz, 1H), 7.16–7.08 (m, 2H), 7.01 (dd, *J* = 13.9, 7.8 Hz, 2H), 6.67 (d, *J* = 8.7 Hz, 1H), 6.55 (t, *J* = 5.3 Hz, 1H), 6.43 (d, *J* = 15.6 Hz, 1H), 5.43–5.30 (m, 4H), 4.59–4.49 (m, 1H), 4.44–4.35 (m, 1H), 4.22–4.04 (m, 6H), 3.85 (s, 3H), 2.78 (t, *J* = 6.5 Hz, 2H), 2.58 (t, *J* = 7.5 Hz, 2H), 2.46–2.16 (m, 4H), 2.09–2.00 (m, 5H), 1.81–1.69 (m, 2H), 1.40–1.22 (m, 20H), 0.96 (dd, *J* = 14.6, 6.8 Hz, 6H), 0.89 (t, *J* = 6.8 Hz, 3H). ^13^C NMR (100 MHz, CDCl_3_) δ 172.78, 171.63, 171.37, 169.51, 166.46, 151.35, 141.09, 140.94, 133.67, 130.23, 130.02, 128.08, 127.89, 123.15, 120.47, 120.32, 111.80,, 61.60, 60.74, 58.58, 55.88, 51.90, 43.57, 34.01, 31.52, 31.06, 30.40, 29.62, 29.34, 29.19, 29.15, 29.04, 27.20, 26.85, 25.64, 24.98, 22.58, 19.32, 17.92, 14.13, 14.08. HRMS *m*/*z* calculated for C_44_H_68_O_10_N_3_: (M + H)^+^ 798.4899, found 798.4887.

#### 4.3.17 Diethyl ((*E*)-3-(3-Methoxy-4-(((9*Z*,12*Z*,15*Z*)-octadeca-9,12,15-trienoyl)oxy)phenyl)acryloyl)glycyl-l-valyl-d-glutamate (**20**)

Synthesized from (9*Z*, 12*Z*, 15*Z*)-octadeca-9,12,15-trienoic acid (58 mg, 0.205 mmol) using General procedure B. The crude product was purified by column chromatography (MeOH/DCM 1:50) to give compound **20** as a white solid (45 mg, 67%). ^1^H NMR (400 MHz, Chloroform-*d*) δ 7.61 (d, *J* = 15.6 Hz, 1H), 7.16–7.08 (m, 3H), 7.01 (d, *J* = 8.0 Hz, 1H), 6.90 (d, *J* = 8.7 Hz, 1H), 6.76 (t, *J* = 5.2 Hz, 1H), 6.45 (d, *J* = 15.6 Hz, 1H), 5.46–5.24 (m, 6H), 4.61–4.49 (m, 1H), 4.49–4.35 (m, 1H), 4.24–4.03 (m, 6H), 3.85 (s, 3H), 2.86–2.76 (m, 4H), 2.58 (t, *J* = 7.5 Hz, 2H), 2.47–2.32 (m, 2H), 2.28–2.15 (m, 2H), 2.13–2.00 (m, 5H), 1.81–1.71 (m, 2H), 1.43–1.31 (m, 8H), 1.24 (dt, *J* = 9.8, 7.1 Hz, 6H), 1.01–0.88 (m, 9H). ^13^C NMR (100 MHz, CDCl_3_) δ 172.79, 171.66, 171.64, 171.38, 169.54, 166.47, 151.35, 141.10, 140.97, 133.65, 131.97, 130.24, 128.30, 128.24, 127.77, 127.11, 123.16, 120.50, 120.28, 111.77,, 61.62, 60.75, 58.60, 55.88, 51.91, 43.57, 34.01, 31.01, 30.41, 29.60, 29.19, 29.15, 29.03, 27.21, 26.84, 25.63, 25.54, 24.98, 20.56, 19.33, 17.90, 14.29, 14.14, 14.09. HRMS *m*/*z* calculated for C_44_H_66_O_10_N_3_: (M + H)^+^ 796.4743, found 796.4736.

#### 4.3.18 Diethyl ((*E*)-3-(3-Methoxy-4-(((6*Z*,9*Z*,12*Z*)-octadeca-6,9,12-trienoyl)oxy)phenyl)acryloyl)glycyl-l-valyl-d-glutamate (**21**)

Synthesized from (6*Z*, 9*Z*, 12*Z*)-octadeca-6,9,12-trienoic acid (58 mg, 0.187 mmol) using General procedure B. The crude product was purified by column chromatography (MeOH/DCM 1:50) to give compound **21** as a white solid (80 mg, 54%). ^1^H NMR (400 MHz, Chloroform-*d*) δ 7.60 (d, *J* = 15.6 Hz, 1H), 7.35 (d, *J* = 7.5 Hz, 1H), 7.18 (d, *J* = 8.5 Hz, 1H), 7.15–7.06 (m, 2H), 7.00 (d, *J* = 7.7 Hz, 2H), 6.49 (d, *J* = 15.6 Hz, 1H), 5.48–5.21 (m, 6H), 4.59–4.52 (m, 1H), 4.49–4.42 (m, 1H), 4.24–4.03 (m, 6H), 3.84 (s, 3H), 2.86–2.77 (m, 4H), 2.59 (t, *J* = 7.4 Hz, 2H), 2.48–2.33 (m, 2H), 2.24–2.11 (m, 4H), 2.09–2.01 (m, 3H), 1.84–1.73 (m, 2H), 1.56–1.47 (m, 2H), 1.39–1.28 (m, 6H), 1.26–1.20 (m, 6H), 0.96 (dd, *J* = 11.8, 6.8 Hz, 6H), 0.91–0.85 (m, 3H). ^13^C NMR (100 MHz, CDCl_3_) δ 172.90, 171.65, 171.48, 171.20, 169.41, 166.41, 151.35, 141.15, 133.58, 130.48, 129.57, 128.46, 128.43, 128.33, 128.05, 127.57, 123.17, 120.62, 120.10, 111.63, 7, 61.66, 60.80, 58.56, 55.88, 51.96, 43.65, 33.90, 31.52, 30.84, 30.41, 29.32, 28.99, 27.22, 26.88, 26.80, 25.65, 24.62, 22.58, 19.34, 17.76, 14.14, 14.11, 14.08. HRMS *m*/*z* calculated for C_44_H_66_O_10_N_3_: (M + H)^+^ 796.4743, found 796.4737.

#### 4.3.19 Diethyl ((*E*)-3-(4-(((4*Z*,7*Z*,10*Z*,13*Z*,16*Z*,19*Z*)-Docosa-4,7,10,13,16,19-hexaenoyl)oxy)-3-methoxyphenyl)acryloyl)glycyl-l-valyl-d-glutamate (**22**)

Synthesized from docosahexaenoic acid (67 mg, 0.187 mmol) using General procedure B. The crude product was purified by column chromatography (MeOH/DCM 1:50) to give compound **22** as a white solid (100 mg, 63%). ^1^H NMR (400 MHz, Chloroform-*d*) δ 7.60 (d, *J* = 15.6 Hz, 1H), 7.40 (d, *J* = 7.7 Hz, 1H), 7.26 (d, *J* = 8.9 Hz, 1H), 7.15–7.05 (m, 3H), 7.04–6.94 (m, 1H), 6.50 (d, *J* = 15.7 Hz, 1H), 5.48–5.32 (m, 12H), 4.61–4.53 (m, 1H), 4.47 (dd, *J* = 8.9, 6.1 Hz, 1H), 4.22–4.00 (m, 6H), 3.84 (s, 3H), 2.88–2.79 (m, 10H), 2.69–2.60 (m, 2H), 2.52 (dd, *J* = 12.7, 7.1 Hz, 2H), 2.43–2.36 (m, 2H), 2.20 (dt, *J* = 14.1, 6.9 Hz, 2H), 2.14–2.00 (m, 3H), 1.32–1.14 (m, 6H), 1.02–0.88 (m, 9H). ^13^C NMR (100 MHz, CDCl_3_) δ 172.87, 171.63, 171.24, 170.95, 169.43, 166.41, 151.32, 141.08, 133.65, 132.04, 129.53, 128.57, 128.33, 128.28, 128.27, 128.09, 128.07, 128.04, 127.87, 127.66, 127.02, 123.16, 120.59, 120.17, 111.66,, 61.65, 60.79, 58.56, 55.90, 51.95, 43.63, 33.91, 30.90, 30.40, 26.81, 25.64, 25.54, 22.80, 20.56, 19.34, 17.80, 14.28, 14.14, 14.10. HRMS *m*/*z* calculated for C_44_H_66_O_10_N_3_: (M + H)^+^ 846.4899, found 846.4889.

#### 4.3.20 Diethyl ((*E*)-3-(4-(((5*Z*,8*Z*,11*Z*,14*Z*,17*Z*)-Icosa-5,8,11,14,17-pentaenoyl)oxy)-3-methoxyphenyl)acryloyl)glycyl-l-valyl-d-glutamate (**23**)

Synthesized from docosahexaenoic acid (62 mg, 0.187 mmol) using General procedure B. The crude product was purified by column chromatography (MeOH/DCM 1:50) to give compound **23** as a white solid (73 mg, 48%). ^1^H NMR (400 MHz, Chloroform-*d*) δ 7.60 (d, *J* = 15.6 Hz, 1H), 7.31–7.27 (m, 1H), 7.14–7.05 (m, 3H), 7.02–6.98 (m, 1H), 6.96–6.89 (m, 1H), 6.48 (d, *J* = 15.6 Hz, 1H), 5.45–5.28 (m, 10H), 4.60–4.39 (m, 2H), 4.22–4.04 (m, 6H), 3.84 (s, 3H), 2.88–2.79 (m, 8H), 2.59 (t, *J* = 7.5 Hz, 2H), 2.45–2.34 (m, 2H), 2.25–2.17 (m, 3H), 2.10–2.05 (m, 2H), 1.87–1.80 (m, 2H), 1.29–1.16 (m, 6H), 1.03–0.89 (m, 9H). ^13^C NMR (100 MHz, CDCl_3_) δ 172.94, 171.63, 171.43, 171.13, 169.35, 166.38, 151.35, 141.19, 141.13, 133.58, 132.05, 129.03, 128.86, 128.59, 128.29, 128.23, 128.20, 128.08, 127.87, 127.01, 123.17, 120.65, 120.05, 111.59,, 61.66, 60.81, 58.53, 55.89, 51.98, 43.68, 33.39, 30.80, 30.41, 26.78, 26.48, 25.64, 25.55, 24.84, 20.56, 19.34, 17.72, 14.28, 14.14, 14.11. HRMS *m*/*z* calculated for C_46_H_66_O_10_N_3_: (M + H)^+^ 820.4743, found 820.4734.

#### 4.3.21 Diethyl ((*E*)-3-(4-((14-Hydroxytetradecanoyl)oxy)-3-methoxyphenyl)acryloyl)glycyl-l-valyl-d-glutamate (**24**)

Synthesized from 14-hydroxytetradecanoic acid (59 μL, 0.240 mmol) using General procedure B. The crude product was purified by Isolera One flash chromatography (acetonitrile/0.1% TFA gradient from 20 to 100%) to obtain a compound **24** as a white solid (30 mg, 38%). ^1^H NMR (400 MHz, Chloroform-*d*) δ 7.67–7.53 (m, 2H), 7.48 (d, *J* = 8.8 Hz, 1H), 7.42–7.30 (m, 1H), 7.10 (d, *J* = 6.9 Hz, 2H), 6.99 (d, *J* = 8.3 Hz, 1H), 6.53 (d, *J* = 15.6 Hz, 1H), 4.61–4.52 (m, 1H), 4.52–4.38 (m, 1H), 4.27–4.00 (m, 6H), 3.83 (s, 3H), 3.63 (t, *J* = 6.7 Hz, 2H), 2.57 (t, *J* = 7.4 Hz, 2H), 2.45–2.35 (m, 2H), 2.26–2.13 (m, 2H), 2.09–1.98 (m, 1H), 1.80–1.70 (m, 2H), 1.57 (q, *J* = 6.9 Hz, 2H), 1.45–1.17 (m, 25H), 1.01–0.89 (m, 6H). ^13^C NMR (100 MHz, CDCl_3_) δ 174.28, 173.04, 171.62, 171.02, 169.29, 166.41, 151.50, 141.41, 141.32, 133.41, 123.28, 120.71, 119.81, 111.51,, 61.69, 60.85, 58.49, 55.76, 52.02, 45.49, 43.74, 34.72, 32.53, 31.81, 30.63, 30.41, 27.04, 26.74, 22.60, 20.55, 19.36, 17.59, 14.14, 14.12, 14.07. HRMS *m*/*z* calculated for C_40_H_64_O_11_N_3_: (M + H)^+^ 762.4535, found 762.4516.

#### 4.3.22 2-(Cholesteryloxy)-ethanoic Acid (**25**)

To a stirring solution of cholesterol (200 mg, 0.517 mmol) in dry DCM (2 mL), ethyl diazoacetate solution 15% in toluene (483 μL, 0569 mmol) and boron trifluoride etherate (10 μL, 0.052 mmol) were added. The stirring continued overnight after which saturated NaHCO_3_ (10 mL) was added and the product was extracted with DCM (3 × 15 mL). The combined organic phases were dried with Na_2_SO_4_ and concentrated under reduced pressure. The crude product was purified by column chromatography (petroleum ether/ethyl acetate gradient from 100 to 70%) to give compound as a white solid (25 mg, 10%). The resulting compound (25 mg, 0.053 mmol) was dissolved in ethanol (3 mL) and chilled on ice. 2 M NaOH (40 μL, 0.080 mmol) was added and the mixture was allowed to warm to room temperature and the stirring continued overnight after which the solution was neutralized with 1 M HCl and concentrated under reduced pressure to give compound **25** as a white solid (23 mg, 100%). ^1^H NMR (400 MHz, Chloroform-*d*) δ 5.38 (d, *J* = 5.3 Hz, 1H), 4.12 (s, 2H), 3.32–3.17 (m, *J* = 11.3, 5.6, 4.7 Hz, 1H), 2.46–2.20 (m, 2H), 2.05–0.76 (m, 50H), 0.68 (s, 3H). *H_2_O is present in NMR spectra.

#### 4.3.23 Diethyl ((*E*)-3-(4-(2-(((8*S*,9*S*,10*R*,13*R*,14*S*,17*R*)-10,13-Dimethyl-17-((*R*)-6-methylheptan-2-yl)-2,3,4,7,8,9,10,11,12,13,14,15,16,17-tetradecahydro-1*H*-cyclopenta[*a*]phenanthren-3-yl)oxy)acetoxy)-3-methoxyphenyl)acryloyl)glycyl-l-valyl-d-glutamate (**26**)

To an ice-chilled stirring solution of **1** (26 mg, 0.048 mmol) in DMF (1 mL), solution od **25** (23 mg, 0.0529 mmol) in DCM (1.5 mL), DIPEA (41 μL, 0.240 mmol) and COMU (23 mg, 0.0529 mmol) were added. The mixture was allowed to warm to room temperature and the stirring continued overnight after which ethyl acetate (30 mL) was added and washed with 1 M HCl (2 × 20 mL), saturated NaHCO_3_ (2 × 20 mL), brine (30 mL), dried over anhydrous Na_2_SO_4_, concentrated *in vacuo*. The crude product was purified by column chromatography (hexane/ethyl acetate 1:4) to give compound **26** as a white solid (20 mg, 43%). ^1^H NMR (400 MHz, Chloroform-*d*) δ 7.59 (d, *J* = 15.5 Hz, 1H), 7.42 (d, *J* = 7.7 Hz, 1H), 7.23 (d, *J* = 8.8 Hz, 1H), 7.14–7.05 (m, 3H), 7.03 (d, *J* = 8.1 Hz, 1H), 6.51 (d, *J* = 15.6 Hz, 1H), 5.42–5.34 (m, 1H), 4.62–4.44 (m, 2H), 4.41 (s, 2H), 4.20–4.05 (m, 6H), 3.82 (s, 3H), 3.45–3.28 (m, 1H), 2.49–2.28 (m, 4H), 2.25–2.12 (m, 2H), 2.07–1.97 (m, 4H), 1.89 (d, *J* = 8.7 Hz, 2H), 1.59–0.83 (m, 46H), 0.68 (s, 3H). ^13^C NMR (100 MHz, CDCl3) δ 172.98, 171.64, 171.08, 169.30, 168.92, 166.33, 151.19, 141.14, 140.60, 140.48, 133.77, 123.05, 122.04, 120.63, 120.15, 111.56, 80.34,, 65.46, 61.68, 60.83, 58.50, 56.76, 56.15, 55.90, 51.99, 50.18, 43.70, 42.32, 39.76, 39.51, 38.70, 37.16, 36.82, 36.19, 35.79, 31.95, 31.88, 30.74, 30.41, 28.24, 28.10, 28.02, 26.75, 24.29, 23.83, 22.83, 22.57, 21.08, 19.37, 19.35, 18.72, 17.66, 14.14, 14.11, 11.86. HRMS *m*/*z* calculated for C_55_H_84_O_11_N_3_: (M + H)^+^ 962.6100, found 962.6086.

#### 4.3.24 Ethyl *N*^5^-((3*S*,5*S*,7*S*)-Adamantan-1-yl)-*N*^2^-((*E*)-3-(4-hydroxy-3-methoxyphenyl)acryloyl)glycyl-l-valyl-d-glutaminate (**28**)

Synthesized from 1-adamantylamine (17 mg, 0.112 mmol) using General procedure C. The crude product was purified by column chromatography (MeOH/DCM 1:50) to give compound **28** as an off-white solid (20 mg, 43%). ^1^H NMR (400 MHz, DMSO-*d*_6_) δ 9.46 (s, 1H), 8.33 (d, *J* = 7.2 Hz, 1H), 8.22 (t, *J* = 5.8 Hz, 1H), 7.89 (d, *J* = 8.9 Hz, 1H), 7.34 (d, *J* = 15.7 Hz, 1H), 7.23 (s, 1H), 7.15 (s, 1H), 7.01 (d, *J* = 8.1 Hz, 1H), 6.79 (d, *J* = 8.1 Hz, 1H), 6.57 (d, *J* = 15.7 Hz, 1H), 4.26–4.04 (m, 4H), 3.90 (d, *J* = 5.7 Hz, 2H), 3.81 (s, 3H), 2.10–2.03 (m, 2H), 2.02–1.93 (m, 4H), 1.93–1.83 (m, 7H), 1.81–1.69 (m, 1H), 1.59 (s, 6H), 1.18 (t, *J* = 7.1 Hz, 3H), 0.85 (t, *J* = 6.8 Hz, 6H). ^13^C NMR (100 MHz, DMSO) δ 172.39, 171.45, 170.82, 169.60, 166.32, 148.83, 148.28, 139.97, 126.78, 122.10, 118.95, 116.10, 111.33, 60.91, 57.97, 55.98, 52.04, 51.05, 42.78, 41.40, 36.51, 35.67, 32.59, 31.20, 29.54, 29.25, 27.26, 19.69, 18.19, 14.53. HRMS *m*/*z* calculated for C_34_H_49_O_8_N_4_: 641.3545 (M + H)^+^, found 614.3529.

#### 4.3.25 Ethyl *N*^5^-(4-((*tert*-Butoxycarbonyl)amino)butyl)-*N*^2^-((*E*)-3-(4-hydroxy-3-methoxyphenyl)acryloyl)glycyl-l-valyl-d-glutaminate (**29**)

Synthesized from *tert*-butyl (4-aminobutyl)carbamate hydrochloride (29 mg, 0.127 mmol) using General procedure C. The crude product was purified by column chromatography (MeOH/DCM 1:30) to give compound **29** as an off-white solid (20 mg, 33%). ^1^H NMR (400 MHz, DMSO-*d*_6_) δ 9.51 (s, 1H), 8.47 (d, *J* = 7.3 Hz, 1H), 8.26 (t, *J* = 5.7 Hz, 1H), 7.94 (d, *J* = 9.0 Hz, 1H), 7.84 (t, *J* = 5.5 Hz, 1H), 7.40 (d, *J* = 15.7 Hz, 1H), 7.22 (d, *J* = 2.0 Hz, 1H), 7.12–7.04 (m, 1H), 6.90–6.79 (m, 2H), 6.63 (d, *J* = 15.7 Hz, 1H), 4.40–4.28 (m, 1H), 4.28–4.19 (m, 1H), 4.14 (qd, *J* = 7.1, 2.6 Hz, 2H), 3.96 (d, *J* = 5.8 Hz, 2H), 3.87 (s, 3H), 3.10–3.01 (m, 2H), 2.96–2.93 (m, 2H), 2.21–2.14 (m, 2H), 2.09–1.98 (m, 2H), 1.91–1.81 (m, 1H), 1.49–1.34 (m, 13H), 1.24 (t, *J* = 7.1 Hz, 3H), 0.91 (t, *J* = 7.2 Hz, 6H).

#### 4.3.26 Ethyl (*R*)-9,14-Bis(*tert*-butoxycarbonyl)-22-((*S*)-2-(2-((*E*)-3-(4-hydroxy-3-methoxyphenyl)acrylamido)acetamido)-3-methylbutanamido)-2,2-dimethyl-4,19-dioxo-3-oxa-5,9,14,18-tetraazatricosan-23-oate (**31**)

Synthesized from triboc-spermine (45 mg, 0.114 mmol) using General procedure C. The crude product was purified by column chromatography (MeOH/DCM 1:20) to give compound **31** as an off-white solid (29 mg, 39%). ^1^H NMR (400 MHz, DMSO-*d*_6_) δ 9.46 (s, 1H), 8.40 (d, *J* = 7.4 Hz, 1H), 8.20 (t, *J* = 5.9 Hz, 1H), 7.87 (d, *J* = 9.0 Hz, 1H), 7.83–7.69 (m, 1H), 7.34 (d, *J* = 15.6 Hz, 1H), 7.15 (d, *J* = 1.9 Hz, 1H), 7.01 (dd, *J* = 8.2, 1.9 Hz, 1H), 6.83–6.72 (m, 2H), 6.57 (d, *J* = 15.7 Hz, 1H), 4.32–4.23 (m, 1H), 4.23–4.13 (m, 1H), 4.13–3.99 (m, 2H), 3.90 (d, *J* = 5.7 Hz, 2H), 3.81 (s, 3H), 3.15–3.04 (m, 11H), 3.02–2.95 (m, 3H), 2.91–2.85 (m, 3H), 2.15–2.10 (m, 2H), 2.01–1.93 (m, 2H), 1.85–1.74 (m, 2H), 1.61–1.50 (m, 5H), 1.44–1.33 (m, 39H), 1.18 (t, *J* = 7.1 Hz, 3H), 0.85 (t, *J* = 7.2 Hz, 6H).

#### 4.3.27 Ethyl (*R*)-10-(*tert*-Butoxycarbonyl)-18-((*S*)-2-(2-((*E*)-3-(4-hydroxy-3-methoxyphenyl)acrylamido)acetamido)-3-methylbutanamido)-2,2-dimethyl-4,15-dioxo-3-oxa-5,10,14-triazanonadecan-19-oate (33)

Synthesized from N1,N4-Bis-Boc-spermidine (56 mg, 0.162 mmol) using General procedure C. The crude product was purified by column chromatography (MeOH/DCM 1:15) to give compound **33** as an off-white solid (47 mg, 42%). ^1^H NMR (400 MHz, DMSO-*d*_6_) δ 8.40 (d, *J* = 7.3 Hz, 1H), 8.25 (t, *J* = 5.8 Hz, 1H), 7.89 (d, *J* = 9.0 Hz, 1H), 7.79 (t, *J* = 5.6 Hz, 1H), 7.37 (d, *J* = 15.7 Hz, 1H), 7.22 (s, 1H), 7.10 (d, *J* = 1.1 Hz, 1H), 6.84–6.71 (m, 1H), 6.65 (d, *J* = 15.8 Hz, 1H), 4.30–4.13 (m, 2H), 4.13–3.96 (m, 2H), 3.91 (d, *J* = 5.9 Hz, 2H), 3.82 (s, 3H), 3.15–2.98 (m, 6H), 2.91–2.83 (m, 2H), 2.16–2.08 (m, 2H), 2.02–1.74 (m, 5H), 1.59–1.48 (m, 4H), 1.43–1.32 (m, 22H), 1.18 (t, *J* = 7.1 Hz, 3H), 0.85 (t, *J* = 7.0 Hz, 6H).

#### 4.3.28 Ethyl *N*^5^-(4-Aminobutyl)-*N*^2^-((*E*)-3-(4-hydroxy-3-methoxyphenyl)acryloyl)glycyl-l-valyl-d-glutaminate (**30**)

Synthesized from **29** (15 mg, 0.021 mmol) using General procedure D. An off-white solid (8 mg, 55%). ^1^H NMR (400 MHz, DMSO-*d*_6_) δ 9.48 (s, 1H), 8.40 (d, *J* = 7.4 Hz, 1H), 8.24 (t, *J* = 5.7 Hz, 1H), 7.96–7.77 (m, 2H), 7.71–7.53 (m, 3H), 7.34 (d, *J* = 15.7 Hz, 1H), 7.15 (d, *J* = 2.0 Hz, 1H), 7.01 (dd, *J* = 8.2, 2.0 Hz, 1H), 6.80 (d, *J* = 8.2 Hz, 1H), 6.57 (d, *J* = 15.7 Hz, 1H), 4.26–4.16 (m, 2H), 4.15–4.02 (m, 2H), 3.97–3.86 (m, 2H), 3.81 (s, 3H), 3.14–2.95 (m, 2H), 2.83–2.71 (m, 2H), 2.18–2.07 (m, 2H), 2.06–1.88 (m, 2H), 1.89–1.74 (m, 1H), 1.57–1.34 (m, 4H), 1.18 (t, *J* = 7.1 Hz, 3H), 0.86 (t, *J* = 6.5 Hz, 6H). HRMS *m*/*z* calculated for C_28_H_44_O_8_N_5_: (M + H)^+^ 578.3184, found 578.3186.

#### 4.3.29 *N*^5^-(3-((4-((3-Aminopropyl)amino)butyl)amino)propyl)-*N*^2^-((*E*)-3-(4-hydroxy-3-methoxyphenyl)acryloyl)glycyl-l-valyl-d-glutaminate (**32**)

Synthesized from **31** (25 mg, 0.025 mmol) using General procedure D. An off-white solid (15 mg, 75%). ^1^H NMR (400 MHz, DMSO-*d*_6_) δ 9.50 (s, 1H), 8.62 (s, 2H), 8.54–8.33 (m, 3H), 8.26 (t, *J* = 5.8 Hz, 1H), 7.99 (t, *J* = 5.8 Hz, 1H), 7.92–7.79 (m, 4H), 7.34 (d, *J* = 15.7 Hz, 1H), 7.15 (d, *J* = 2.0 Hz, 1H), 7.05–6.95 (m, 1H), 6.80 (d, *J* = 8.1 Hz, 1H), 6.57 (d, *J* = 15.7 Hz, 1H), 4.28–4.18 (m, 2H), 4.13–4.04 (m, 2H), 3.94–3.89 (m, 2H), 3.81 (s, 3H), 2.99–2.86 (m, 12H), 2.16 (t, *J* = 7.5 Hz, 2H), 2.06–1.96 (m, 2H), 1.92–1.80 (m, 4H), 1.73–1.68 (m, 1H), 1.64–1.58 (m, 4H), 1.18 (t, *J* = 7.1 Hz, 3H), 0.87 (t, *J* = 6.3 Hz, 6H). ^13^C NMR (100 MHz, DMSO) δ 172.64, 172.22, 172.04, 171.57, 169.60, 166.40, 159.63, 159.30, 158.97, 158.64, 148.91, 148.30, 147.83, 145.08, 139.96, 132.51, 126.75, 122.14, 120.64, 118.94, 118.56, 116.12, 115.73, 115.61, 112.82, 111.32, 60.95, 58.13, 58.06, 55.98, 55.93, 52.00, 46.59, 46.54, 45.08, 44.35, 40.57, 40.37, 40.16, 39.95, 39.74, 39.53, 39.32, 36.65, 36.13, 31.70, 31.14, 26.98, 26.48, 24.25, 23.14, 23.08, 19.62, 18.23, 18.20, 14.46. HRMS *m*/*z* calculated for C_34_H_58_O_8_N_7_: (M + H)^+^ 692.4341, found 692.4359.

#### 4.3.30 Ethyl *N*^5^-(3-((4-Aminobutyl)amino)propyl)-*N*^2^-((*E*)-3-(4-hydroxy-3-methoxyphenyl)acryloyl)glycyl-l-valyl-d-glutaminate (**34**)

Synthesized from **33** (47 mg, 0.057 mmol) using General procedure D. An off-white solid (28 mg, 67%). ^1^H NMR (400 MHz, DMSO-*d*_6_) δ 8.54–8.34 (m, 3H), 8.25 (t, *J* = 5.7 Hz, 1H), 7.91–7.80 (m, 4H), 7.34 (d, *J* = 15.7 Hz, 1H), 7.15 (d, *J* = 2.0 Hz, 1H), 7.06–6.96 (m, 1H), 6.80 (d, *J* = 8.1 Hz, 1H), 6.57 (d, *J* = 15.7 Hz, 1H), 4.26–4.16 (m, 2H), 4.13–4.01 (m, 2H), 3.90 (t, *J* = 4.8 Hz, 2H), 3.81 (s, 3H), 3.07–2.84 (m, 8H), 2.19–2.07 (m, 2H), 2.03–1.91 (m, 2H), 1.87–1.80 (m, 2H), 1.58–1.40 (m, 6H), 1.18 (t, *J* = 7.1 Hz, 3H), 0.86 (t, *J* = 6.4 Hz, 6H).*The OH proton peak is not observed.

#### 4.3.31 1-Ethyl 5-(2-Morpholinoethyl) ((*E*)-3-(4-hydroxy-3-methoxyphenyl)acryloyl)glycyl-l-valyl-d-glutamate (**35**)

To an ice-chilled stirring solution of **27** (110 mg, 0.186 mmol) in DMF (2 mL), 4-(2-hydroxyethyl)morpholine (20 μL, 0.169 mmol), DIPEA (147 μL, 0.845 mmol) and COMU (80 mg, 0.186 mmol) were added. The mixture was allowed to warm to room temperature and the stirring continued overnight after which reaction mixture was concentrated *in vacuo*. The crude product was purified by Isolera One flash chromatography (acetonitrile/0.1% TFA gradient from 20 to 100%) and column chromatography (DCM/MeOH 17:1) to give compound **35** as a white solid (10 mg, 10%). ^1^H NMR (400 MHz, Chloroform-*d*) δ 7.57 (d, *J* = 15.5 Hz, 1H), 7.11–7.04 (m, 1H), 7.04–6.97 (m, 2H), 6.91 (d, *J* = 8.2 Hz, 1H), 6.77–6.67 (m, 1H), 6.57–6.47 (m, 1H), 6.33 (d, *J* = 15.5 Hz, 1H), 4.60–4.50 (m, 1H), 4.41–4.34 (m, 1H), 4.25–4.05 (m, 6H), 3.93 (s, 3H), 3.70 (t, *J* = 4.7 Hz, 4H), 2.60 (t, *J* = 5.7 Hz, 2H), 2.53–2.45 (m, 4H), 2.41 (q, *J* = 7.1, 6.5 Hz, 2H), 2.31–2.19 (m, 2H), 2.08–1.99 (m, 1H), 1.27–1.24 (m, 3H), 0.96 (dd, *J* = 15.0, 6.8 Hz, 6H). *The OH proton peak is not observed.

#### 4.3.32 Diethyl ((*E*)-3-(4-Hydroxy-3,5-dimethoxyphenyl)acryloyl)glycyl-l-valyl-d-glutamate (**37**)

Compound **36** (200 mg, 0.440 mmol eq) was deprotected using General procedure D and coupled to 3,5-dimethoxy-4-hydroxycinnamic acid (108 mg, 0.480 mmol) using DIPEA (383 μL, 2.200 mmol), HOBt (65 mg, 0.480 mmol), DMAP (catalytic amount) and EDC × HCl (92 mg, 0.480 mmol) in DMF (4 mL). The mixture was allowed to warm to room temperature and the stirring continued overnight after which ethyl acetate (25 mL) was added and washed with 1 M HCl (2 × 10 mL), saturated NaHCO_3_ (2 × 10 mL), brine (30 mL), dried over anhydrous Na_2_SO_4_, concentrated *in vacuo*. The crude product was purified by column chromatography (MeOH/DCM 1:30) to give compound **37** as a white solid (113 mg, 57%). ^1^H NMR (400 MHz, Chloroform-*d*) δ 7.53 (d, *J* = 15.5 Hz, 1H), 7.31–7.27 (m, 1H), 7.13 (d, *J* = 8.7 Hz, 1H), 6.88 (t, *J* = 5.4 Hz, 1H), 6.78–6.73 (m, 2H), 6.37 (d, *J* = 15.5 Hz, 1H), 4.60–4.50 (m, 1H), 4.44–4.38 (m, 1H), 4.21–4.06 (m, 6H), 3.90 (s, 6H), 2.45–2.35 (m, 2H), 2.27–2.15 (m, 2H), 2.09–1.98 (m, 1H), 1.27–1.20 (m, 6H), 0.96 (dd, *J* = 11.7, 6.8 Hz, 6H). ^13^C NMR (100 MHz, CDCl_3_) δ 172.97, 171.72, 171.27, 169.62, 166.93, 147.19, 142.27, 136.80, 126.07, 117.53, 104.97, 77.35, 77.03, 76.71, 61.72, 60.83, 58.61, 56.32, 52.01, 43.66, 30.67, 30.41, 26.74, 19.34, 17.69, 14.14, 14.10. HRMS *m*/*z* calculated for C_27_H_40_O_10_N_3_: (M + H)^+^ 566.2708, found 566.2697.

### 4.4 Preparation and Evaluation of SLN and NLC

SLN loaded with NOD2 agonist **2** were prepared by the melt-emulsification method. The lipid (45 mg; Compritol 888 ATO, Gattefossé, France) was weighed into a centrifuge tube and 200 μL of adjuvant solution in ethanol (12.5 mg/mL) was added. The ethanol was then allowed to evaporate completely before the lipid was melted in a water bath and 2 mL of heated (85–90 °C) aqueous stabilizer solution (0.9%; w/w) was added to the melted lipid. Poloxamer 188 and polysorbate 80 in weight ratio 2:1 were used as stabilizers. The emulsion of melted lipid in stabilizer solution was sonicated by ultrasound probe (Ultrasonic processor, Cole Parmer Antylia Scientific, USA) for 150 s at 40% amplitude. After sonication, the resulting nanoemulsion was cooled in an ice bath to allow the lipid droplets to solidify and SLN to form. NLC loaded with NOD2 agonist **2** were prepared by the same method, except that 5% of the solid lipid was replaced by a liquid lipid (Miglyol 812, Sasol, Germany). The NOD2 agonist-free SLN and NLC were prepared by the same procedure without addition of adjuvant.

The average hydrodynamic size and particle size distribution of lipid nanoparticles were determined by photon correlation spectroscopy, and **2** content in the SLN and NLC dispersion was determined by high-performance liquid chromatography.

### 4.5 Mice

C57BL/6, OT I (C57BL/6-Tg(TcraTcrb)1100Mjb/J), and OT II (C57BL/6-Tg(TcraTcrb)425Cbn/Crl) mice were purchased from Jackson Laboratory (Bar Harbor, ME, USA) and bred at the University of Leiden (The Netherlands). The mice were kept under standard laboratory conditions, with food and water provided *ad libitum*. The mice were euthanized while sedated, by cervical dislocation. All animal work was performed according to the guidelines of the European Parliament Directive 2010/63 EU and the experimental work was approved by the Animal Ethics Committee of Leiden University. For culture conditions of BMDCs, see below.

### 4.6 Cell Cultures

#### 4.6.1 HEK-Blue NOD1 and NOD2 Cells

HEK-Blue NOD1 and NOD2 cells (Invivogen, San Diego, CA, USA) were cultured in Dulbecco’s modified Eagle’s medium (Sigma-Aldrich, St. Louis, MO, USA), following the manufacturer’s instructions. The medium was supplemented with 10% heat-inactivated fetal bovine serum (Gibco), 2 mM l-glutamine (Sigma-Aldrich), 100 U/mL penicillin (Sigma-Aldrich), 100 μg/mL streptomycin (Sigma-Aldrich), and 100 μg/mL normocin (Invivogen) for two passages. Subsequent passages were cultured in medium additionally supplemented with 30 μg/mL blasticidin (Invivogen) and 100 μg/mL zeocin (Invivogen). Cultures were maintained in a humidified atmosphere at 37 °C with 5% CO_2_.

#### 4.6.2 Peripheral Blood Mononuclear Cells

PBMCs from healthy and consenting donors were obtained from heparinized blood through density gradient centrifugation using Ficoll-Paque (Pharmacia, Sweden). Following isolation, the cells were suspended in RPMI 1640 medium (Sigma-Aldrich, St. Louis, MO, USA) supplemented with 10% heat-inactivated fetal bovine serum (Gibco), 2 mM l-glutamine (Sigma-Aldrich), 100 U/mL penicillin (Sigma-Aldrich), and 100 μg/mL streptomycin (Sigma-Aldrich) for subsequent assays.

#### 4.6.3 Cancer Cell Line

The K562 cell line, derived from chronic myelogenous leukemia^104^ (ATCC, Manassas, VA, USA), was maintained in RPMI 1640 medium (Sigma-Aldrich, St. Louis, MO, USA) supplemented with 10% heat-inactivated fetal bovine serum (Gibco), 2 mM l-glutamine (Sigma-Aldrich), 100 U/mL penicillin (Sigma-Aldrich), and 100 μg/mL streptomycin (Sigma-Aldrich).

#### 4.6.4 Bone-Marrow-Derived Dendritic Cells

Bone marrow cells were extracted from the tibia of C57BL/6 mice and cultured in Dulbecco’s modified Eagle’s medium (Lonza, Basel, Switzerland), supplemented with 10% heat-inactivated fetal bovine serum (Lonza), 2 mM l-glutamine (Lonza), 100 U/mL penicillin (Lonza), 100 μg/mL streptomycin (Lonza), and 20 ng/mL granulocyte-macrophage colony-stimulating factor (ImmunoTools, Friesoythe, Germany) for a duration of 7 days at 37 °C with 5% CO_2_. The purity of the BMDCs was assessed using PE-labeled antimouse CD11c (Biolegend, San Diego, CA, USA) through flow cytometry, revealing over 90% of cells to be positive for CD11c.

### 4.7 Cytotoxicity

The compounds under investigation were dissolved in DMSO and subsequently diluted in culture medium to attain the desired final concentrations, ensuring that the DMSO concentration did not exceed 0.1%. HEK-Blue NOD2 cells were plated at a density of 40,000 cells per well in 96-well plates containing 100 μL of culture medium. Cells were then treated with 20 μM of each compound or with the corresponding vehicle (0.1% DMSO; control cells). Following an 18 h incubation period at 37 °C with 5% CO_2_, the metabolic activity was evaluated using the CellTiter 96 Aqueous One Solution cell proliferation assay (Promega, Madison, WI, USA), following the manufacturer’s protocol. Each experiment was conducted in duplicate and repeated as two independent biological replicates.

### 4.8 Measurement of NF-kB Transcriptional Activity (HEK-Blue Detection)

The HEK-Blue NOD1 and NOD2 cell line reporter assay was generated from HEK293 cells through cotransfection of the hNOD1 or hNOD2 gene, respectively, along with an NF-κB-inducible secreted embryonic alkaline phosphatase (SEAP) reporter gene. Upon activation of NOD1 or NOD2, NF-κB triggers the production of SEAP, which can be quantitatively measured colorimetrically. HEK-Blue NOD1 and NOD2 cells were seeded at a density of 2.5 × 10^5^ cells/mL in 96-well plates containing 200 μL of HEK-Blue detection medium (Invivogen, San Diego, CA, USA) and exposed to the compounds (7–8 different concentrations ranging from 1 nM to 20 μM for EC_50_ determination) or the corresponding vehicle (0.1% DMSO). After 18 h of incubation at 37 °C with 5% CO_2_, SEAP activity was measured spectrophotometrically by absorbance at 630 nm (BioTek Synergy microplate reader; Winooski, VT, USA). EC_50_ values were determined using Prism software (version 9; GraphPad Software, CA, USA). Each experiment was conducted in duplicate and repeated in at least two independent biological replicates.

### 4.9 Cytokine Release from Peripheral Blood Mononuclear Cells

Peripheral blood mononuclear cells were plated at a density of 1 × 10^6^ cells/mL in 48-well plates containing 500 μL of growth medium. Cells were then treated with the compounds (2 μM) or the corresponding vehicle (0.1% DMSO), both in the absence and presence of LPS (10 ng/mL). Following an 18 h incubation period at 37 °C with 5% CO_2_, cell-free supernatants were collected and stored at −80 °C until analysis. Cytokine production was quantified using the LEGENDplex Human Essential Immune Response Panel (Biolegend) (comprising IL-4, IL-2, CXCL10 (IP-10), IL-1β, TNF-α, CCL2 (MCP-1), IL-17A, IL-6, IL-10, IFN-γ, IL-12p70, CXCL8 (IL-8), TGF-β1) on an Attune NxT flow cytometer (Thermo Fisher Scientific, Waltham, MA, USA). Standard curves were constructed using recombinant cytokines provided in the kit. Data analysis was performed using Biolegend software Qognit and Prism (GraphPad, San Diego, CA, USA) software. The experiments were independently replicated four times. Statistical significance was determined using two-way analysis of variance (ANOVA) followed by Tukey’s multiple comparisons test.

### 4.10 Peripheral Blood Mononuclear Cell Cytotoxicity

The PBMC cytotoxicity assays utilizing K562 cells were conducted following previously established methods, with some adjustments.^82^ PBMCs were plated at a density of 4 × 10^5^ cells per well in 96-well plates and treated with compounds (10 μM), in the presence or absence of LPS (1 μg/mL), IL-2 (200 U/mL), or the corresponding vehicle (0.1% DMSO) for 18 h. Meanwhile, K562 cells were stained with CFSE (Invitrogen, Carlsbad, CA, USA), washed twice with complete medium, and then added (1 × 10^4^ cells per well) to the pretreated PBMCs to achieve a final effector cell to target tumor cell ratio of 40:1. Following a 4-h coincubation period at 37 °C with 5% CO_2_, cells were stained with Sytox blue dead cell stain (Invitrogen) and analyzed using an Attune NxT flow cytometer (Thermo Fisher Scientific, Waltham, MA, USA) along with FlowJo software (Tree Star, Inc., Ashland, OR, USA). Cells positive for both CFSE and Sytox blue were designated as dead K562 cells. PBMCs alone and CFSE-labeled cancer cells alone were also exposed to the compounds at equivalent concentrations and stained with Sytox blue to assess any direct cytotoxic effects of the compounds on PBMCs and cancer cells separately. Each experiment was conducted in duplicate and repeated in three independent biological replicates. Statistical analysis was performed using one-way ANOVA followed by Dunnett’s multiple comparisons test.

### 4.11 Bone-Marrow-Derived Dendritic Cell Antigen Presentation

CD4^+^ and CD8^+^ T cells were isolated from splenocytes of OT II and OT I transgenic mice using CD4^+^ and CD8^+^ T cell isolation kits (Miltenyi Biotec, Germany) following the manufacturer’s instructions. The purified T cells were labeled with CFSE (Invitrogen, Carlsbad, CA, USA) and subsequently washed. Next, 5 × 10^4^ T cells were combined with 1 × 10^4^ BMDCs/well (pretreated with desmuramylpeptides at 10 μM and 50 μg/mL ovalbumin [Invivogen, San Diego, CA, USA] for 18 h and then washed). Following a 72-h incubation period at 37 °C with 5% CO_2_, supernatants were harvested and stored at −80 °C for cytokine analysis. The cells were then stained with Fixable viability dye eFluor 780 (eBioscience, Thermo Fisher Scientific, MA, USA), anti-Thy1.2-PE-Cy7 antibodies (Biolegend, San Diego, CA, USA), anti-CD8-eFluor450 antibodies (eBioscience), anti-CD4-eFluor450 antibodies (eBioscience), and anti-CD25-APC antibodies (Biolegend) before analysis using a Beckman Coulter Cytoflex S flow cytometer (CA, USA) and FlowJo software (Tree Star, Inc., Ashland, OR, USA). Live Thy1.2+/CD4+ and Thy1.2+/CD8+ cells were assessed for CFSE dilution and CD25 expression. Each experiment was performed in duplicate and repeated in two independent biological replicates. Statistical significance was determined using paired *t* tests.

### 4.12 T Cell Cytokine Release

Following 72 h of coincubation with BMDCs (pretreated with desmuramylpeptides [10 μM] and washed), supernatants from CD4^+^ and CD8^+^ T cells were harvested as previously described. Cytokine concentrations were assessed using the Cytometric Bead Array Mouse Th1/Th2/Th17 Cytokine Kit (including IL-2, IL-4, IL-6, IFN-γ, TNF, IL-17A, IL-10; BD Bioscience) on an Attune NxT flow cytometer (Thermo Fisher Scientific, Waltham, MA, USA). Standard curves were constructed using recombinant cytokines provided in the kit. Data analysis was conducted using FlowJo (Tree Star, Inc., Ashland, OR, USA) and Prism (GraphPad, San Diego, CA, USA) software. Each experiment was performed in duplicate and replicated in two independent biological runs.

### 4.13 Monocyte Conversion Assay

The monocyte conversion assay was conducted following previously established methods, with some adjustments.^18^ Peripheral blood mononuclear cells were plated at a density of 3 × 10^6^ cells/mL in 24-well plates containing 700 μL of growth medium. Cells were then treated with MDP and desmuramylpeptides (10 μM) or the corresponding vehicle (0.1% DMSO). Following an incubation period at 37 °C with 5% CO_2_, cells were transferred to 1.5 mL tube using phosphate-buffered saline (PBS) and scraper. The cells were blocked with human TruStain Fcx (BioLegend) and stained using antihuman CD16 antibodies/Alexa Fluor 488 (BioLegend); antihuman CD14 antibodies/PE-Cy7 (BioLegend); antihuman HLA-DR antibodies/APC-Fire750 (BioLegend) and blocked with TrueStain Monocyte Blocker. A viability dye Helix NP Blue (BioLegend) was added to the previous panel to discriminate live cells. PBMC were analyzed using an Attune NxT flow cytometer (Thermo Fisher Scientific, Waltham, MA, USA) and the FlowJo software (Tree Star, Inc., Ashland, OR, USA). Initially, lymphocytes (CD14^–^, CD16^–^) and NK (CD16^–^, HLA-DR^–^) were eliminated, after which classical, intermediate and nonclassical monocytes were specifically identified by selective gating strategy as follows: CD14^+^ CD16^–^ (classical), CD14^+^ CD16^+^ (intermediate) and CD14^–^ CD16^+^ (nonclassical). Each experiment was conducted in duplicate and repeated in three independent biological replicates.

### 4.14 Screening against PAINS

All compounds underwent screening against the PAINS filter^105^ conducted using CANVAS software (Schrödinger, Release 2021-2, New York, USA), and successfully passed the filter.

### 4.15 Statistics

The data were analyzed using Prism software (version 9; GraphPad Software, CA, USA). Statistical significance was determined according to the specific procedures outlined in each experiment. A *p*-value <0.05 was considered statistically significant.

## Supporting Information Available

Supporting Figures and Tables; ^1^H and ^13^C NMR spectra of tested compounds; COSY spectra of representative compounds; HRMS spectra of tested compounds; HPLC traces of representative compounds (PDF)
SMILES_NOD2 (CSV)

## Abbreviations Used

BMDC: bone-marrow-derived dendritic cell
CARD: caspase recruitment domain
CFSE: carboxyfluorescein succinimidyl ester
COMU: (1-cyano-2-ethoxy-2-oxoethylidenaminooxy)dimethylamino-morpholino-carbenium hexafluorophosphate
DBU: 1,8-diazabicyclo[5.4.0]undec-7-ene
DC: dendritic cell
DIPEA: *N,N*-diisopropylethylamine
DMAP: 4-dimethylaminopyridine
DMF: dimethylformamide
EDC: 1-ethyl-3-(3-(dimethylamino)propyl)carbodiimide
EPA: eicosapentaenoic acid
EtOAc: ethyl acetate
HOBt: 1-hydroxybenzotriazole
IFN: interferon
IL: interleukin
IP-10: interferon γ-induced protein 10
MCP-1: monocyte chemoattractant protein-1
LPS: lipopolysaccharide
MDP: muramyl dipeptide
MurNAc: *N*-acetylmuramic acid
MTS: (3-(4,5-dimethylthiazol-2-yl)-5-(3-carboxymethoxyphenyl)-2-(4-sulfophenyl)-2*H*-tetrazolium)
NF-κB: nuclear factor κB
NK: natural killer
NLC: nanostructured lipid carriers
NOD: nucleotide-binding oligomerization domain
PBMC: peripheral blood mononuclear cell
PRR: pattern recognition receptor
PUFA: poly unsaturated fatty acids
SAR: structure–activity relationship
SEAP: secreted embryonic alkaline phosphatase
SLN: solid lipid nanoparticles
TFA: trifluoroacetic acid
TGF: transforming growth factor
THF: tetrahydrofuran
TLR: Toll-like receptor
TNF: tumor necrosis factor
