# Supplementary material for: Distinctive Immune Signatures Driven by Structural Alterations in Desmuramylpeptide NOD2 Agonists
Source: J Med Chem. 2024 Sep 30;67(19):17585–607. doi: 10.1021/acs.jmedchem.4c01577 (PMC11472310; doi:10.1021/acs.jmedchem.4c01577)
Supplement: Supplementary file 1 — jm4c01577_si_001.pdf [file jm4c01577_si_001.pdf]

Supporting information

Distinctive Immune Signatures Driven by Structural Alterations in Desmuramylpeptide NOD2 Agonists

Špela Janež,[1] Samo Guzelj,[1] Petra Kocbek,[1] Eveline A. de Vlieger,[2] Bram Slütter,[2] Žiga Jakopin[*,1]

[1]Faculty of Pharmacy, University of Ljubljana, SI-1000 Ljubljana, Slovenia

[2]Div. BioTherapeutics, Leiden Academic Centre for Drug Research, Leiden University, 2333 CC Leiden, The Netherlands

*Corresponding Author

Žiga Jakopin

Phone: +386 1 4769 646

Fax: +386 1 4258 031

E-mail: ziga.jakopin@ffa.uni-lj.si

Table of contents

1. Supporting figures S2
2. Supporting tables S3
3. 1H and 13C NMR spectra of tested compounds S7
4. COSY spectra of representative compounds S33
5. HRMS spectra of tested compounds S37
6. Representative UHPLC traces S63

1. Supporting figures

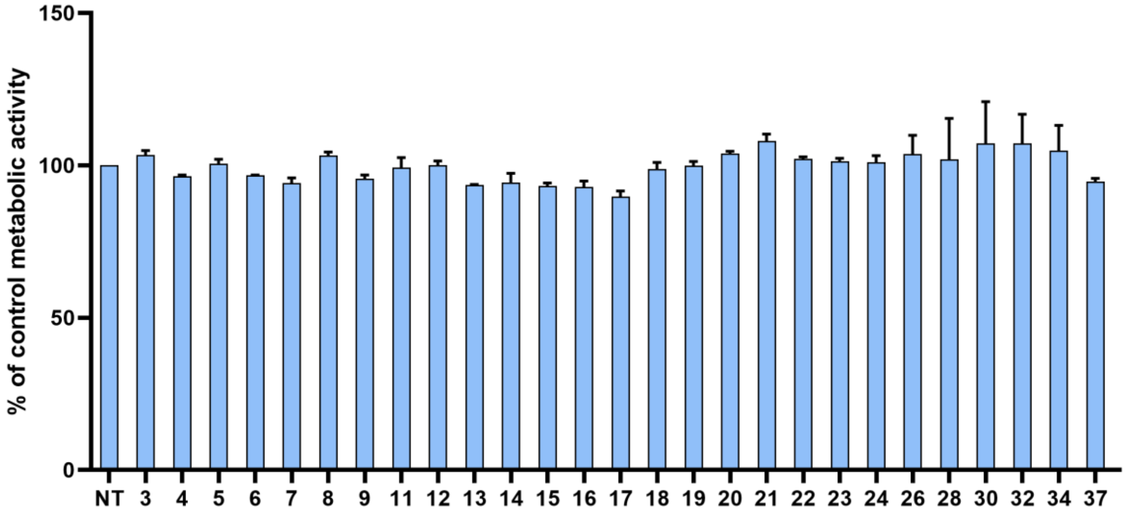

Figure S1: Metabolic activities of HEK-Blue NOD2 cells after 18 h stimulation with MDP (10 µM) or the desmuramylpeptides (10 µM). The results are shown relative to that of the untreated control (0.1 % DMSO; NT). Data are means ±SEM of two independent experiments.

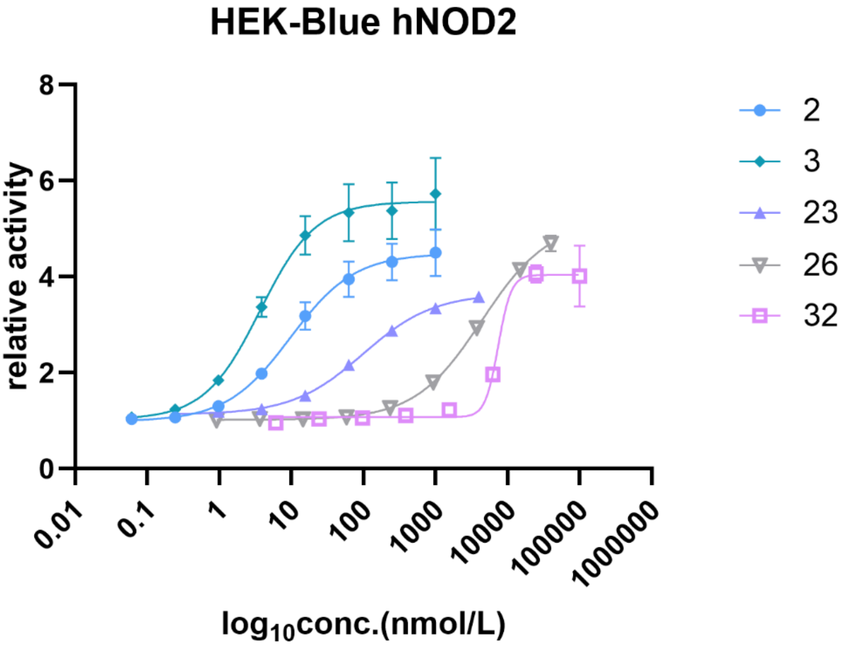

Figure S2: Dose-response curves for compounds **2**, **3**, **23, 26** and **32** on HEK-blue NOD2 cell line. The results are shown as relative activity to untreated control (0.1 % DMSO; NT). Data are means ±SEM of three independent experiments.

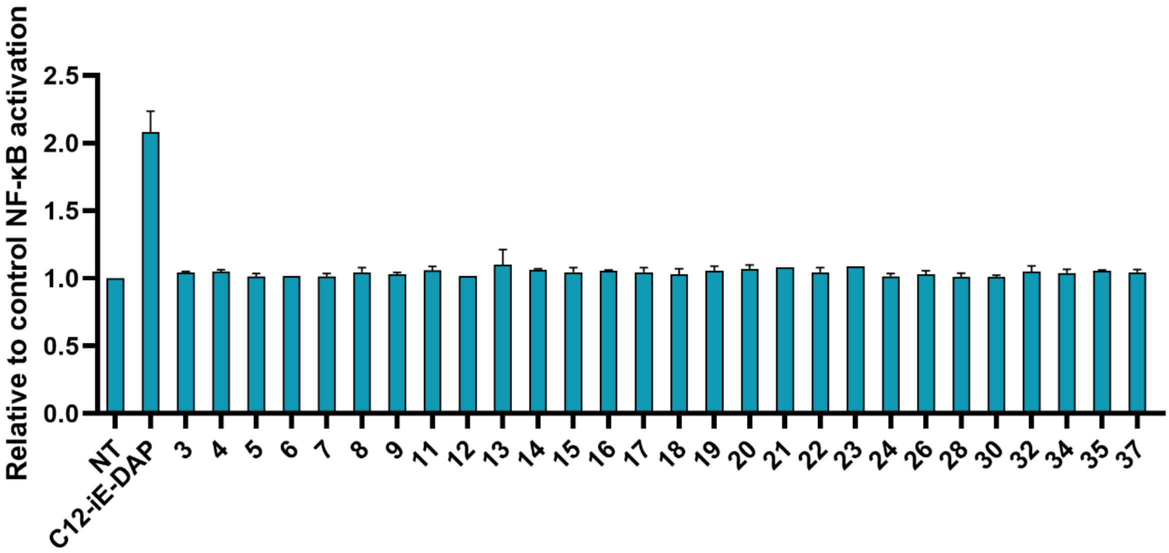

Figure S3: NOD1 agonistic activities of desmuramypeptides. HEK-Blue NOD1 cells were treated with C12-iE-DAP (1 µM) or the desmuramypeptides (20 µM) for 18 h. The SEAP activities are shown relative to that of the untreated control (0.1 % DMSO; NT). Data are means ±SEM of two independent experiments. C12-iE-DAP was used as the positive control.

2. Supporting tables

Table S1: Cytokine concentrations measured after 18 h stimulation of PBMCs with MDP (2 µM) or the desmuramylpeptides (2 µM) in the absence or presence of LPS (10 ng/mL). Data are mean ± SEM of two* or three independent experiments.

Compound	IL-4 (pg/mL)	IL-2 (pg/mL)	IP-10 (pg/mL)	IL-1β (pg/mL)	TNF-α (pg/mL)	MCP-1 (pg/mL)	IL-17A (pg/mL)	IL-6 (pg/mL)	IL-10 (pg/mL)	IFN-γ (pg/mL)	IL-12p70 (pg/mL)	IL-8 (pg/mL)	TGF-β1 (pg/mL)
**Control***	0.3 ± 0.1	0.3 ± 0.2	18.2 ± 15.3	0 ± 0	0.2 ± 0	14.5 ± 7.2	0 ± 0	0.9 ± 0.2	0.3 ± 0.1	0 ± 0	0 ± 0	8.2 ± 0.1	0 ± 0
**MDP***	0.4 ± 0.1	0.5 ± 0.1	3.4 ± 0.5	11.4 ± 0	13.3 ± 0.1	34.3 ± 4.4	0 ± 0	199.4 ± 5	0.4 ± 0	0 ± 0	0.1 ± 0	697.5 ± 54	0 ± 0
**2***	0.6 ± 0	0.5 ± 0.1	10.3 ± 3.3	4.1 ± 2.1	6.7 ± 1.9	81.7 ± 9.9	0.2 ± 0.2	163.9 ± 42.5	0.3 ± 0.2	0.2 ± 0.2	0.1 ± 0.1	430.6 ± 178.2	0 ± 0
**3***	0.6 ± 0	0.3 ± 0	8.8 ± 4.9	6.3 ± 1.3	5.7 ± 0.2	28.1 ± 2.8	0 ± 0	166.3 ± 15.8	0.3 ± 0	0 ± 0	0 ± 0	278.7 ± 10	0 ± 0
**23***	0.6 ± 0.2	0.4 ± 0.1	3.9 ± 0.5	3.9 ± 0.2	5.2 ± 0	51.8 ± 7.6	0 ± 0	191.9 ± 10.6	0.9 ± 0.6	0 ± 0	0.1 ± 0	497.1 ± 42.1	0 ± 0
**26***	0.4 ± 0.1	0.3 ± 0	5.5 ± 0.8	0 ± 0	2.9 ± 0.2	10.9 ± 1.2	0.3 ± 0.3	46.3 ± 3.7	0.2 ± 0.2	0 ± 0	0 ± 0	216.6 ± 3.1	0 ± 0
**28***	0.4 ± 0.1	0.2 ± 0	2.5 ± 0.2	0 ± 0	0.3 ± 0	3.8 ± 1	0 ± 0	2.3 ± 0.2	0.2 ± 0.2	0 ± 0	0 ± 0	8.1 ± 1.4	0 ± 0
**32***	0.1 ± 0	0.1 ± 0.1	9.4 ± 6.4	15.8 ± 15.8	48.8 ± 48.6	137.8 ± 130.6	0 ± 0	1654 ± 1652.9	3.7 ± 3.3	8.8 ± 8.8	3.5 ± 3.5	1203.9 ± 1195.6	0 ± 0
**Control + LPS**	1.4 ± 0	2 ± 0.4	67.3 ± 15.9	331.2 ± 11.9	754.2 ± 44.6	4393.3 ± 386.4	1.1 ± 0.4	25954.3 ± 1510.7	128.1 ± 23.2	40 ± 1.5	19.6 ± 3.2	3170.7 ± 1790.3	0 ± 0
**MDP + LPS**	1.4 ± 0.1	0.6 ± 0	122.3 ± 11.6	1280 ± 3.1	2843.3 ± 155.1	1279.7 ± 92	0.4 ± 0.2	45007.4 ± 1637.1	181.7 ± 1	393.8 ± 5.3	68.7 ± 4.6	3901.4 ± 9.5	0 ± 0
**2 + LPS**	1.5 ± 0.1	0.7 ± 0.1	82.3 ± 8.4	2640.1 ± 142.6	2765.1 ± 305.2	596.2 ± 62.2	0.4 ± 0.1	53410.5 ± 380.4	196.4 ± 9.1	173 ± 24.7	30.2 ± 3.1	6127.5 ± 129.4	2.7 ± 0.4
**3 + LPS**	1.5 ± 0.1	0.5 ± 0.1	64.1 ± 4.3	2616.7 ± 114.4	1547.2 ± 32.9	381.5 ± 27.4	0.8 ± 0.4	48874.5 ± 1063.3	216.7 ± 5.2	93.6 ± 11	23.8 ± 2.7	2405.4 ± 244.9	2.4 ± 0.1
**23 + LPS**	1.7 ± 0.2	0.5 ± 0	75.1 ± 11.5	3399.9 ± 101.7	1932 ± 110.9	403.1 ± 89.2	0.3 ± 0	52364.2 ± 596.2	249 ± 17.1	183.4 ± 12.3	34.5 ± 0.6	4830.3 ± 452.4	3.4 ± 0.3
**26 + LPS**	1.4 ± 0.1	0.6 ± 0	116.6 ± 10.8	2069.4 ± 121.2	3915.9 ± 188.7	735.5 ± 117.2	0.8 ± 0.2	51802.8 ± 615.4	185.6 ± 11.4	363.1 ± 18.4	89.3 ± 15.1	6066.9 ± 1200.8	1.8 ± 0
**28 + LPS**	1.1 ± 0.1	0.7 ± 0.1	58.6 ± 15.8	453.4 ± 31.5	1102.1 ± 97.4	1443.8 ± 268.4	0.3 ± 0	36409.4 ± 3306.8	78.8 ± 7	75.1 ± 17.6	35.2 ± 7	7731.1 ± 2399.4	0 ± 0
**32 + LPS**	1.2 ± 0	0.6 ± 0.1	69.6 ± 6.5	664.6 ± 5.9	1770.3 ± 86.2	1019.7 ± 140.5	0.9 ± 0.4	39954 ± 514.1	94.3 ± 5.7	126.6 ± 2.7	61.7 ± 6.7	5546.8 ± 2023.4	0 ± 0

Table S2: Relative cytotoxicity of compounds and percentage of dead PBCMs. Data are means ± SEM of duplicates of three independent experiments.

	**Cytotoxicity (ratio to control)**	**Percent of dead PBMCs**
**Control**	1.0 ± 0.0	0.78% ± 0.04%
**Control + LPS**	1.8 ± 0.08	4.16% ± 0.47
**MDP**	1.17 ± 0.07	1.11% ± 0.15%
**MDP+LPS**	2.71 ± 0.16	7.95% ± 0.95%
**2**	1.00 ± 0.05	0.96% ± 0.14%
**2 + LPS**	1.54 ± 0.08	8.07% ± 0.47%
**3**	1.04 ± 0.06	1.14% ± 0.23%
**3 + LPS**	0.98 ± 0.18	3.34% ± 0.35%
**23**	1.02 ± 0.08	1.28% ± 0.06%
**23 + LPS**	1.30 ± 0.26	11.4% ± 2.64%
**26**	1.34 ± 0.05	1.08% ± 0.10%
**26 + LPS**	2.39 ± 0.27	9.93% ± 0.49%
**28**	0.91 ± 0.02	0.99% ± 0.21%
**28 + LPS**	2.19 ± 0.07	10.44% ± 0.63%
**32**	1.10 ± 0.06	1.01% ± 0.22%
**32 + LPS**	2.95 ± 0.41	7.20% ± 0.36%
**IL-2**	4.24 ± 1.28	1.06% ± 0.27%

Table S3: Cytokine concentrations in co-culture supernatants. Data are means ± SEM of duplicates of two independent experiments.

**IL-13 (pg/mL)**	16 ± 11.7	54.5 ± 14.7	13.7 ± 3.7	12.6 ± 3.4	15 ± 8.4	13.6 ± 5.2	0.5 ± 0	8.7 ± 3.7	0.5 ± 0.1	0.6 ± 0.1	0.6 ± 0.1	0.6 ± 0.1
**IL-22 (pg/mL)**	194 ± 171.8	2851.4 ± 1526.8	134 ± 78.7	195.5 ± 150.1	296.2 ± 200.9	217.8 ± 131.1	0.5 ± 0.1	815.8 ± 479.8	0.7 ± 0.2	0.6 ± 0.1	0.8 ± 0.3	0.8 ± 0.3
**IL-17F (pg/mL)**	7.8 ± 6.7	186.8 ± 172.5	2.3 ± 1.1	4.3 ± 2.9	7.7 ± 6.1	3 ± 1.8	0.9 ± 0	103.6 ± 99.5	1 ± 0	1 ± 0	1 ± 0.1	1 ± 0.1
**IL-17A (pg/mL)**	49.9 ± 48.7	725.6 ± 682.6	24.1 ± 16.2	42.2 ± 38.8	37.8 ± 32.8	43.1 ± 32.2	0.6 ± 0.2	399.7 ± 393.2	1 ± 0.5	0.7 ± 0.3	0.8 ± 0.3	0.8 ± 0.3
**IL-9 (pg/mL)**	473.4 ± 208.2	2067.8 ± 141.5	629.1 ± 159.1	907.4 ± 274.2	808.8 ± 323.6	806 ± 235	4.2 ± 0.3	146.4 ± 19.6	14.5 ± 6.5	20.9 ± 7.4	16.5 ± 5.3	14.2 ± 6.3
**IL-10 (pg/mL)**	4.2 ± 2.4	55.4 ± 10.5	4.4 ± 1.6	3.2 ± 0.8	3.5 ± 1.6	5.1 ± 1.8	1.8 ± 0.6	45.2 ± 6.5	2.5 ± 2.1	1.2 ± 0.6	3.1 ± 2.1	3.5 ± 2.2

	Compound	IFN-γ (pg/mL)	IL-5 (pg/mL)	TNF-α (pg/mL)	IL-2 (pg/mL)	IL-6 (pg/mL)	IL-4 (pg/mL)
$CD4^+$ T cells	**NT**	191 ± 119.6	8.9 ± 7.3	88 ± 26	1255.2 ± 317.3	136.3 ± 20.9	10.5 ± 3.7
	**LPS**	1558.6 ± 604.2	25.9 ± 20.8	392.8 ± 136.8	4695.5 ± 437.4	26654.6 ± 2397.3	7.3 ± 0.5
	**3**	227.8 ± 42.1	5.5 ± 3.4	113.8 ± 12	4061.8 ± 817.2	179.3 ± 38.6	18.4 ± 3.2
	**23**	146.3 ± 63.6	4.8 ± 2.8	126.7 ± 37	3834.8 ± 467.4	177.9 ± 77.8	9.6 ± 1.9
	**26**	85.6 ± 31.5	3.5 ± 2.7	86.8 ± 19.1	1435 ± 246.5	179.7 ± 55.3	3.5 ± 1.2
	**28**	216.3 ± 66.8	4.1 ± 1.7	130.1 ± 27	2019.5 ± 275.9	273.4 ± 61.4	12.3 ± 4
$CD8^+$ T cells	**NT**	31.8 ± 8.4	0.2 ± 0	22.2 ± 4	369.5 ± 38.2	29.2 ± 7.4	0 ± 0
	**LPS**	3238.9 ± 720.5	1,8 ± 0,5	308.5 ± 38.2	67 ± 11.2	21930.5 ± 2561.5	4.3 ± 0.4
	**3**	67.5 ± 26.4	0,3 ± 0,1	31.1 ± 5.3	463.8 ± 36.6	64.9 ± 22.6	0 ± 0
	**23**	90.9 ± 11.9	0.3 ± 0	31.5 ± 5.8	518.5 ± 70.3	35 ± 19.6	0 ± 0
	**26**	102.6 ± 34.6	0.3 ± 0.1	39.3 ± 10.1	444.9 ± 86.1	56.1 ± 27.6	0.1 ± 0.1
	**28**	68 ± 37	0.3 ± 0.1	31.1 ± 13	742.1 ± 238.7	71.6 ± 30.5	0.1 ± 0.1

Table S4: Average particle size (d), polydispersity index (PI) and zeta potential (ZP) of SLN and NLC loaded with NOD2 agonist **2** and concentration of NOD2 agonist **2** in nanoparticle dispersion.

	d [nm]	PI	ZP [mV]	c (**2**) [µg/mL]
SLN	154.1 ± 1.9	0.21 ± 0.02	-18.0 ± 3.1	74.2
NLC	182.1 ± 6.6	0.29 ± 0.04	-20.7 ± 1.9	81.5

3. 1H and 13C NMR spectra of tested compounds

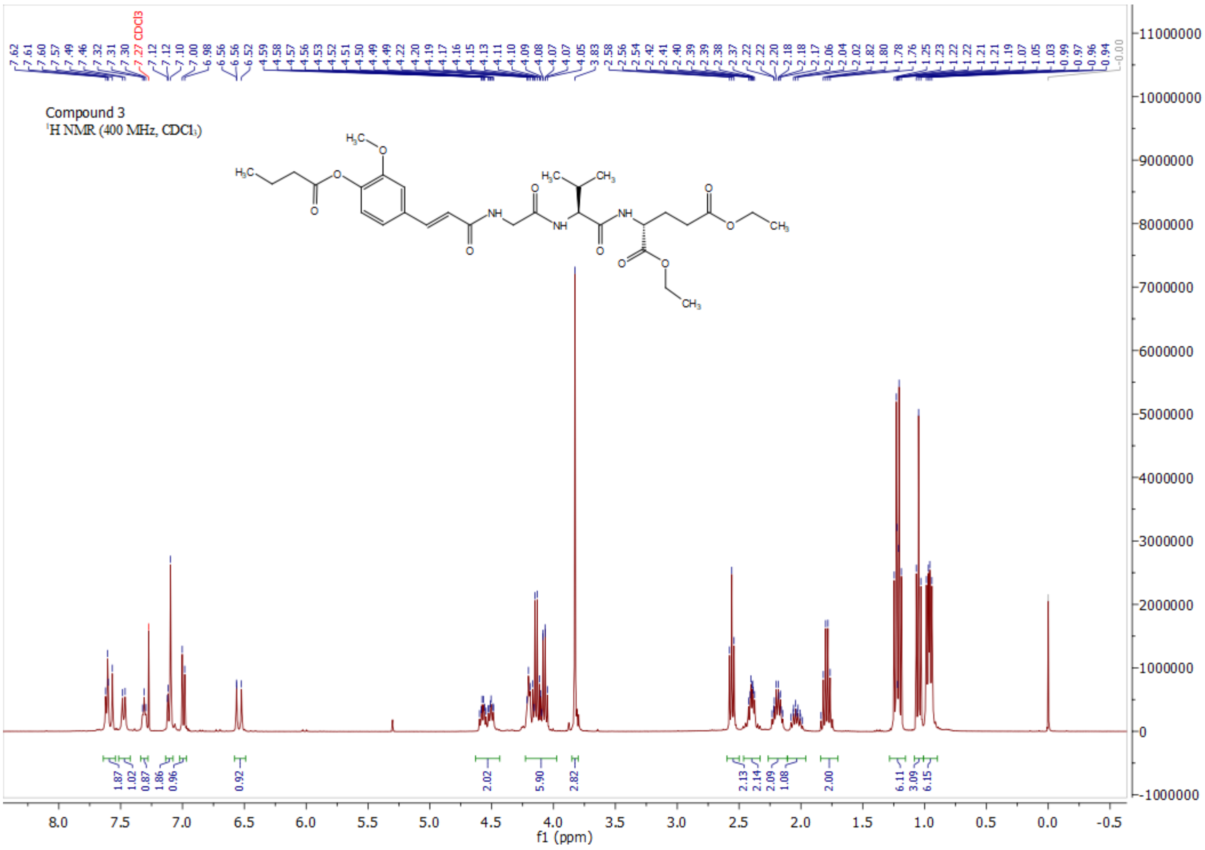

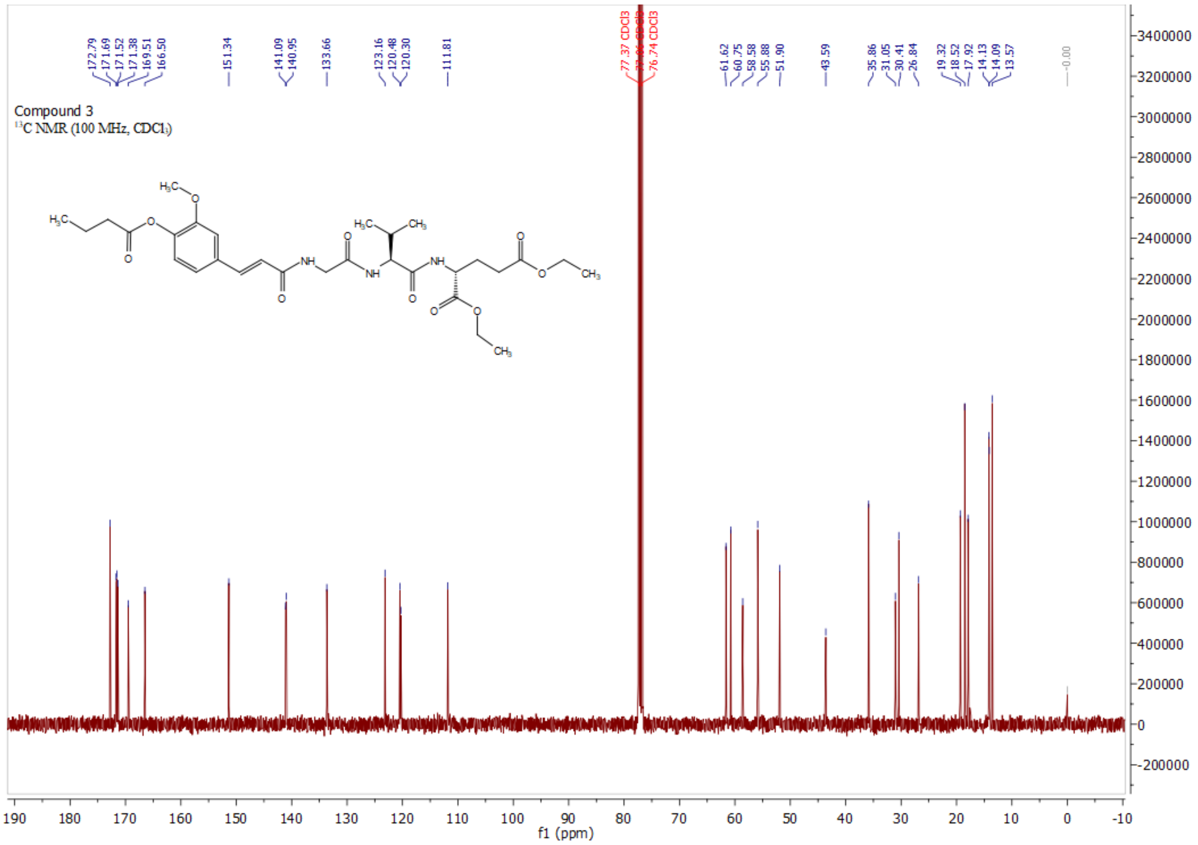
Compound 3
^{13}C NMR (100 MHz, $CDCl_3$)
f1 (ppm)

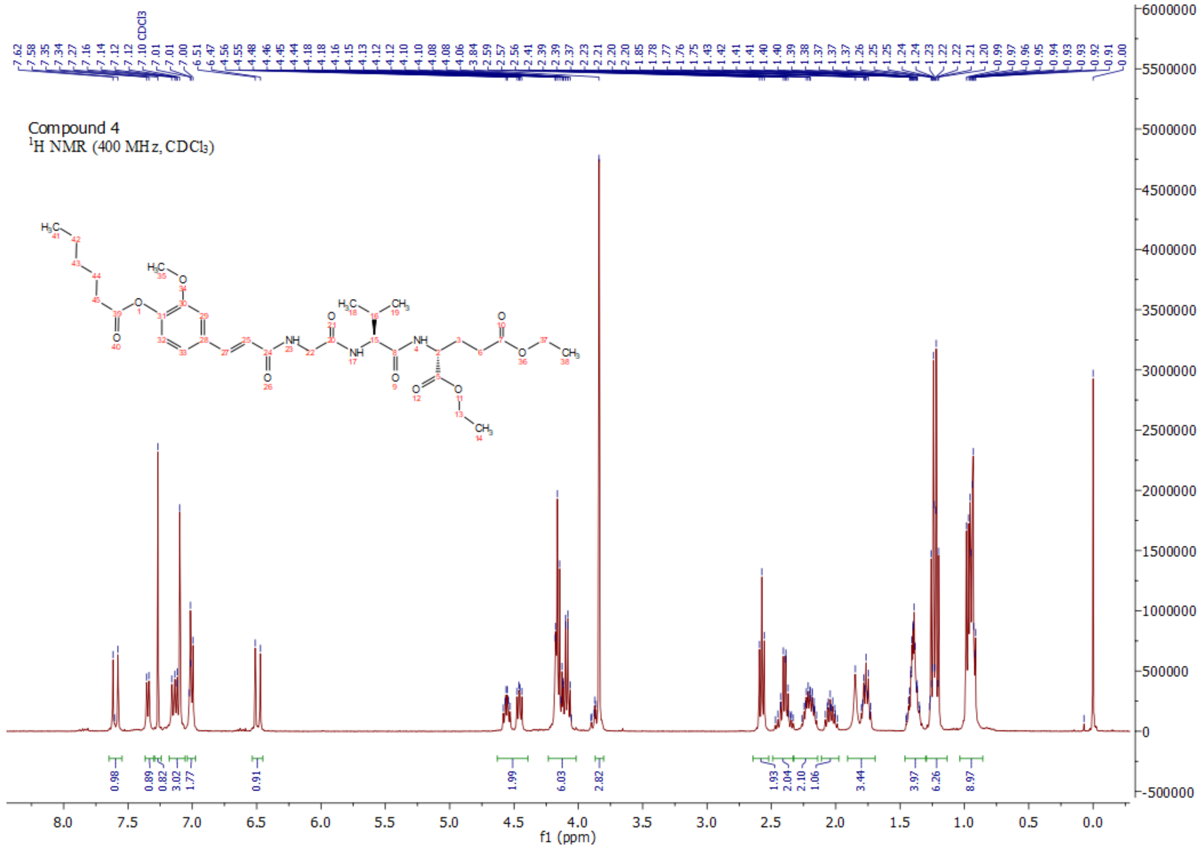
Compound 4
^{1}H NMR (400 MHz, $CDCl_3$)
f1 (ppm)

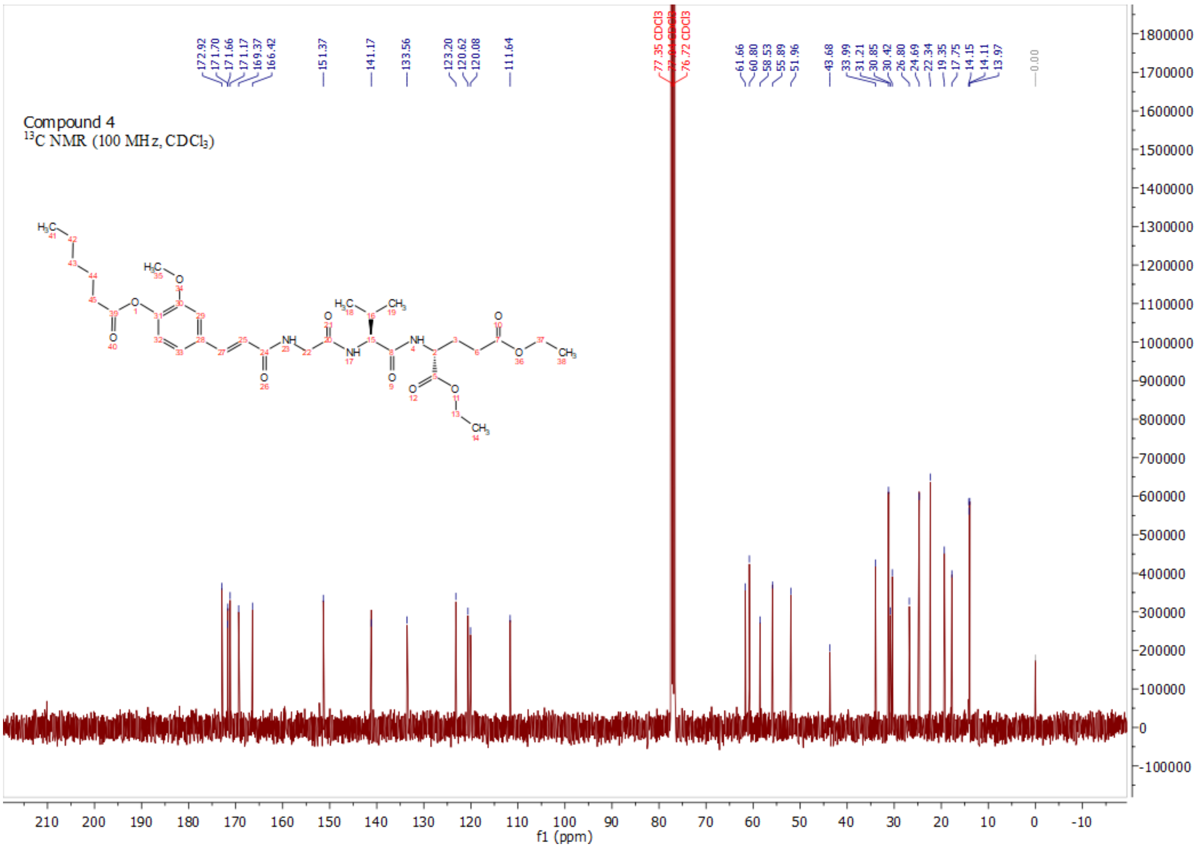
Compound 4
13C NMR (100 MHz, CDCl3)
f1 (ppm)

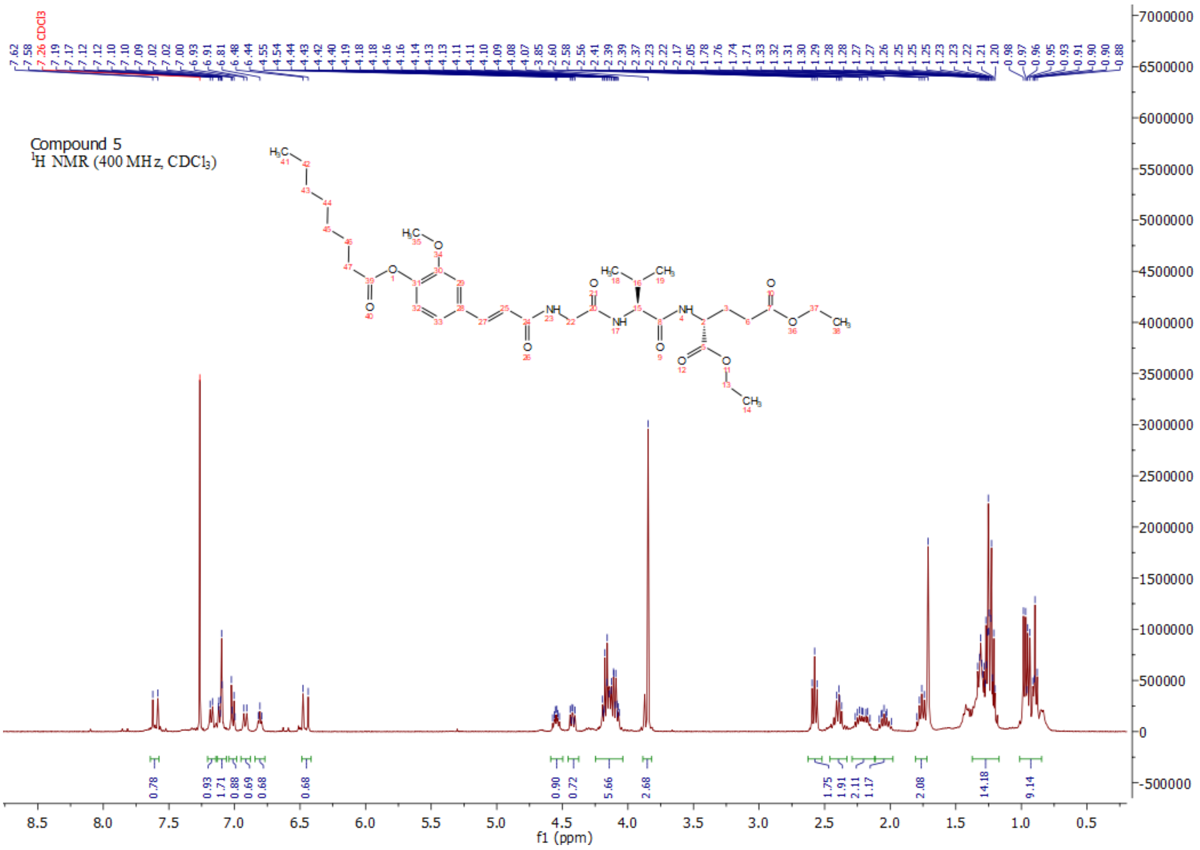
Compound 5
1H NMR (400 MHz, CDCl3)
f1 (ppm)

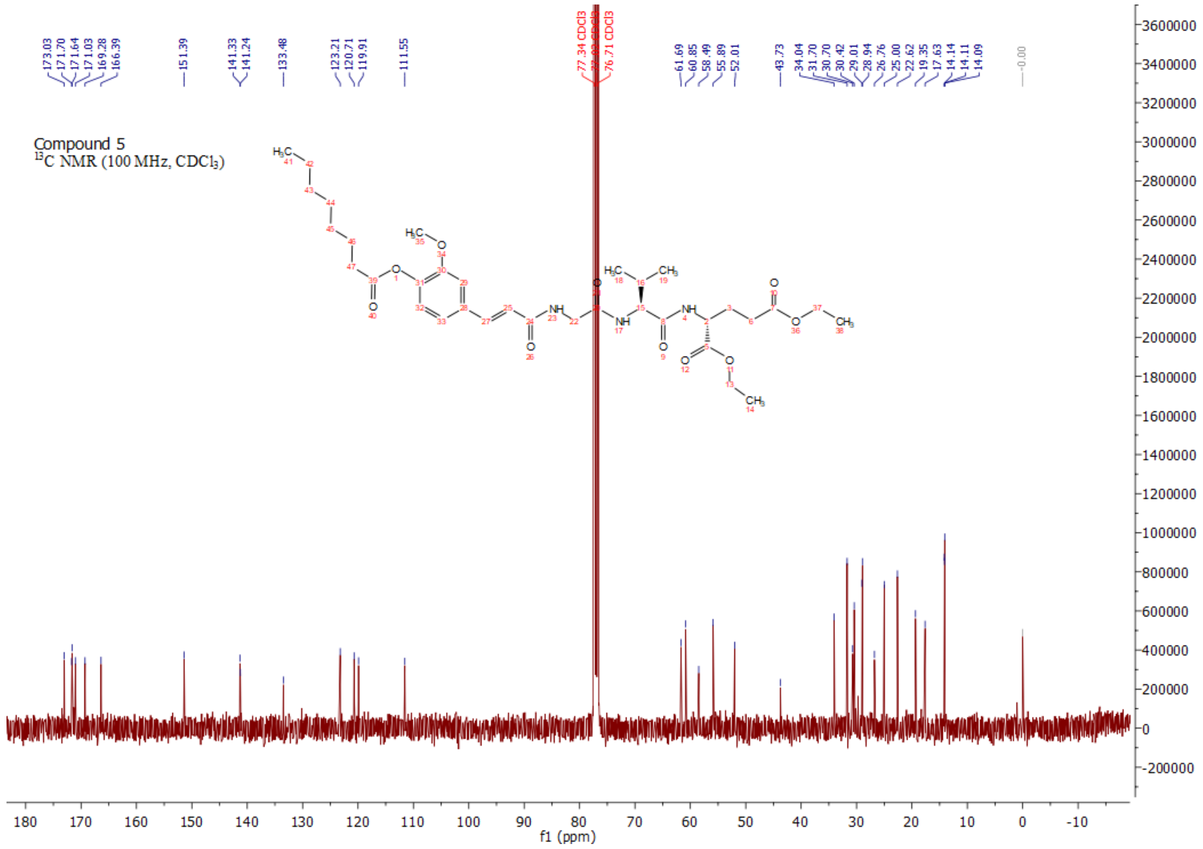

Compound 5
^{13}C NMR (100 MHz, CDCl$_3$)
f1 (ppm)

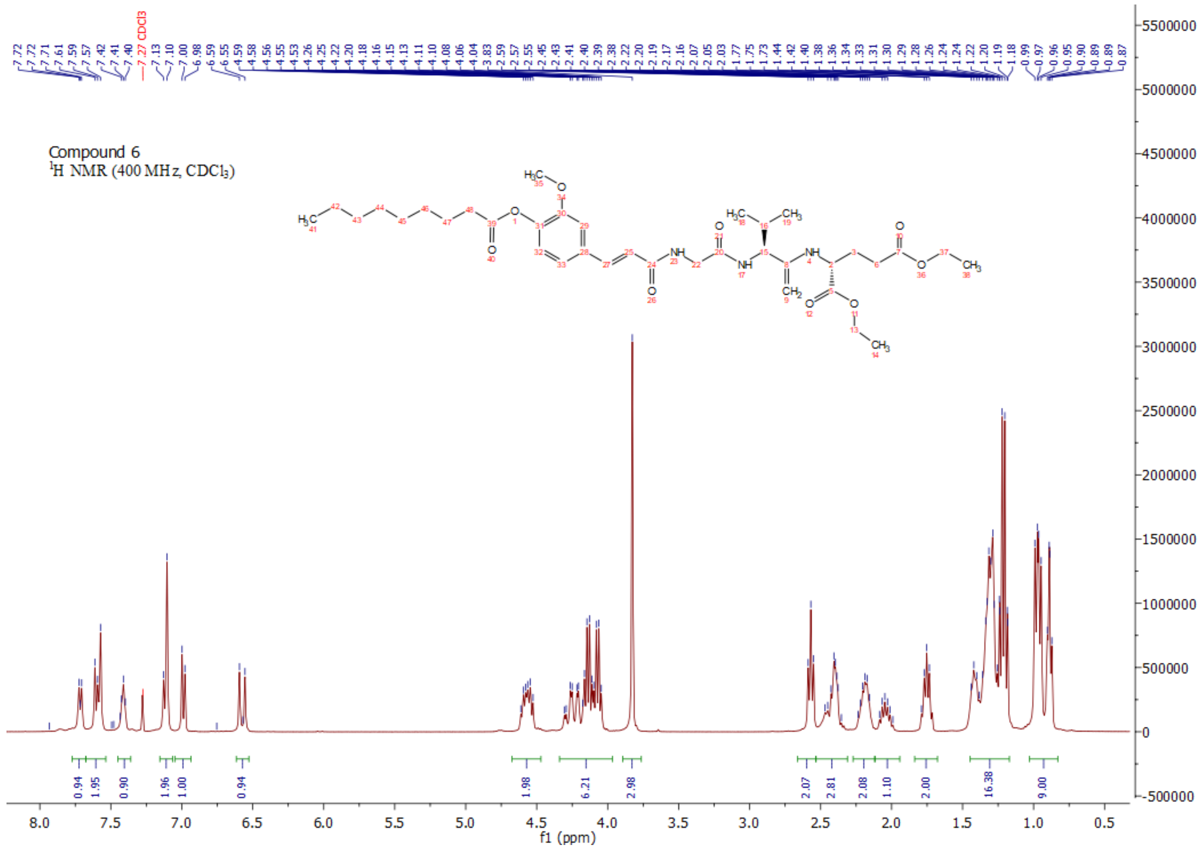

Compound 6
^{1}H NMR (400 MHz, CDCl$_3$)
f1 (ppm)

Compound 6
^{13}C NMR (100 MHz, $CDCl_3$)

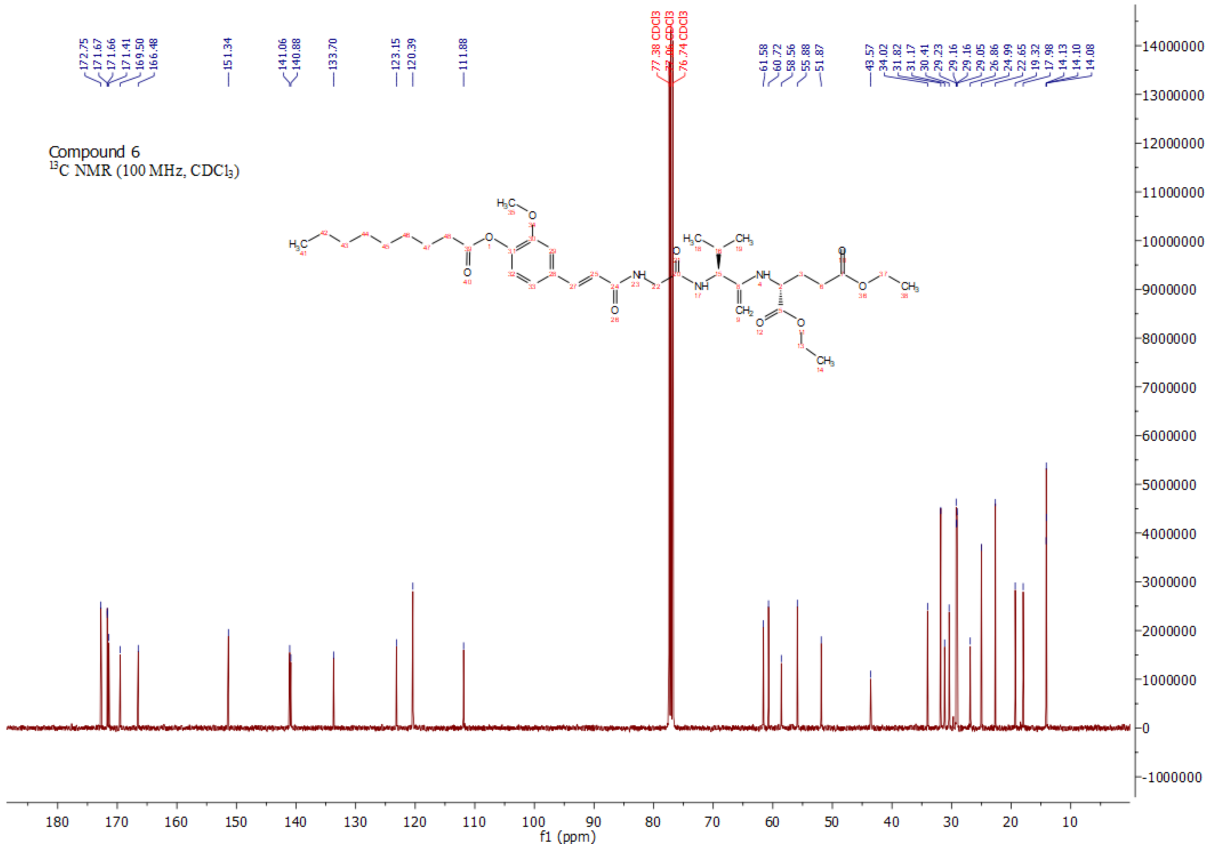

Compound 7
^{1}H NMR (400 MHz, $CDCl_3$)

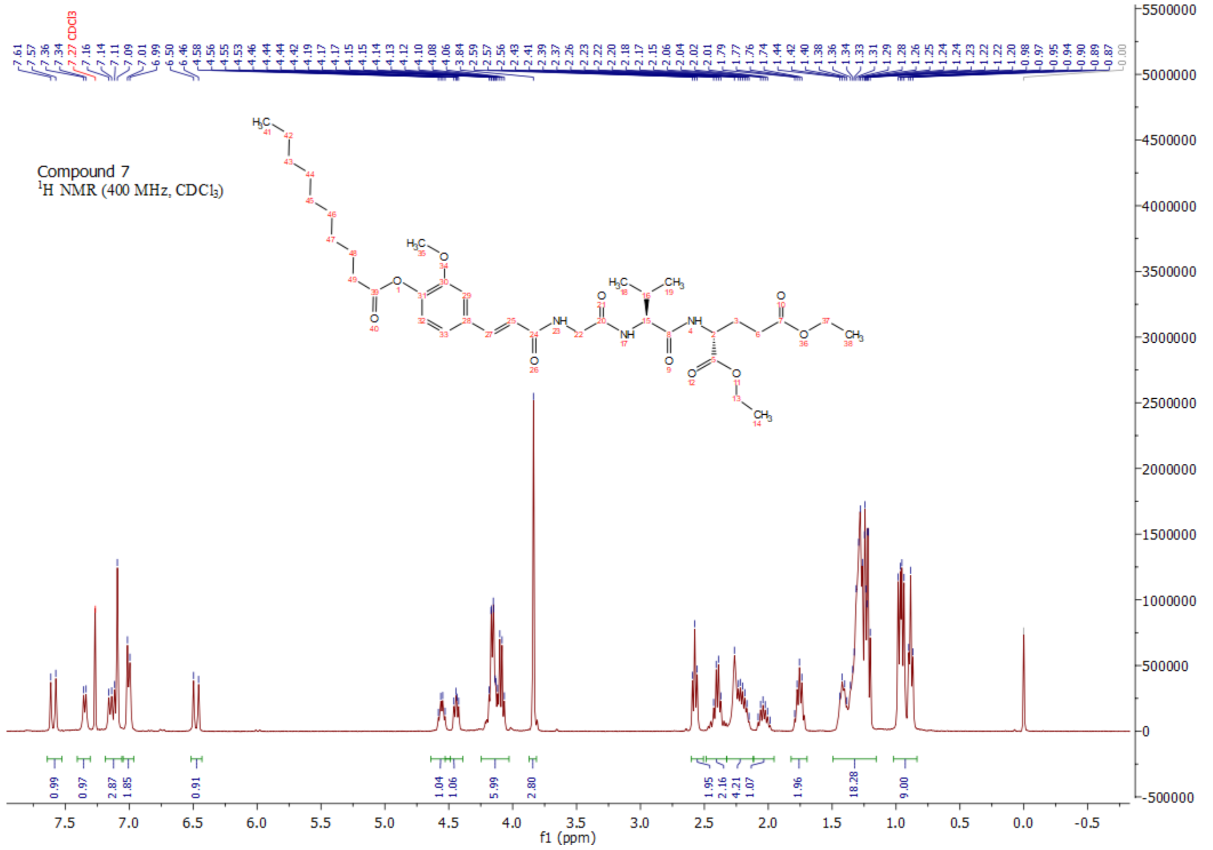

Compound 7
^{13}C NMR (100 MHz, $CDCl_3$)

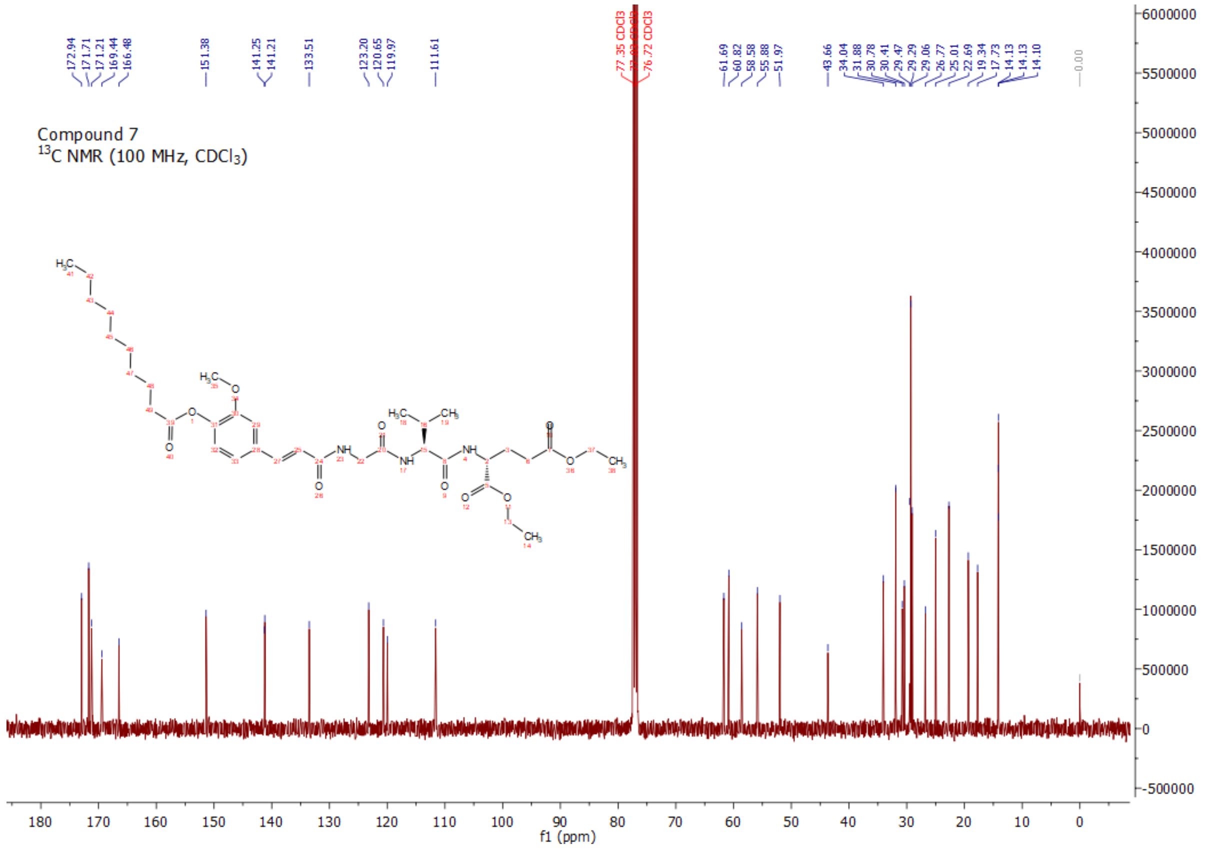

Compound 8
^{1}H NMR (400 MHz, $CDCl_3$)

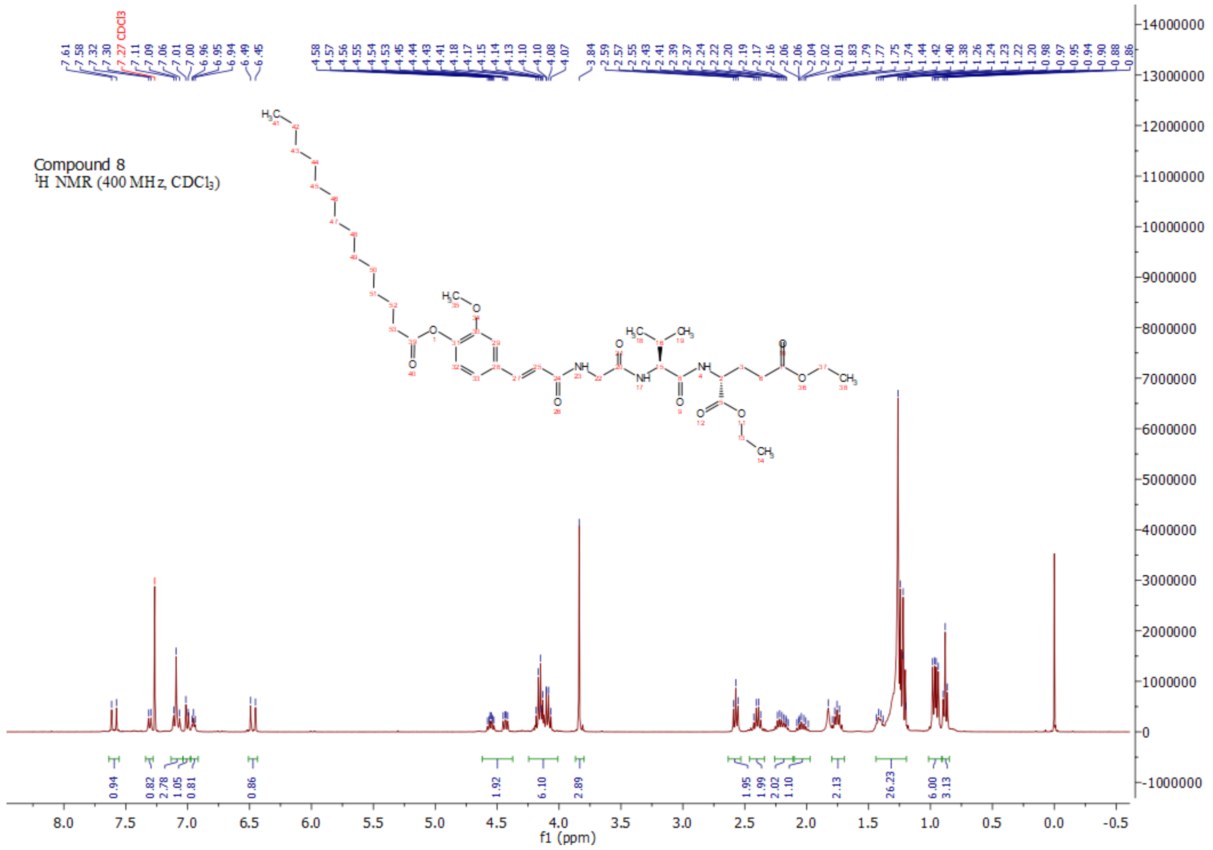

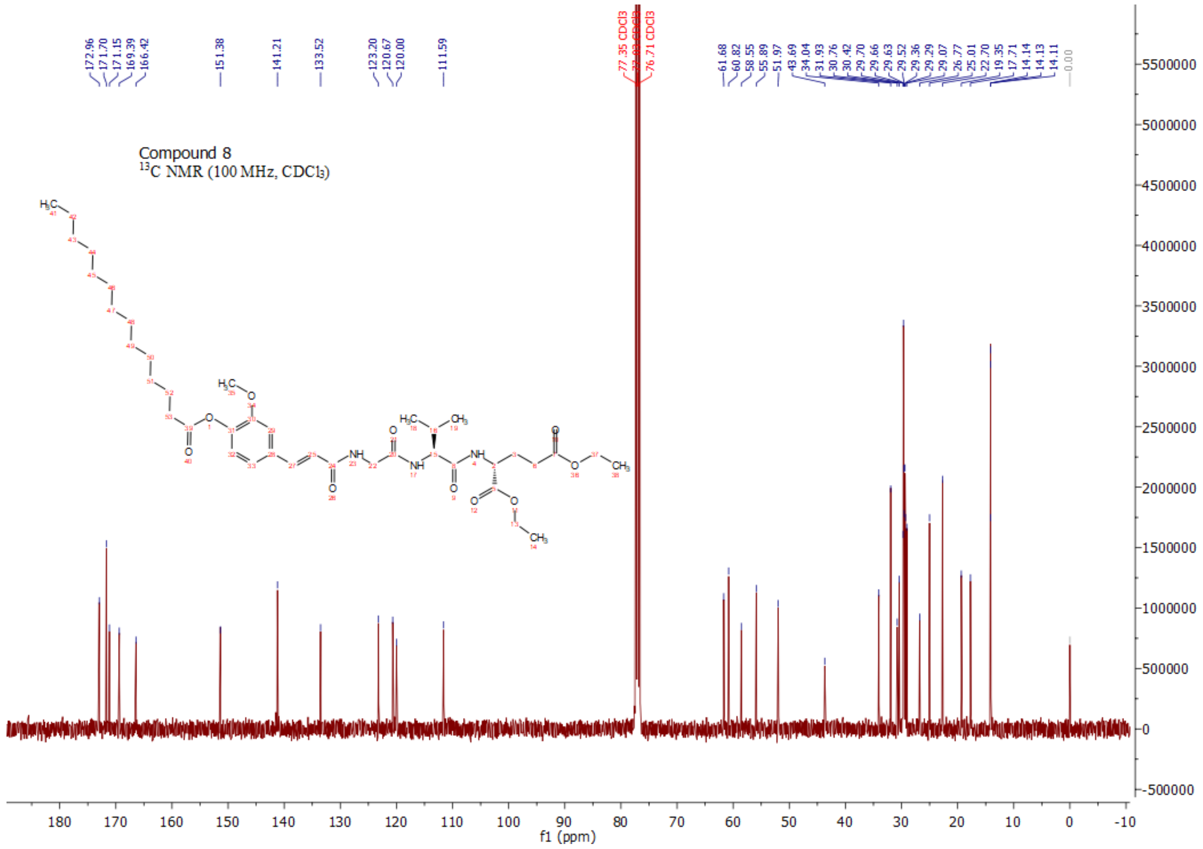
Compound 8
13C NMR (100 MHz, CDCl3)
f1 (ppm)

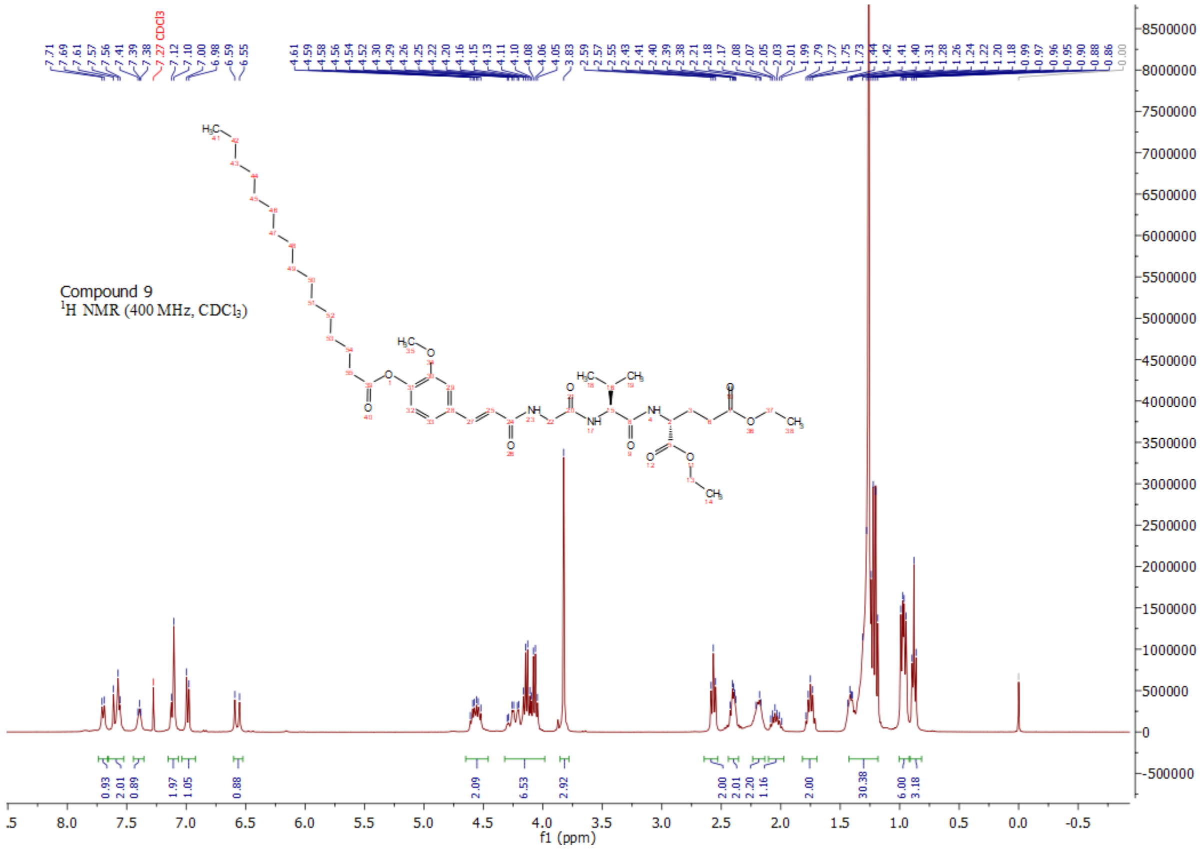
Compound 9
1H NMR (400 MHz, CDCl3)
f1 (ppm)

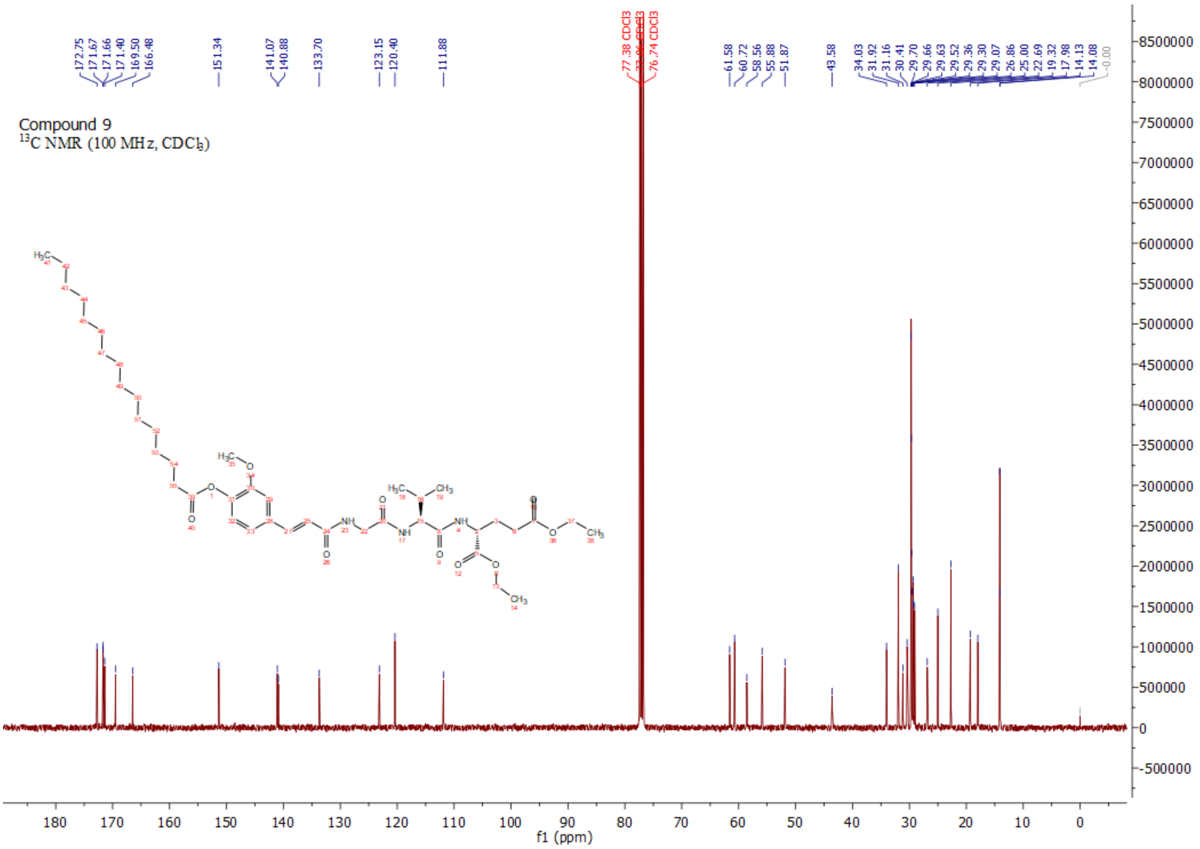
Compound 9
^{13}C NMR (100 MHz, CDCl$_3$)
f1 (ppm)

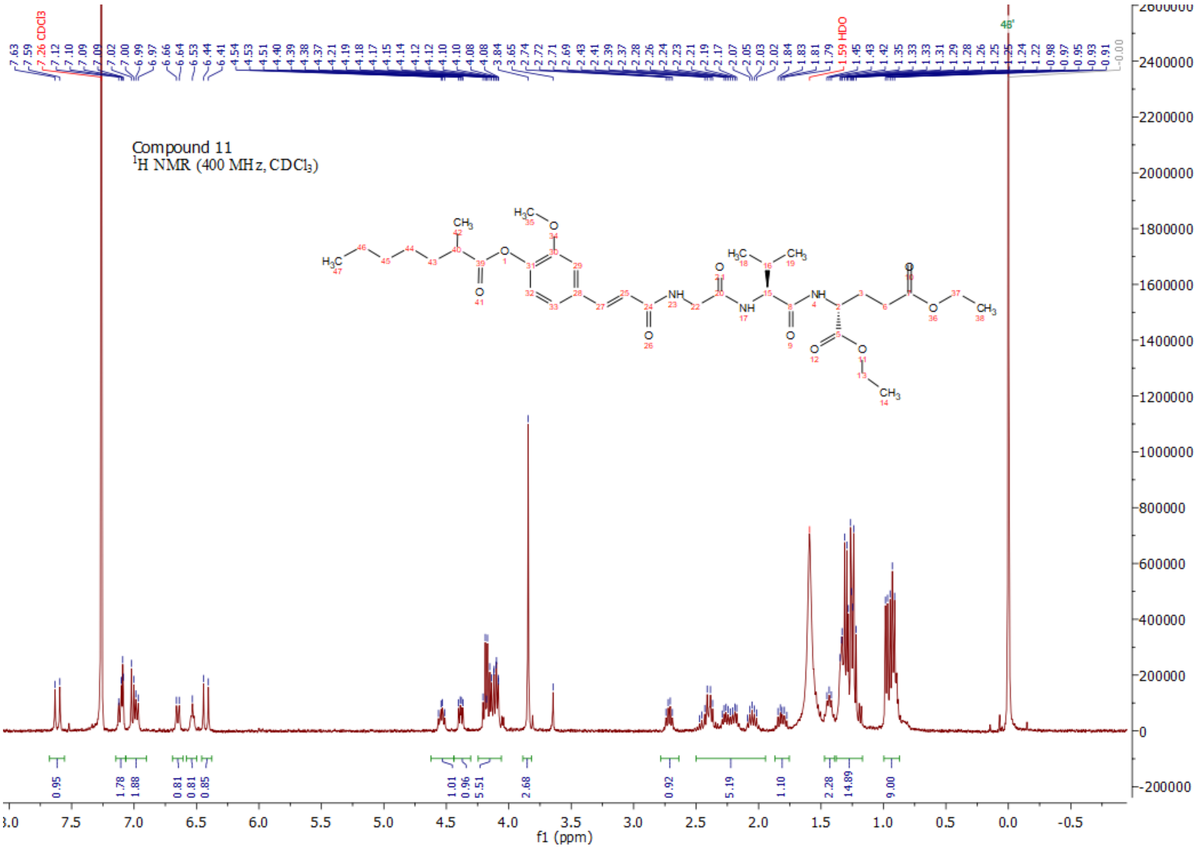
Compound 11
^{1}H NMR (400 MHz, CDCl$_3$)
f1 (ppm)

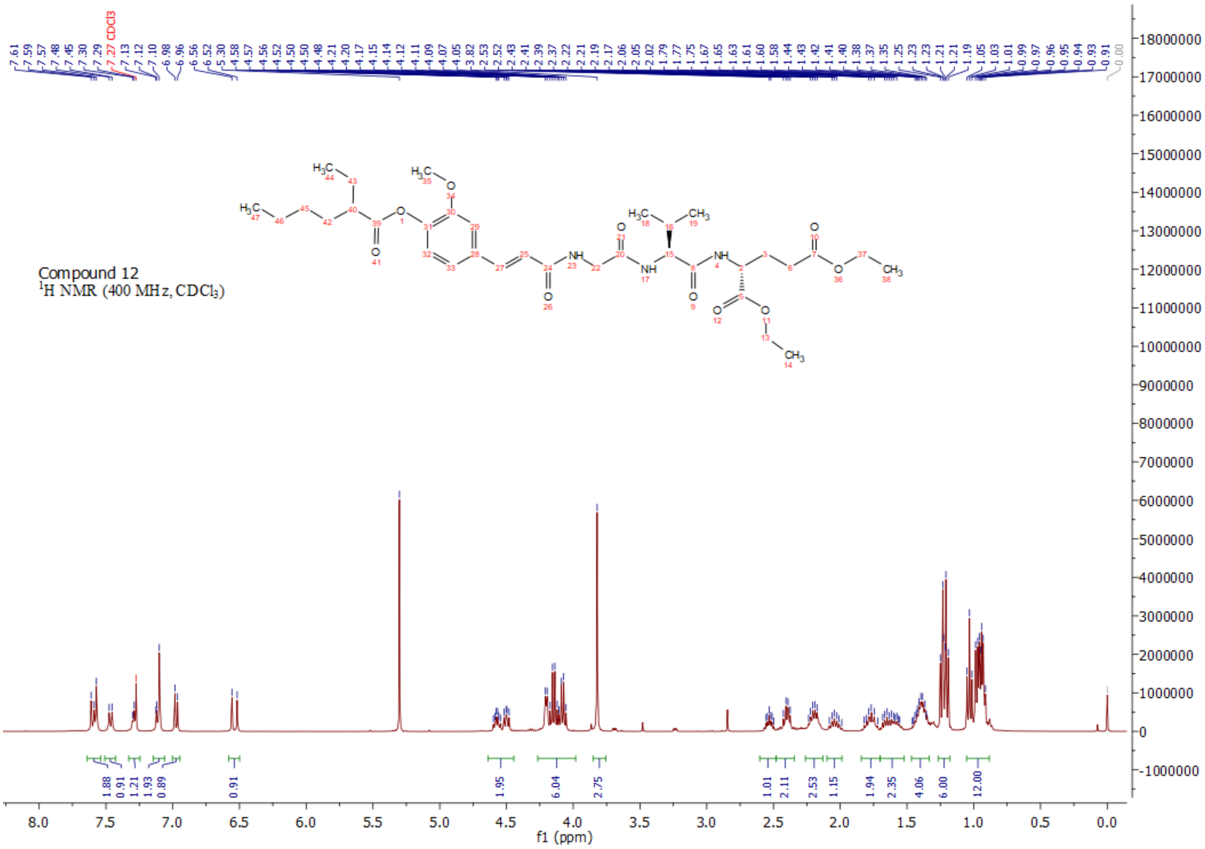
Compound 12
1H NMR (400 MHz, CDCl3)
f1 (ppm)

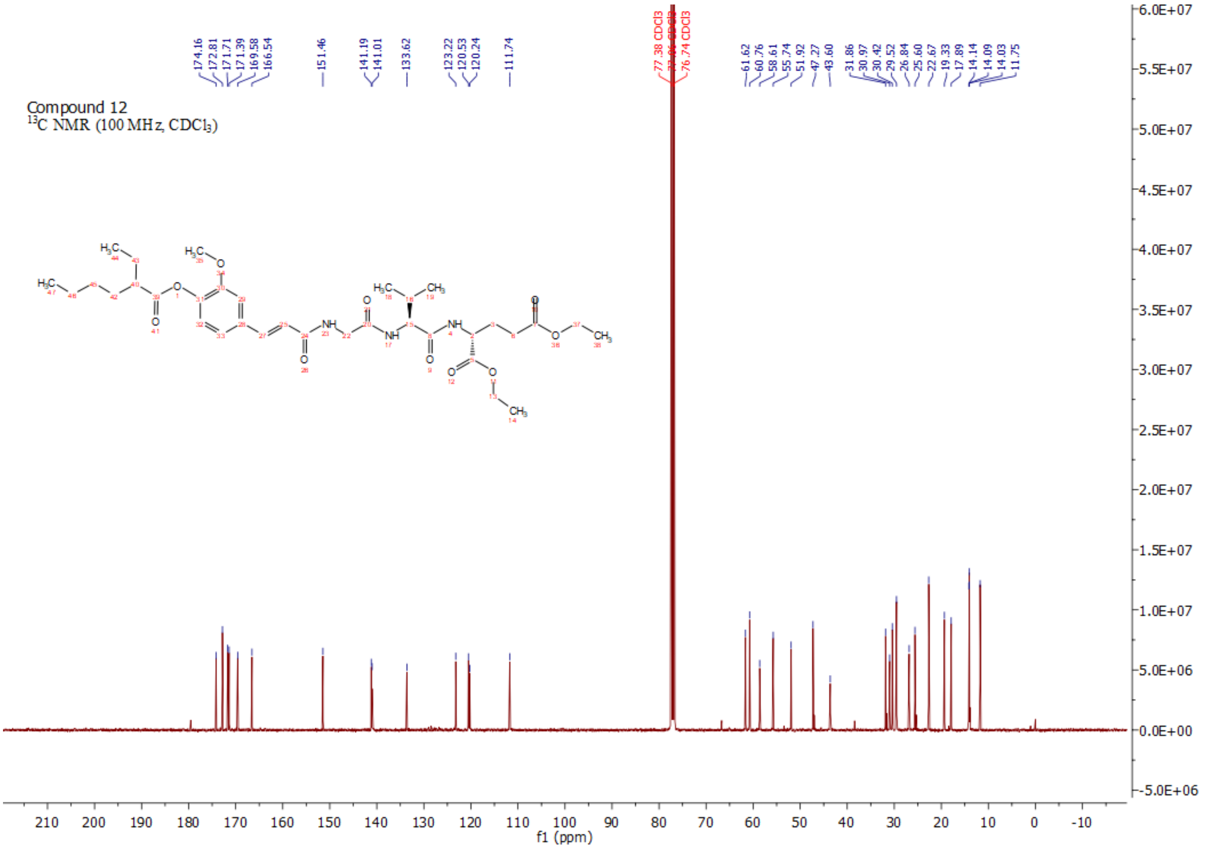
Compound 12
13C NMR (100 MHz, CDCl3)
f1 (ppm)

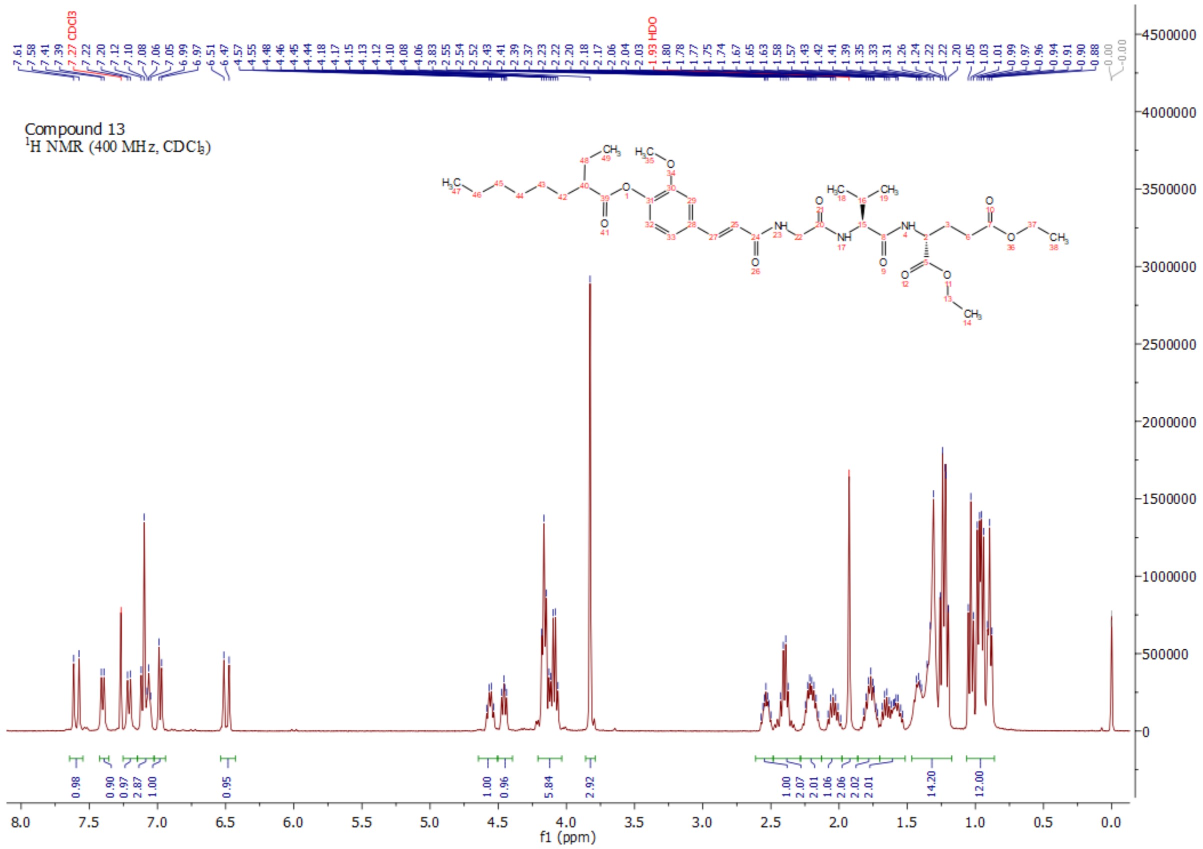
Compound 13
^{1}H NMR (400 MHz, CDCl$_3$)
f1 (ppm)

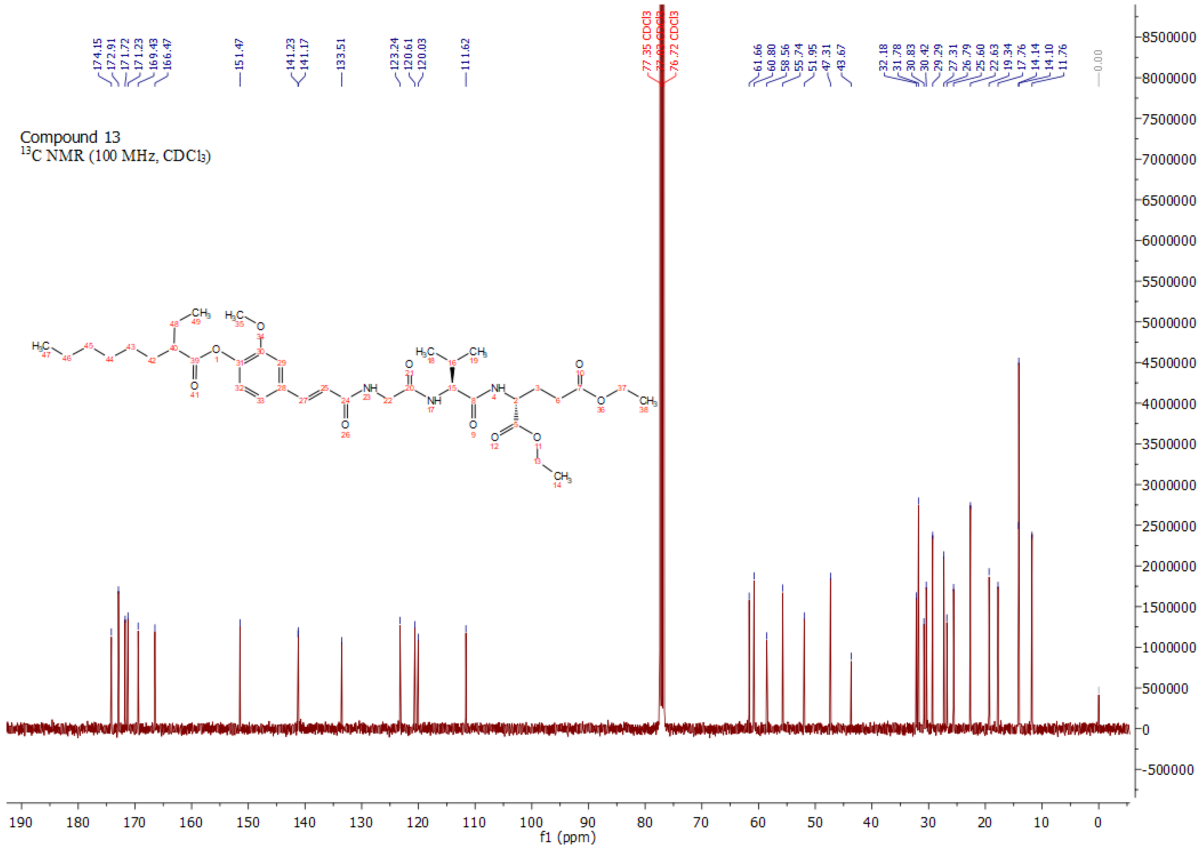
Compound 13
^{13}C NMR (100 MHz, CDCl$_3$)
f1 (ppm)

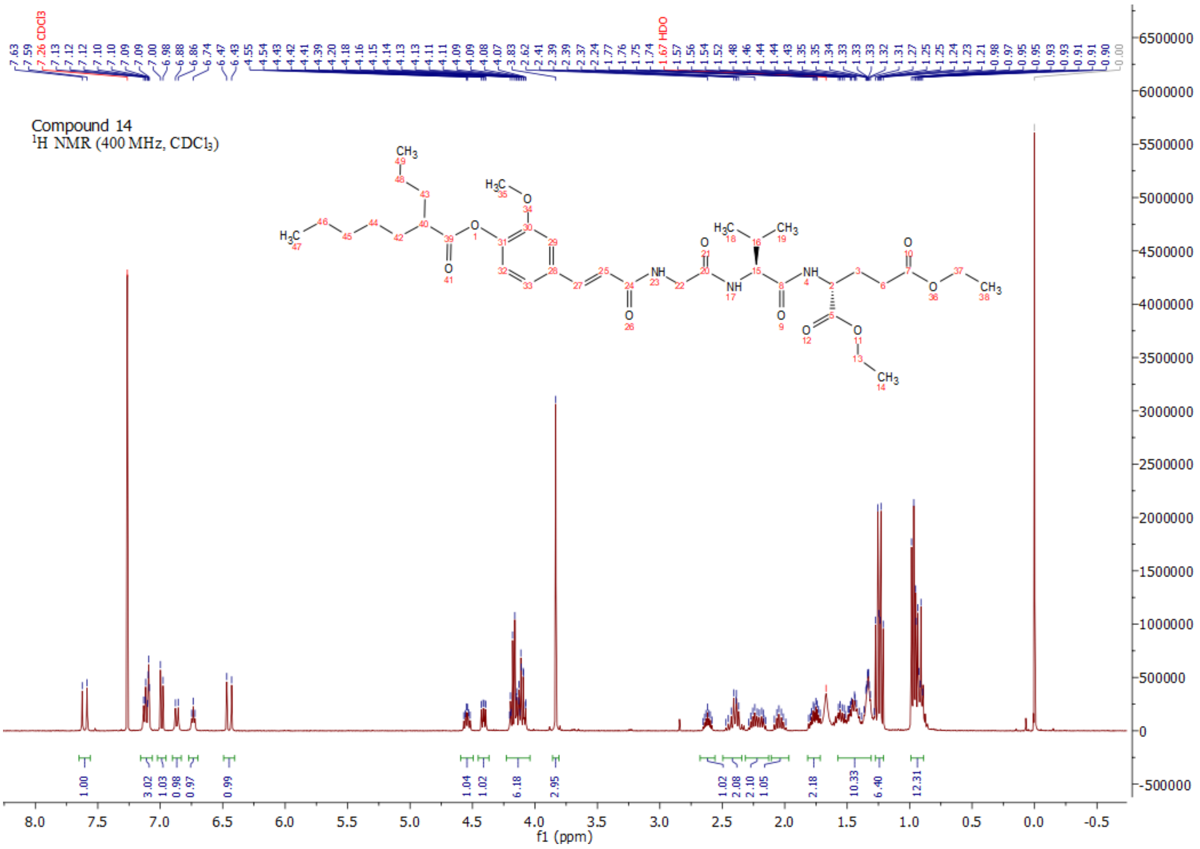
Compound 14
^{1}H NMR (400 MHz, $CDCl_3$)

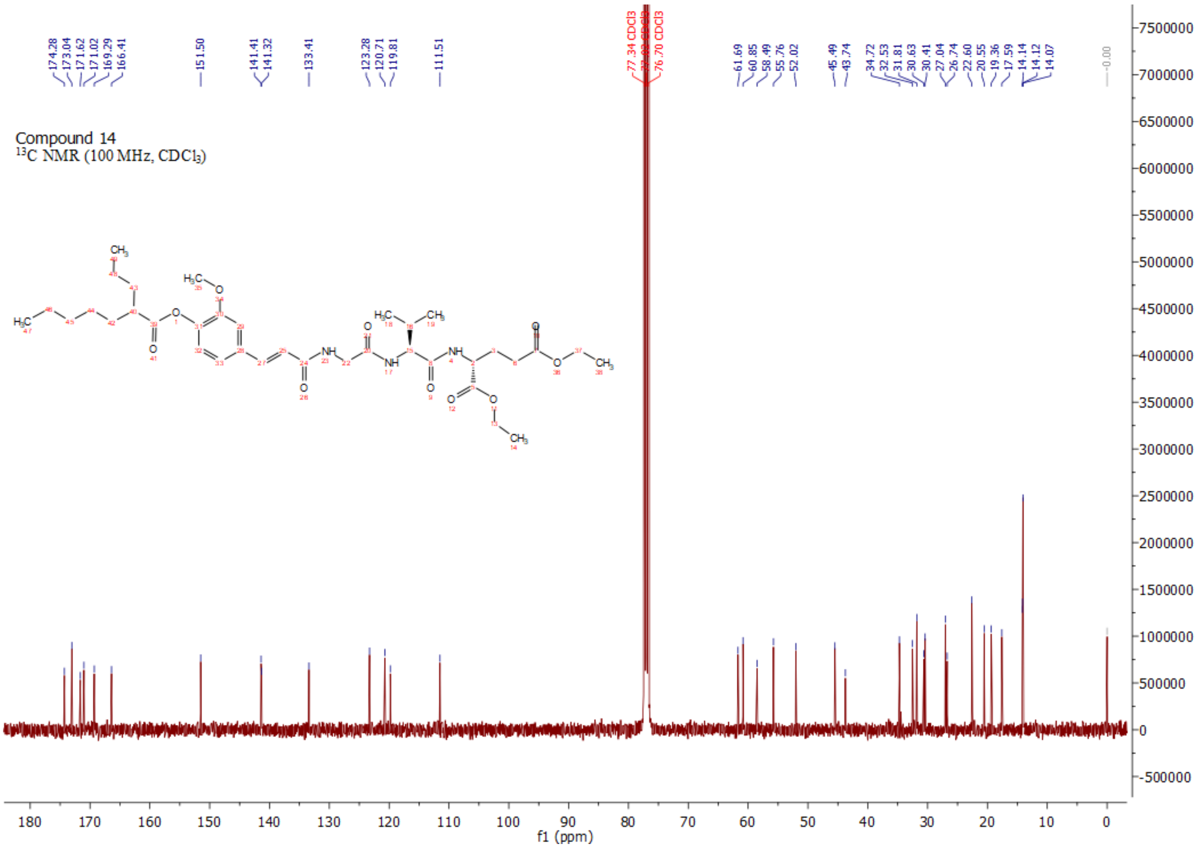
Compound 14
^{13}C NMR (100 MHz, $CDCl_3$)

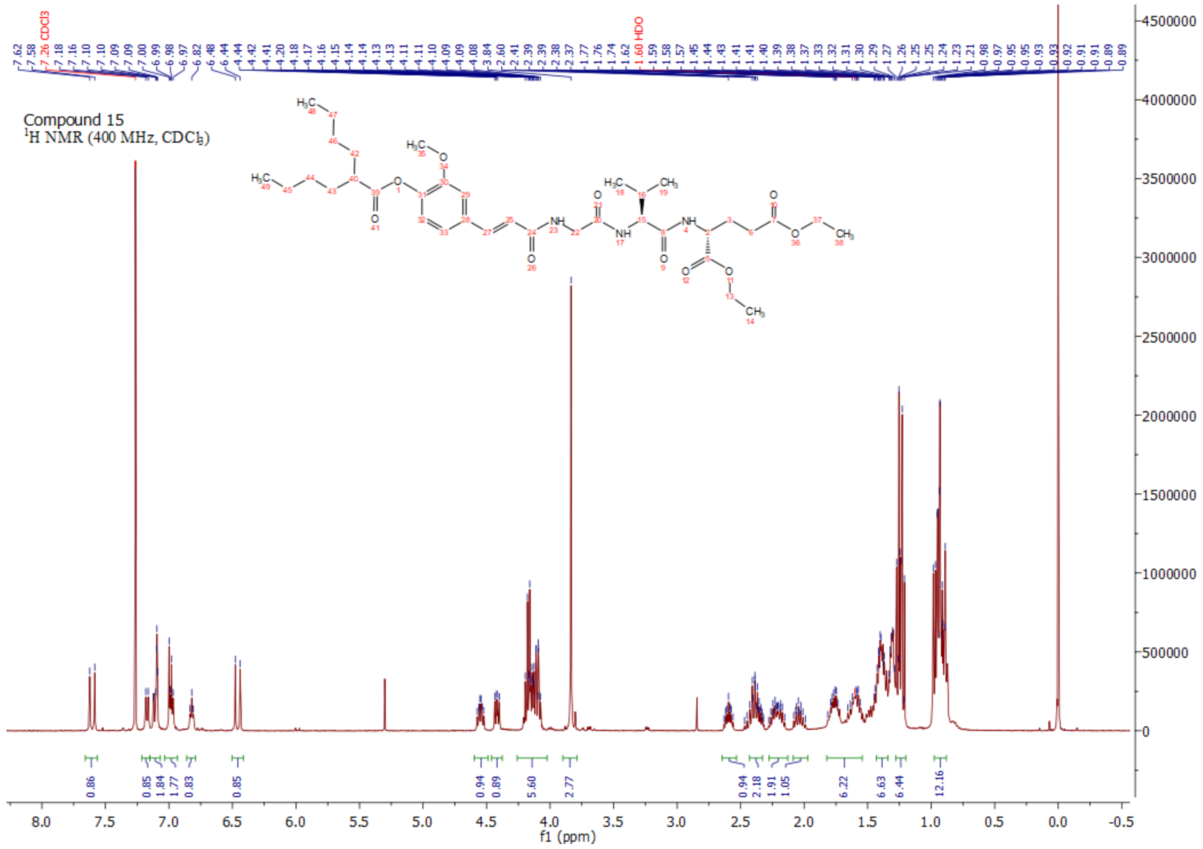
Compound 15
^{1}H NMR (400 MHz, CDCl$_3$)
f1 (ppm)

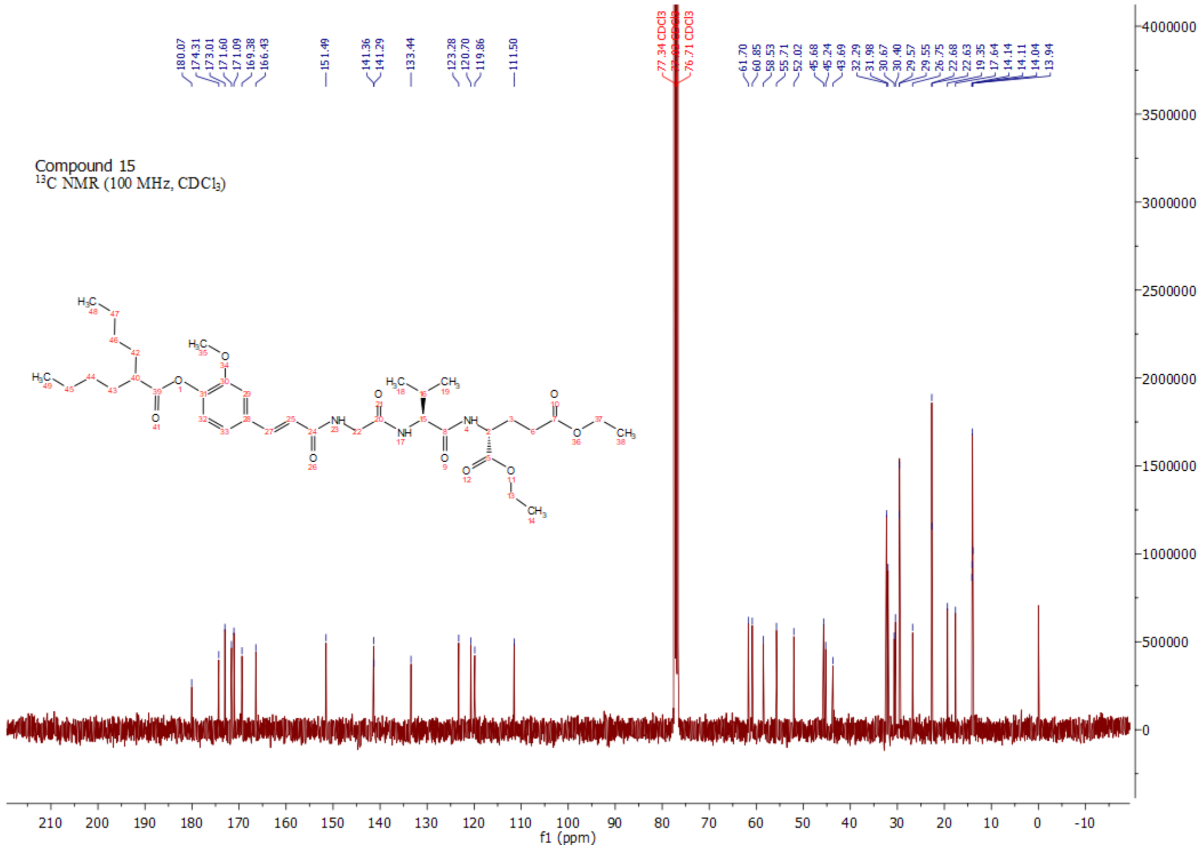
Compound 15
^{13}C NMR (100 MHz, CDCl$_3$)
f1 (ppm)

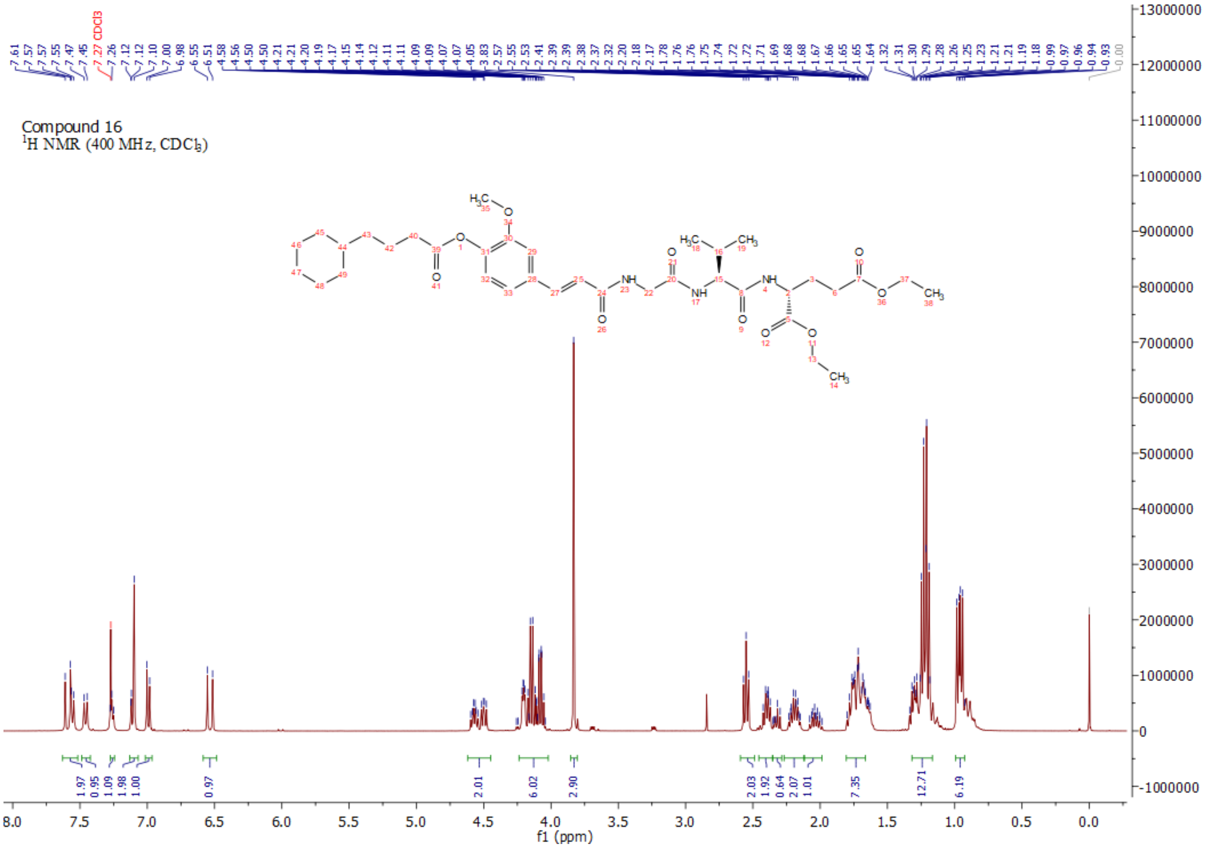

7.61
7.57
7.57
7.55
7.47
7.45
7.27 CDCl3
7.26
7.12
7.12
7.10
7.00
6.98
6.55
6.51
4.58
4.56
4.50
4.50
4.21
4.21
4.20
4.19
4.17
4.15
4.14
4.12
4.11
4.11
4.09
4.09
4.07
4.07
4.05
3.83
2.57
2.55
2.53
2.41
2.39
2.39
2.38
2.37
2.32
2.20
2.18
2.17
1.78
1.76
1.76
1.75
1.74
1.72
1.72
1.71
1.69
1.68
1.68
1.67
1.66
1.65
1.65
1.64
1.32
1.31
1.30
1.29
1.28
1.26
1.25
1.23
1.21
1.21
1.19
1.18
0.99
0.97
0.96
0.94
0.93
0.00
Compound 16
1H NMR (400 MHz, CDCl3)
1.97
0.95
1.09
1.98
1.00
0.97
2.01
6.02
2.90
2.03
1.92
0.64
2.07
1.01
7.35
12.71
6.19
f1 (ppm)

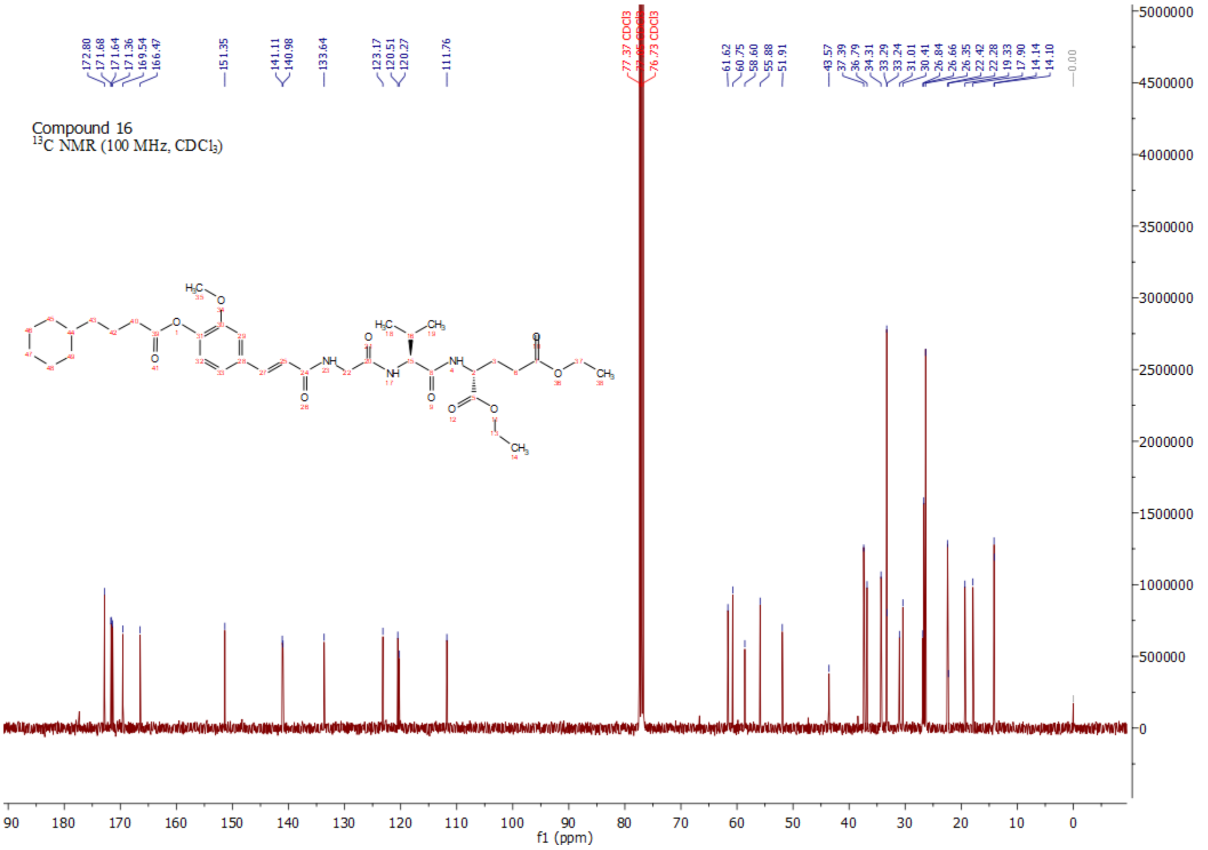

172.80
171.68
171.64
171.36
169.54
166.47
151.35
141.11
140.98
133.64
123.17
120.51
120.27
111.76
77.37 CDCl3
76.73 CDCl3
61.62
60.75
58.60
55.88
51.91
43.57
37.39
36.79
34.31
33.29
33.24
31.01
30.41
26.84
26.66
26.35
22.42
22.28
19.33
17.90
14.14
14.10
0.00
Compound 16
13C NMR (100 MHz, CDCl3)
f1 (ppm)

Compound 17
^{1}H NMR (400 MHz, $CDCl_3$)

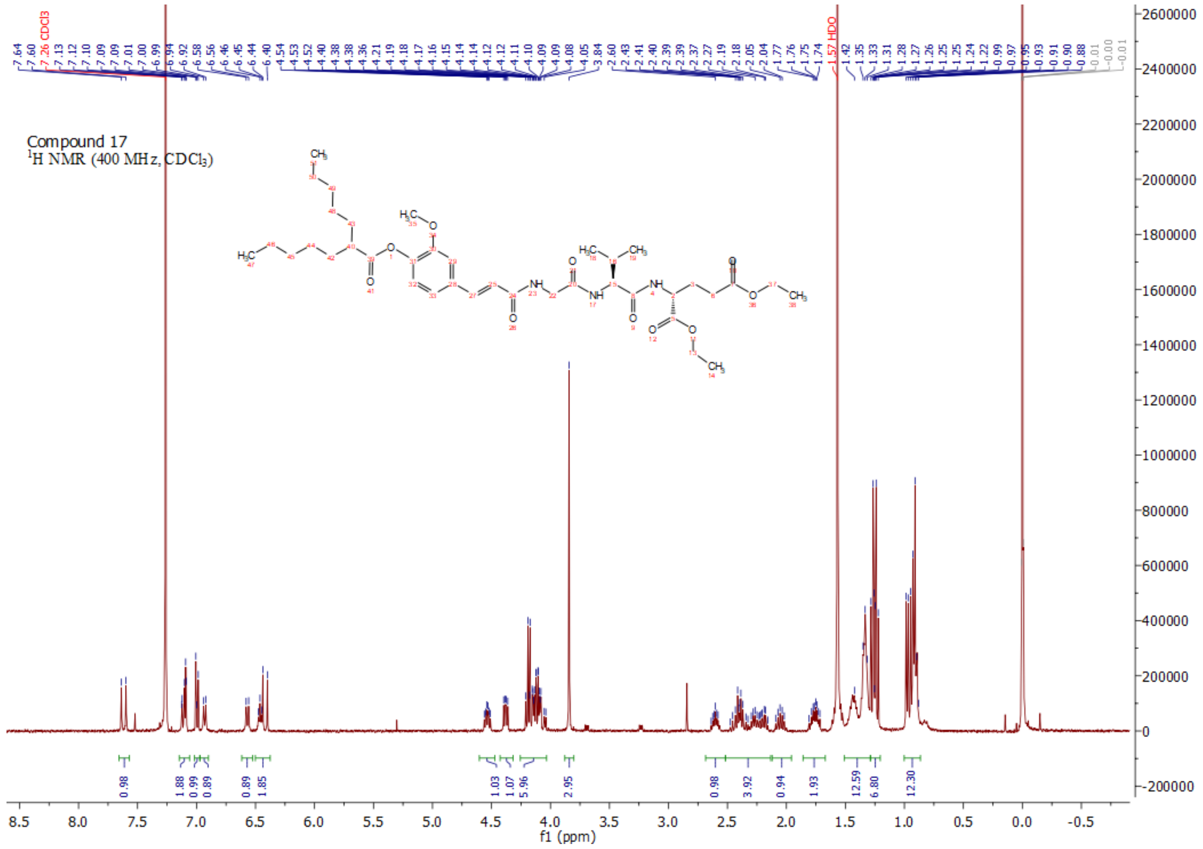

Compound 18
^{1}H NMR (400 MHz, $CDCl_3$)

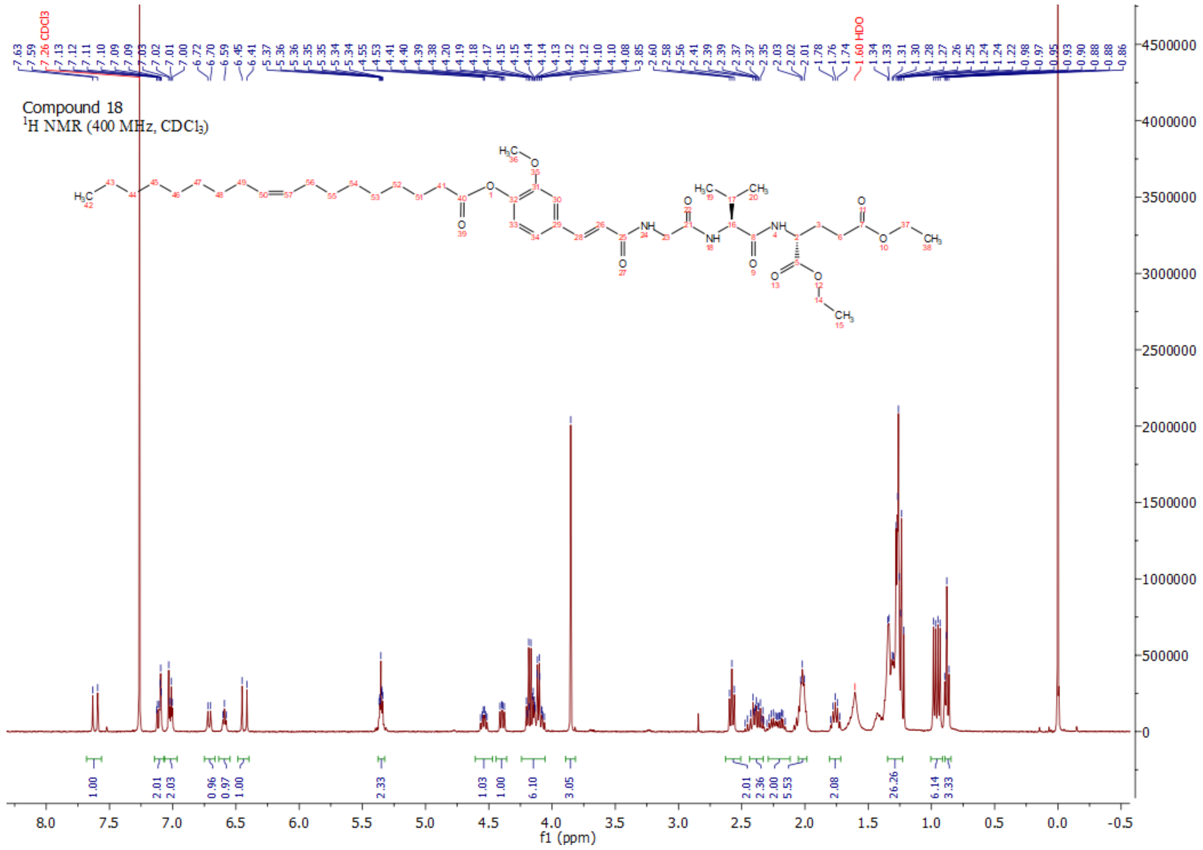

Compound 18
^{13}C NMR (100 MHz, CDCl$_3$)

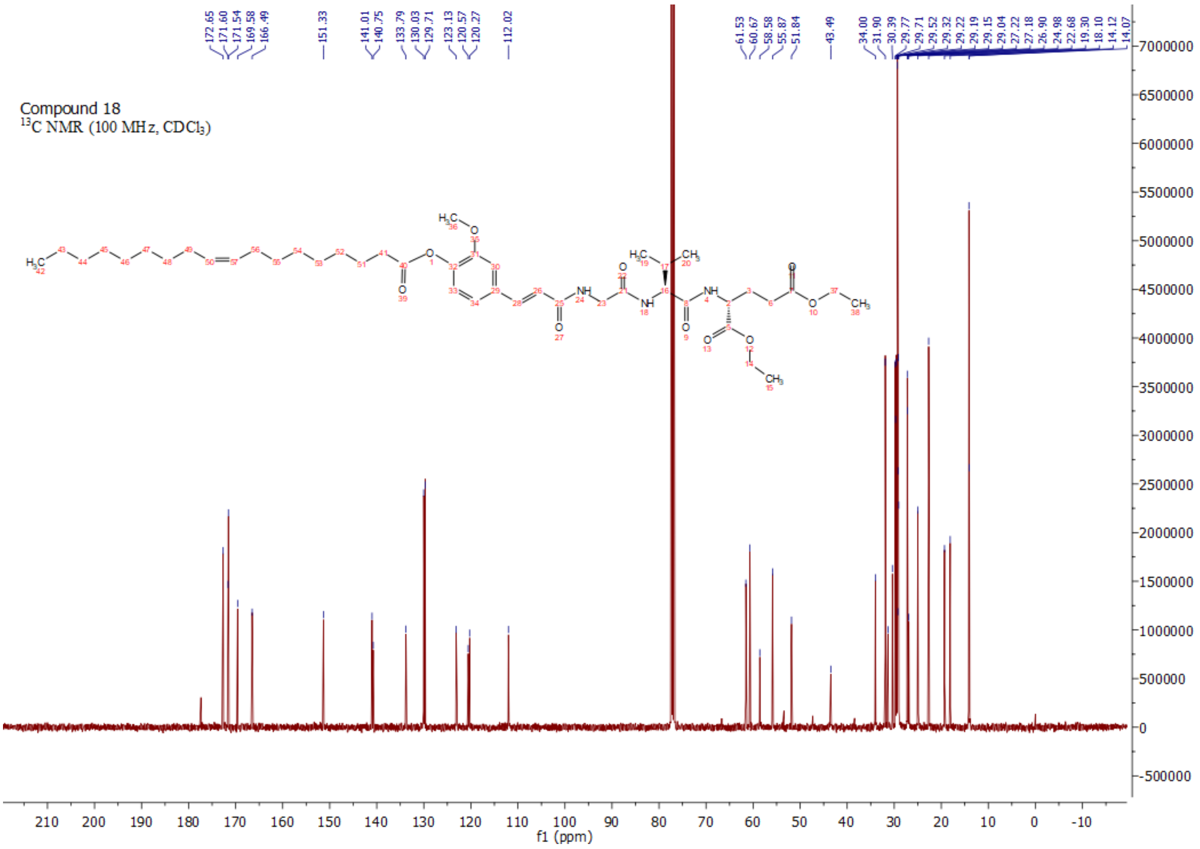

Compound 19
^{1}H NMR (400 MHz, CDCl$_3$) .

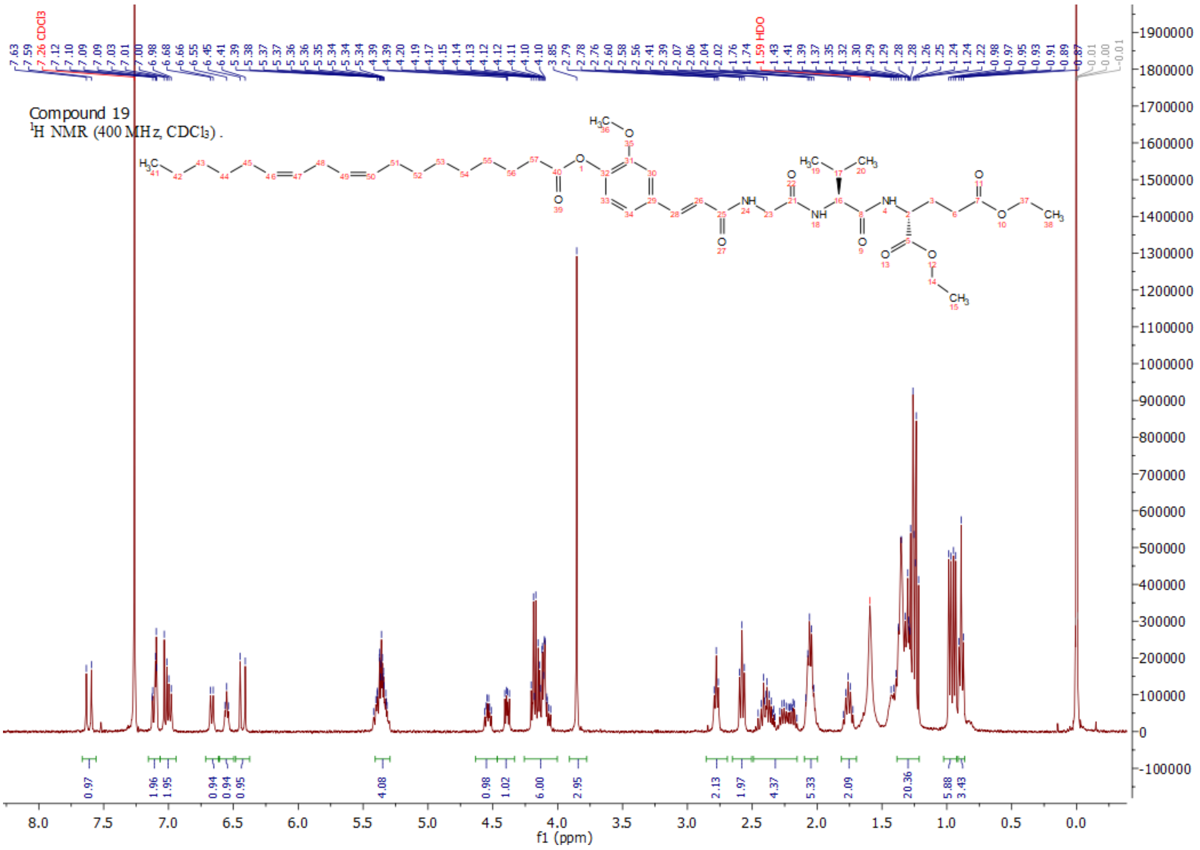

Compound 19
^{13}C NMR (100 MHz, $CDCl_3$)

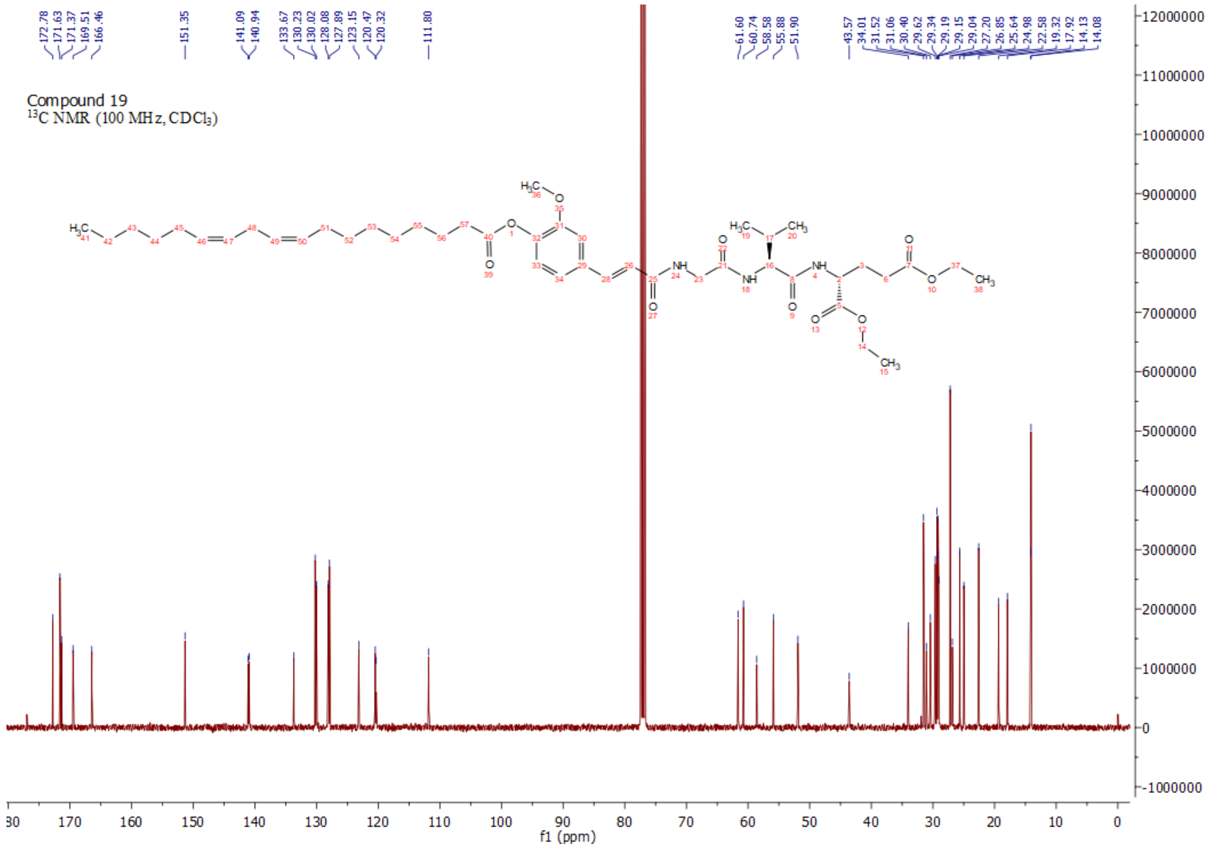

Compound 20
^{1}H NMR (400 MHz, $CDCl_3$)

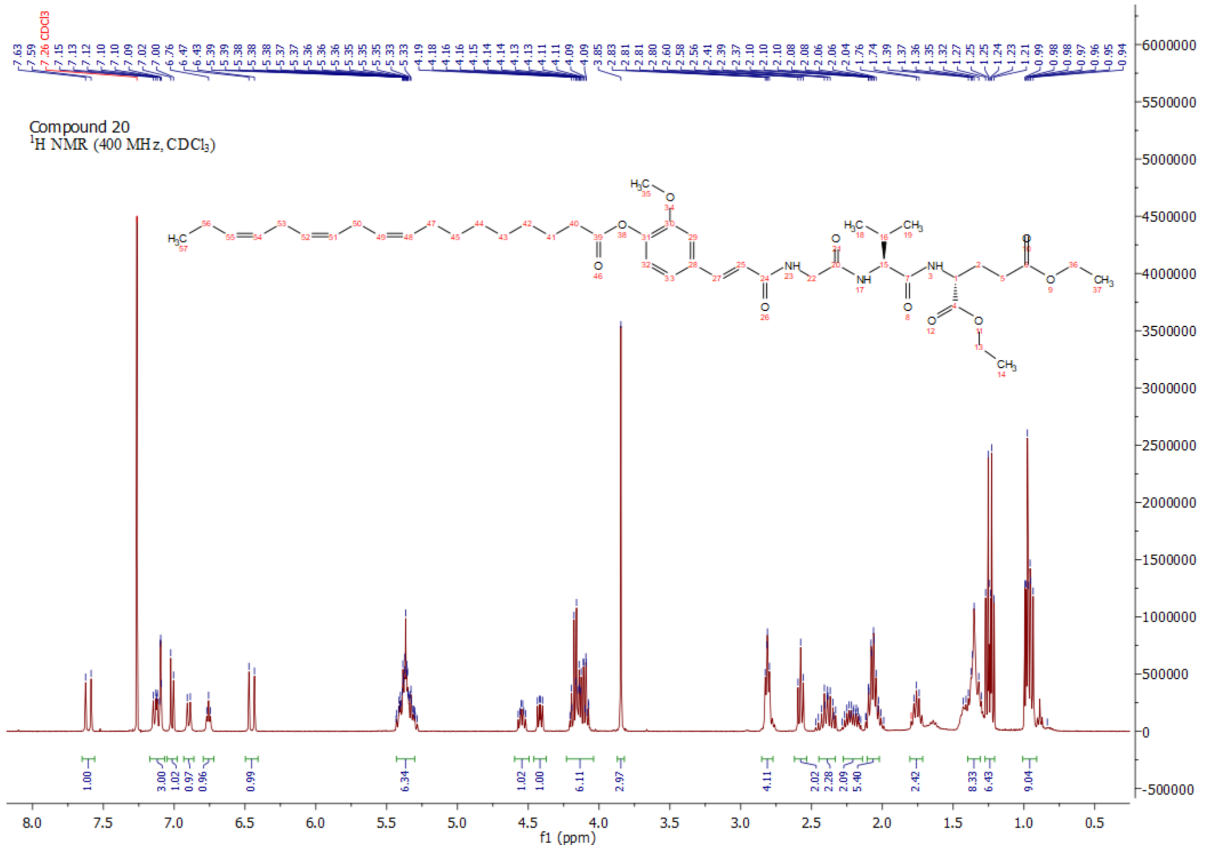

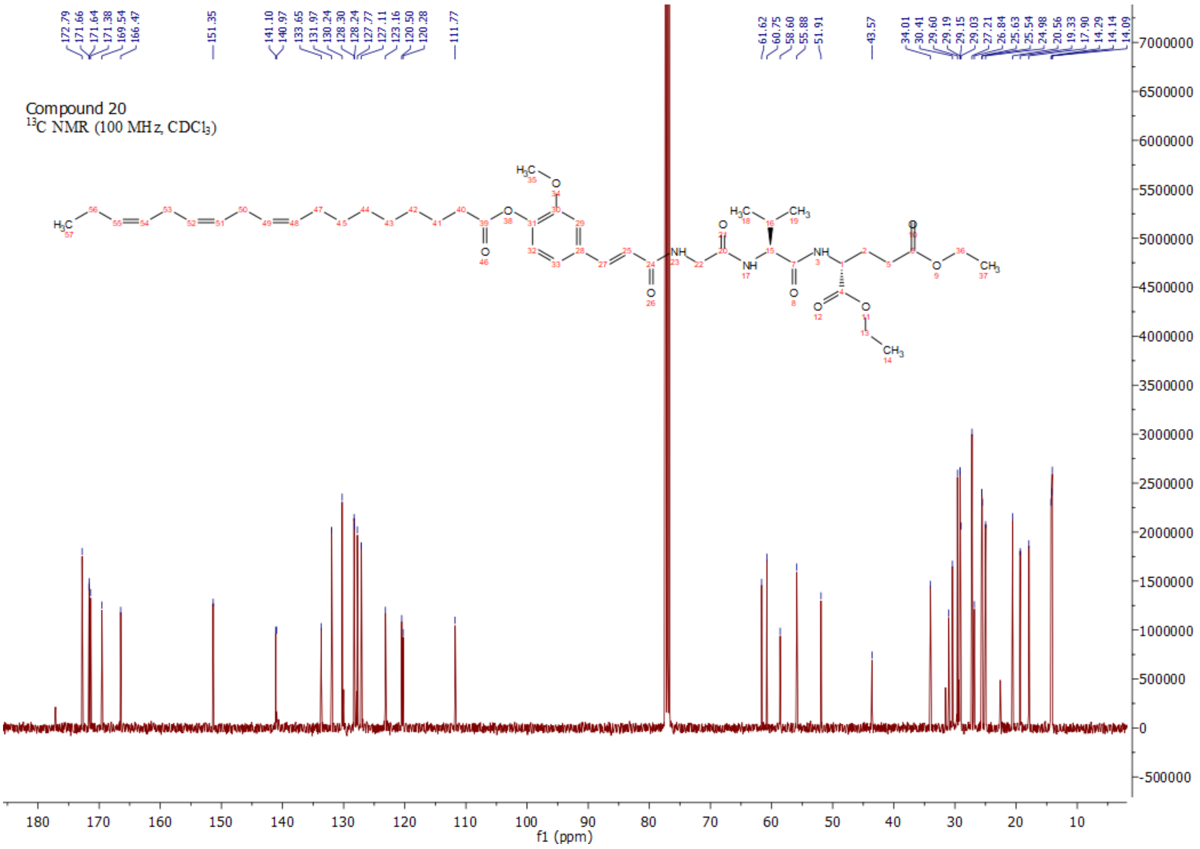

Compound 20
13C NMR (100 MHz, CDCl3)
f1 (ppm)

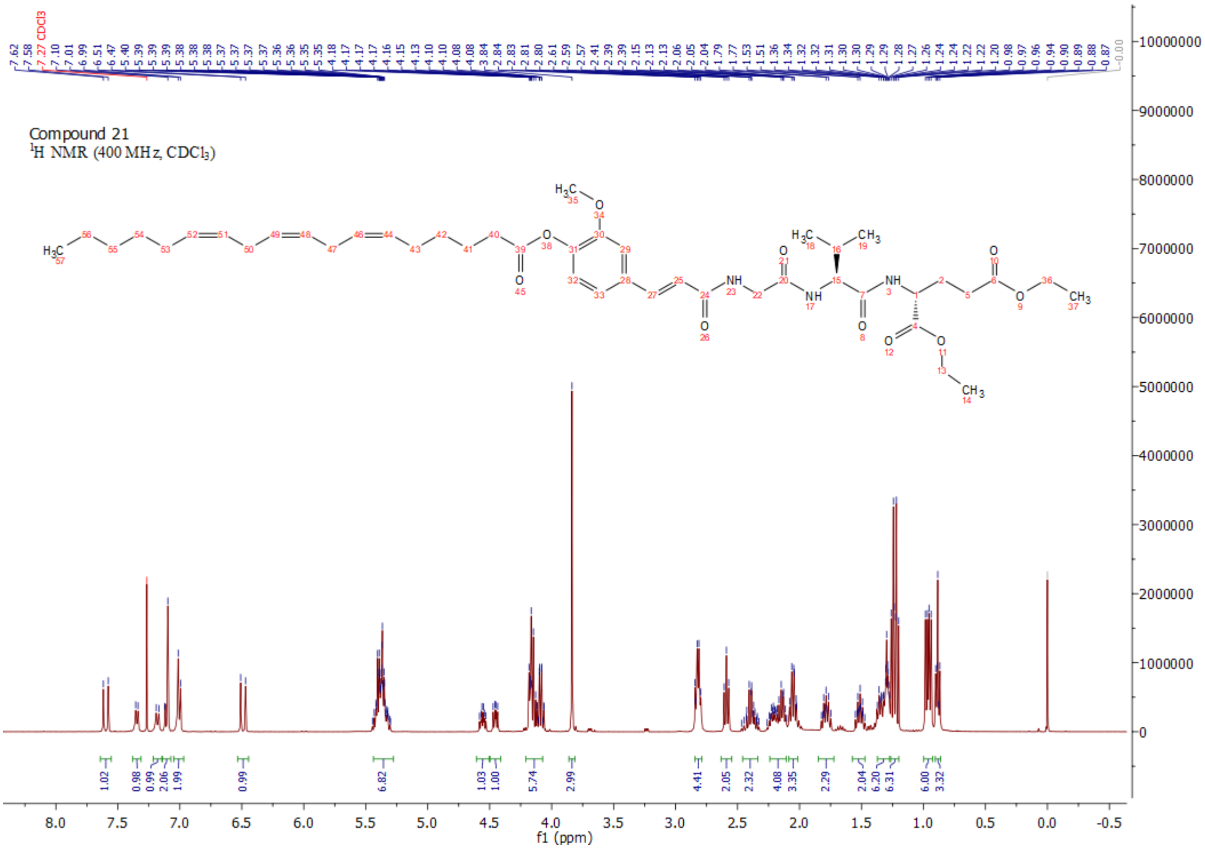

Compound 21
1H NMR (400 MHz, CDCl3)
f1 (ppm)

Compound 21
^{13}C NMR (100 MHz, $CDCl_3$)

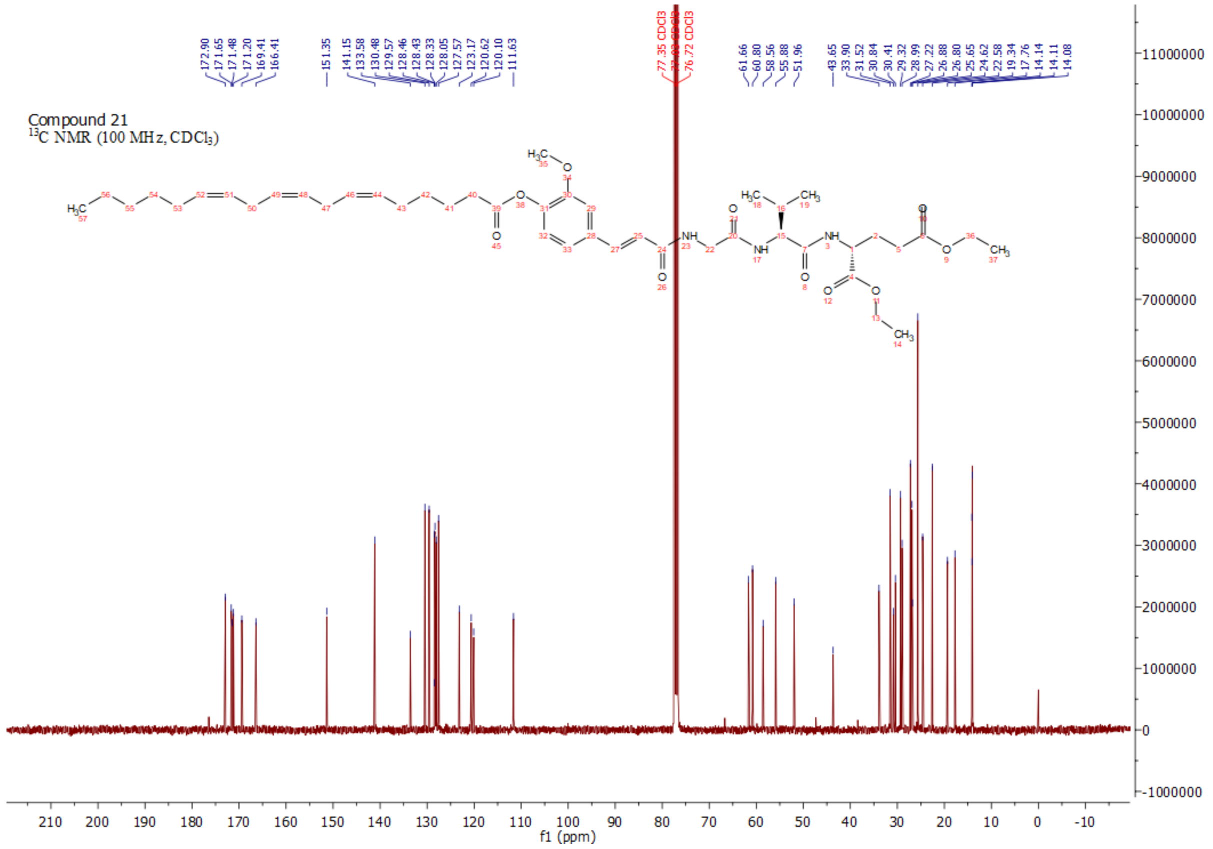

Compound 22
^{1}H NMR (400 MHz, $CDCl_3$)

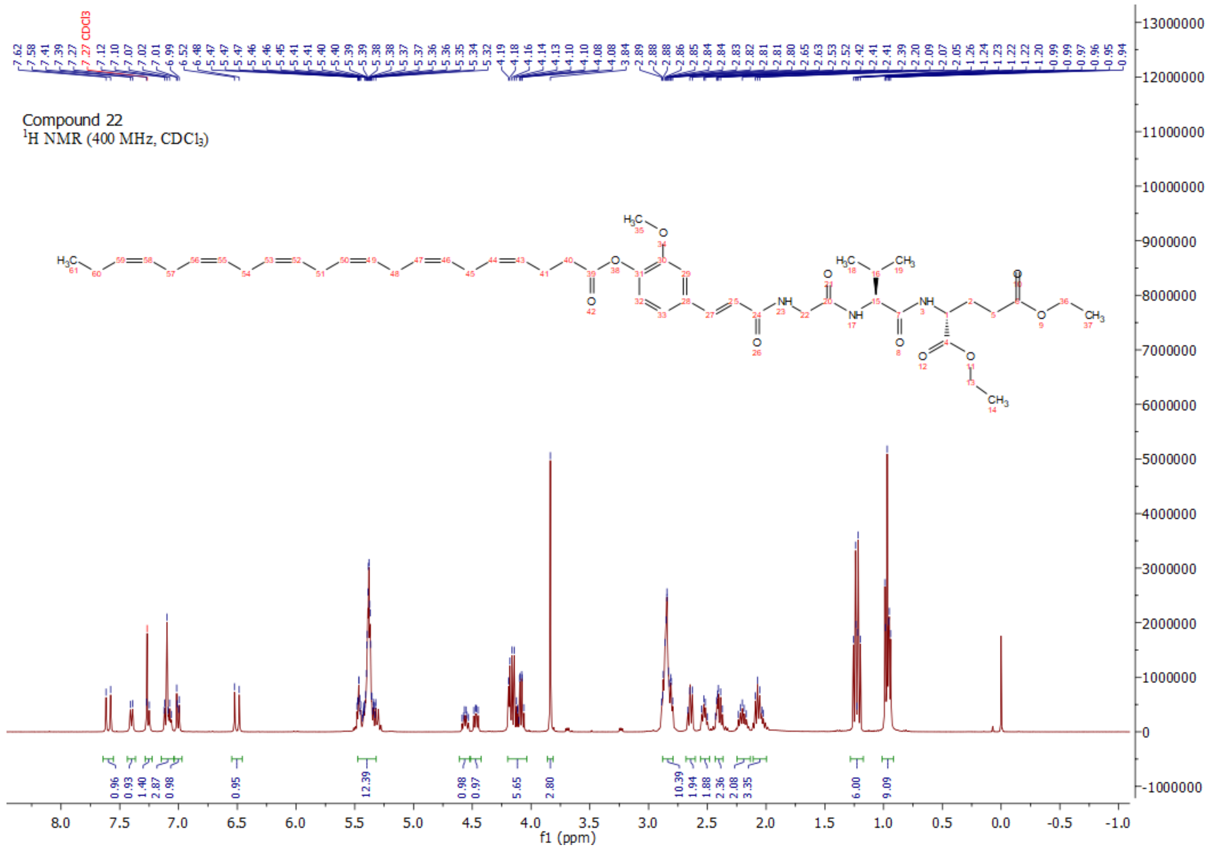

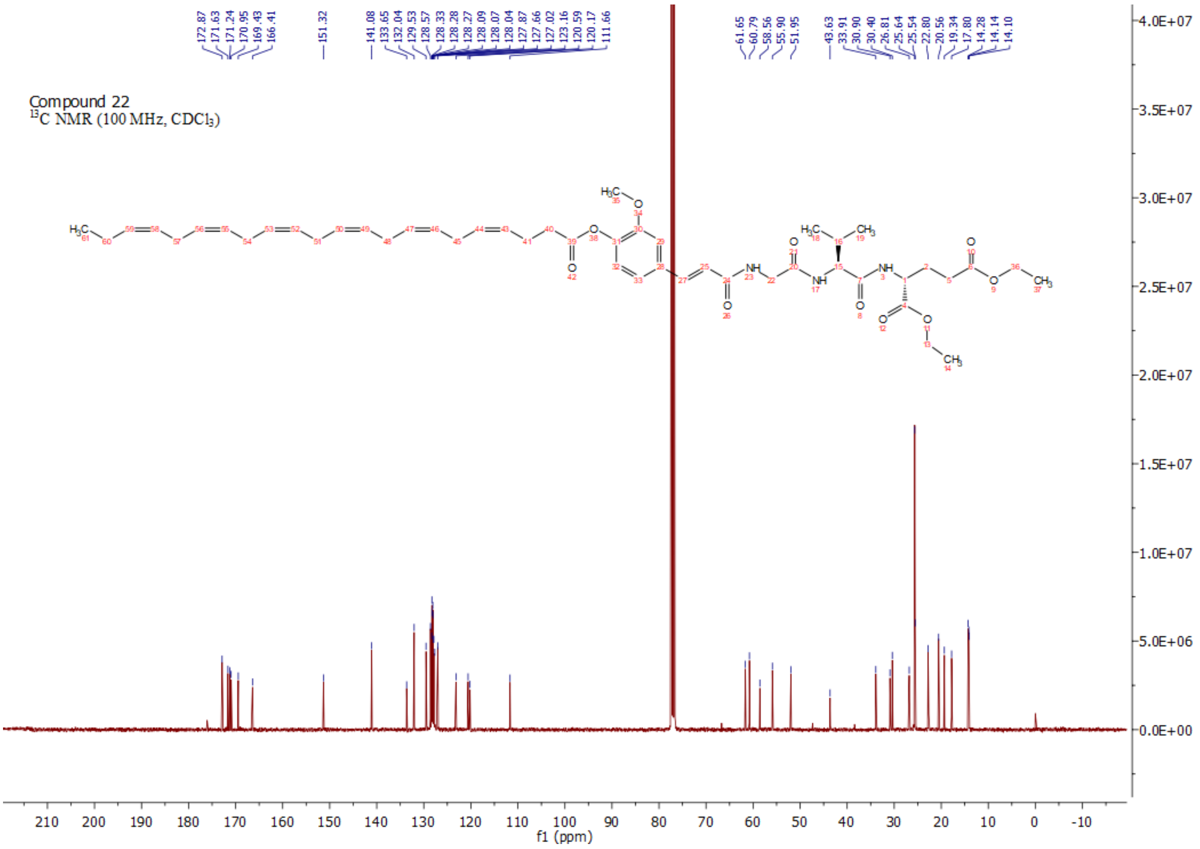
Compound 22
^{13}C NMR (100 MHz, CDCl$_3$)
172.87 171.63 171.24 170.95 169.43 166.41 151.32 141.08 133.65 132.04 129.53 128.57 128.33 128.28 128.27 128.09 128.07 128.04 127.87 127.66 127.02 123.16 120.59 120.17 111.66
61.65 60.79 58.56 55.90 51.95 43.63 33.91 30.90 30.40 26.81 25.64 25.54 22.80 20.56 19.34 17.80 14.28 14.14 14.10
f1 (ppm)

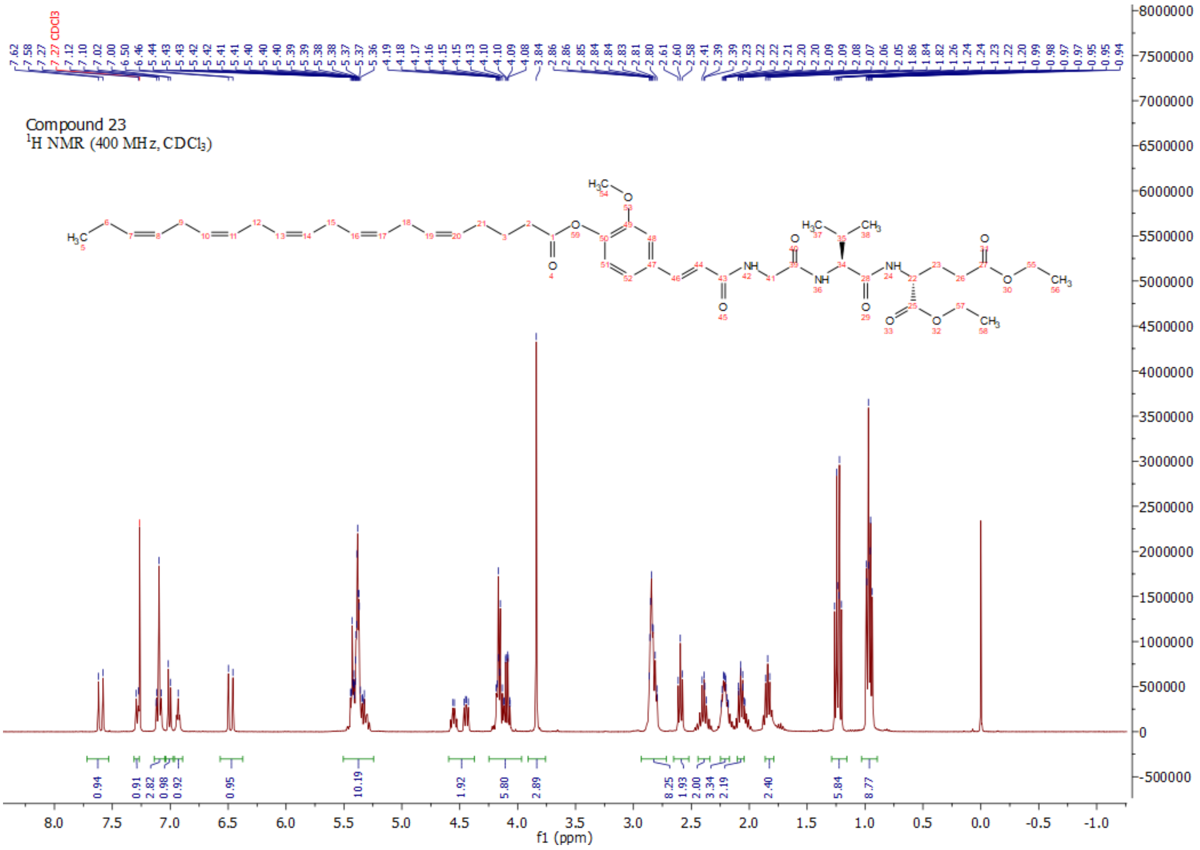
Compound 23
^{1}H NMR (400 MHz, CDCl$_3$)
7.27 CDCl3
f1 (ppm)

Compound 23
^{13}C NMR (100 MHz, $CDCl_3$)

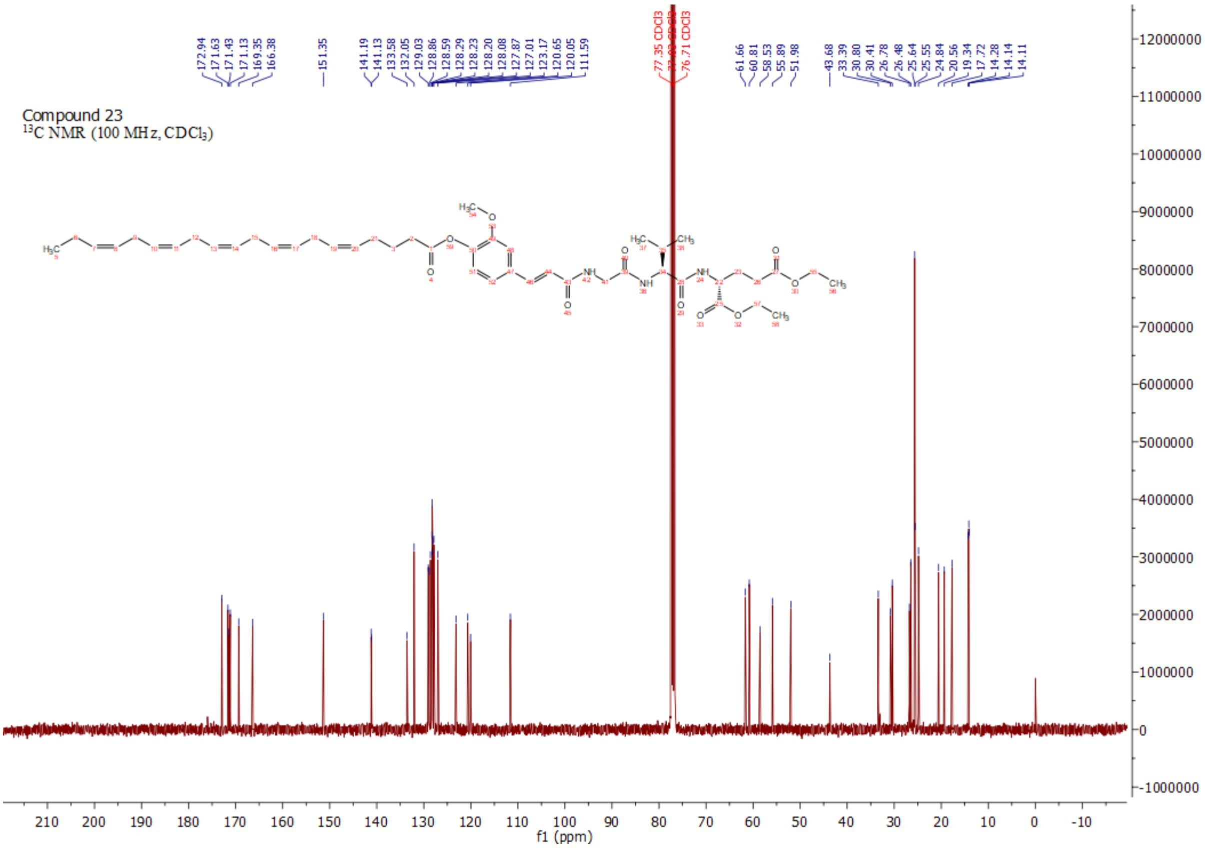

Compound 24
^{1}H NMR (400 MHz, $CDCl_3$)

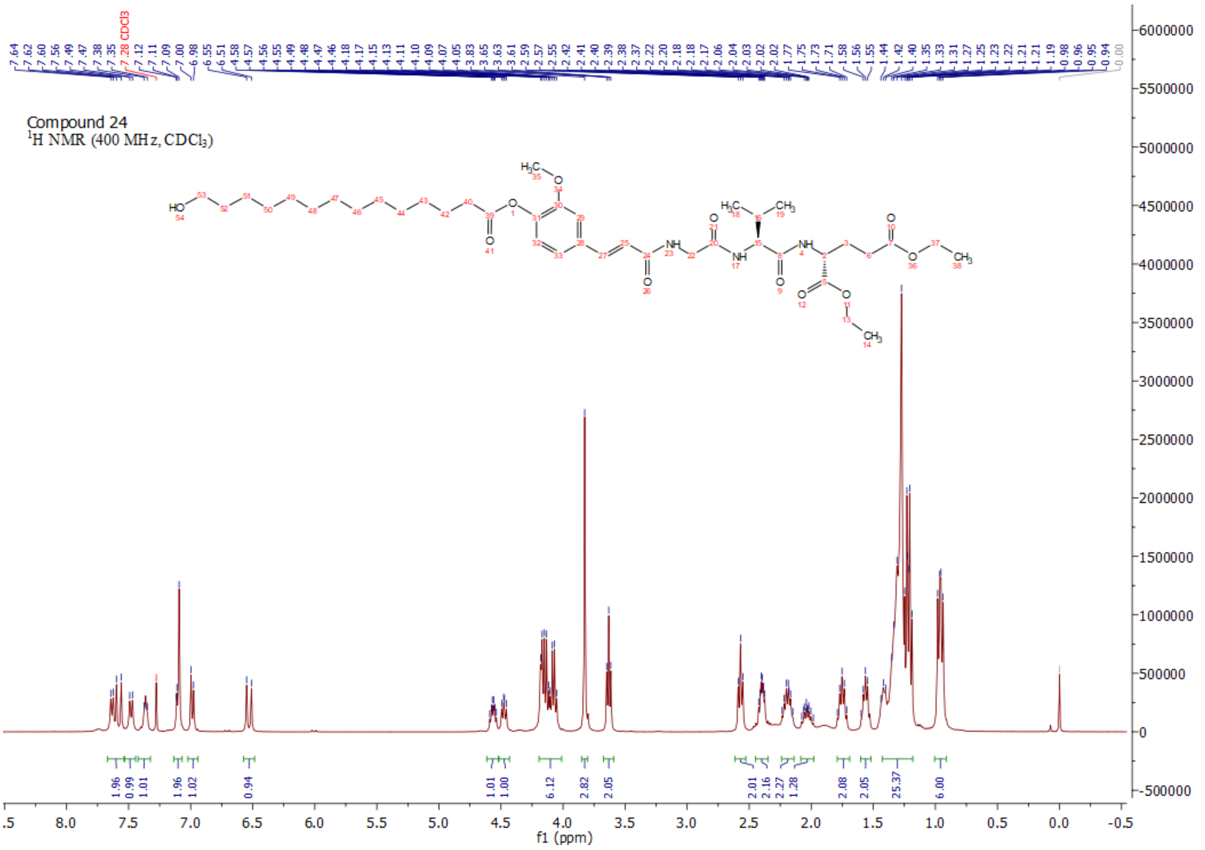

Compound 24
^{13}C NMR (100 MHz, $CDCl_3$)

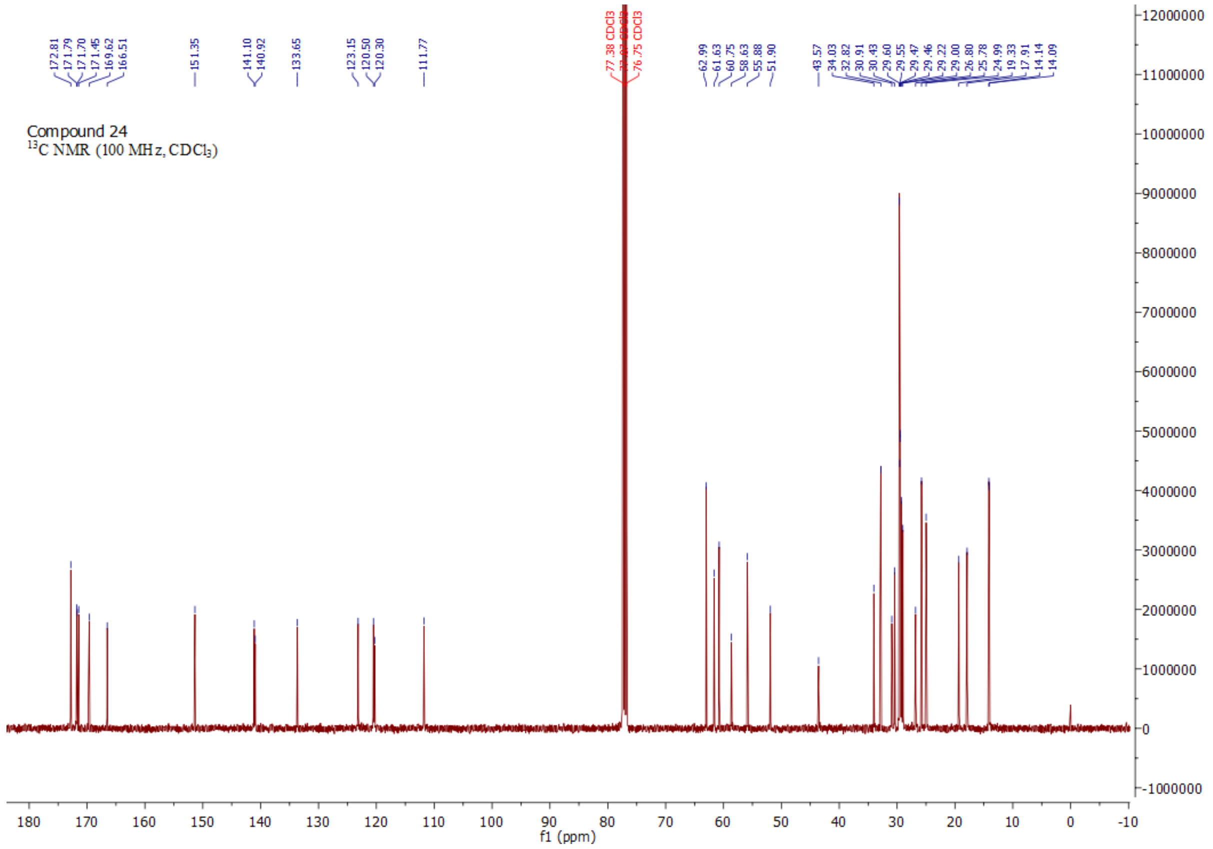

Compound 26
^{1}H NMR (400 MHz, $CDCl_3$)

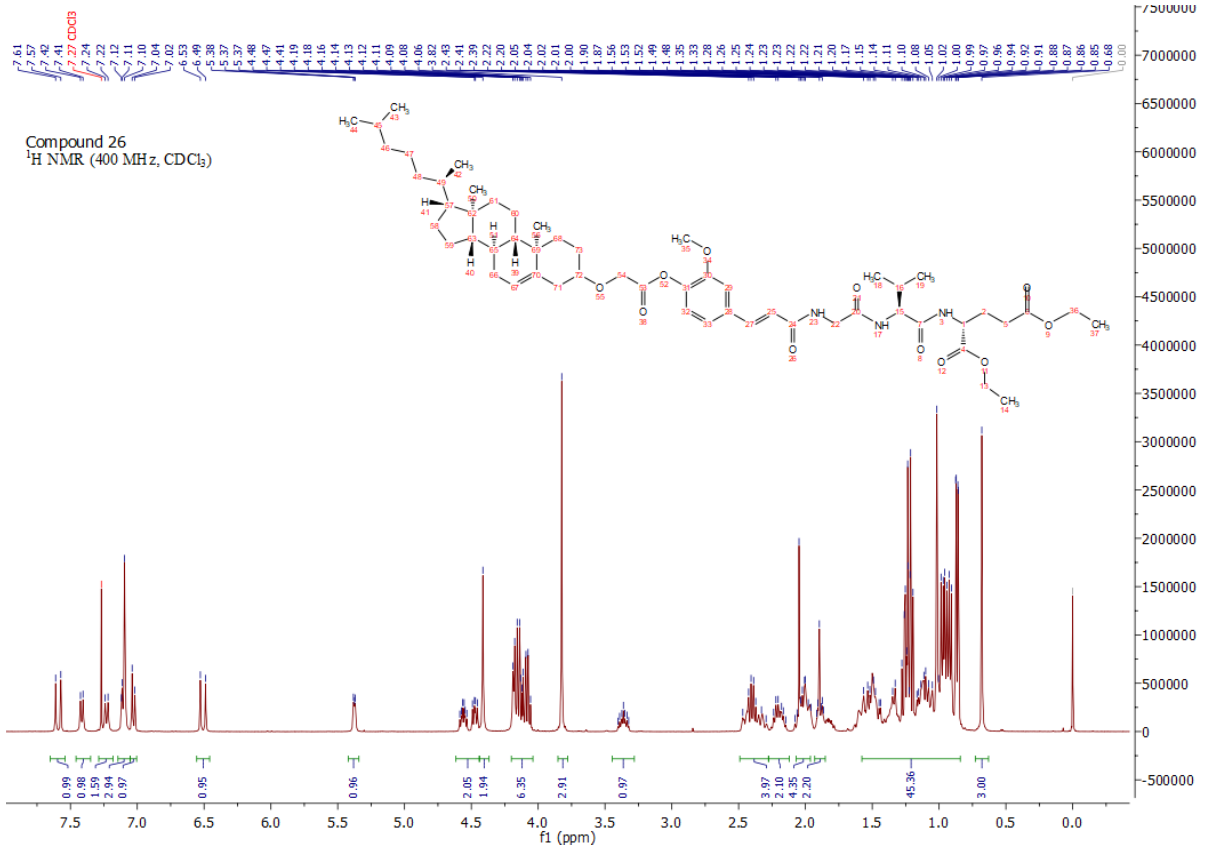

Compound 26
^{13}C NMR (100 MHz, $CDCl_3$)

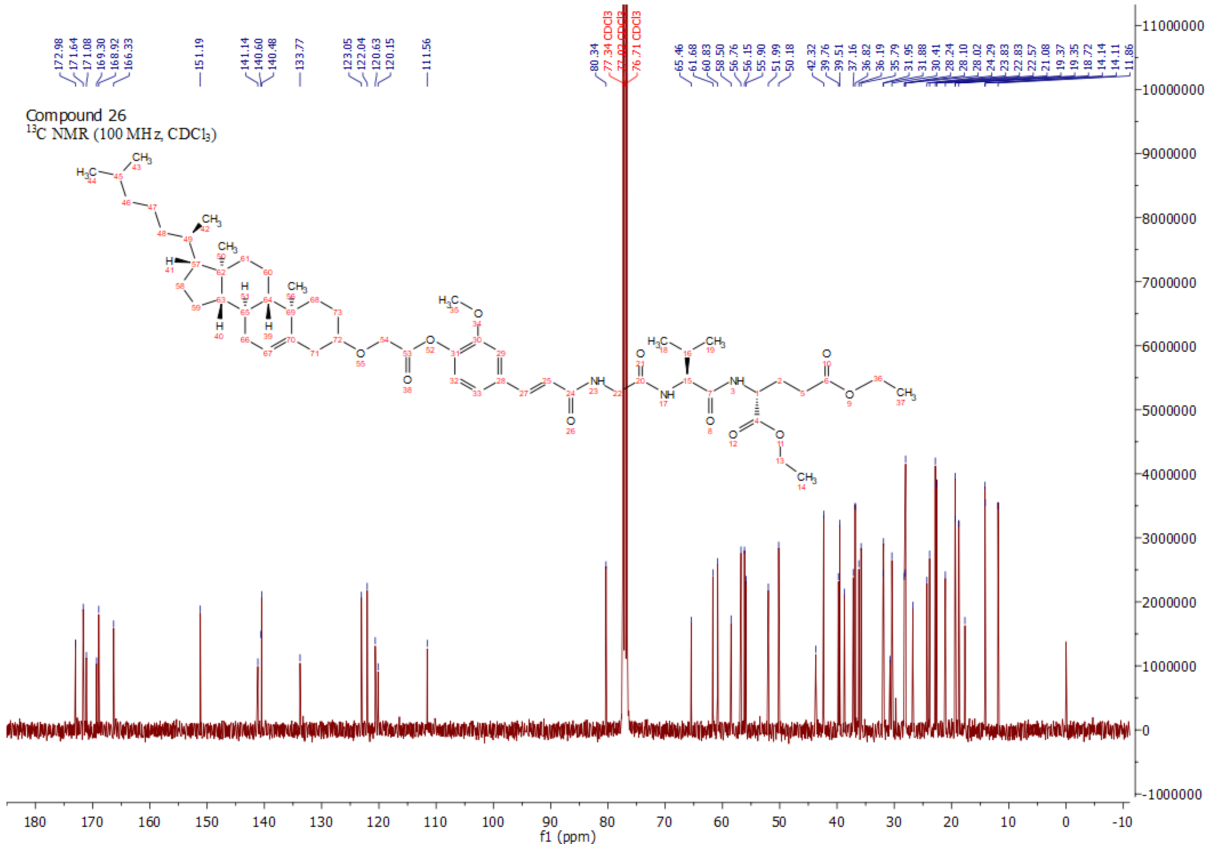

Compound 28
^{1}H NMR (400 MHz, DMSO)

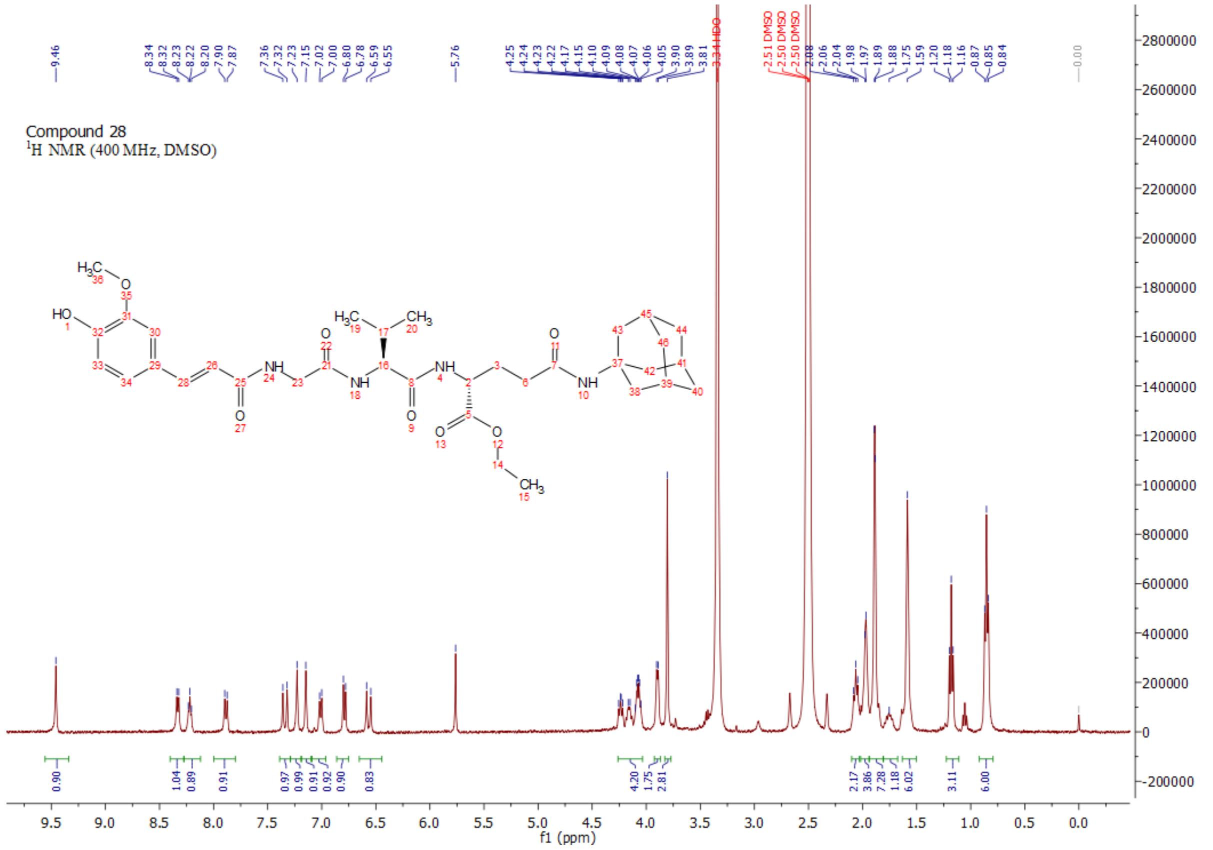

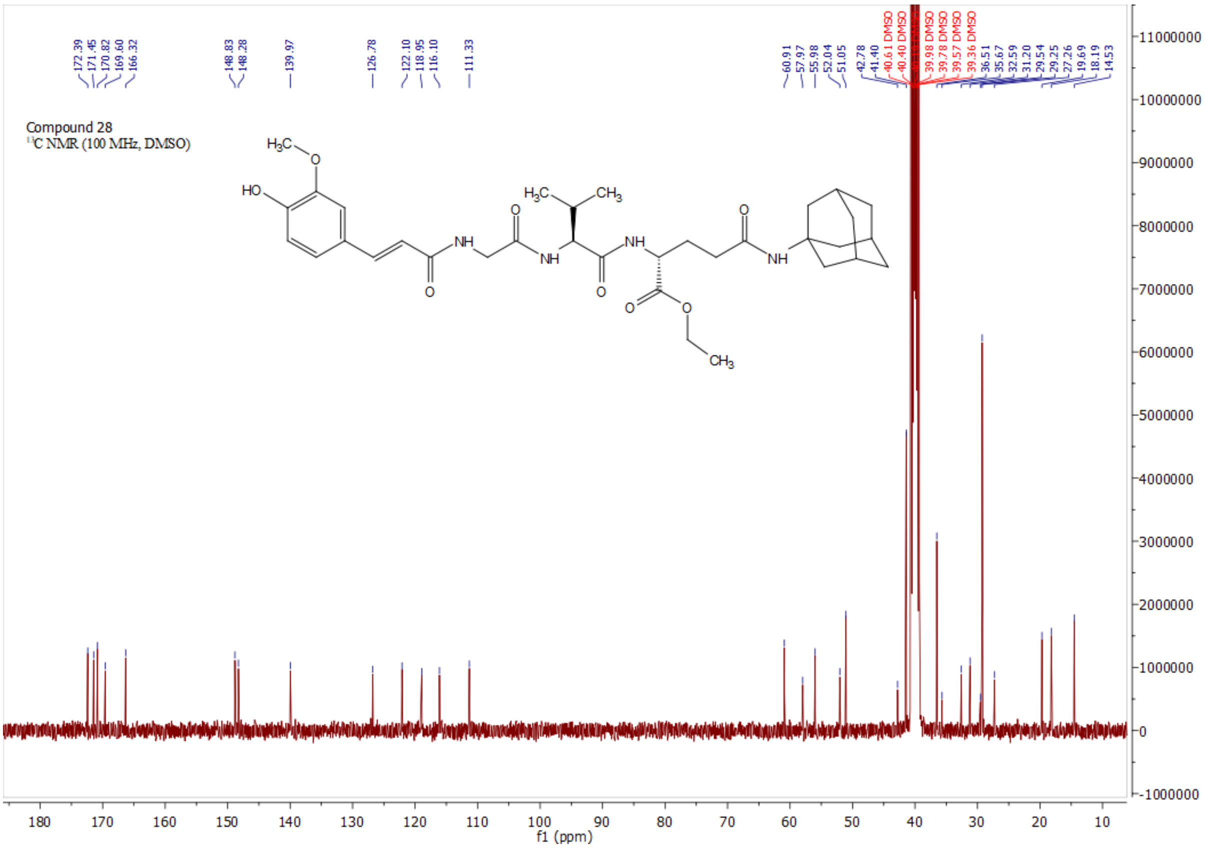
Compound 28
13C NMR (100 MHz, DMSO)
f1 (ppm)

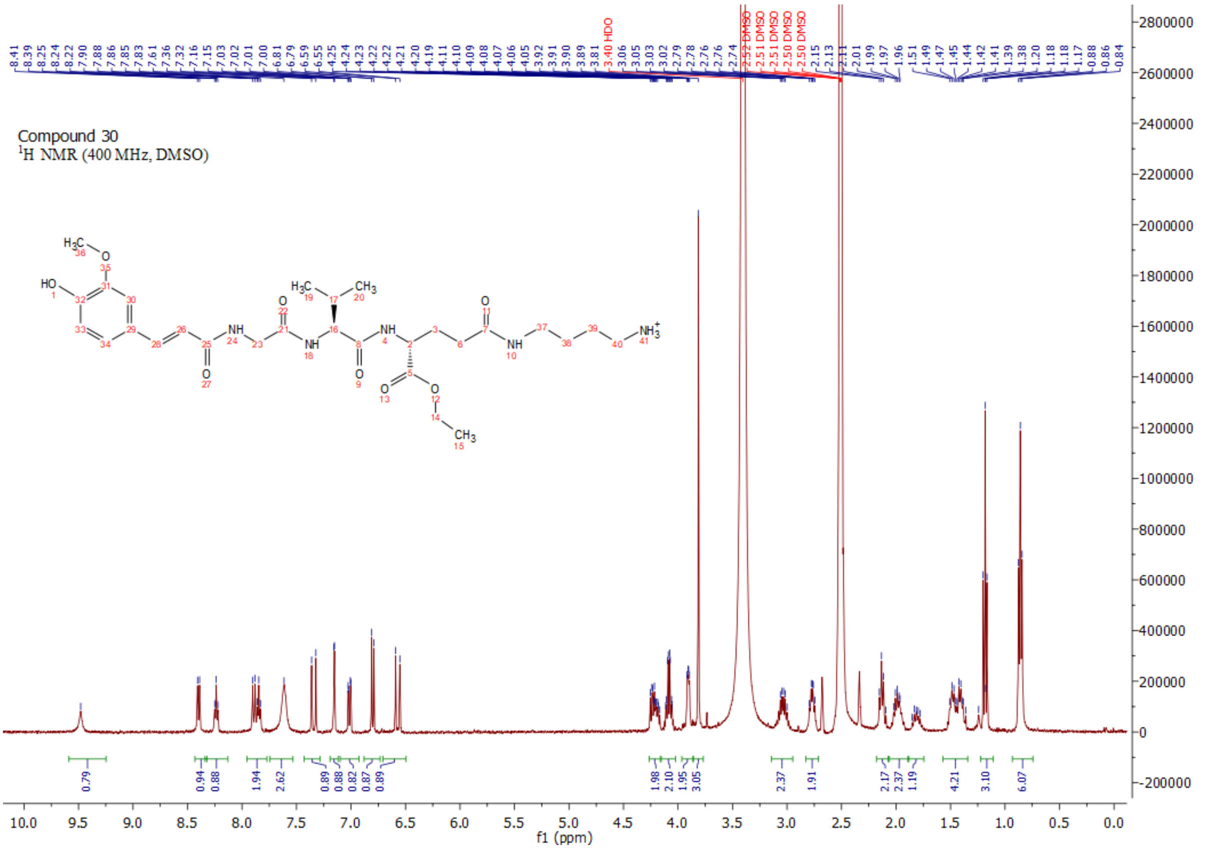
Compound 30
1H NMR (400 MHz, DMSO)
f1 (ppm)

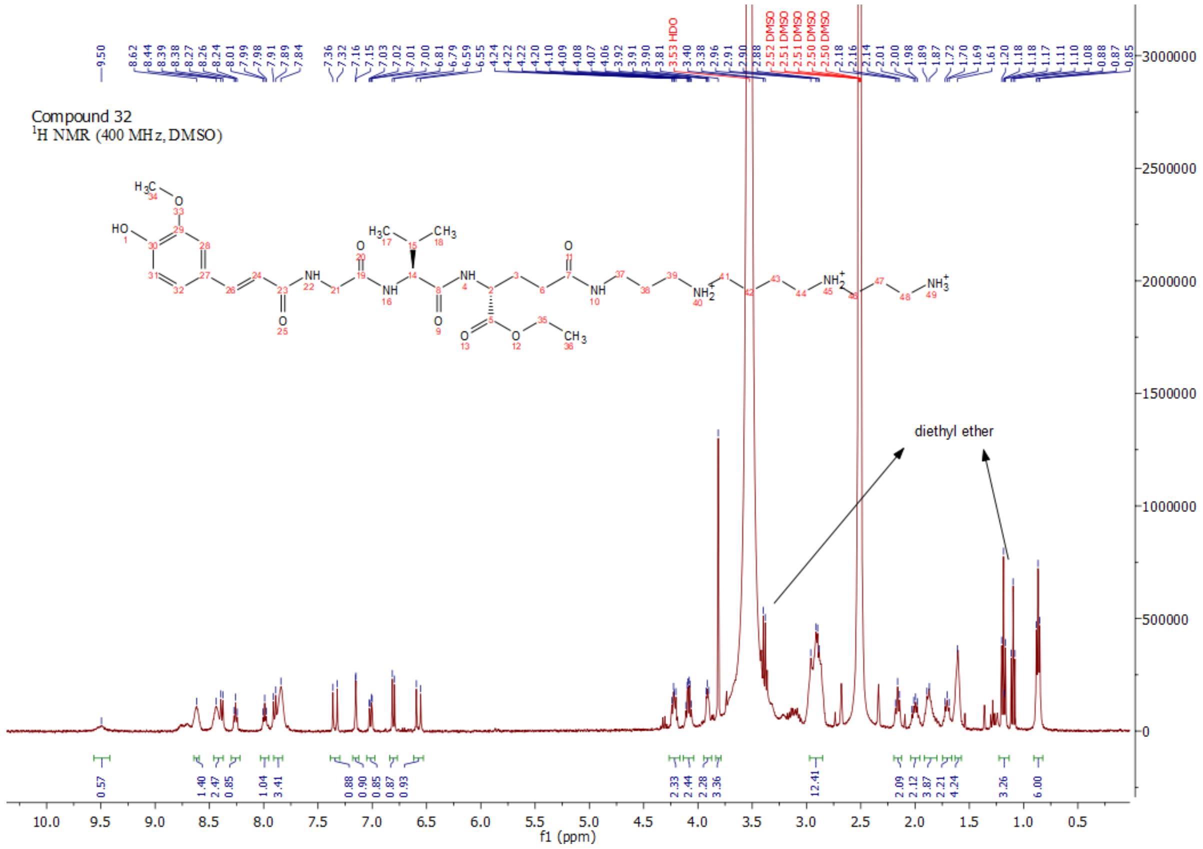
Compound 32
^{1}H NMR (400 MHz, DMSO)
diethyl ether
f1 (ppm)

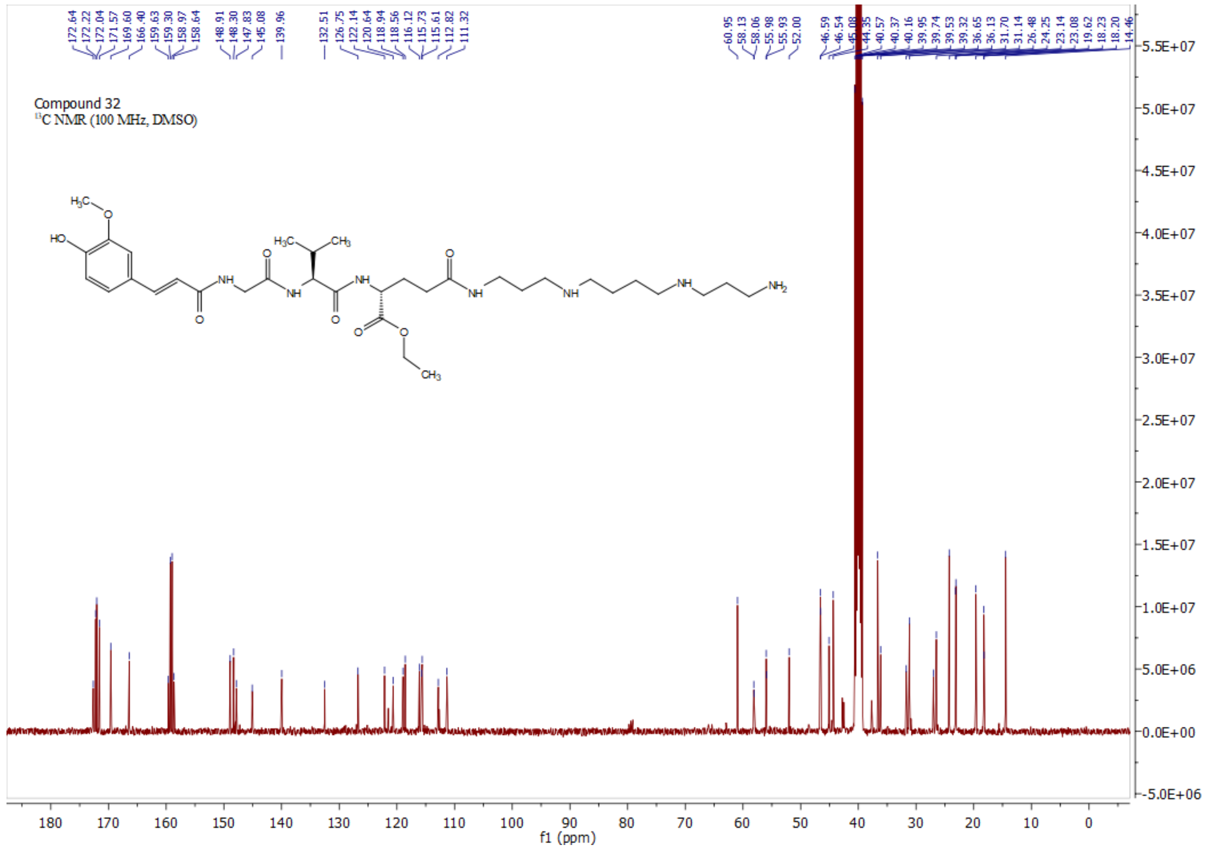
Compound 32
^{13}C NMR (100 MHz, DMSO)
f1 (ppm)

Compound 34
^{1}H NMR (400 MHz, DMSO)

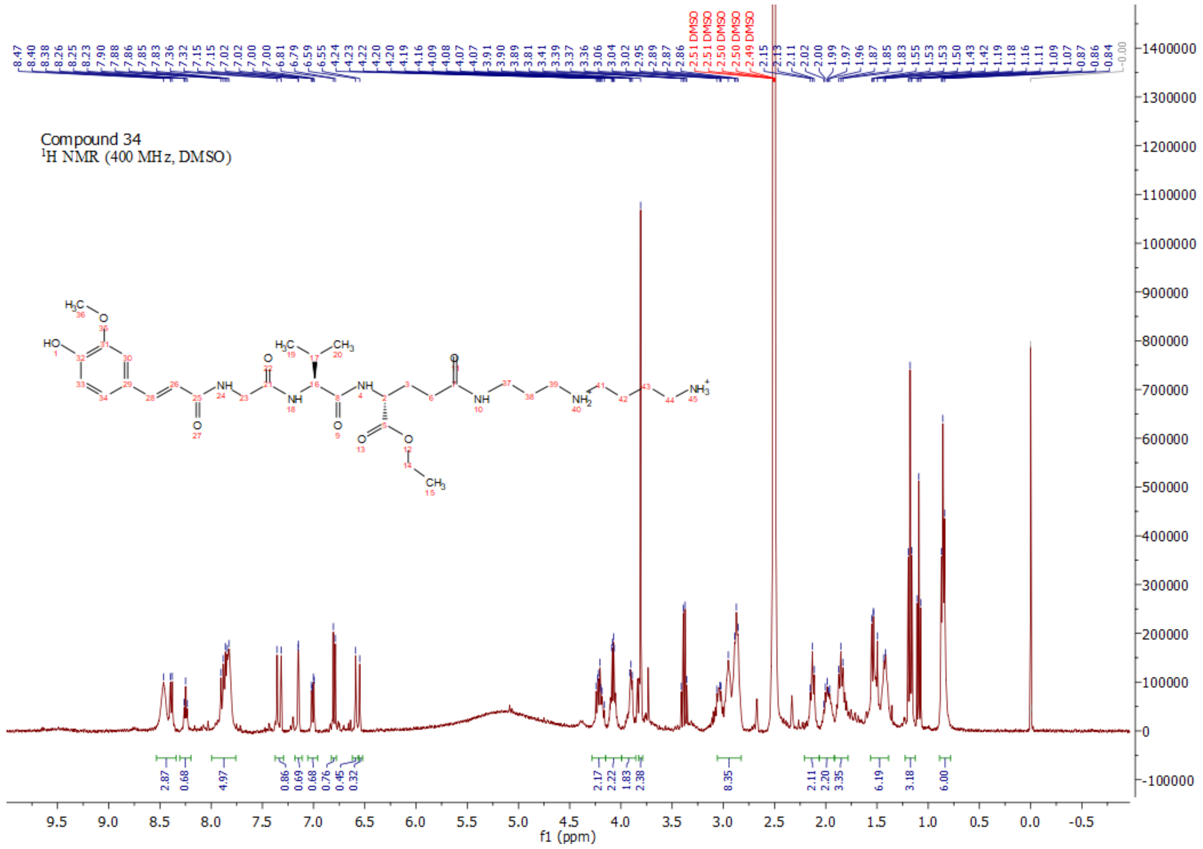

Compound 35
^{1}H NMR (400 MHz, $CDCl_3$)

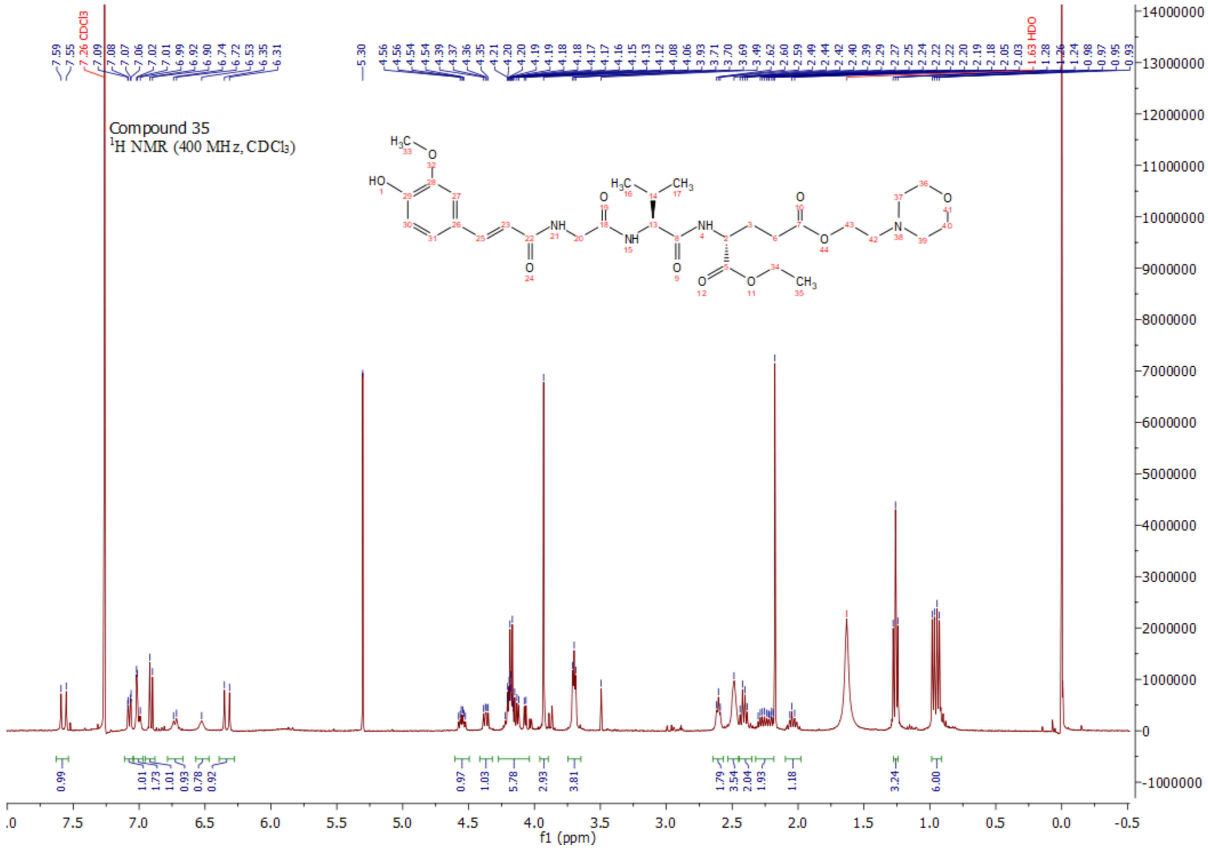

Compound 37
^{1}H NMR (400 MHz, CDCl$_3$)

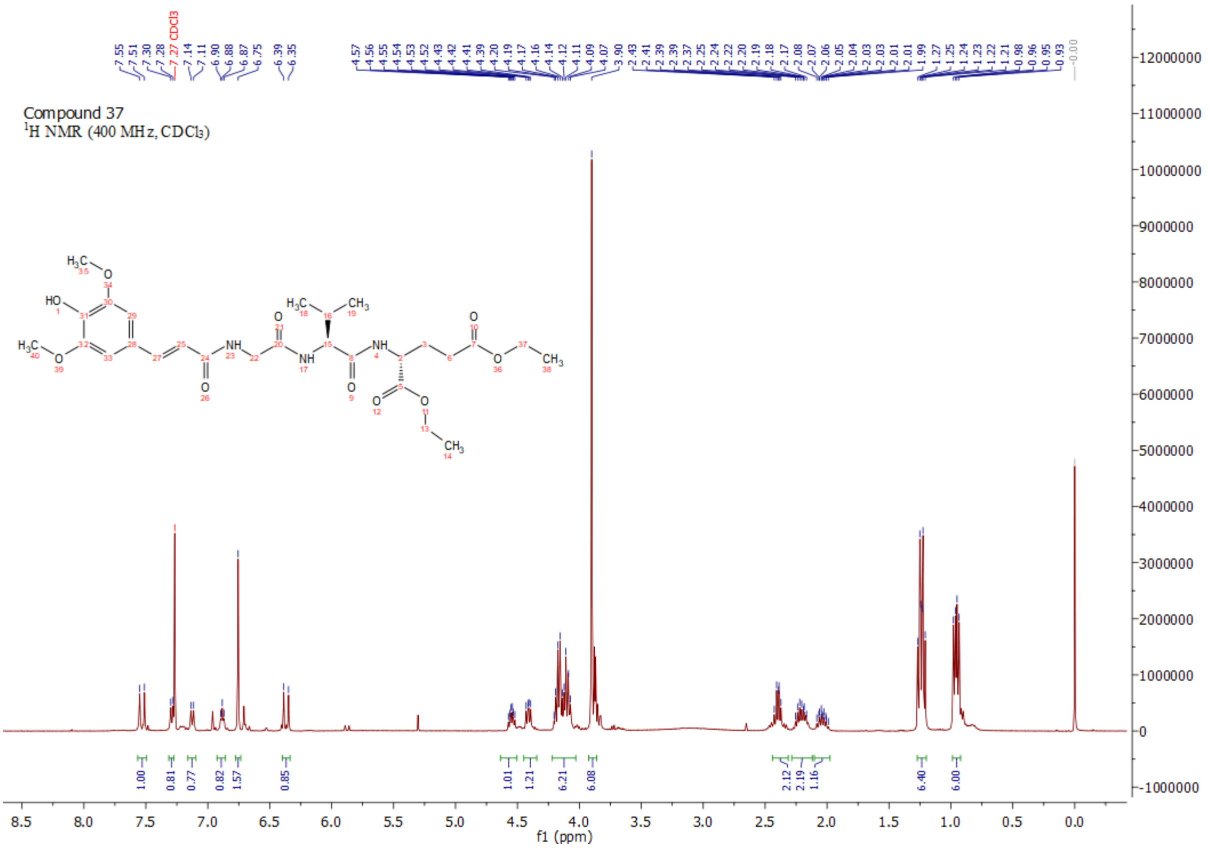

Compound 37
^{13}C NMR (100 MHz, CDCl$_3$)

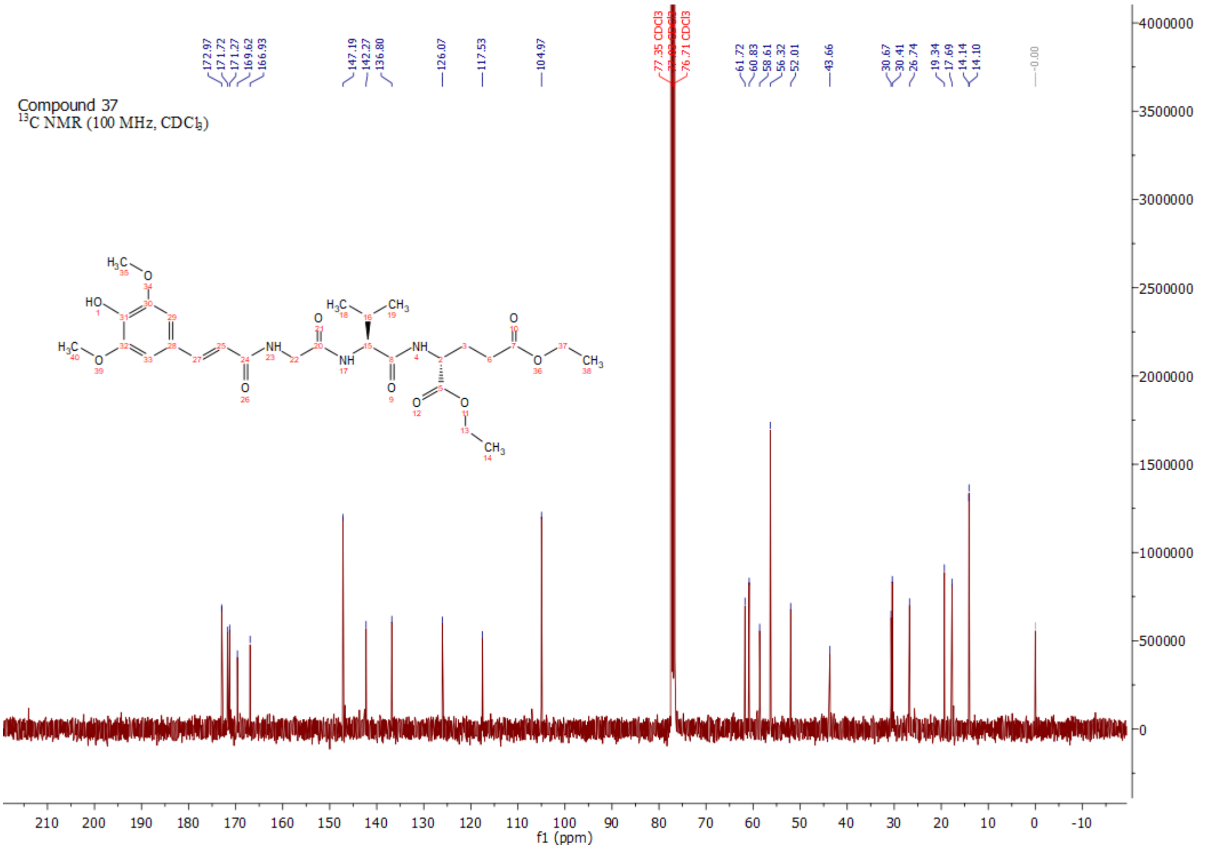

4. COSY spectra of representative compounds

Compound 3
^1H - ^1H COSY, $CDCl_3$

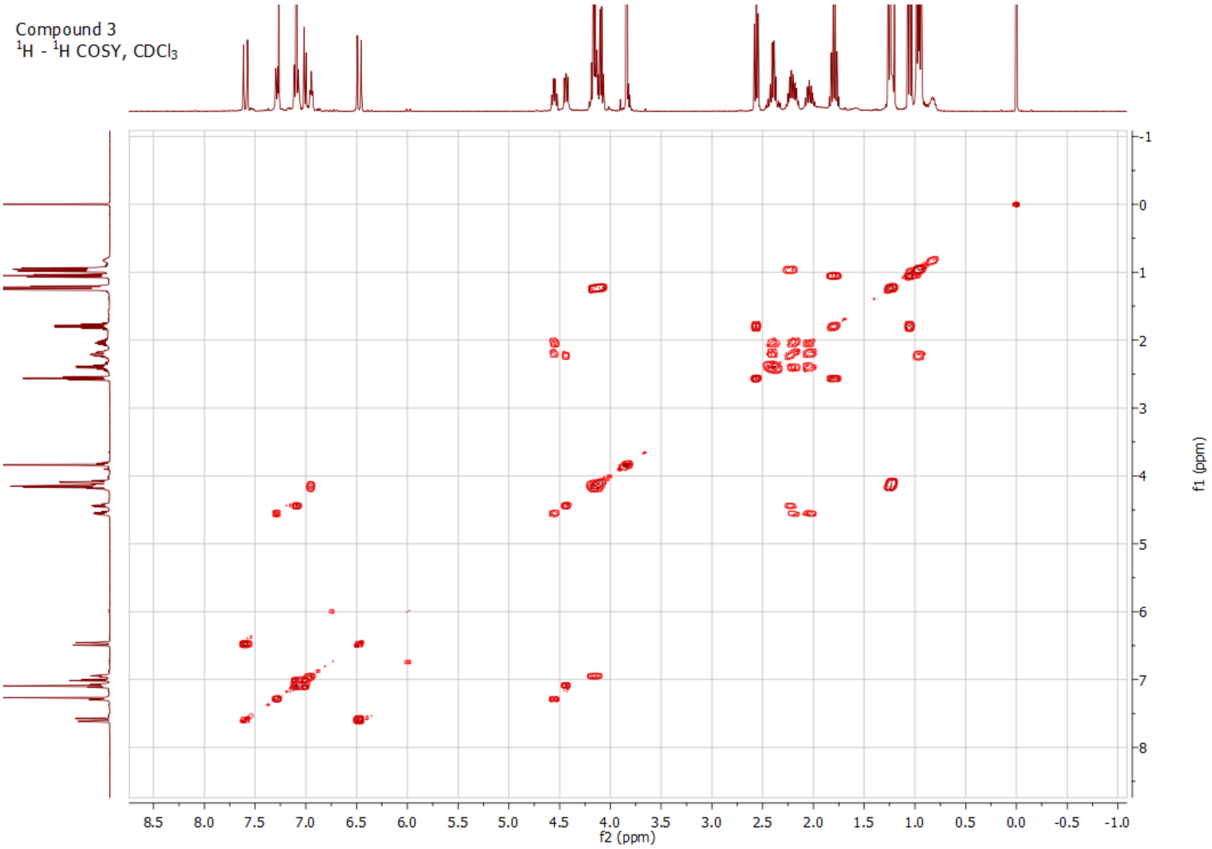

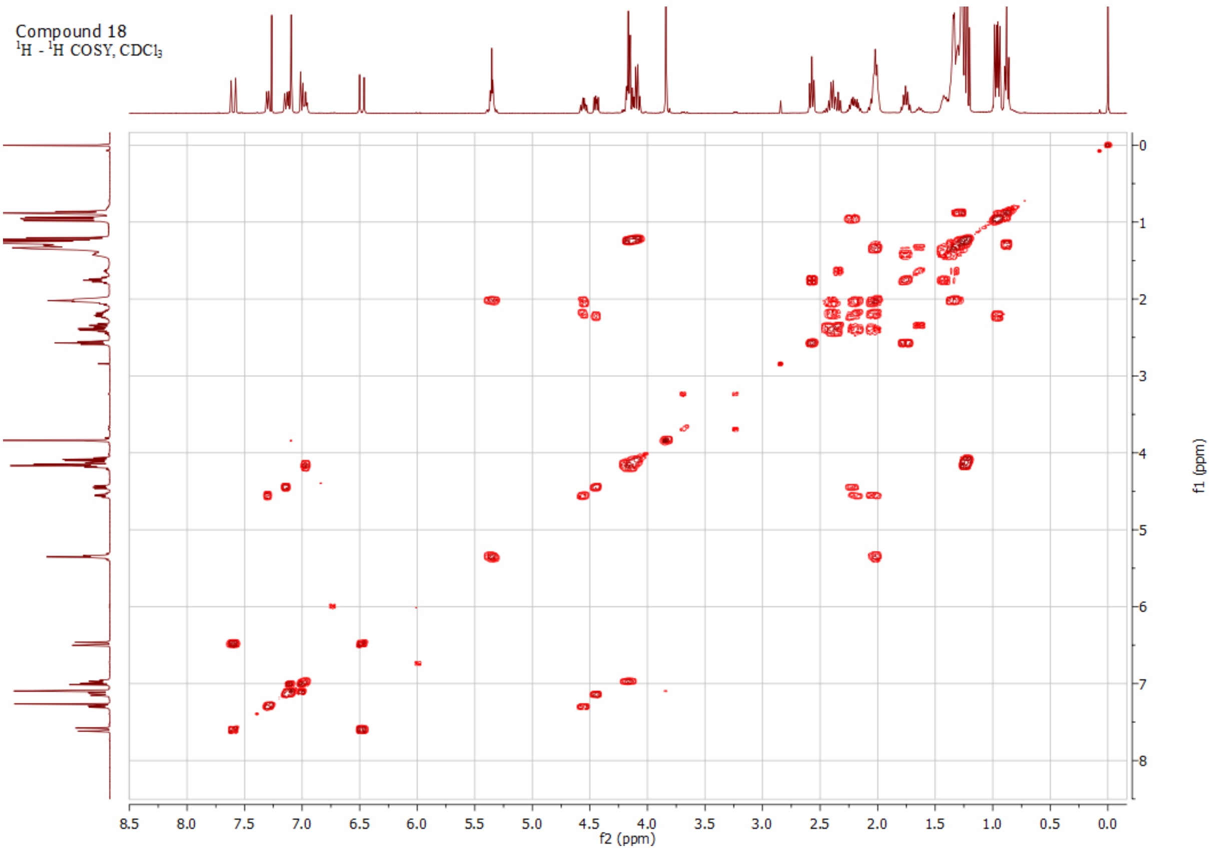
Compound 18
^{1}H - ^{1}H COSY, $CDCl_3$
f2 (ppm)
f1 (ppm)

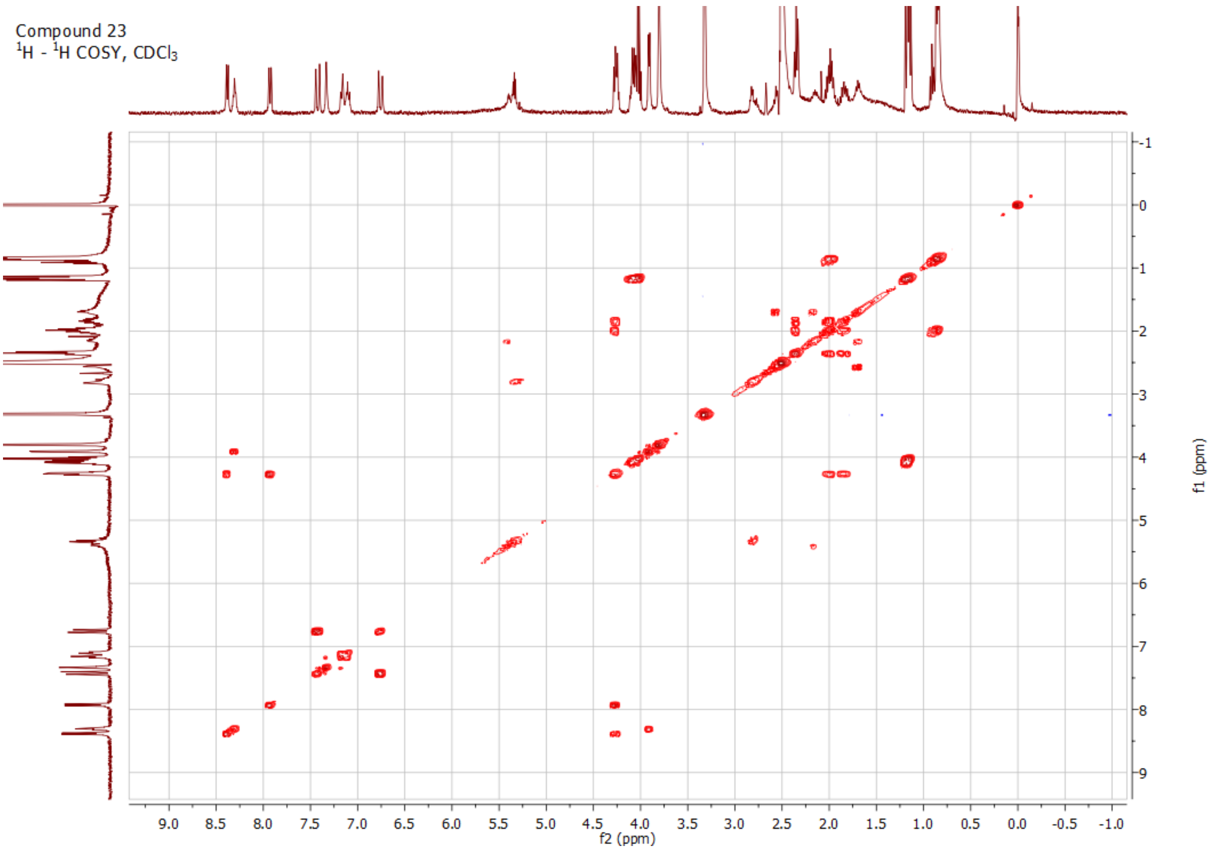
Compound 23
^{1}H - ^{1}H COSY, $CDCl_3$
f2 (ppm)
f1 (ppm)

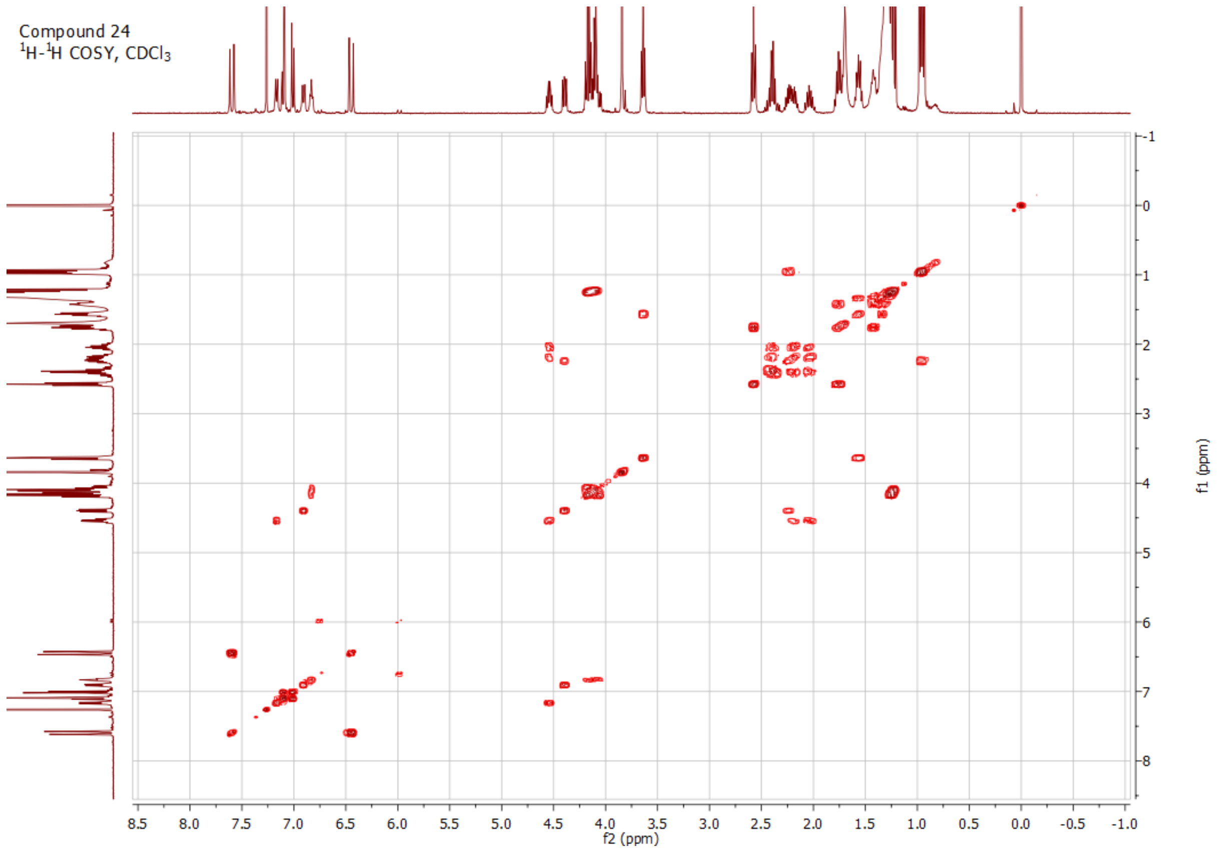
Compound 24
^{1}H-^{1}H COSY, CDCl$_3$
f2 (ppm)
f1 (ppm)

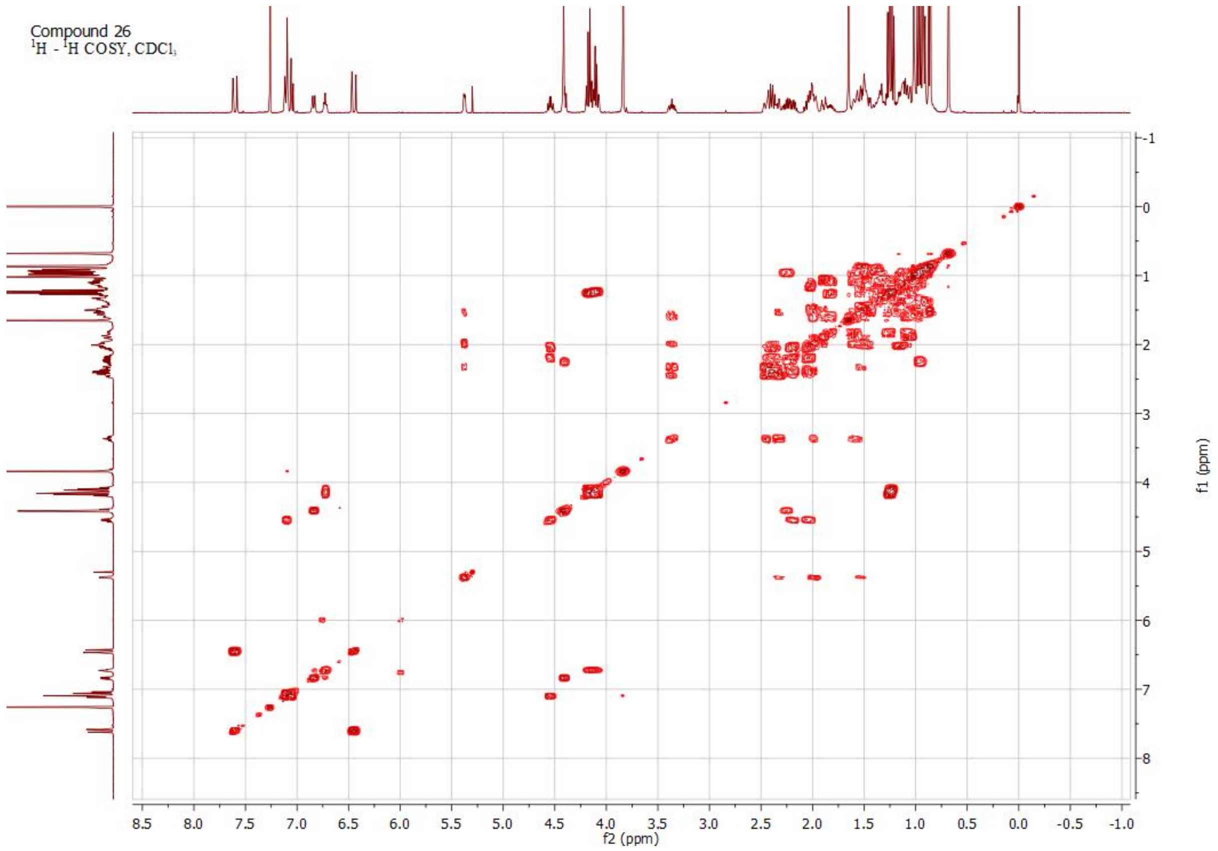
Compound 26
^{1}H - ^{1}H COSY, CDCl$_3$
f2 (ppm)
f1 (ppm)

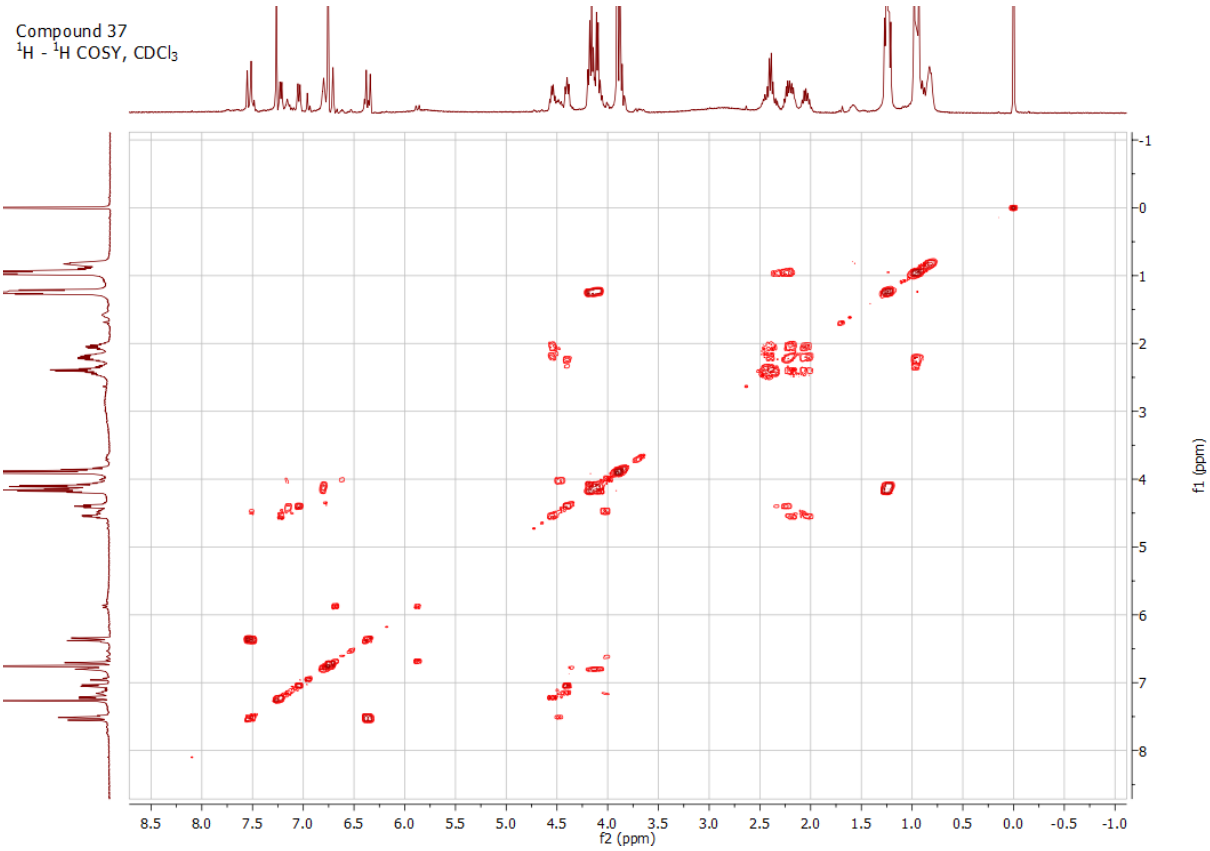

Compound 37
^1H - ^1H COSY, $CDCl_3$
f2 (ppm)
f1 (ppm)

5. HRMS spectra of tested compounds

Compound **3**

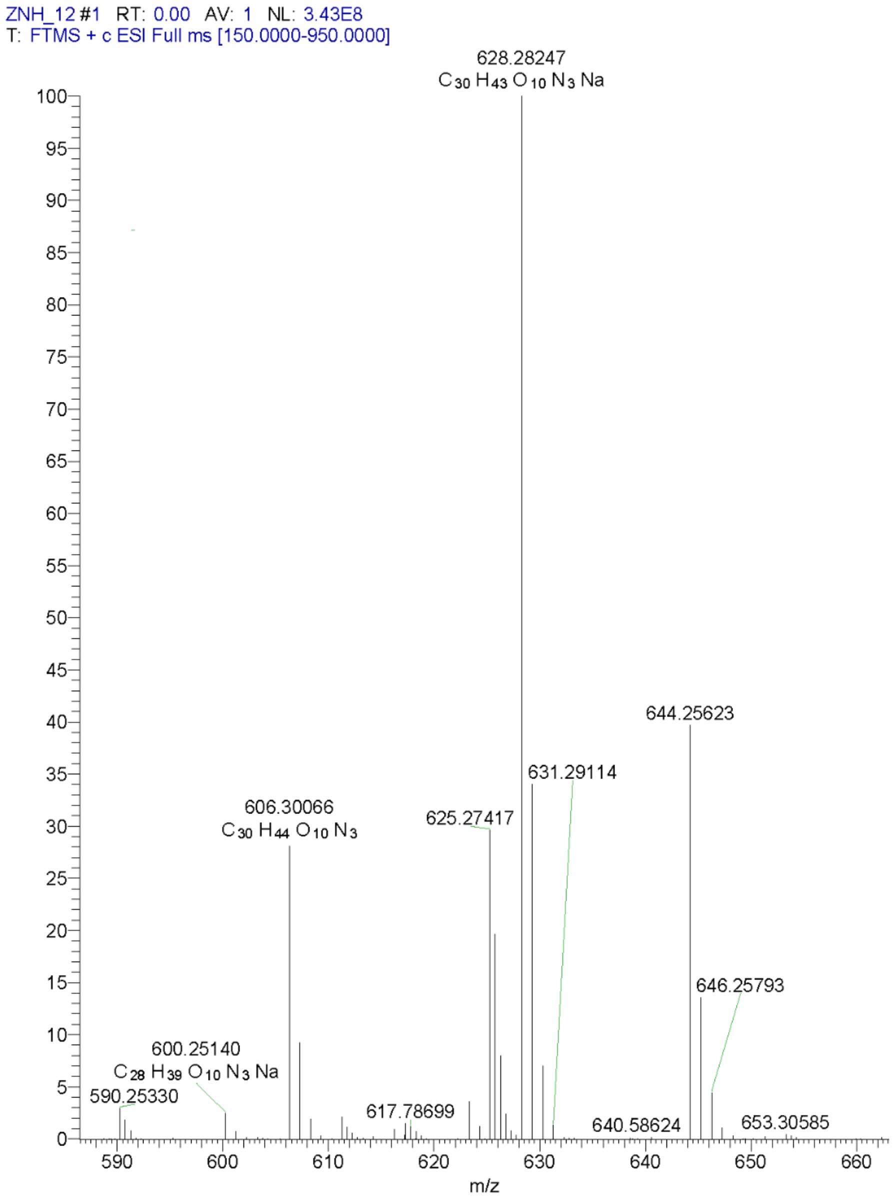

Elemental composition search on mass 606.30066

m/z= 601.30066-611.30066

m/z	Theo. Mass	Delta (ppm)	RDB equiv.	Composition
606.30066	606.30212	-2.41	10.5	$C_{30}H_{44}O_{10}N_3$

Compound **4**

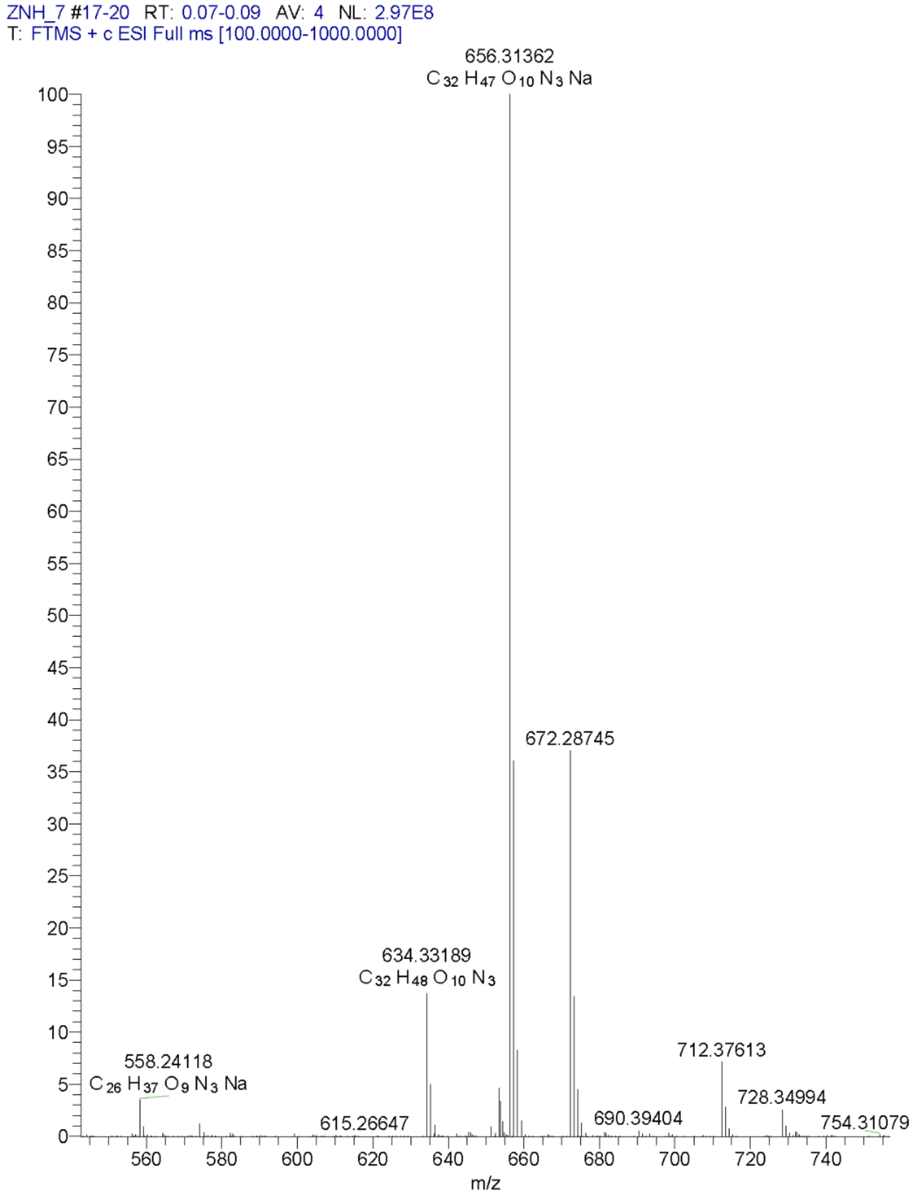

Elemental composition search on mass 634.33189

m/z= 629.33189-639.33189

m/z	Theo. Mass	Delta (ppm)	RDB equiv.	Composition
634.33189	634.33342	-2.41	10.5	$C_{32}H_{48}O_{10}N_3$

Compound **5**

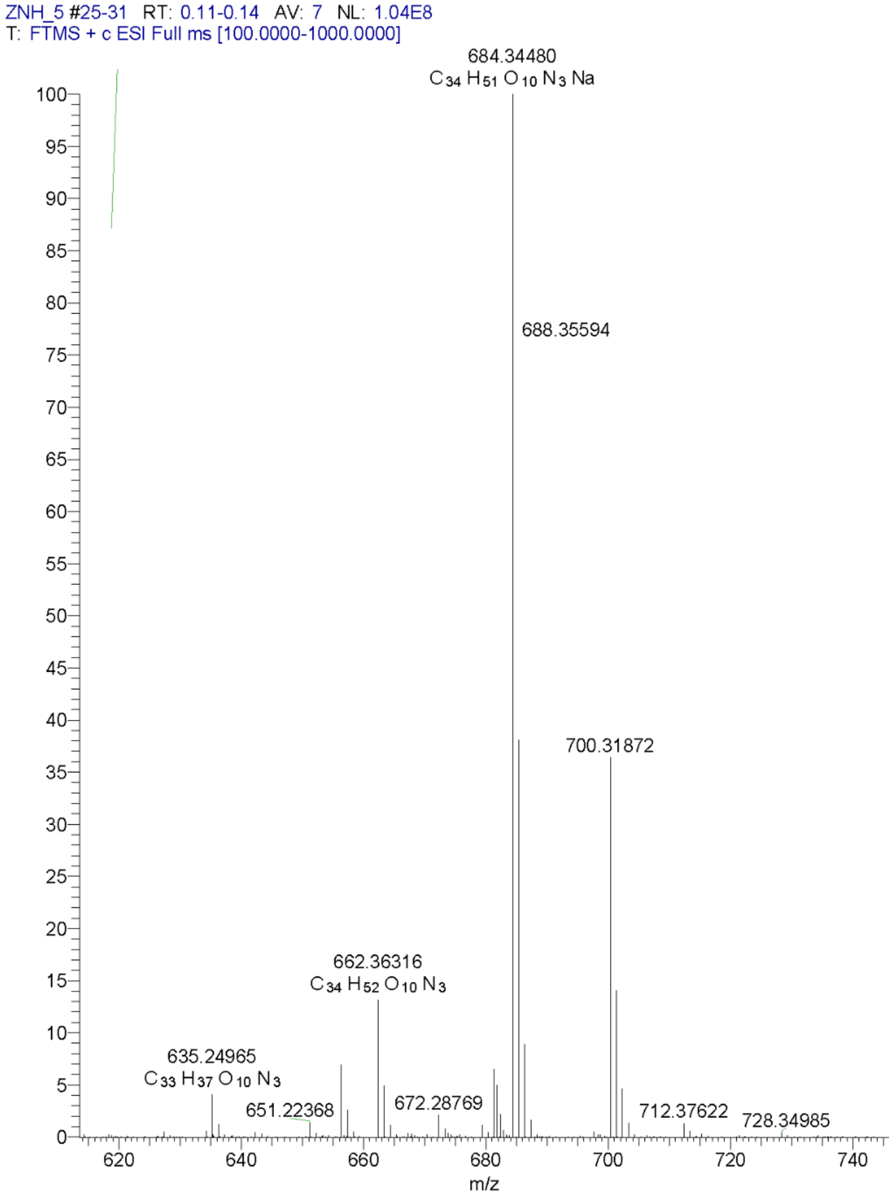

Elemental composition search on mass 662.36316

m/z= 657.36316-667.36316

m/z	Theo. Mass	Delta (ppm)	RDB equiv.	Composition
662.36316	662.36472	-2.36	10.5	$C_{34}H_{52}O_{10}N_3$

Compound **6**

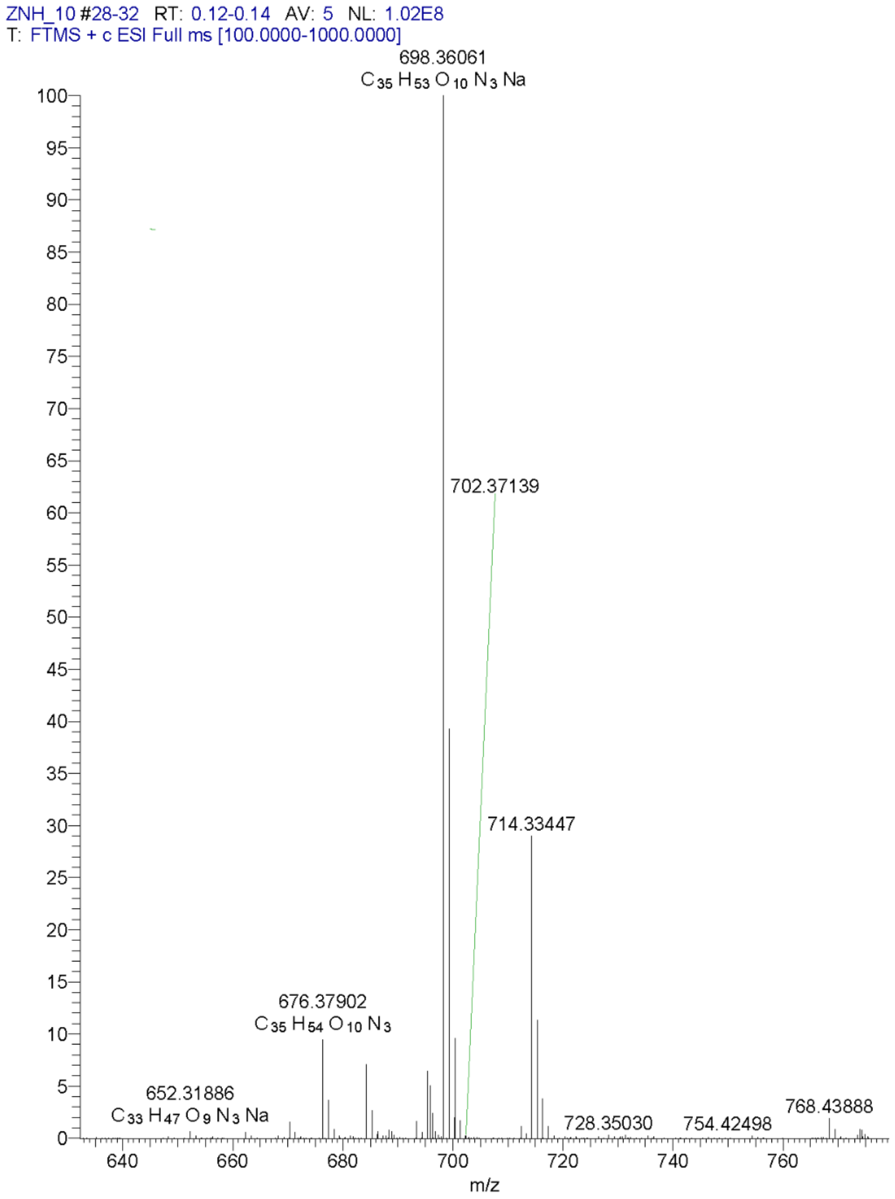

Elemental composition search on mass 676.37902

m/z= 671.37902-681.37902

m/z	Theo. Mass	Delta (ppm)	RDB equiv.	Composition
676.37902	676.38037	-2.00	10.5	$C_{35}H_{54}O_{10}N_3$

Compound **7**

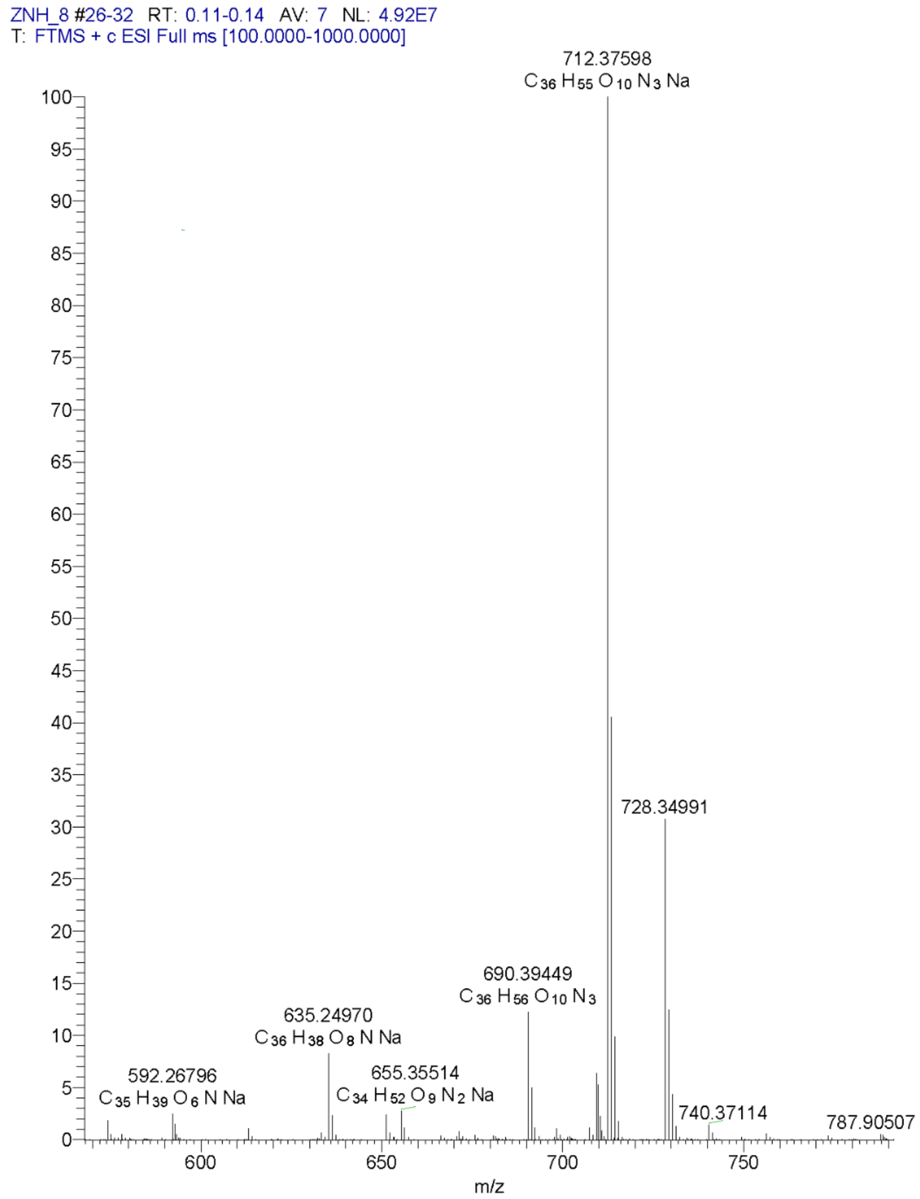

Elemental composition search on mass 690.39449

m/z= 685.39449-695.39449

m/z	Theo. Mass	Delta (ppm)	RDB equiv.	Composition
690.39449	690.39602	-2.22	10.5	$C_{36}H_{56}O_{10}N_3$

Compound **8**

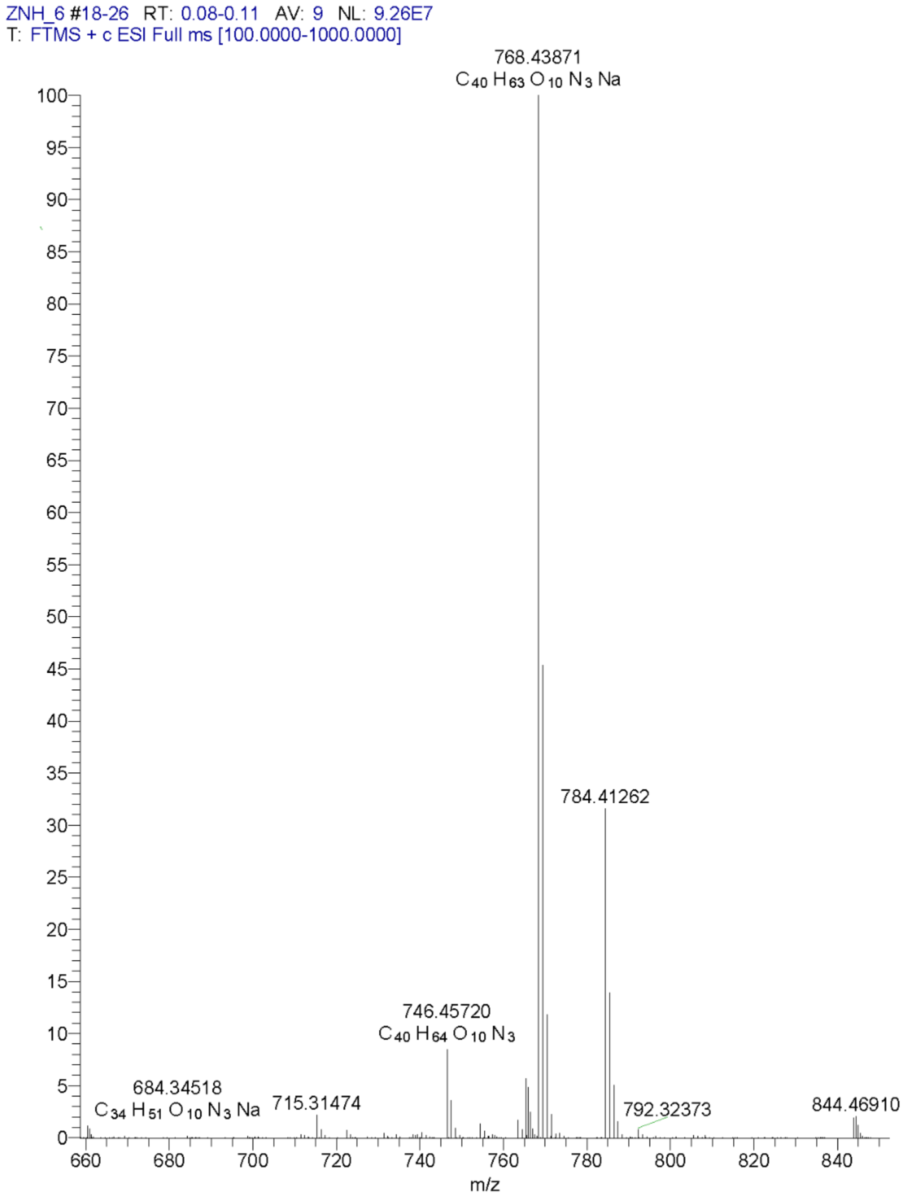

Elemental composition search on mass 746.45720

m/z= 741.45720-751.45720

m/z	Theo. Mass	Delta (ppm)	RDB equiv.	Composition
746.45720	746.45862	-1.90	10.5	$C_{40}H_{64}O_{10}N_3$

Compound **9**

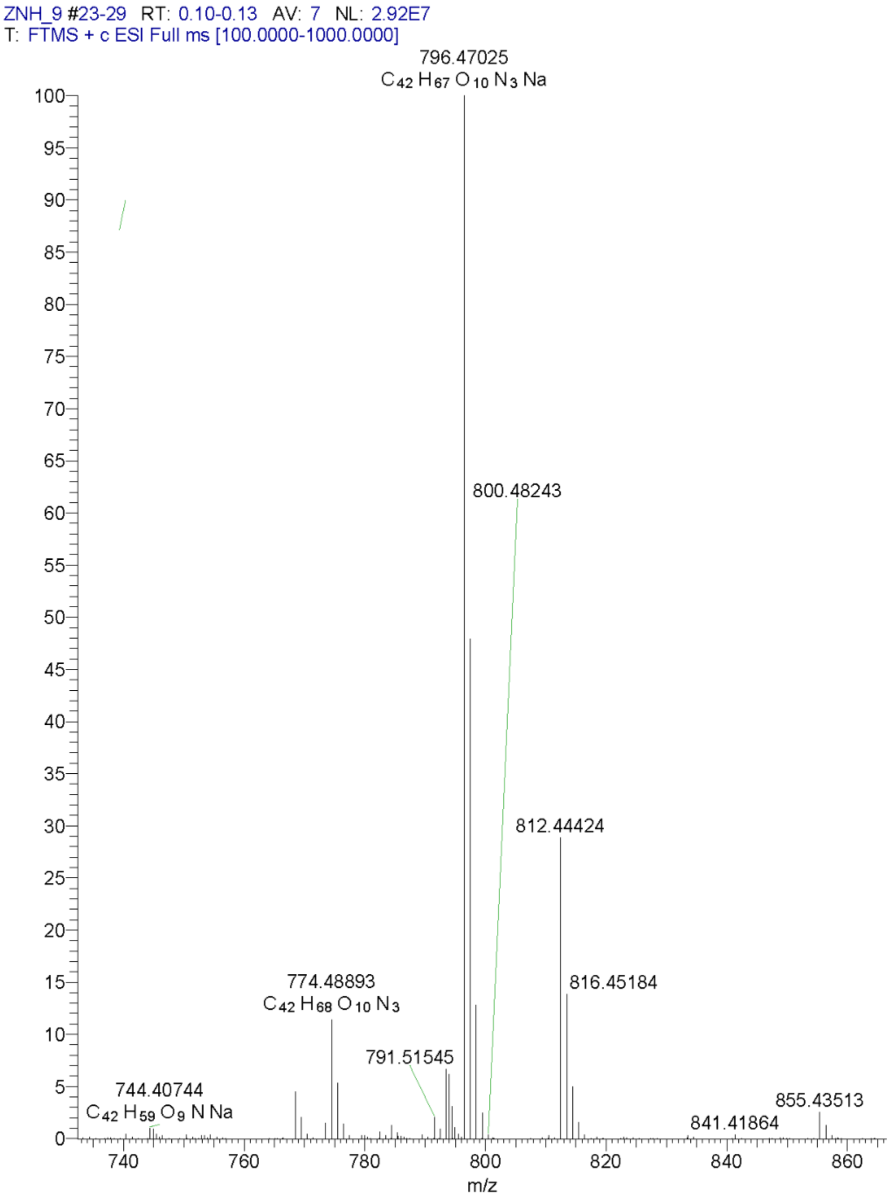

Elemental composition search on mass 774.48893

m/z= 769.48893-779.48893

m/z	Theo. Mass	Delta (ppm)	RDB equiv.	Composition
774.48893	774.48992	-1.28	10.5	$C_{42}H_{68}O_{10}N_3$

Compound **11**

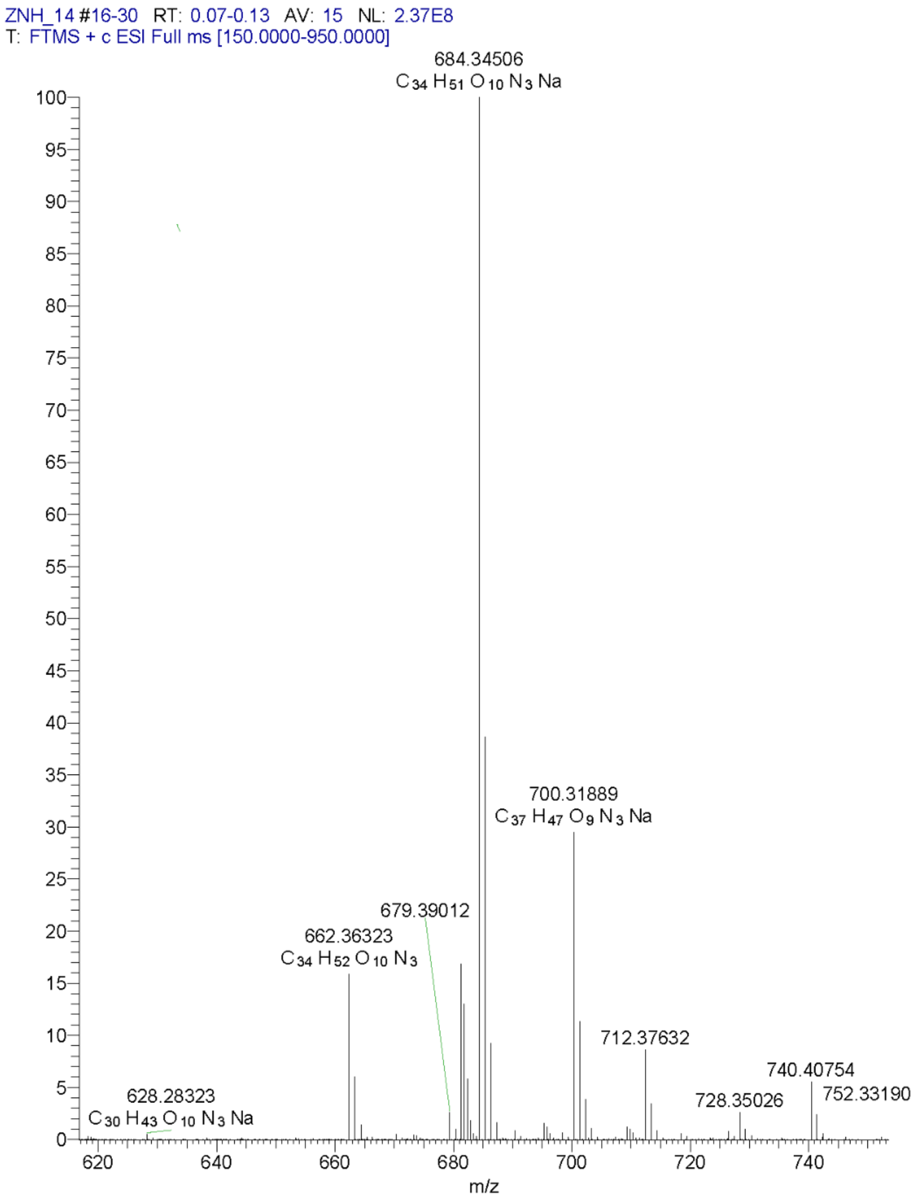

Elemental composition search on mass 662.36323

m/z= 657.36323-667.36323

m/z	Theo. Mass	Delta (ppm)	RDB equiv.	Composition
662.36323	662.36472	-2.25	10.5	$C_{34}H_{52}O_{10}N_3$

Compound **12**

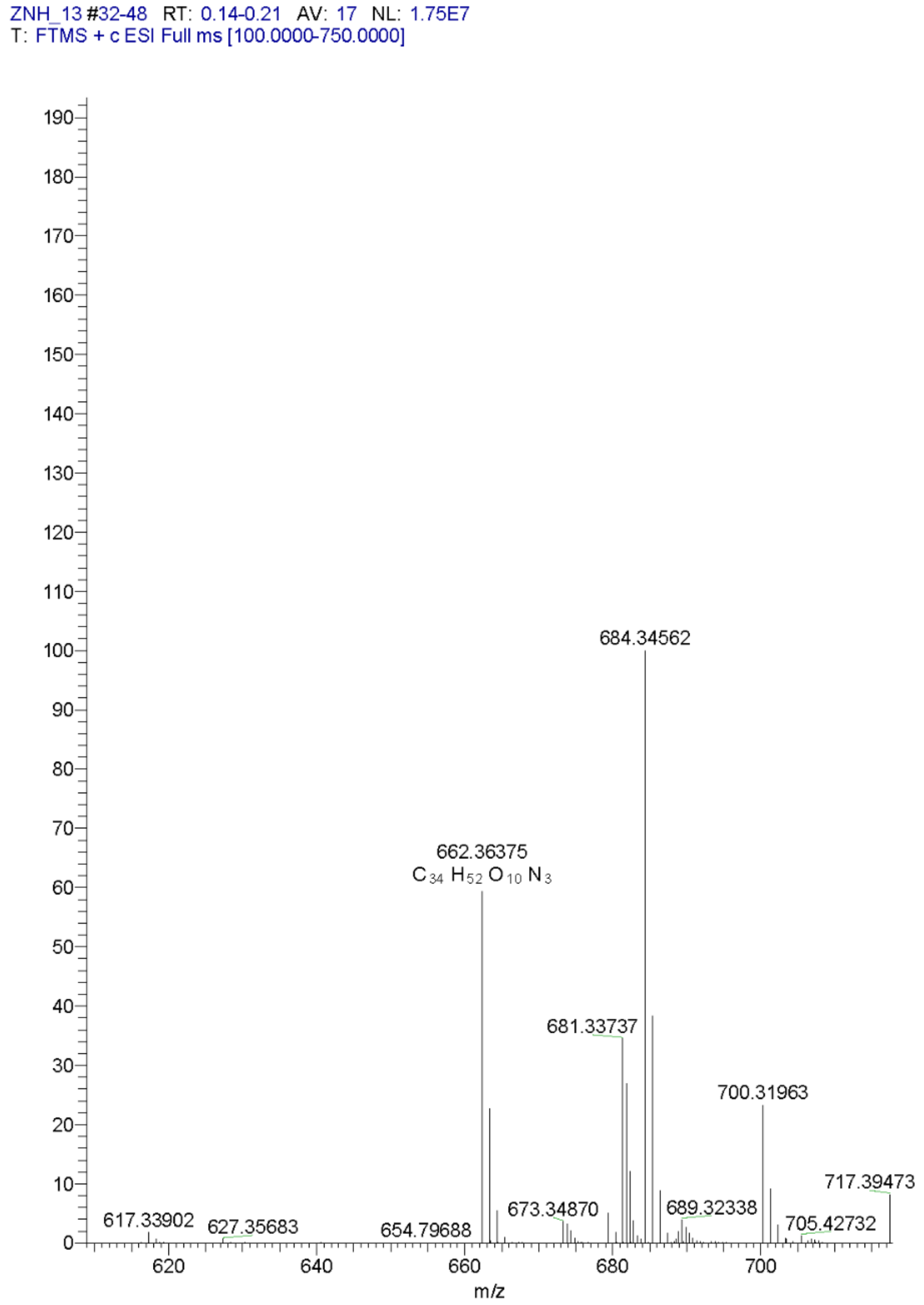

Elemental composition search on mass 662.36375

m/z= 657.36375-667.36375

m/z	Theo. Mass	Delta (ppm)	RDB equiv.	Composition
662.36375	662.36472	-1.47	10.5	$C_{34}H_{52}O_{10}N_3$

Compound **13**

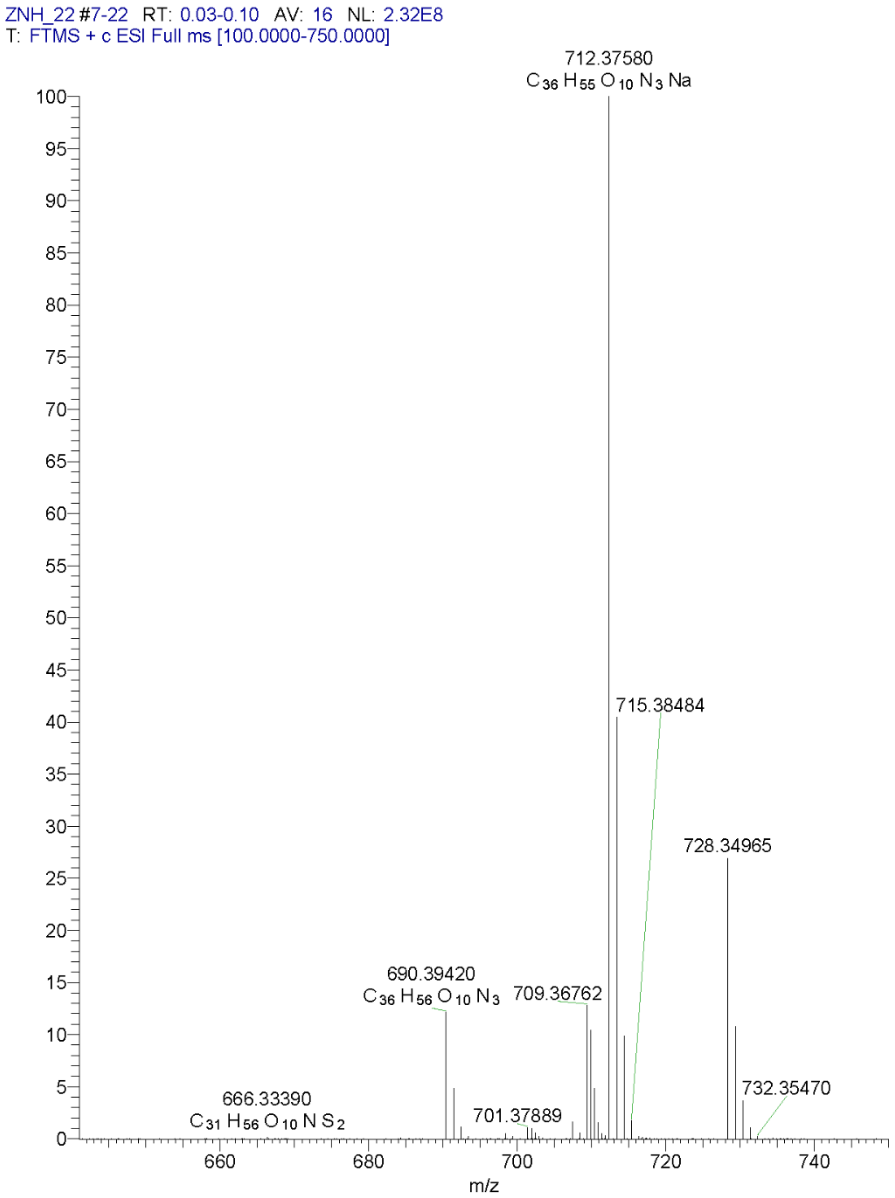

Elemental composition search on mass 690.39420

m/z= 685.39420-695.39420

m/z	Theo. Mass	Delta (ppm)	RDB equiv.	Composition
690.39420	690.39602	-2.64	10.5	$C_{36}H_{56}O_{10}N_3$

Compound **14**

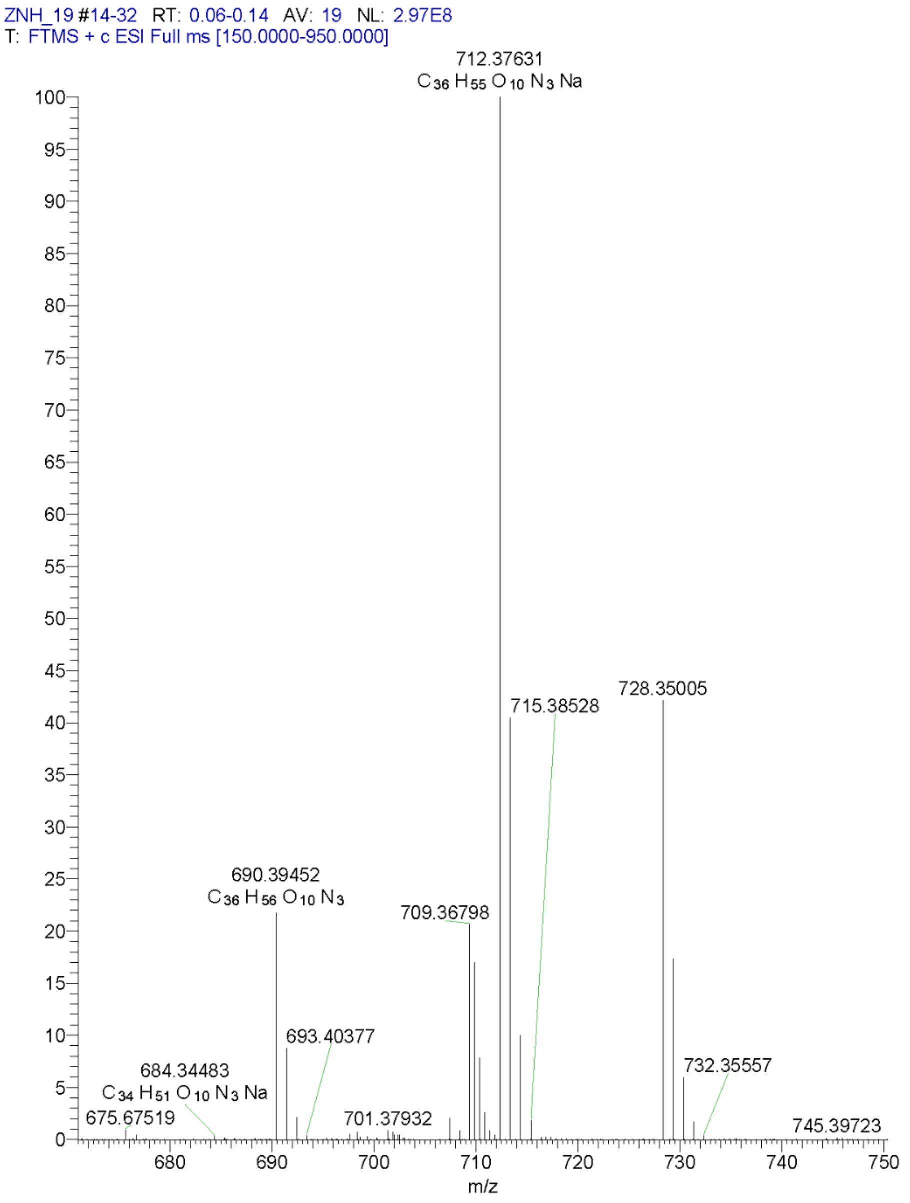

Elemental composition search on mass 690.39452

m/z= 685.39452-695.39452

m/z	Theo. Mass	Delta (ppm)	RDB equiv.	Composition
690.39452	690.39602	-2.17	10.5	$C_{36}H_{56}O_{10}N_3$

Compound **15**

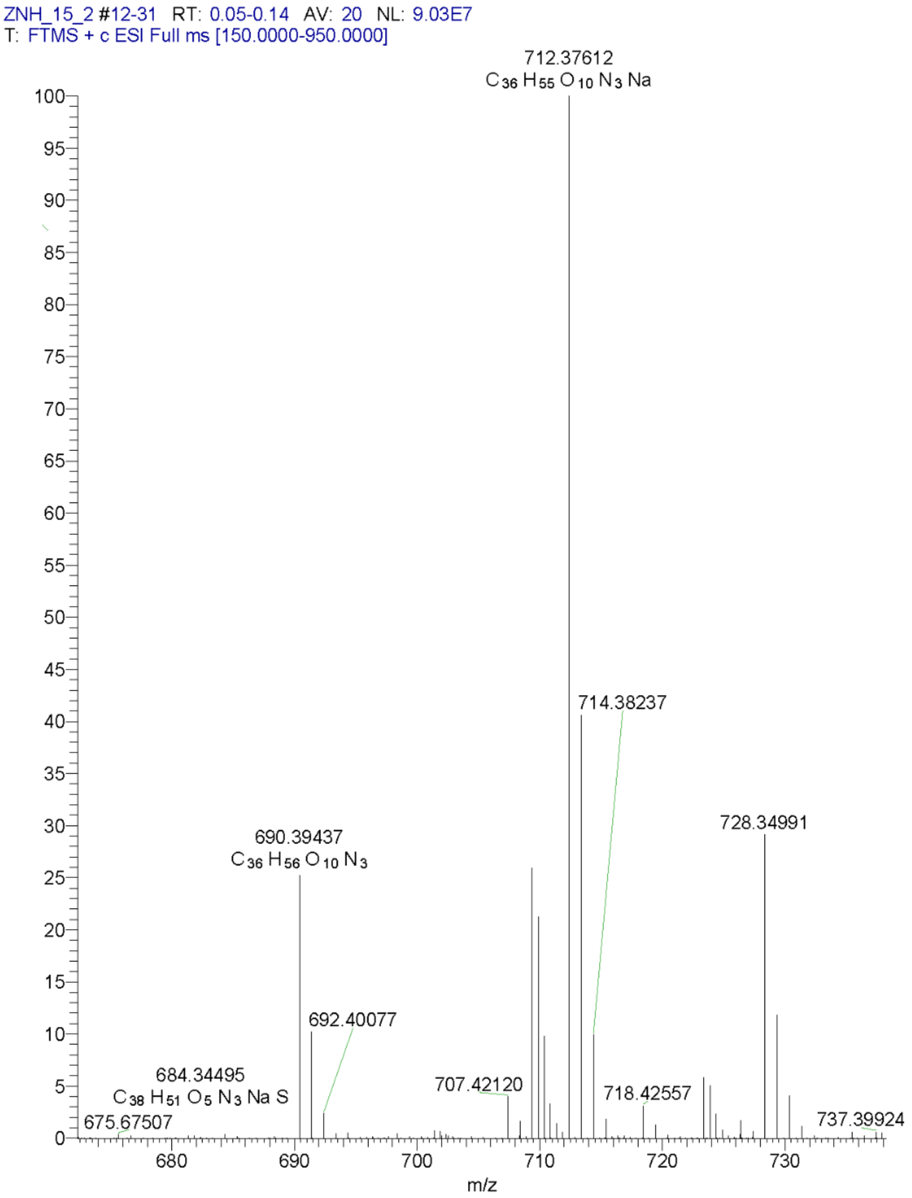

Elemental composition search on mass 690.39437

m/z= 685.39437-695.39437

m/z	Theo. Mass	Delta (ppm)	RDB equiv.	Composition
690.39437	690.39602	-2.39	10.5	$C_{36}H_{56}O_{10}N_3$

Compound **16**

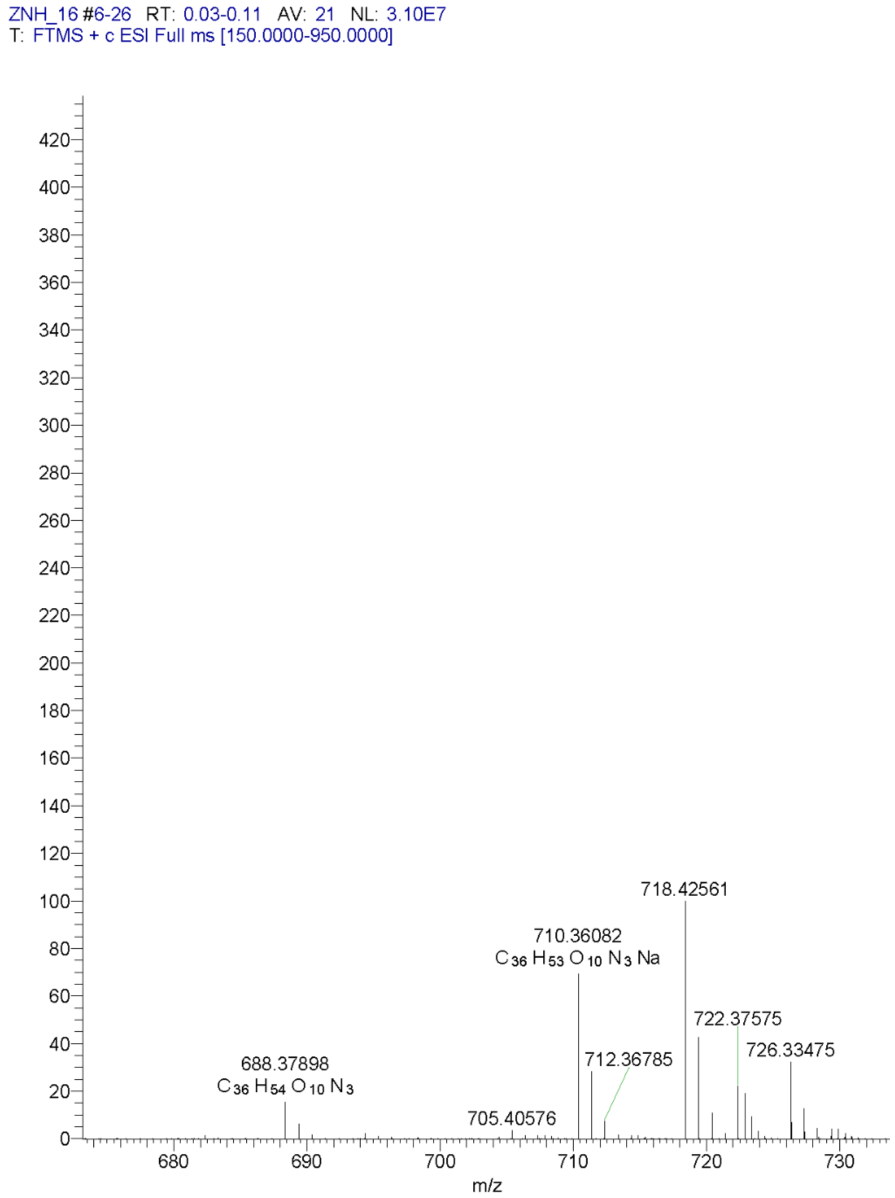

Elemental composition search on mass 688.37898

m/z= 683.37898-693.37898

m/z	Theo. Mass	Delta (ppm)	RDB equiv.	Composition
688.37898	688.38037	-2.02	11.5	$C_{36}H_{54}O_{10}N_3$

Compound **17**

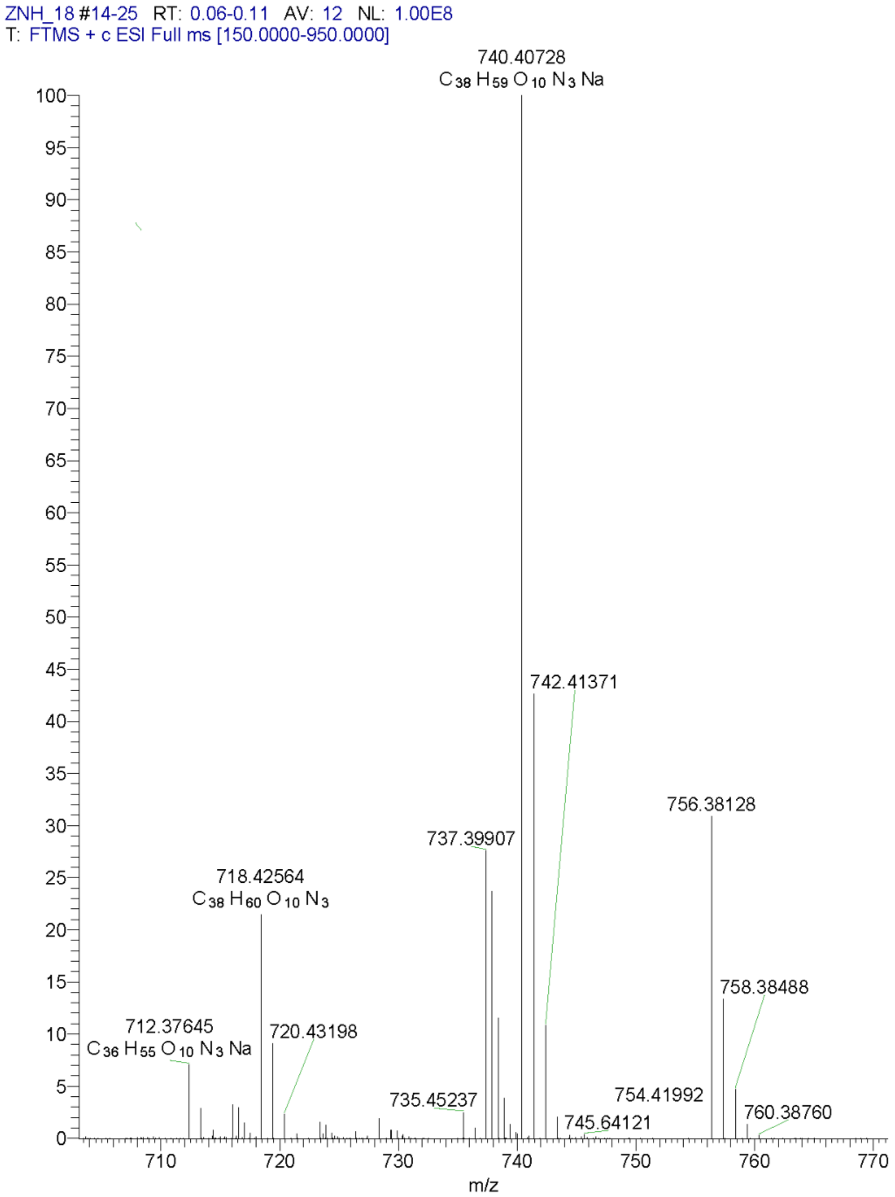

Elemental composition search on mass 718.42564

m/z= 713.42564-723.42564

m/z	Theo. Mass	Delta (ppm)	RDB equiv.	Composition
718.42564	718.42732	-2.34	10.5	$C_{38}H_{60}O_{10}N_3$

Compound **18**

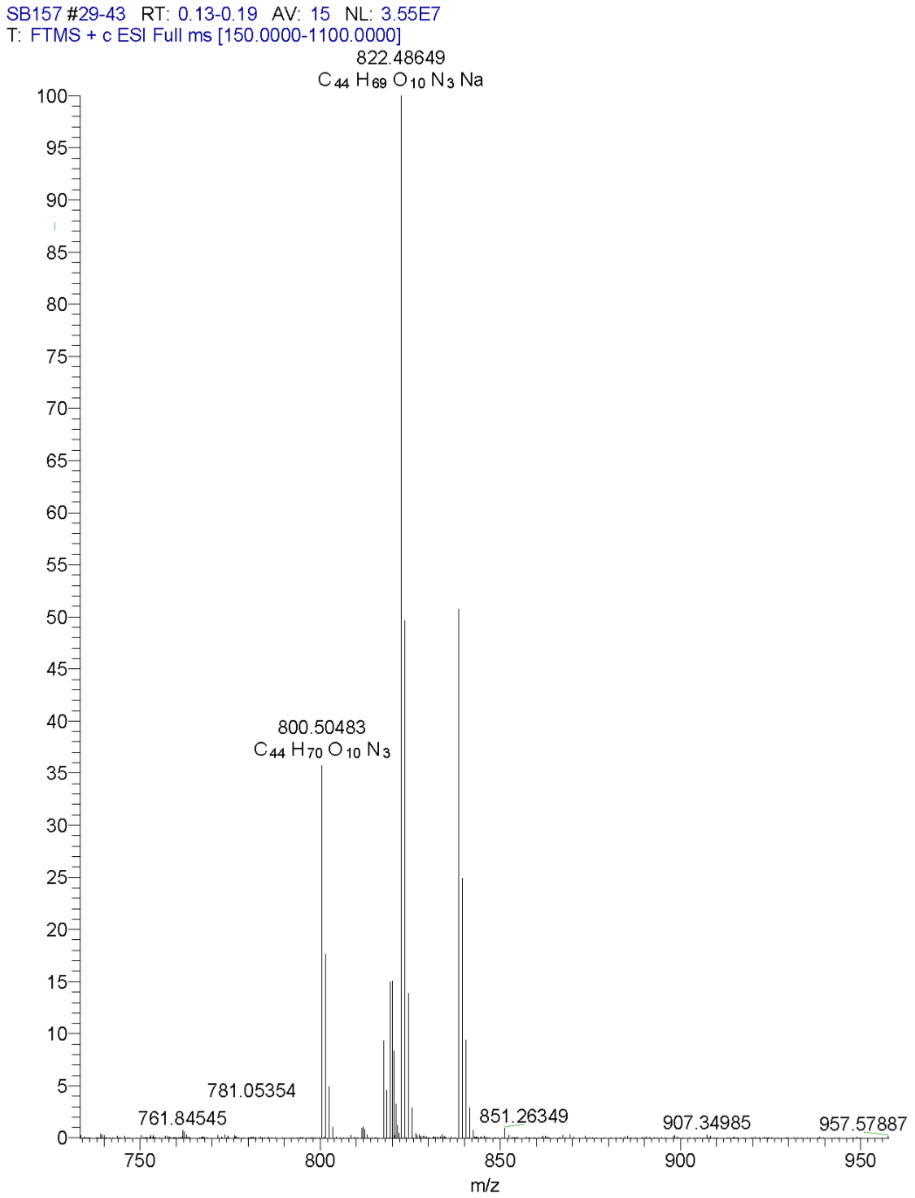

Elemental composition search on mass 800.50483

m/z= 795.50483-805.50483

m/z	Theo. Mass	Delta (ppm)	RDB equiv.	Composition
800.50483	800.50557	-0.93	11.5	$C_{44}H_{70}O_{10}N_3$

Compound **19**

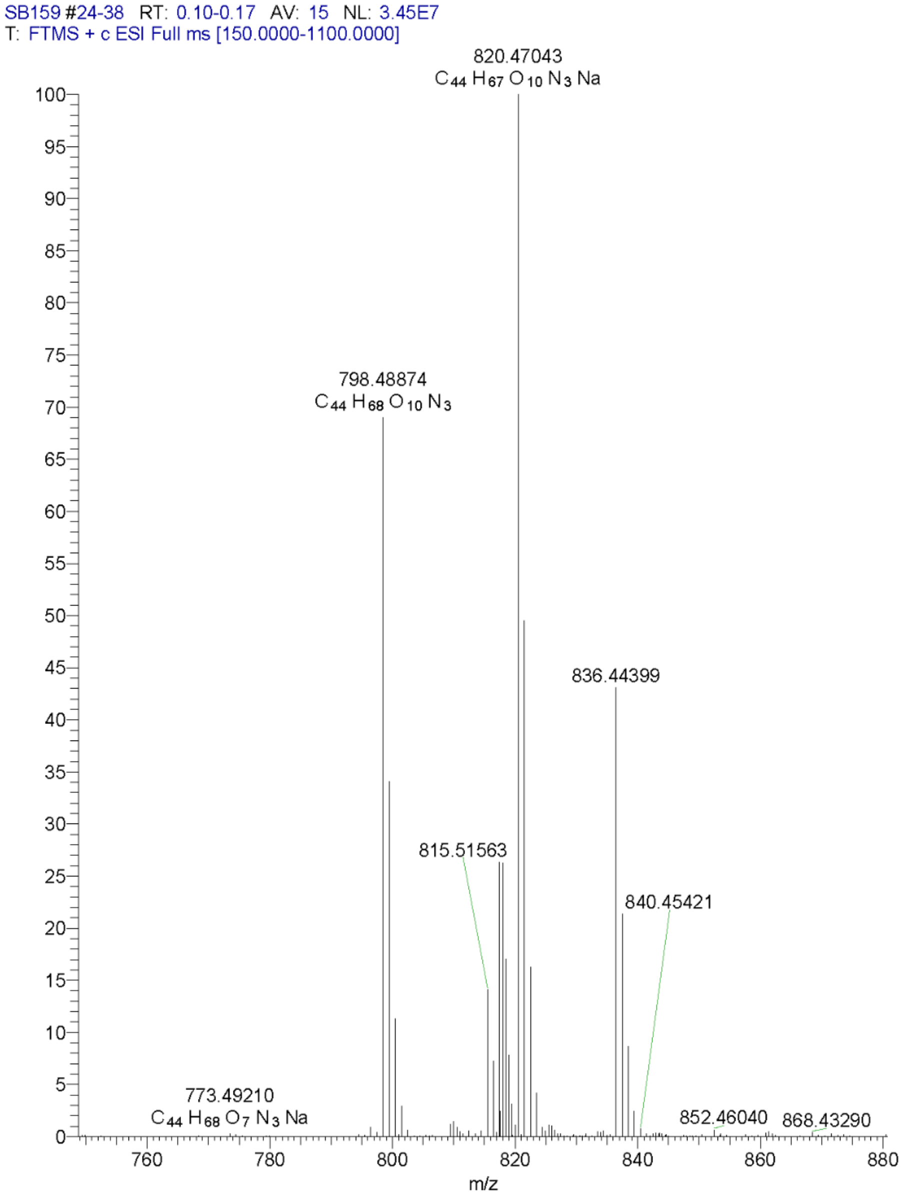

Elemental composition search on mass 798.48874

m/z= 793.48874-803.48874

m/z	Theo. Mass	Delta (ppm)	RDB equiv.	Composition
798.48874	798.48992	-1.48	12.5	$C_{44}H_{68}O_{10}N_3$

Compound **20**

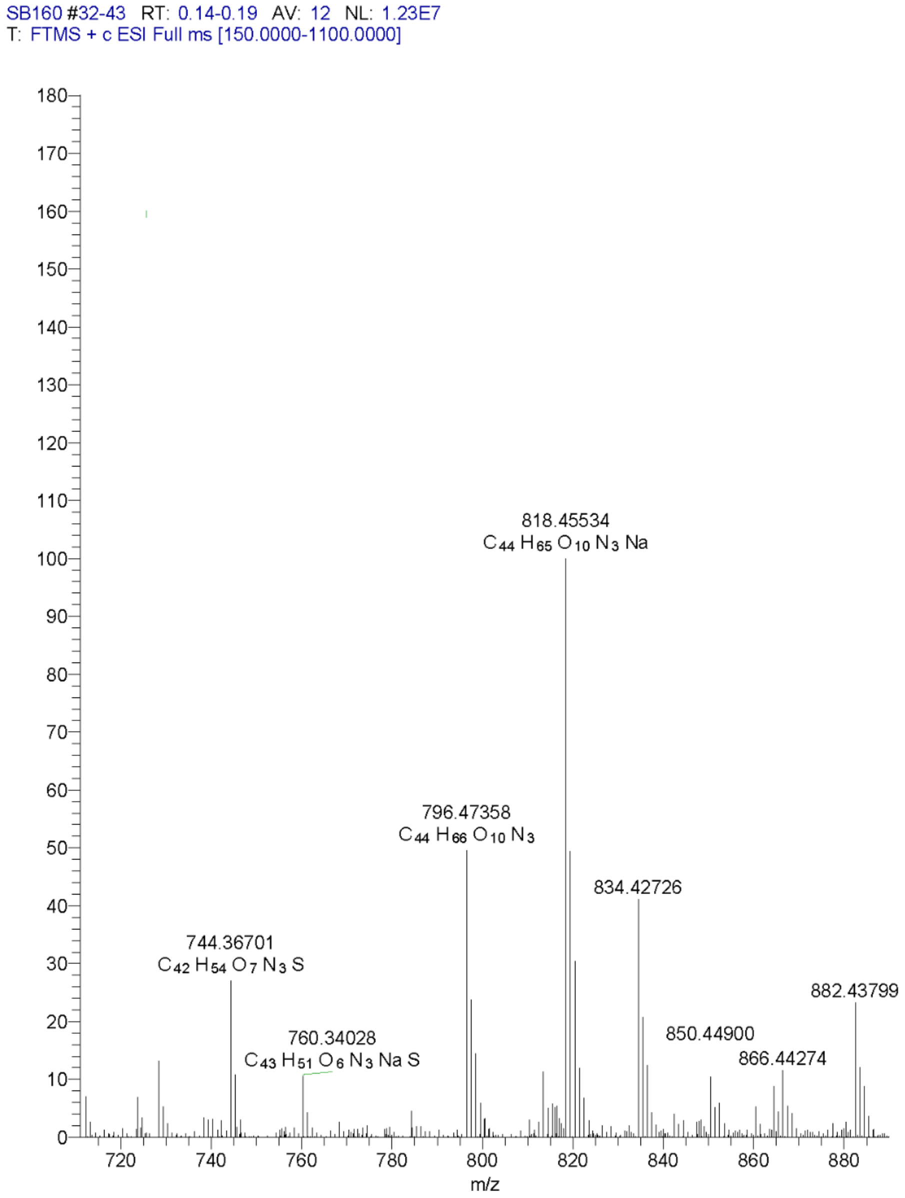

Elemental composition search on mass 796.47358

m/z= 791.47358-801.47358

m/z	Theo. Mass	Delta (ppm)	RDB equiv.	Composition
796.47358	796.47427	-0.87	13.5	$C_{44}H_{66}O_{10}N_3$

Compound **21**

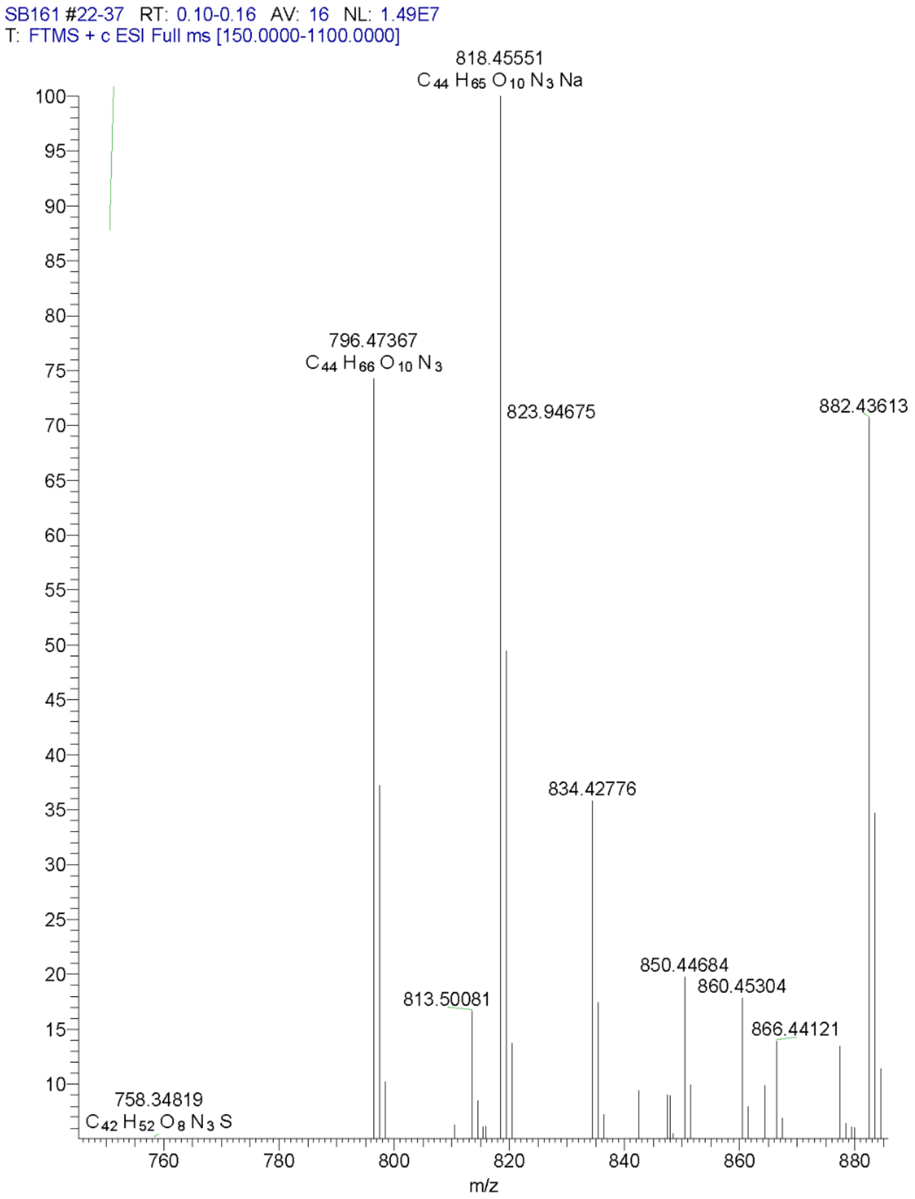

Elemental composition search on mass 796.47367

m/z= 791.47367-801.47367

m/z	Theo. Mass	Delta (ppm)	RDB equiv.	Composition
796.47367	796.47427	-0.76	13.5	$C_{44}H_{66}O_{10}N_3$

Compound **22**

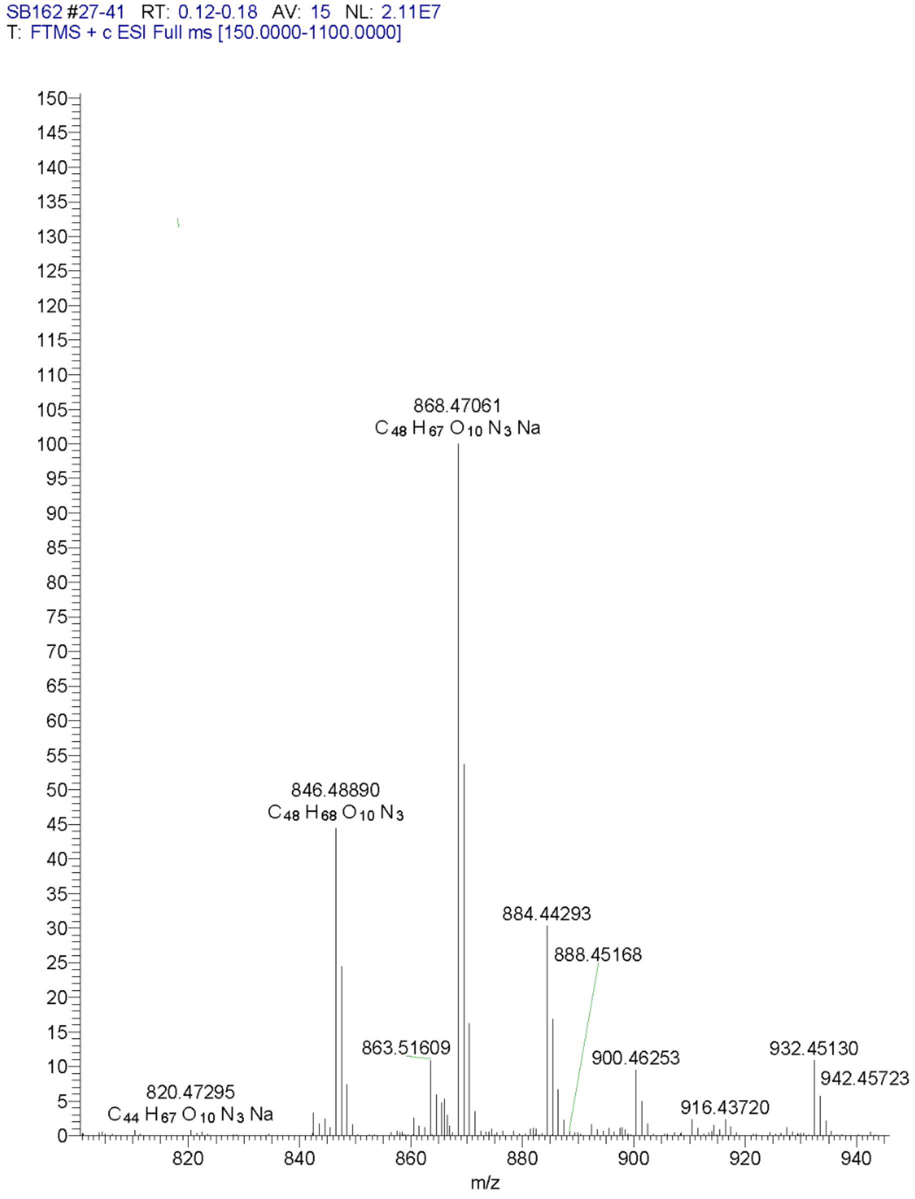

Elemental composition search on mass 846.48890

m/z= 841.48890-851.48890

m/z	Theo. Mass	Delta (ppm)	RDB equiv.	Composition
846.48890	846.48992	-1.21	16.5	$C_{48}H_{68}O_{10}N_3$

Compound **23**

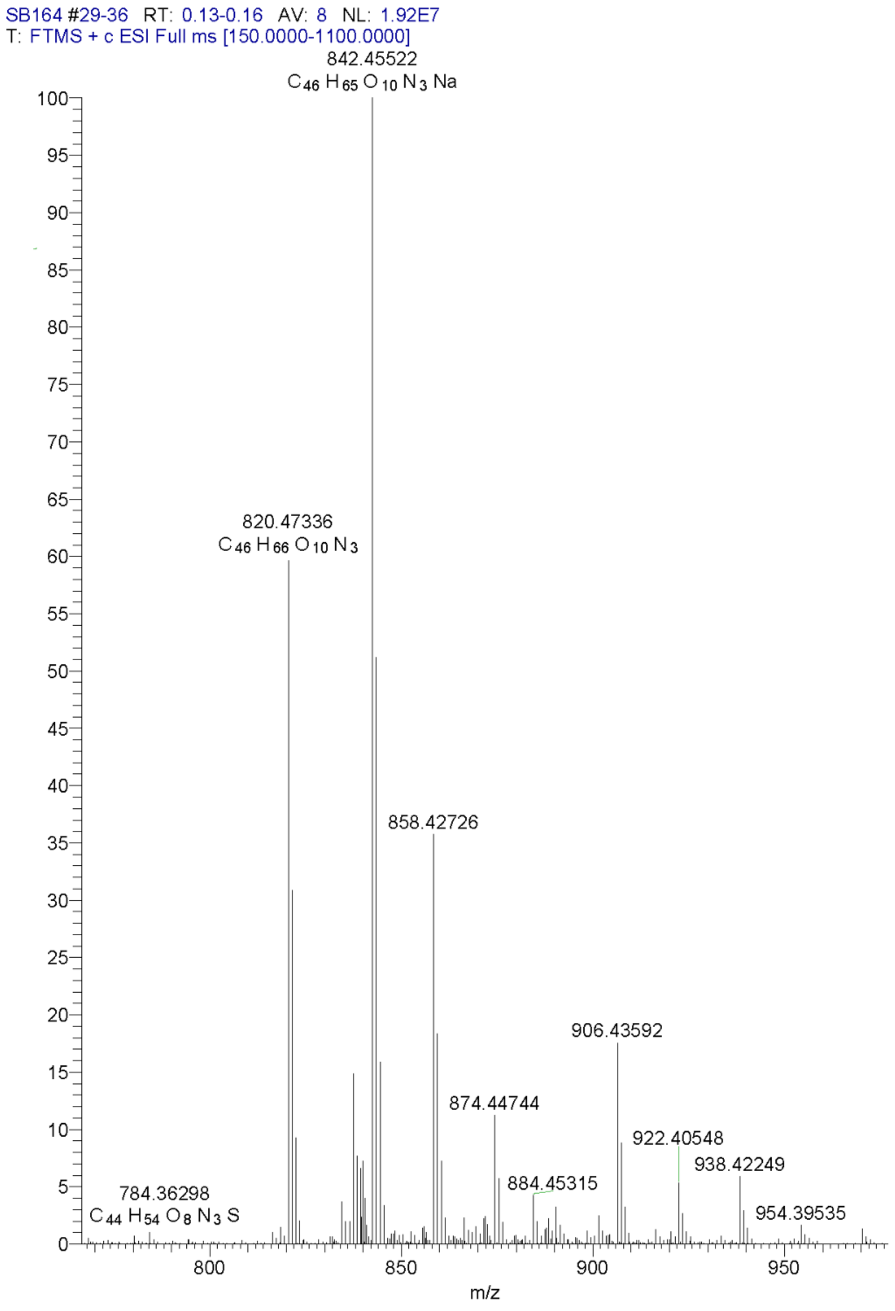

Elemental composition search on mass 820.47336

m/z= 815.47336-825.47336

m/z	Theo. Mass	Delta (ppm)	RDB equiv.	Composition
820.47336	820.47427	-1.11	15.5	$C_{46}H_{66}O_{10}N_3$

Compound **24**

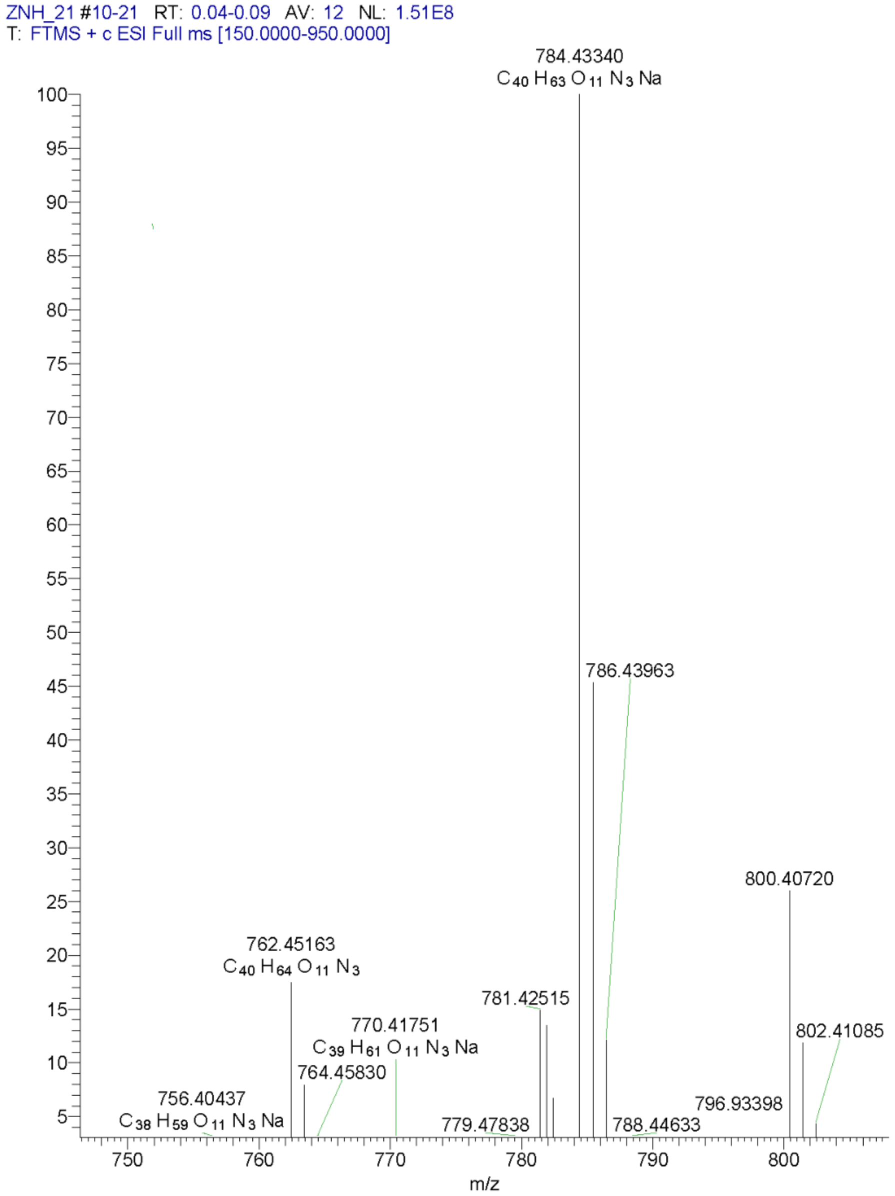

Elemental composition search on mass 762.45163

m/z= 757.45163-767.45163

m/z	Theo. Mass	Delta (ppm)	RDB equiv.	Composition
762.45163	762.45354	-2.50	10.5	$C_{40}H_{64}O_{11}N_3$

Compound **26**

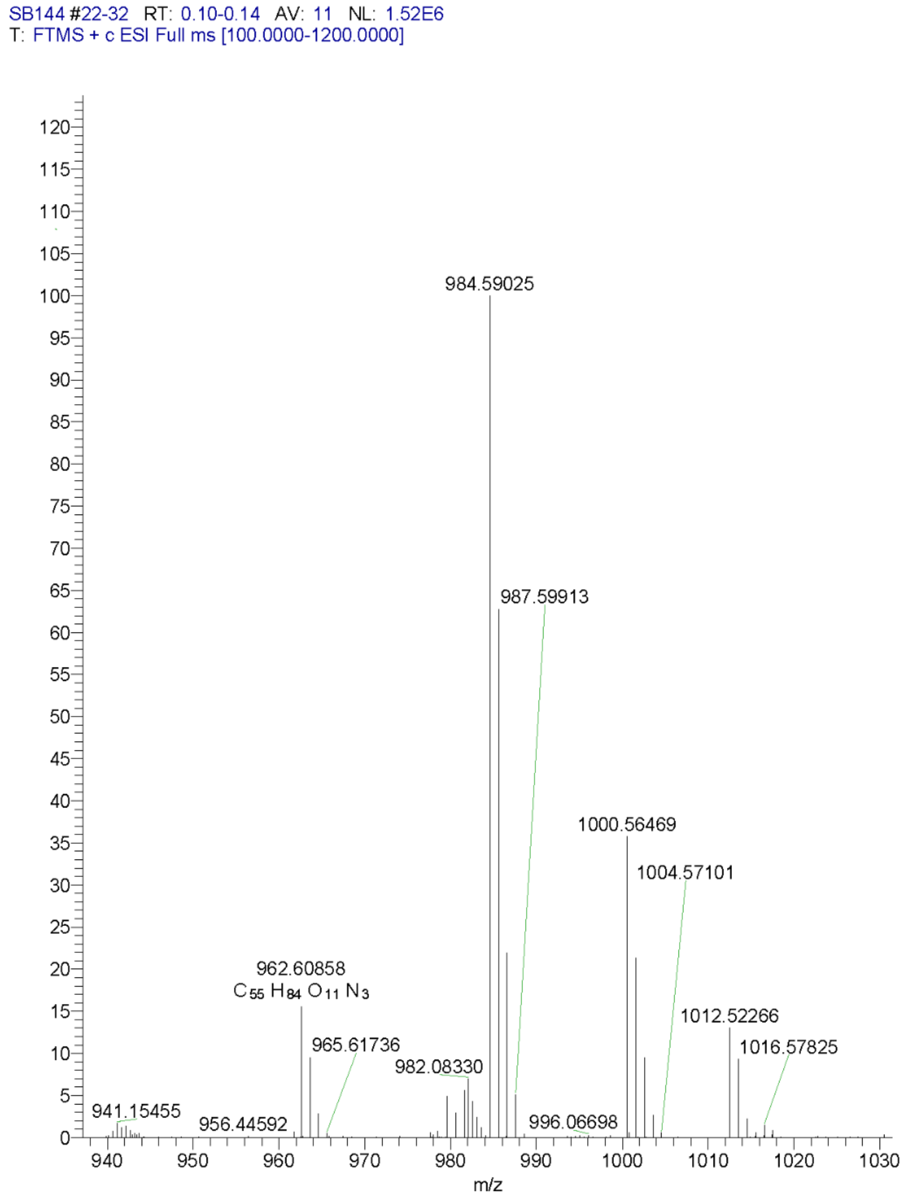

Elemental composition search on mass 962.60858

m/z= 957.60858-967.60858

m/z	Theo. Mass	Delta (ppm)	RDB equiv.	Composition
962.60858	962.61004	-1.51	15.5	$C_{55}H_{84}O_{11}N_3$

Compound **28**

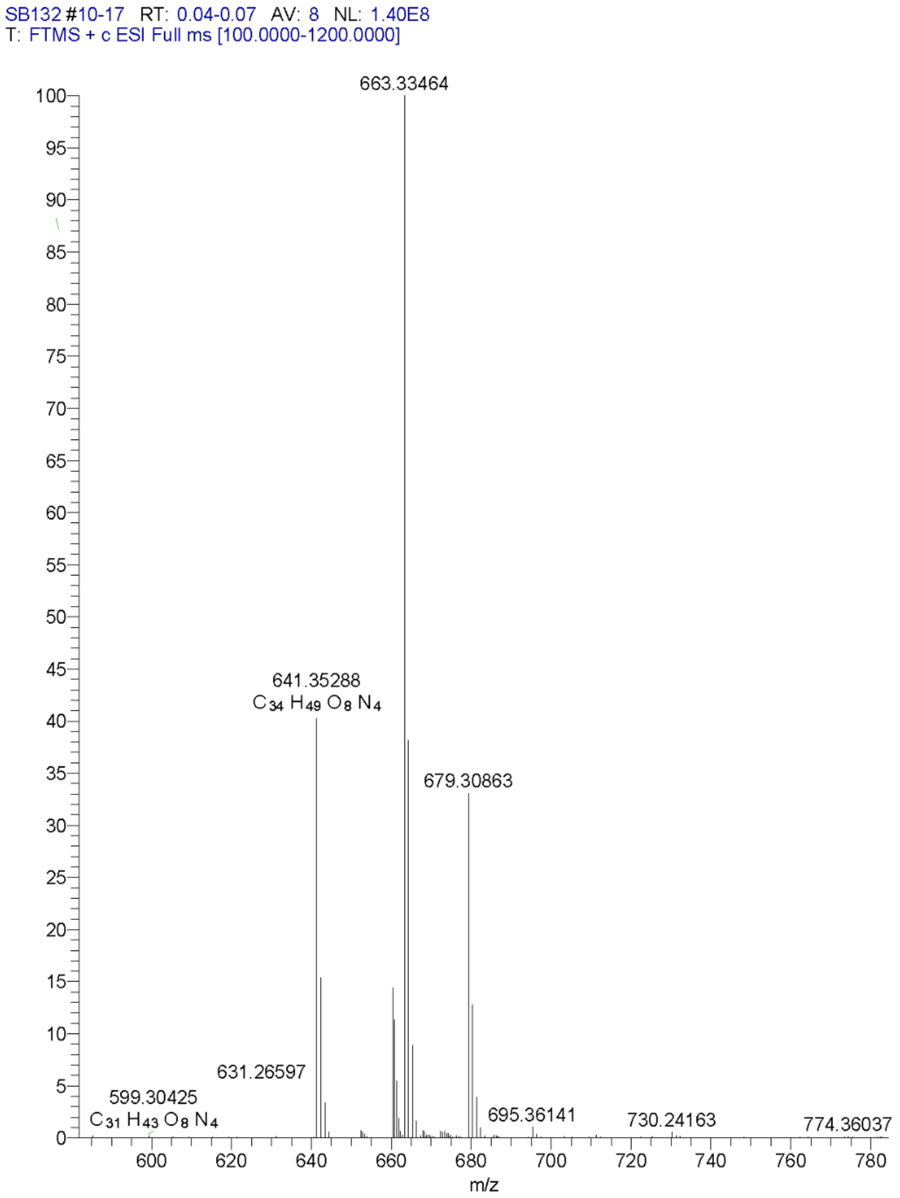

Elemental composition search on mass 641.35288

m/z= 636.35288-646.35288

m/z	Theo. Mass	Delta (ppm)	RDB equiv.	Composition
641.35288	641.35449	-2.51	12.5	$C_{34}H_{49}O_8N_4$

Compound **30**

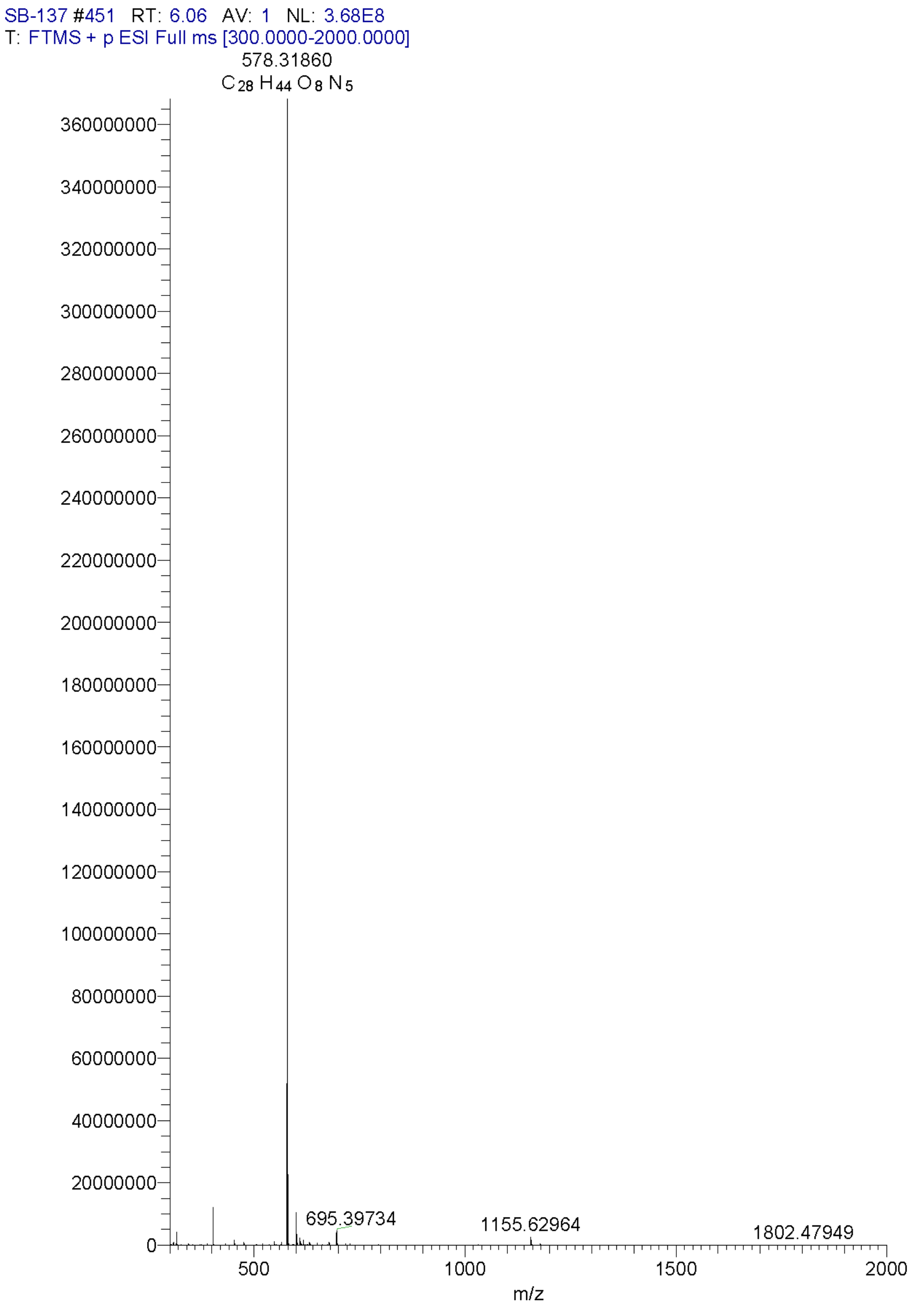

Elemental composition search on mass 578.31860

m/z= 573.31860-583.31860

m/z	Theo. Mass	Delta (ppm)	RDB equiv.	Composition
578.31860	578.31844	0.28	9.5	$C_{28}H_{44}O_8N_5$

Compound **32**

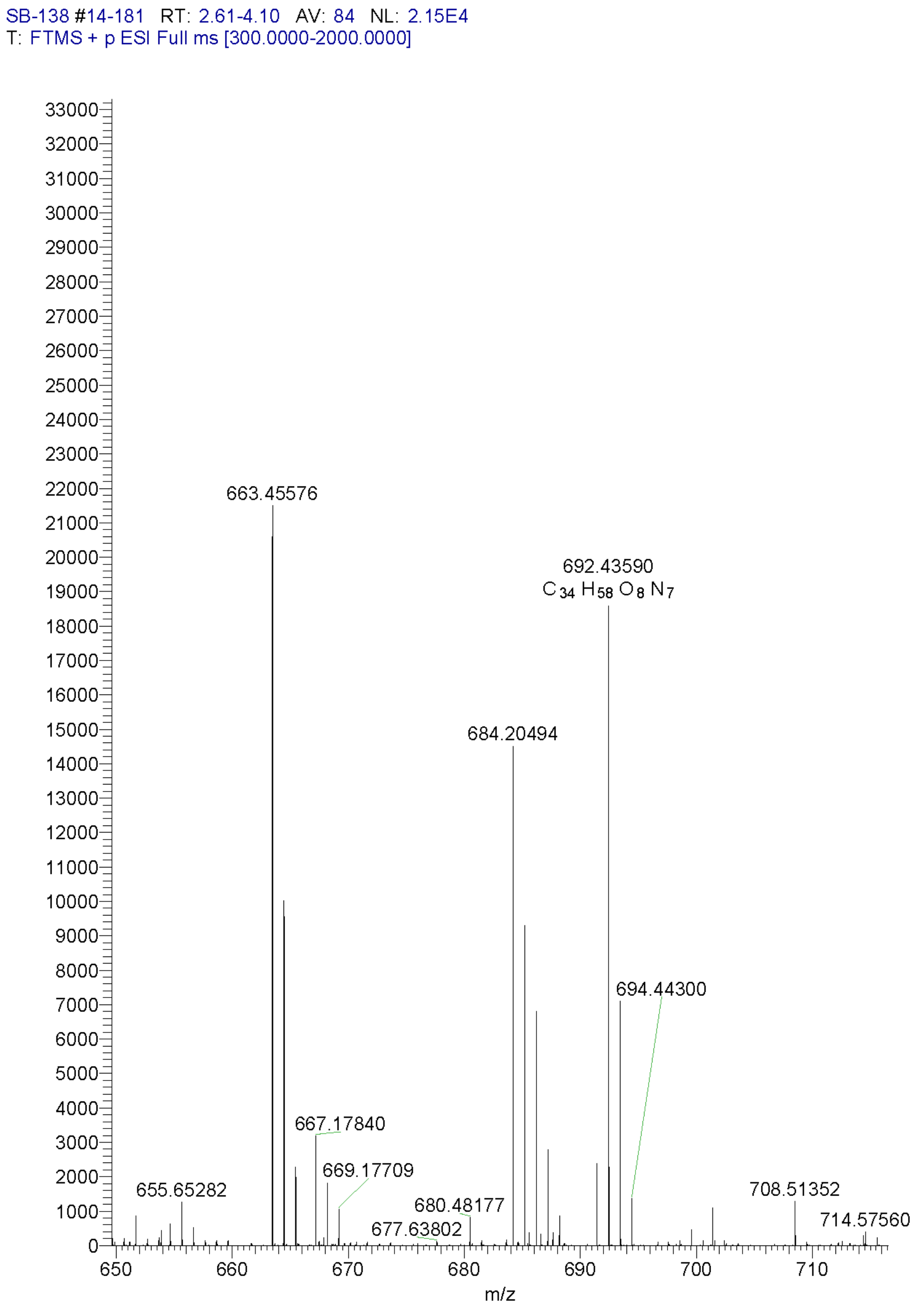

Elemental composition search on mass 692.43590

m/z= 687.43590-697.43590

m/z	Theo. Mass	Delta (ppm)	RDB equiv.	Composition
692.43590	692.43414	2.54	9.5	$C_{34}H_{58}O_8N_7$

Compound **37**

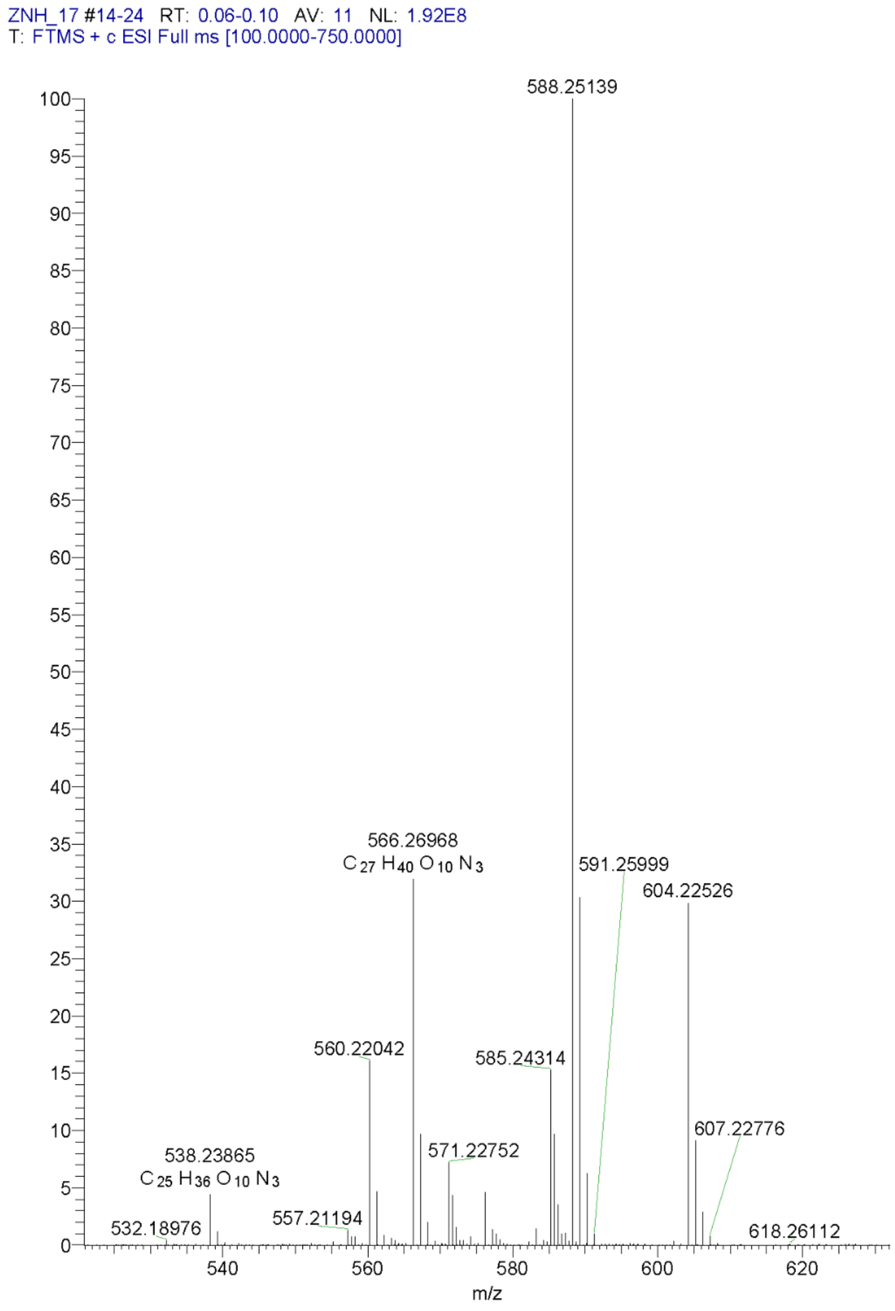

Elemental composition search on mass 566.26968

m/z= 561.26968-571.26968

m/z	Theo. Mass	Delta (ppm)	RDB equiv.	Composition
566.26968	566.27082	-2.01	9.5	$C_{27}H_{40}O_{10}N_3$

6. Representative UHPLC traces

Chromatogram and Results

Injection Details

Injection Name:	**Compound 3**	*Run Time (min):*	**12,50**
Vial Number:		*Injection Volume:*	**5,00**
Injection Type:		*Channel:*	**UV_VIS_2**
Calibration Level:		*Wavelength:*	**254**
Instrument Method:		*Bandwidth:*	
Processing Method:	**Quantitative**	*Dilution Factor:*	
Injection Date/Time:		*Sample Weight:*	

Chromatogram

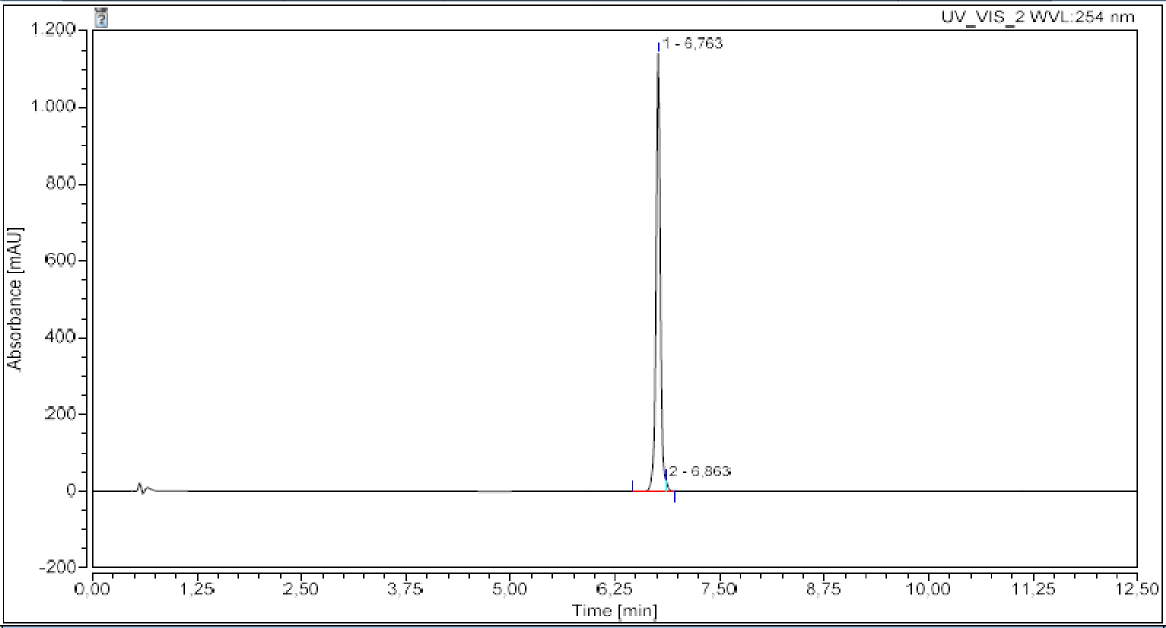

Integration Results

No.	Peak Name	Retention Time min	Area mAU*min	Height mAU	Relative Area %	Relative Height %	Amount n.a.
1		6,763	65,301	1141,127	98,67	97,88	n.a.
2		6,863	0,880	24,746	1,33	2,12	n.a.
**Total:**			**66,180**	**1165,872**	**100,00**	**100,00**	

Chromatogram and Results

Injection Details

Injection Name:	**Compound 6**	*Run Time (min):*	**12,50**
Vial Number:		*Injection Volume:*	**5,00**
Injection Type:		*Channel:*	**UV_VIS_2**
Calibration Level:		*Wavelength:*	**254**
Instrument Method:		*Bandwidth:*	
Processing Method:	**Quantitative**	*Dilution Factor:*	
Injection Date/Time:		*Sample Weight:*	

Chromatogram

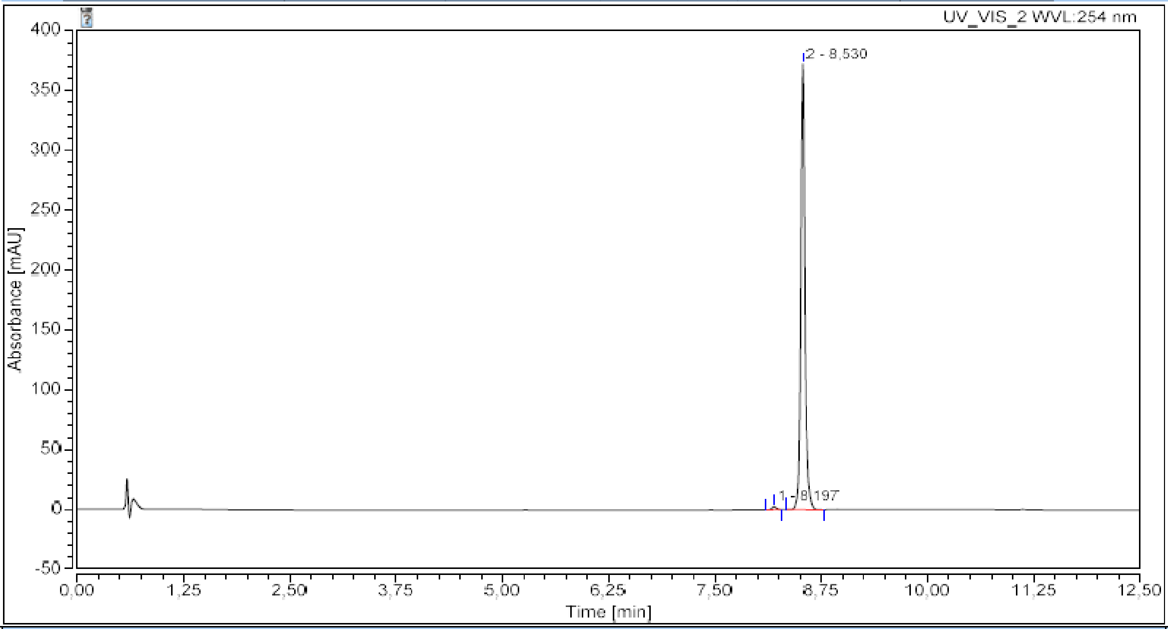

Integration Results

No.	Peak Name	Retention Time min	Area mAU*min	Height mAU	Relative Area %	Relative Height %	Amount n.a.
1		8,197	0,155	2,931	0,73	0,78	n.a.
2		8,530	21,057	372,341	99,27	99,22	n.a.
**Total:**			**21,212**	**375,272**	**100,00**	**100,00**	

Chromatogram and Results

Injection Details

Injection Name:	**Compound 7**	*Run Time (min):*	**12,50**
Vial Number:		*Injection Volume:*	**5,00**
Injection Type:		*Channel:*	**UV_VIS_2**
Calibration Level:		*Wavelength:*	**254**
Instrument Method:		*Bandwidth:*	
Processing Method:	**Quantitative**	*Dilution Factor:*	
Injection Date/Time:		*Sample Weight:*	

Chromatogram

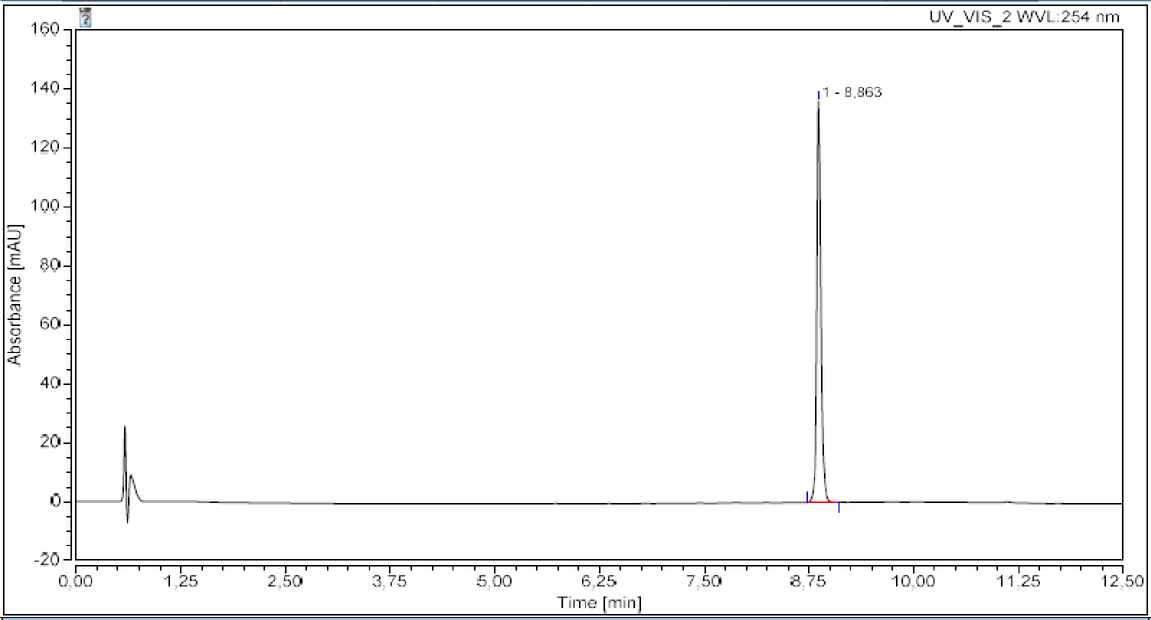

Integration Results

No.	Peak Name	Retention Time<br>min	Area<br>mAU*min	Height<br>mAU	Relative Area<br>%	Relative Height<br>%	Amount<br>n.a.
1		8,863	7,518	135,910	100,00	100,00	n.a.
**Total:**			**7,518**	**135,910**	**100,00**	**100,00**	

Chromatogram and Results

Injection Details

Injection Name:	**Compound 14**	*Run Time (min):*	**12,50**
Vial Number:		*Injection Volume:*	**5,00**
Injection Type:		*Channel:*	**UV_VIS_2**
Calibration Level:		*Wavelength:*	**254**
Instrument Method:		*Bandwidth:*	
Processing Method:	**Quantitative**	*Dilution Factor:*	
Injection Date/Time:		*Sample Weight:*	

Chromatogram

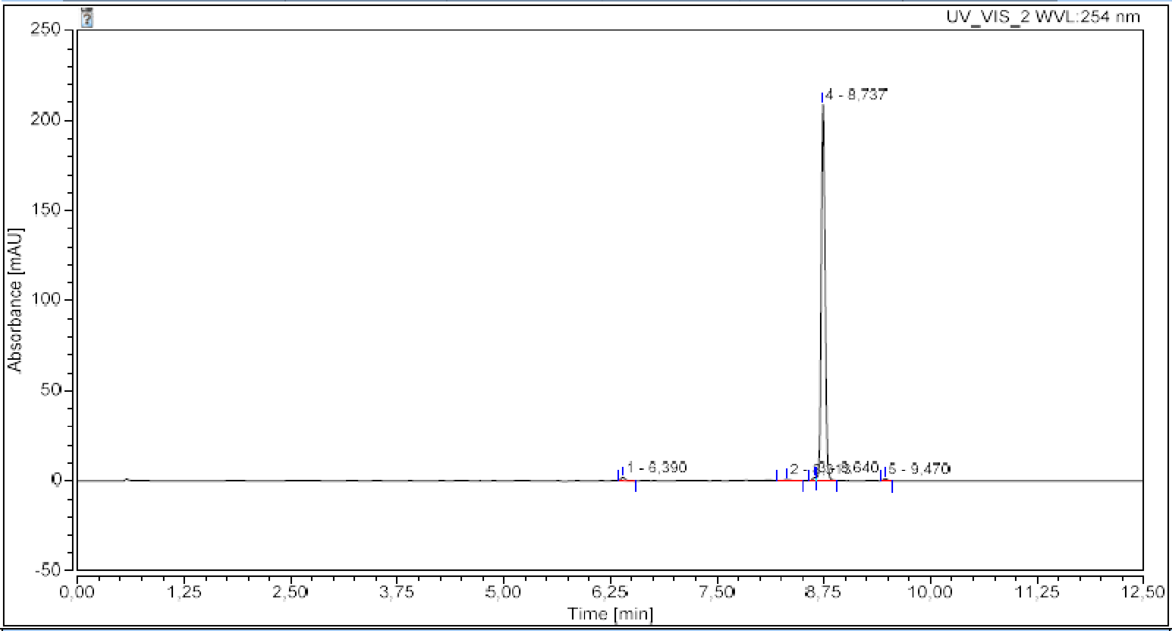

Integration Results

No.	Peak Name	Retention Time min	Area mAU*min	Height mAU	Relative Area %	Relative Height %	Amount n.a.
1		6,390	0,102	1,908	0,87	0,89	n.a.
2		8,313	0,051	0,517	0,43	0,24	n.a.
3		8,640	0,077	1,731	0,66	0,81	n.a.
4		8,737	11,493	208,724	97,52	97,49	n.a.
5		9,470	0,061	1,213	0,52	0,57	n.a.
**Total:**			**11,786**	**214,092**	**100,00**	**100,00**	

Chromatogram and Results

Injection Details

Injection Name:	**Compound 16**	*Run Time (min):*	**12,50**
Vial Number:		*Injection Volume:*	**5,00**
Injection Type:		*Channel:*	**UV_VIS_2**
Calibration Level:		*Wavelength:*	**254**
Instrument Method:		*Bandwidth:*	
Processing Method:	**Quantitative**	*Dilution Factor:*	
Injection Date/Time:		*Sample Weight:*	

Chromatogram

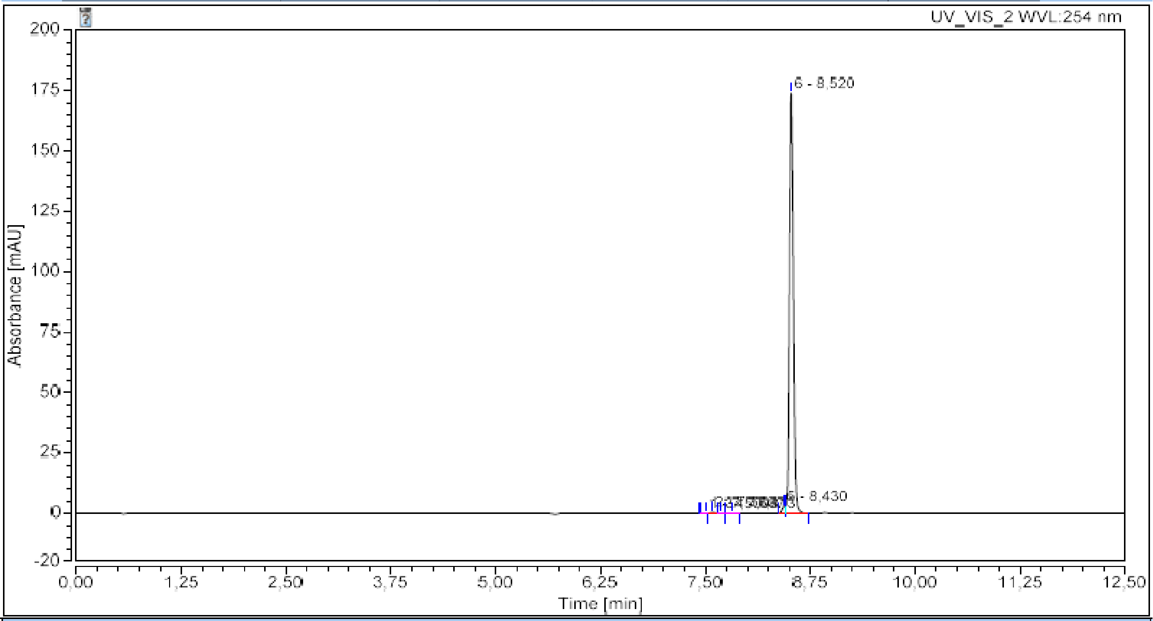

Integration Results

No.	Peak Name	Retention Time min	Area mAU*min	Height mAU	Relative Area %	Relative Height %	Amount n.a.
1		7,500	0,002	0,031	0,02	0,02	n.a.
2		7,580	0,028	0,419	0,29	0,24	n.a.
3		7,687	0,001	0,018	0,01	0,01	n.a.
4		7,813	0,004	0,059	0,04	0,03	n.a.
5		8,430	0,105	2,684	1,06	1,52	n.a.
6		8,520	9,703	173,903	98,58	98,19	n.a.
**Total:**			**9,843**	**177,115**	**100,00**	**100,00**	

Chromatogram and Results

Injection Details

Injection Name:	**Compound 17**	*Run Time (min):*	**12,50**
Vial Number:		*Injection Volume:*	**5,00**
Injection Type:		*Channel:*	**UV_VIS_2**
Calibration Level:		*Wavelength:*	**254**
Instrument Method:		*Bandwidth:*	
Processing Method:	**Quantitative**	*Dilution Factor:*	
Injection Date/Time:		*Sample Weight:*	

Chromatogram

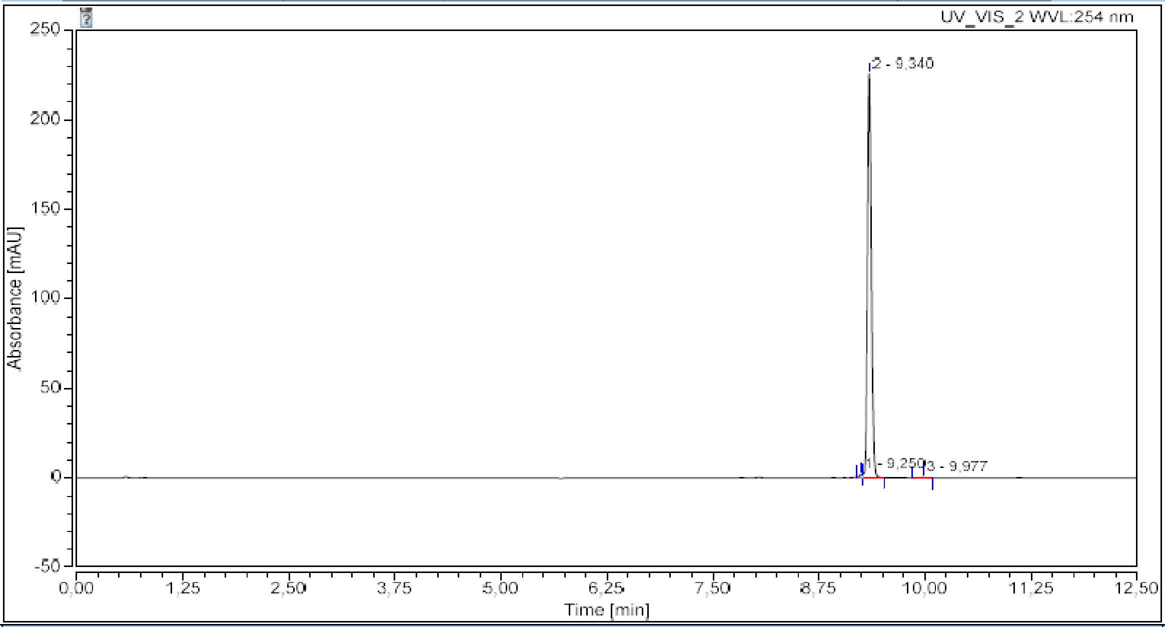

Integration Results

No.	Peak Name	Retention Time min	Area mAU*min	Height mAU	Relative Area %	Relative Height %	Amount n.a.
1		9,250	0,067	1,717	0,54	0,75	n.a.
2		9,340	12,278	225,574	99,23	99,04	n.a.
3		9,977	0,028	0,465	0,23	0,20	n.a.
**Total:**			**12,373**	**227,756**	**100,00**	**100,00**	

Chromatogram and Results

Injection Details

Injection Name:	**Compound 21**	*Run Time (min):*	**12,50**
Vial Number:		*Injection Volume:*	**5,00**
Injection Type:		*Channel:*	**UV_VIS_2**
Calibration Level:		*Wavelength:*	**254**
Instrument Method:		*Bandwidth:*	
Processing Method:	**Quantitative**	*Dilution Factor:*	
Injection Date/Time:		*Sample Weight:*	

Chromatogram

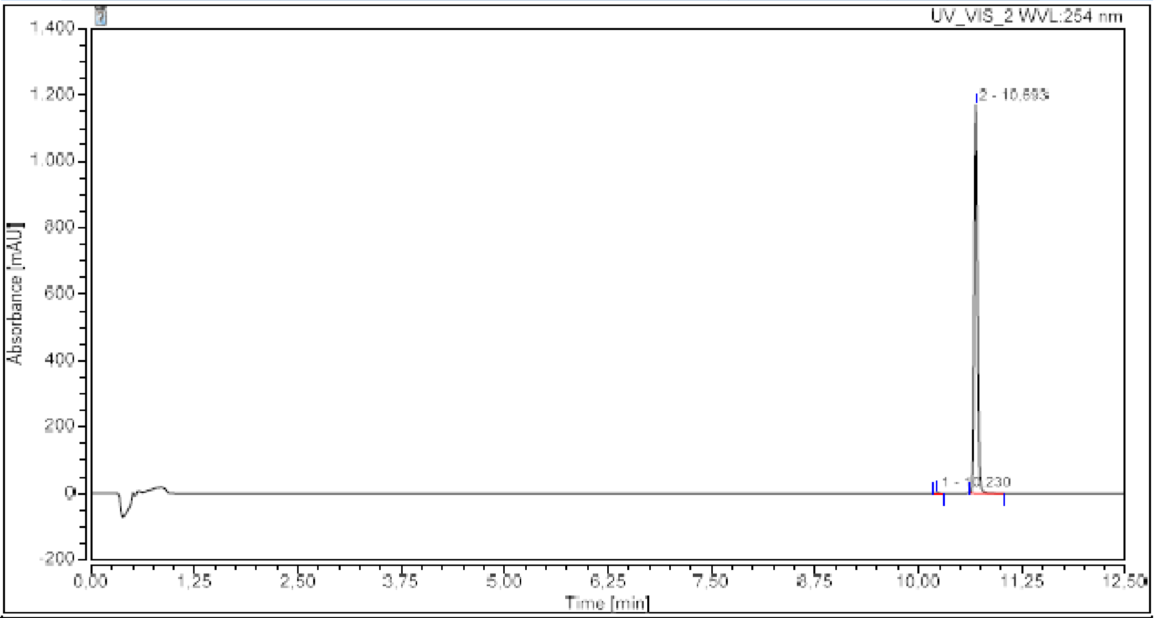

Integration Results

No.	Peak Name	Retention Time min	Area mAU*min	Height mAU	Relative Area %	Relative Height %	Amount n.a.
1		10,230	0,129	2,924	0,24	0,25	n.a.
2		10,693	52,444	1170,006	99,76	99,75	n.a.
**Total:**			**52,572**	**1172,930**	**100,00**	**100,00**	

Chromatogram and Results

Injection Details

Injection Name:	**Compound 22**	*Run Time (min):*	**12,50**
Vial Number:		*Injection Volume:*	**5,00**
Injection Type:		*Channel:*	**UV_VIS_2**
Calibration Level:		*Wavelength:*	**254**
Instrument Method:		*Bandwidth:*	
Processing Method:	**Quantitative**	*Dilution Factor:*	
Injection Date/Time:		*Sample Weight:*	

Chromatogram

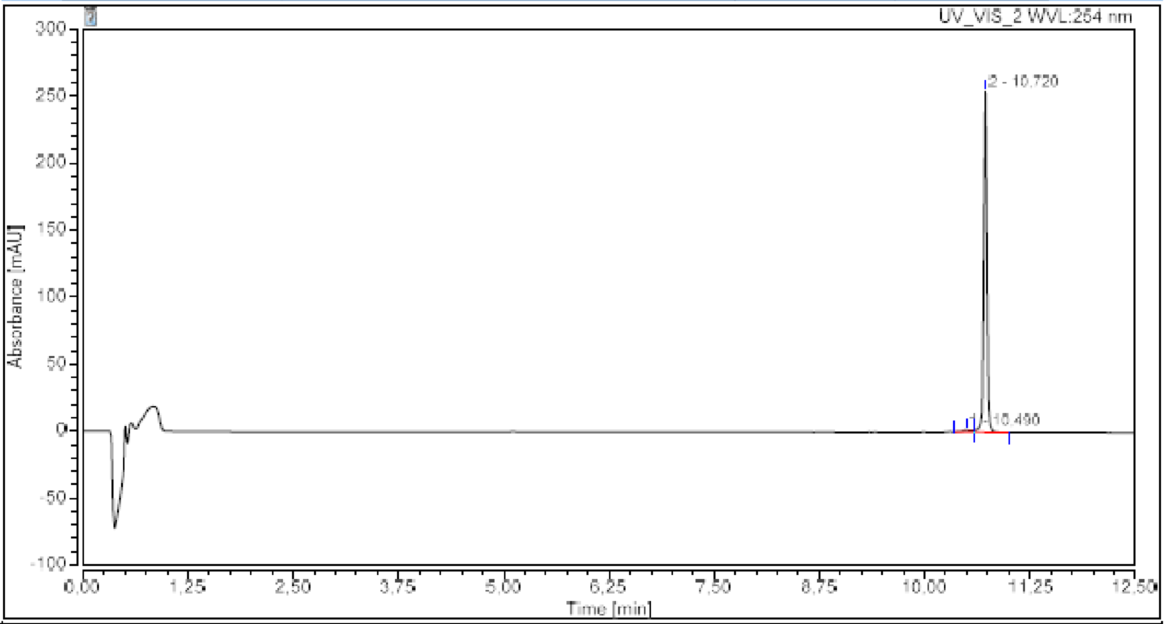

Integration Results

No.	Peak Name	Retention Time min	Area mAU*min	Height mAU	Relative Area %	Relative Height %	Amount n.a.
1		10,490	0,177	1,751	1,54	0,68	n.a.
2		10,720	11,322	254,214	98,46	99,32	n.a.
**Total:**			**11,499**	**255,964**	**100,00**	**100,00**	

Chromatogram and Results

Injection Details

Injection Name:	**Compound 23**	*Run Time (min):*	**12,50**
Vial Number:		*Injection Volume:*	**5,00**
Injection Type:		*Channel:*	**UV_VIS_2**
Calibration Level:		*Wavelength:*	**254**
Instrument Method:		*Bandwidth:*	
Processing Method:	**Quantitative**	*Dilution Factor:*	
Injection Date/Time:		*Sample Weight:*	

Chromatogram

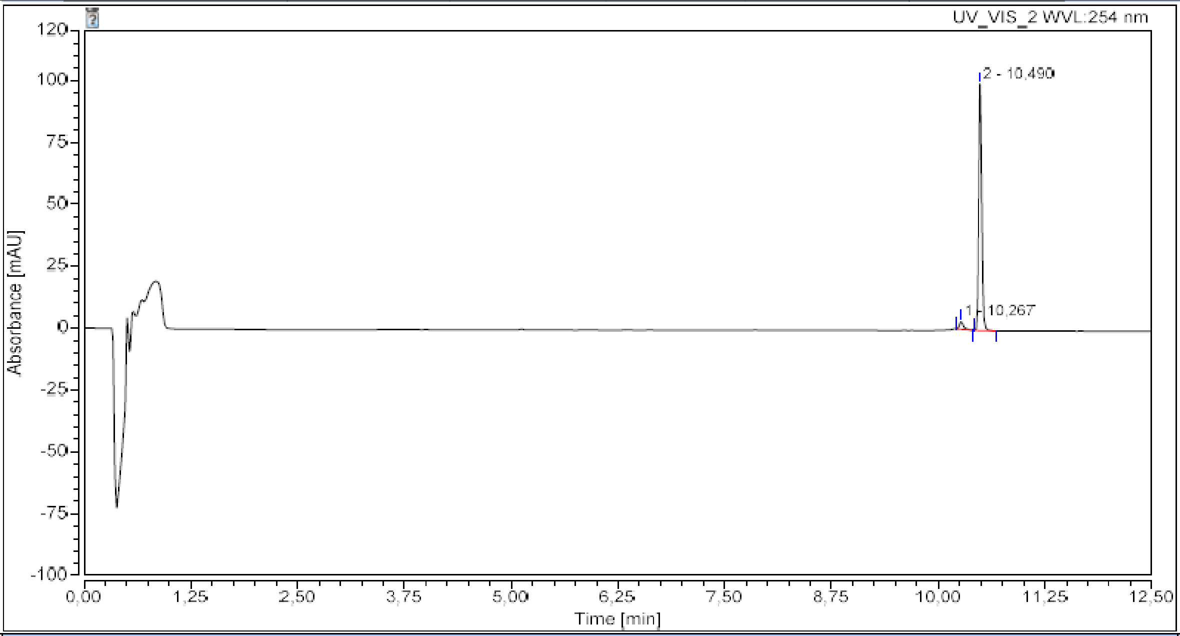

Integration Results

No.	Peak Name	Retention Time min	Area mAU*min	Height mAU	Relative Area %	Relative Height %	Amount n.a.
1		10,267	0,166	3,092	3,75	3,01	n.a.
2		10,490	4,249	99,547	96,25	96,99	n.a.
**Total:**			**4,415**	**102,638**	**100,00**	**100,00**	
